# Targeting CDK12 disrupts estrogen-receptor chromatin recruitment and ER-MED1 transcription in advanced ER+ breast cancer

**DOI:** 10.1093/jnci/djaf295

**Published:** 2025-10-15

**Authors:** Daniela Ottaviani, Mihaela Ola, Alessandra Allotta, Yasmine Maati Chaibi, Seán Hickey, Petra Jagust, Nicola Cosgrove, Sinéad Cocchiglia, Fiona Bane, Ramón Fallon, Gordon Daly, Aisling Hegarty, Lance Hudson, Katherine Sheehan, Shannon Kalsi, Stephen Shovlin, Aoibhín Powell, Ash Bahl, Ed Ainscow, Steffi Oesterreich, Adrian V Lee, Fergus J Couch, Arnold D K Hill, Damir Varešlija, Leonie Young

**Affiliations:** Endocrine Oncology Research Group, Department of Surgery, Royal College of Surgeons in Ireland, University of Medicine and Health Sciences, Dublin, Ireland; Endocrine Oncology Research Group, Department of Surgery, Royal College of Surgeons in Ireland, University of Medicine and Health Sciences, Dublin, Ireland; Endocrine Oncology Research Group, Department of Surgery, Royal College of Surgeons in Ireland, University of Medicine and Health Sciences, Dublin, Ireland; Endocrine Oncology Research Group, Department of Surgery, Royal College of Surgeons in Ireland, University of Medicine and Health Sciences, Dublin, Ireland; Endocrine Oncology Research Group, Department of Surgery, Royal College of Surgeons in Ireland, University of Medicine and Health Sciences, Dublin, Ireland; Endocrine Oncology Research Group, Department of Surgery, Royal College of Surgeons in Ireland, University of Medicine and Health Sciences, Dublin, Ireland; Endocrine Oncology Research Group, Department of Surgery, Royal College of Surgeons in Ireland, University of Medicine and Health Sciences, Dublin, Ireland; Endocrine Oncology Research Group, Department of Surgery, Royal College of Surgeons in Ireland, University of Medicine and Health Sciences, Dublin, Ireland; Endocrine Oncology Research Group, Department of Surgery, Royal College of Surgeons in Ireland, University of Medicine and Health Sciences, Dublin, Ireland; Endocrine Oncology Research Group, Department of Surgery, Royal College of Surgeons in Ireland, University of Medicine and Health Sciences, Dublin, Ireland; Endocrine Oncology Research Group, Department of Surgery, Royal College of Surgeons in Ireland, University of Medicine and Health Sciences, Dublin, Ireland; Endocrine Oncology Research Group, Department of Surgery, Royal College of Surgeons in Ireland, University of Medicine and Health Sciences, Dublin, Ireland; Department of Surgery, Beaumont Hospital, Dublin, Ireland; Endocrine Oncology Research Group, Department of Surgery, Royal College of Surgeons in Ireland, University of Medicine and Health Sciences, Dublin, Ireland; Department of Surgery, Beaumont Hospital, Dublin, Ireland; Endocrine Oncology Research Group, Department of Surgery, Royal College of Surgeons in Ireland, University of Medicine and Health Sciences, Dublin, Ireland; Department of Pathology, Beaumont Hospital, Dublin, Ireland; Endocrine Oncology Research Group, Department of Surgery, Royal College of Surgeons in Ireland, University of Medicine and Health Sciences, Dublin, Ireland; Endocrine Oncology Research Group, Department of Surgery, Royal College of Surgeons in Ireland, University of Medicine and Health Sciences, Dublin, Ireland; Endocrine Oncology Research Group, Department of Surgery, Royal College of Surgeons in Ireland, University of Medicine and Health Sciences, Dublin, Ireland; The School of Pharmacy and Biomolecular Sciences, The Royal College of Surgeons University of Medicine and Health Sciences, Dublin, Ireland; Carrick Therapeutics Ltd, Blanchardstown Corporate Park, Dublin, Ireland; Carrick Therapeutics Ltd, Blanchardstown Corporate Park, Dublin, Ireland; Women’s Cancer Research Center, Magee-Womens Research Institute, UPMC Hillman Cancer Center, Pittsburgh, PA 15213, United States; Department of Pharmacology and Chemical Biology, University of Pittsburgh, Pittsburgh, PA 15213, United States; Women’s Cancer Research Center, Magee-Womens Research Institute, UPMC Hillman Cancer Center, Pittsburgh, PA 15213, United States; Department of Pharmacology and Chemical Biology, University of Pittsburgh, Pittsburgh, PA 15213, United States; Department of Laboratory Medicine and Pathology, Mayo Clinic, Rochester, MN 55901, United States; Beaumont RCSI Cancer Centre, Beaumont Hospital, Dublin, Ireland; Endocrine Oncology Research Group, Department of Surgery, Royal College of Surgeons in Ireland, University of Medicine and Health Sciences, Dublin, Ireland; The School of Pharmacy and Biomolecular Sciences, The Royal College of Surgeons University of Medicine and Health Sciences, Dublin, Ireland; Endocrine Oncology Research Group, Department of Surgery, Royal College of Surgeons in Ireland, University of Medicine and Health Sciences, Dublin, Ireland; Department of Surgery, Beaumont Hospital, Dublin, Ireland; Beaumont RCSI Cancer Centre, Beaumont Hospital, Dublin, Ireland

## Abstract

**Background:**

Cyclin-dependent kinase 12 (CDK12) regulates general gene transcription elongation and plays multiple roles in RNA splicing, DNA damage-response, cell cycle, and genomic stability. However, transcriptional partners that guide CDK12-specific gene programs have not been identified. Genomic alterations in CDK12 have been observed in multiple cancers, exhibiting both pro-tumorigenic and tumor-suppressive functions, suggesting a context-dependent mechanism of action.

**Methods:**

CDK12 copy number alterations and gene expression levels were analyzed in matched primary and brain metastatic patient tumors. Clinical significance was assessed by immunohistochemistry in a large cohort of primary breast cancer patient tumors. RNA sequencing, ChIP sequencing, and molecular studies were conducted to explore CDK12’s mechanism of action, and pharmacological studies were performed both in vitro and in vivo using models of advanced (endocrine-resistant and metastatic) estrogen receptor positive (ER+) disease.

**Results:**

CDK12 amplifications and gene overexpression were observed in brain metastatic tumors. In ER+ primary patient tumors, high CDK12 protein expression was significantly associated with poor overall survival, particularly within the ER+/HER2-negative group. In ER+ endocrine resistant models, CDK12 regulated estrogen signaling pathways, with ER/MED1 identified as the master transcriptional complex directing CDK12-specific pro-tumorigenic gene programs. Pharmacological inhibition of CDK12 significantly reduced viability in endocrine resistant and metastatic cell and organoid models in vitro, and decreased metastatic spread in vivo.

**Conclusion:**

This work describes a novel mechanism for CDK12, suggesting a potential vulnerability in ER+ breast cancer. These findings provide a basis for further investigation into the role of CDK12 inhibition as a therapeutic approach, particularly in advanced disease settings.

Breast cancer disease progression to metastasis is a result of genetic adaptations resulting in dysregulated transcriptional programs, causing cancer cells to become highly dependent on gene expression regulators.^1^^,^^2^ This dependency on the core transcriptional machinery results in a particular sensitivity to transcriptional perturbation. Small molecules targeting the transcriptional machinery, including cyclin-dependent kinases (CDKs) and associated proteins, have therefore gained attention in recent years as potential therapeutic targets for cancer treatment.^3^^,^^4^

Transcription-associated CDKs (CDK7, CDK8, CDK9, CDK12, CDK13, and CDK19) are serine/threonine kinases that regulate gene transcription by phosphorylation of the carboxy-terminal domain (CTD) of the DNA-directed RNA polymerase II subunit (RPB1) of RNA polymerase II (RNA Pol II) during the transcription cycle.^5^ The concerted action of transcriptional CDKs and their associated cyclins regulate each of the initiation, pausing, elongation, and termination phases of gene transcription.^6^^,^^7^ CDK12, crucial in the elongation phase, interacts with CDK13 and cyclin K (CCNK) to modulate RNA Pol II processing.^8-11^ In addition to its involvement in transcription, CDK12 has functions in overseeing RNA splicing, managing protein translation, guiding cell cycle progression, orchestrating the DNA damage response (DDR), and preserving genomic stability.^12-15^ However, how CDK12 selectively regulates transcriptional programs to mediate specific gene programs is yet to be elucidated.

CDK12 is frequently altered in human cancer and appears to exert a dual role depending on the specific biological context. It has been characterized as a tumor suppressor in triple-negative breast cancer (TNBC), high-grade serous ovarian cancer, and prostate cancer, where *CDK12* inactivating mutations are associated with increased genomic instability and a more aggressive clinical course.^16-19^ However, in HER2-positive (HER2+) breast cancer, CDK12 acts as a pro-tumorigenic factor, often co-amplified with *ERBB2* (HER2) and cooperating with WNT and IRS1-ErbB-PI3K pathways,^20^^,^^21^ conferring poor prognosis.^22^^,^^23^

Work to date has focused on the contribution of CDK12 to disease progression in HER2+ and to a lesser extent TNBC, but the role of CDK12 in ER+ disease remains largely unknown. In this study, we investigated the role of CDK12 in advanced ER+ breast cancer. We describe a novel mechanism by which CDK12 can use the master regulatory complex ER/MED1, modifying ER chromatin accessibility to direct a pro-tumorigenic transcriptional program. This study validates CDK12 as a *bona fide* therapeutic target in ER+ patient tumors and provides compelling evidence for the efficacy of targeting this transcriptional CDK in endocrine-resistant and metastatic ER+ breast cancer.

## Methods

An expanded description of the material and methods used in this study is provided in the Supplementary Methods.

### Human data

Informed and written consent was obtained before clinical material was collected under the observational clinical trial NCT01840293 (https://clinicaltrials.gov), after ethical approval from Beaumont Hospital Medical Research Ethics Committee (Dublin, Ireland). Clinical data from the University of Pittsburgh and Mayo Clinic were approved as previously described.^24^

### Tissue microarray (TMA)

Tissue microarrays (TMAs) were processed on an automated staining platform. Scores were validated and annotated by a pathologist using the H-score method (CDK12), and the DAB H-score (MED1) with HALO (Indica Labs). Further information is available in the Supplementary Methods. Clinical data are detailed in Tables 1, 2, and 3.

**Table 1.** Clinical and histopathological features of patient primary tumors stratified by CDK12 protein expression. RCSI CDK12 TMA cohort (*n* = 820).

	Total		High CDK12		Low CDK12			
	*n* = 820	%	*n* = 229	%	*n* = 591	%	*P*	Statistical test
Receptor Status								
Estrogen								
ER positive	657	80%	180	79%	477	81%	.4962	Two-sided Fisher exact test
ER negative	163	20%	49	21%	114	19%		
Progesterone								
PR positive	499	61%	131	57%	368	62%	.202	Two-sided Fisher exact test
PR negative	321	39%	98	43%	223	38%		
HER2								
HER2 positive	132	16%	38	17%	94	16%	.8325	Two-sided Fisher exact test
HER2 negative	688	84%	191	83%	497	84%		
Clinical subtype								
ER+/HER2-	575	70%	158	69%	417	71%	.9059	χ^2^
HER2	132	16%	38	17%	94	16%		
TNBC	113	14%	33	14%	80	14%		
Histology								
Invasive ductal carcinoma (IDC)	692	84%	183	80%	509	86%	**.0188**	χ^2^
Invasive lobular carcinoma (ILC)	90	11%	38	17%	52	9%		
IDC + ILC	11	1%	2	1%	9	2%		
Other	22	3%	4	2%	18	3%		
Unknown	5	1%	2	1%	3	1%		
TNM system								
T stage								
T1	221	27%	56	24%	165	28%	.0703	χ^2^
T2	431	53%	117	51%	314	53%		
T3	147	18%	51	22%	96	16%		
T4	16	2%	2	1%	14	2%		
Unknown	5	1%	3	1%	2	0%		
N stage								
N0	358	44%	99	43%	259	44%	.0517	χ^2^
N1	294	36%	68	30%	226	38%		
N2	76	9%	30	13%	46	8%		
N3	73	9%	25	11%	48	8%		
Nx	12	1%	5	2%	7	1%		
Unknown	7	1%	2	1%	5	1%		
M stage								
M0	107	13%	17	7%	90	15%	**.0084**	χ^2^^a^
M1	9	1%	4	2%	5	1%		
Mx	701	85%	205	90%	496	84%		
Unknown	3	0%	3	1%	0	0%		
Pathological staging								
Stage IA	282	34%	68	30%	214	36%	**.0028**	χ^2^
Stage IB	182	22%	51	22%	131	22%		
Stage IIA	88	11%	27	12%	61	10%		
Stage IIB	94	11%	24	10%	70	12%		
Stage IIIA	82	10%	31	14%	51	9%		
Stage IIIB	33	4%	8	3%	25	4%		
Stage IIIC	35	4%	9	4%	26	4%		
Stage IV	9	1%	4	2%	5	1%		
Unknown	15	2%	7	3%	8	1%		
Lymphovascular invasion								
Yes	394	48%	101	44%	293	50%	.0787	χ^2^
No	404	49%	125	55%	279	47%		
Unknown	22	3%	3	1%	19	3%		
Recurrence								
Yes	169	21%	49	21%	120	20%	.7729	Two-sided Fisher exact test
No	651	79%	180	79%	471	80%		
Recurrence time								
Early	102	60%	29	59%	73	61%	.8637	Two-sided Fisher exact test
Late	67	40%	20	41%	47	39%		
Developed metastasis								
Yes	137	17%	40	17%	97	16%	.7544	Two-sided Fisher exact test
No	683	83%	189	83%	494	84%		
Metastatic spread								
Local	22	16%	8	20%	14	14%	.4481	Two-sided Fisher exact test
Distant	115	84%	32	80%	83	86%		

a Test excluded zero value category “Unknown.”

Abbreviations: ER = estrogen receptor; PR = progesterone receptor; HER2 = human epidermal growth receptor 2; IDC = invasive ductal carcinoma; ILC = invasive lobular carcinoma; TNM System, T (Tumor) = size/extent of primary tumor (T1 ≤2 cm; T2 >2-5 cm; T3 >5 cm; T4 = invasion of chest wall/skin); N (Nodes) = lymph node involvement (Nx = nodal status unassessed; N0 = none; N1 = movable axillary; N2 = fixed/matted or internal mammary; N3 = extensive) ; M (Metastasis) = spread to distant sites (Mx = metastasis status radiologically unassessed; M0 = none; M1 = present).

Significant *P* values (<.05) are highlighted in bold.

**Table 2.** Clinical and histopathological features of patient primary tumors stratified by MED1 protein expression.

	Total		High MED1		Low MED1				
	*n* = 807	%	*n* = 651	%	*n* = 156	%	*P*		Statistical test
Receptor status									
Estrogen									
ER positive	644	80%	515	79%	129	83%	.3744		Two-sided Fisher exact test
ER negative	163	20%	136	21%	27	17%			
Progesterone									
PR positive	494	61%	405	62%	89	57%	.235		Two-sided Fisher exact test^a^
PR negative	312	39%	245	38%	67	43%			
N/A	1	0%	1	0%	0	0%			
HER2									
HER2 positive	133	16%	123	19%	10	6%	**<.0001**		Two-sided Fisher exact test
HER2 negative	674	84%	528	81%	146	94%			
Clinical subtype									
ER+/HER2-	561	70%	440	68%	121	78%	**.0008**		χ^2^
HER2	133	16%	123	19%	10	6%			
TNBC	113	14%	88	14%	25	16%			
Histology									
Invasive ductal carcinoma (IDC)	674	84%	540	83%	134	86%	.8425		χ^2^
Invasive lobular carcinoma (ILC)	84	10%	69	11%	15	10%			
IDC + ILC	12	1%	11	2%	1	1%			
Other	31	4%	26	4%	5	3%			
Unknown	6	1%	5	1%	1	1%			
TNM system									
T stage									
T1	209	26%	163	25%	46	29%	.1221		χ^2^
T2	426	53%	337	52%	89	57%			
T3	140	17%	123	19%	17	11%			
T4	18	2%	16	2%	2	1%			
Unknown	14	2%	12	2%	2	1%			
N stage									
N0	336	42%	274	42%	62	40%	.1218		χ^2^^b^
N1	257	32%	197	30%	60	38%			
N2	64	8%	55	8%	9	6%			
N3	71	9%	63	10%	8	5%			
Nx	2	0%	2	0%	0	0%			
Unknown	77	10%	60	9%	17	11%			
M stage									
M0	104	13%	78	12%	26	17%	**.0358**		χ^2^^c^
M1	9	1%	9	1%	0	0%			
Mx	691	86%	563	86%	128	82%			
Unknown	3	0%	1	0%	2	1%			
Pathological staging									
Stage IA	279	35%	222	34%	57	37%	**.0258**		χ^2^
Stage IB	176	22%	144	22%	32	21%			
Stage IIA	86	11%	70	11%	16	10%			
Stage IIB	92	11%	66	10%	26	17%			
Stage IIIA	80	10%	70	11%	10	6%			
Stage IIIB	35	4%	33	5%	2	1%			
Stage IIIC	35	4%	24	4%	11	7%			
Stage IV	9	1%	9	1%	0	0%			
Unknown	15	2%	13	2%	2	1%			
Histological grade									
Grade I	108	13%	90	14%	18	12%	.258		χ^2^^d^
Grade II	389	48%	319	49%	70	45%			
Grade III	304	38%	236	36%	68	44%			
Unknown	6	1%	6	1%	0	0%			
Lymphovascular invasion									
Yes	388	48%	302	46%	86	55%	.1012		χ^2^
No	398	49%	330	51%	68	44%			
Unknown	21	3%	19	3%	2	1%			
Recurrence									
Yes	169	21%	138	21%	31	20%	.8266		Two-sided Fisher exact test
No	638	79%	513	79%	125	80%			
Recurrence time									
Early	102	60%	83	60%	19	61%	>.9999		Two-sided Fisher exact test
Late	67	40%	55	40%	12	39%			
Developed metastasis									
Yes	134	17%	109	17%	25	16%	.9049		Two-sided Fisher exact test
No	673	83%	542	83%	131	84%			
Metastatic spread									
Local	22	16%	15	14%	7	28%	.1296		Two-sided Fisher exact test
Distant	112	84%	94	86%	18	72%			

RCSI MED1 TMA cohort (*n* = 807).

a Test excluded zero value category “NA.”

b Test excluded zero value category “Nx.”

c Test excluded zero value category “M1.”

d Test excluded zero values’ category “Unknown.”

Abbreviations: ER = estrogen receptor; PR = progesterone receptor; HER2 = human epidermal growth receptor 2; IDC = invasive ductal carcinoma; ILC = invasive lobular carcinoma; TNM System, T (Tumor) = size/extent of primary tumor (T1 ≤ 2 cm; T2 > 2-5 cm; T3 > 5 cm; T4 = invasion of chest wall/skin); N (Nodes) = lymph node involvement (Nx = nodal status unassessed; N0 = none; N1 = movable axillary; N2 = fixed/matted or internal mammary; N3 = extensive); M (Metastasis) = spread to distant sites (Mx = metastasis status radiologically unassessed; M0 = none; M1 = present).

Significant *P* values (<.05) are highlighted in bold.

**Table 3.** Clinical and histopathological features of patient primary tumors stratified by MED1 and CDK12 protein expression. RCSI MED1/CDK12 TMA cohort (*n* = 788).

	Total		^1^ High CDK12/High MED1		^2^ High CDK12/Low MED1		^3^ Low CDK12/High MED1		^4^ Low CDK12/Low MED1		*P* (all groups)	*P* (1 vs 4)	Statistical test
	*n* = 788	%	*n* = 182	%	*n* = 37	%	*n* = 452	%	*n* = 117	%			
Receptor status													
Estrogen													
ER positive	628	80%	138	76%	33	89%	362	80%	95	81%	.2849	.3182	Two-sided Fisher exact test
ER negative	160	20%	44	24%	4	11%	90	20%	22	19%			
Progesterone													
PR positive	479	61%	102	56%	20	54%	289	64%	68	58%	.1917	.811	Two-sided Fisher exact test
PR negative	309	39%	80	44%	17	46%	163	36%	49	42%			
HER2													
HER2 positive	128	16%	34	19%	2	5%	85	19%	7	6%	**.0006**	**.0017**	Two-sided Fisher exact test
HER2 negative	660	84%	148	81%	35	95%	367	81%	110	94%			
Clinical subtype													
ER+/HER2-	549	70%	119	65%	31	84%	309	68%	90	77%	**.0084**	**.0075**	χ^2^
HER2	128	16%	34	19%	2	5%	85	19%	7	6%			
TNBC	111	14%	29	16%	4	11%	58	13%	20	17%			
Histology													
Invasive ductal carcinoma (IDC)	660	84%	145	80%	30	81%	383	85%	102	87%	**.0423**	.0696	Two-sided Fisher exact test^a^
Invasive lobular carcinoma (ILC)	82	10%	28	15%	6	16%	39	9%	9	8%			
IDC + ILC	11	1%	2	1%	0	0%	8	2%	1	1%			
Other	30	4%	5	3%	1	3%	20	4%	4	3%			
Unknown	5	1%	2	1%	0	0%	2	0%	1	1%			
TNM system													
T stage													
T1	203	26%	42	23%	10	27%	116	26%	35	30%	.2089^b^	.0541	χ^2^
T2	417	53%	90	49%	22	59%	239	53%	66	56%			
T3	140	18%	44	24%	4	11%	79	17%	13	11%			
T4	17	2%	2	1%	0	0%	13	3%	2	2%			
Unknown	11	1%	4	2%	1	3%	5	1%	1	1%			
N stage													
N0	329	42%	69	38%	18	49%	198	44%	44	38%	.0951	**.0245**	χ^2c^
N1	251	30%	53	29%	12	32%	139	31%	47	40%			
N2	63	8%	23	13%	3	8%	31	7%	6	5%			
N3	69	8%	19	10%	3	8%	42	9%	5	4%			
Nx	2	0%	2	1%	0	0%	0	0%	0	0%			
Unknown	74	12%	16	9%	1	3%	42	9%	15	13%			
M stage													
M0	103	13%	14	8%	2	5%	63	14%	24	21%	**.0086**	**.0023**	Two-sided Fisher exact test^d^
M1	9	1%	4	2%	0	0%	5	1%	0	0%			
Mx	673	85%	163	90%	33	89%	384	85%	93	79%			
Unknown	3	0%	1	1%	2	5%	0	0%	0	0%			
Pathological staging													
Stage IA	270	34%	51	28%	13	35%	163	36%	43	37%	.1417	**.0154**	χ^2^
Stage IB	175	22%	42	23%	6	16%	101	22%	26	22%			
Stage IIA	83	11%	20	11%	5	14%	47	10%	11	9%			
Stage IIB	92	12%	17	9%	7	19%	49	11%	19	16%			
Stage IIIA	79	10%	28	15%	3	8%	41	9%	7	6%			
Stage IIIB	32	4%	8	4%	0	0%	23	5%	1	1%			
Stage IIIC	35	4%	7	4%	2	5%	17	4%	9	8%			
Stage IV	9	1%	4	2%	0	0%	5	1%	0	0%			
Unknown	13	2%	5	3%	1	3%	6	1%	1	1%			
Histological grade													
Grade I	105	13%	22	12%	5	14%	65	14%	13	11%	.832	.4898	χ^2^^e^
Grade II	379	48%	92	51%	17	46%	217	48%	53	45%			
Grade III	298	38%	66	36%	15	41%	166	37%	51	44%			
Unknown	6	1%	2	1%	0	0%	4	1%	0	0%			
Lymphovascular invasion													
Yes	381	48%	74	41%	23	62%	222	49%	62	53%	**.034**	**.0416**	Two-sided Fisher exact test^e^
No	387	49%	106	58%	14	38%	214	47%	53	45%			
Unknown	20	3%	2	1%	0	0%	16	4%	2	2%			
Recurrence													
Yes	162	21%	40	22%	7	19%	92	20%	23	20%	.9599	.6653	Two-sided Fisher exact test
No	626	79%	142	78%	30	81%	360	80%	94	80%			
Recurrence time													
Early	98	60%	23	58%	5	71%	56	61%	14	61%	.9469	>.9999	Two-sided Fisher exact test
Late	64	40%	17	43%	2	29%	36	39%	9	39%			
Developed metastasis													
Yes	131	17%	33	18%	6	16%	73	16%	19	16%	.9345	.7553	Two-sided Fisher exact test
No	657	83%	149	82%	31	84%	379	84%	98	84%			
Metastatic spread													
Local	21	16%	6	18%	2	33%	8	11%	5	26%	.1527	.5031	Two-sided Fisher exact test
Distant	110	84%	27	82%	4	67%	65	89%	14	74%			

a Test excluded zero values’ categories “IDC + ILC,” “Other,” and “Unknown.”

b Test excluded zero value category “T4.”

c Test excluded zero value category “Nx.”

d Test excluded zero values’ categories “M1” and “Unknown.”

e Test excluded zero value category “Unknown.”

Abbreviations: ER = estrogen receptor; PR = progesterone receptor; HER2 = human epidermal growth receptor 2; IDC = invasive ductal carcinoma; ILC = invasive lobular carcinoma; TNM System, T (Tumor) = size/extent of primary tumor (T1 ≤ 2 cm; T2 > 2-5 cm; T3 > 5 cm; T4 = invasion of chest wall/skin); N (Nodes) = lymph node involvement (Nx = nodal status unassessed; N0 = none; N1 = movable axillary; N2 = fixed/matted or internal mammary; N3 = extensive); M (Metastasis) = spread to distant sites (Mx = metastasis status radiologically unassessed; M0 = none; M1 = present).

Significant *P* values (<.05) are highlighted in bold.

### Survival analysis

Kaplan-Meier estimates of breast cancer-specific overall survival and progression-free survival were determined for protein expression in our TMA cohorts for CDK12 (*n* = 820), MED1 (*n* = 807), and merged cohorts CDK12/MED1 (*n* = 788).

### RNA sequencing (RNA-seq)

Total RNA was isolated from LY2 cells transfected with siRNA targeting CDK12 (siCDK12) and nontargeting control (siCtrl) for 48 hours. Library preparation and RNA-seq (200 ng RNA) were performed by BGI Genomics (Hong Kong).

### Chromatin immunoprecipitation (ChIP) sequencing

Chromatin immunoprecipitation (ChIP) was performed on LY2 cells transfected with siCDK12 or siCtrl (48 hours), followed by treatment with 1 µmol/L β-estradiol (E4389, Merck). Library preparation and DNA sequencing (10 ng DNA) were performed by BGI Genomics (Hong Kong).

### In vitro studies

Cell/organoid viability and cell colony formation assays were performed using the CDK12 inhibitor CT7116 (300 nmol/L) alone, or in combination with fulvestrant (100 nmol/L) or ribociclib (1 µmol/L), and assessed with MTS assays (cells) and CellTiter-Glo 3D (organoids). Cell colonies were counted using ImageJ software.

### Organoids

Organoid cultures derived from brain (T347, T638) and lung metastatic (HCI-05, HCI-11) patient samples^24^ were generated as previously described.^25^

### In vivo study

Treatment with CDK12 inhibitor CT7311 (0.5 mg/kg) was initiated 24 hours post-intracardiac injection of LY2 luciferase-tagged cells in female NOD SCID mice. The drug was administered via intraperitoneal injection on a schedule of 5 on/2 off. Bioluminescent imaging (BLI) was undertaken weekly (In Vivo Imaging System, Newton 7.0), including a final image before termination.

### Statistical analysis

Statistical significance was set at *P *< .05. All analyses were performed using GraphPad Prism (10.1.0) and R software (4.2.1): log-rank Mantel-Cox test (survival analysis), cutpointr (v1.1.2) (ROC curve analysis), coxph (v3.4-0) (hazard ratios), dba.analyze and ChIPpeakAnno^26^ (v3.32.0) (ChIP-seq), 1-tailed unpaired *t* test (ER and MED1 ChIP-qPCR), 2-tailed unpaired *t* test with Welch’s correction when appropriate (cell viability, colony formation, organoid assays), ordinary 1-way ANOVA (combo drug treatments), 2-tailed Mann-Whitney test (CDK12 gene expression in patient samples, whole-body BLI, and ex vivo organs).

## Results

### CDK12 is frequently altered in advanced breast cancer

**Figure 1.**
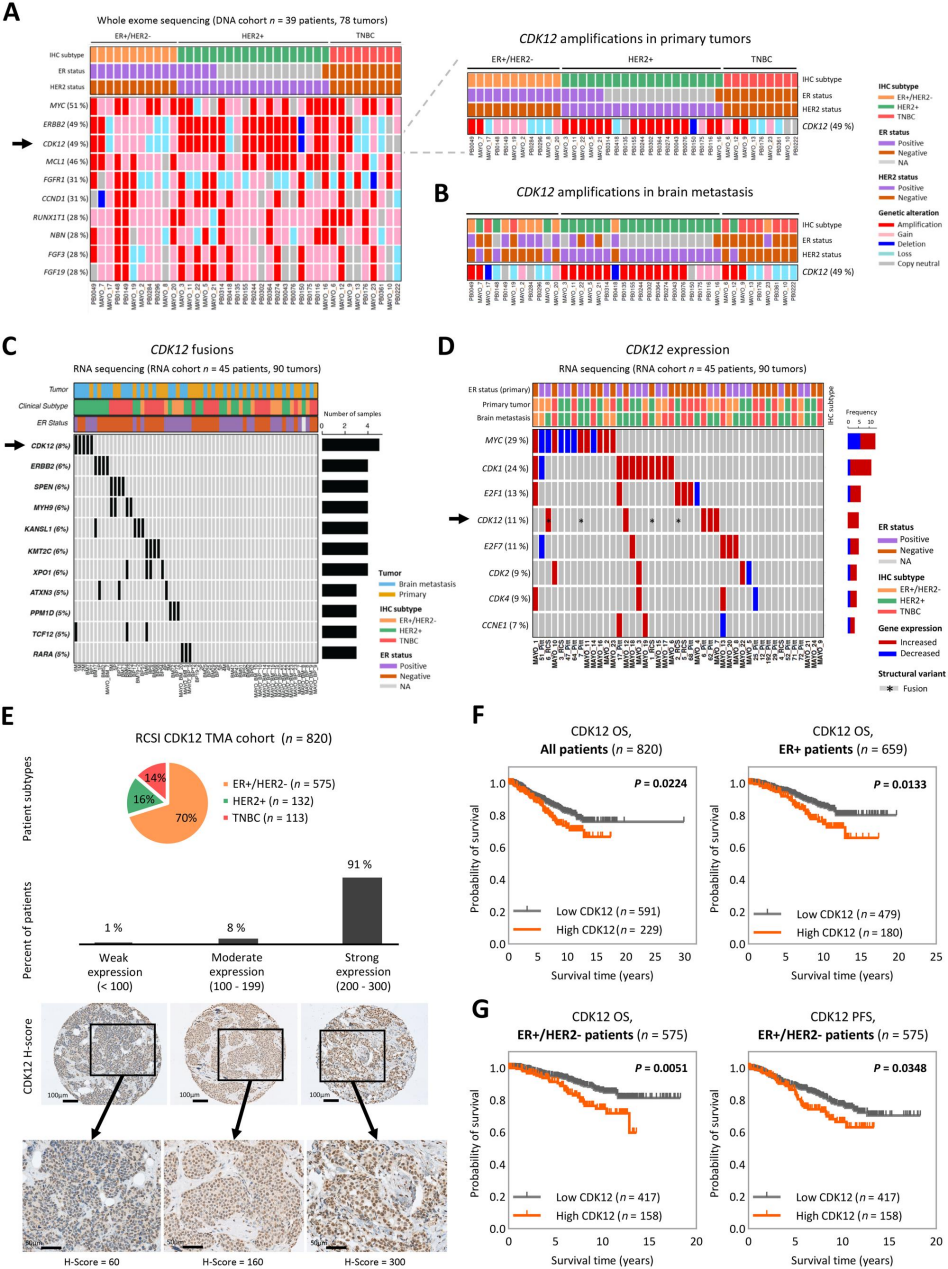
CDK12 is frequently altered in primary and brain metastatic breast cancer. **A**) Somatic copy number alteration analysis in matched primary and brain metastatic tumors from the DNA cohort (whole exome sequencing, DNA cohort: *n* = 39 patients, 78 tumors). Oncoprint shows the top 10 most frequently amplified genes in primary breast tumors. The proportion of patients with gene amplifications is indicated next to each gene. *CDK12* (highlighted with a black arrow) was amplified in 49% of primary tumors (*n* = 19). **B**) *CDK12* amplifications were also observed in 49% of matched brain metastatic tumors (*n* = 19). **C**) RNA sequencing of matched primary and brain metastatic tumors (RNA cohort, *n* = 45 patients, 90 tumors) revealed recurrent high-confidence CDK12 gene fusions (black arrow) in 4 brain metastases and 1 primary tumor (*n* = 4 patients, 5 samples; 8%). **D**) Differential gene expression analysis from RNA-seq data (brain metastasis vs primary tumors) identified aberrantly elevated CDK12 mRNA levels (black arrow) in 11% (*n* = 5) of brain metastases. Samples with *CDK12* fusions are marked with an asterisk (*). **E**) CDK12 protein expression was assessed by immunohistochemistry in primary tumors using a tissue microarray (TMA) from the RCSI cohort (*n* = 820). The cohort composition by molecular subtype is shown in the pie chart: ER+/HER2-, HER2+, and triple-negative breast cancer (TNBC). CDK12 expression levels, quantified by H-score, were classified as weak (<100, *n* = 9, 1%), moderate (100-199, *n* = 63, 8%), and strong (200-300, *n* = 748, 91%). Representative IHC images of CDK12 staining are shown. Scale bars: 100 μm and 50 μm. **F**) Kaplan-Meier survival analysis based on CDK12 protein expression (H-score ≥295 = high CDK12; <295 = low CDK12). High CDK12 expression was significantly associated with poor overall survival (OS) in all breast cancer patients (*n* = 820, *P* = .0224) and in ER+ patients (*n* = 659, *P* = .0133). **G**) In the ER+/HER2- population (*n* = 575), high CDK12 expression correlated significantly with both poor OS (*P* = .0051) and progression-free survival (PFS, *P* = .0348). Statistical significance was determined using the log-rank (Mantel-Cox) test.

We previously characterized a large cohort of paired primary and brain metastatic breast cancer patient samples (*n* = 39 patients, *n* = 78 tumors).^24^ In this study, somatic copy number alteration (SCNA) analysis identified *CDK12* amplifications in almost half (49%) of both the primary (*n* = 19) and brain metastatic (*n* = 19) tumors, with co-amplification of *ERBB2* (Figure 1A, B). Among the 19 metastatic tumors with *CDK12* amplifications, 16 retained the alteration from the matched primary tumor, whereas 3 represented newly acquired amplifications (Figure 1B). Consistently, analysis of online datasets revealed *CDK12* amplifications in both primary (13%; METABRIC^27-29^) and metastatic (31%; the Metastatic Breast Cancer project, cBioportal^30-32^) breast cancer (Figure S1A, B).

Fusion analysis of exon capture RNA-sequencing (RNA-seq) in matched primary and brain metastatic samples (*n *= 45 patients, *n* = 90 tumors) identified 2994 high-confidence transcript fusions in 31 matched samples (Table S1). Cross-referencing with known breast cancer driver genes^33^^,^^34^ defined 11 recurrent fused genes (Figure S1C), with *CDK12* as the top-ranked fusion, present in 8% of patient samples (1 primary tumor, 4 brain metastases; Figure 1C, Table S1). We next analyzed CDK12 gene expression between primary and brain metastatic samples. Differential analysis of CDKs and cyclin-related genes frequently altered in metastatic breast cancer (Figure S1D) revealed an increased expression of *CDK12* in brain metastases compared with matched primary tumors (*n* = 5, 11%; Figure 1D, Table S2). Integration of *CDK12* expression with SCNA and fusions across all tumor samples from this study demonstrated elevated *CDK12* expression levels in primary and brain metastatic tumors harboring *CDK12* amplifications or *CDK12* fusions (Table S3, Figure S1E, F).

Notably, high *CDK12* expression was found to correlate with poor overall survival (OS) in breast cancer patients (*P *= .034), and specifically in ER+ tumors (HER2-positive/HER2-negative) (*P *= .0091), but not in ER-negative (ER-) patients (*P *= .49) (KM plotter;^35^  Figure S2A, B).

We then evaluated CDK12 protein expression in a primary breast cancer tissue microarray (TMA) comprising 820 patients (Table 1). CDK12 was strongly expressed in 91% of tumors (*n* = 748), most of these from the ER+/HER2- subtype (*n *= 451, 64.6%) (Figure 1E; Figure S2C, D). Survival analysis revealed a significant association between high CDK12 protein expression and poor OS in all breast cancer patient subtypes (*n* = 820, *P *= .0224), as well as within the ER+ population (*n* = 659, *P *= .0133) (Figure 1F). In addition, a significant association between high CDK12 expression and progression-free survival (PFS) was observed exclusively in ER+/HER2- patients (*n* = 575, OS: *P *= .0051, PFS: *P *= .0348) (Figure 1G). No significant association was found in the ER- population (*n* = 161, *P *= .7294) (Figure S2B), nor in HER2+ (ER+ and ER-) or TNBC subtypes for OS or PFS (Figure S2E-H). Multivariate analyses confirmed CDK12 as an independent adverse prognostic factor, irrespective of other clinical variables (age at diagnosis, tumor stage and grade), in the overall patient cohort (Figure S3A), with increasingly significant *P*-values observed in ER+ and ER+/HER2- subtypes (Figure S3B, C).

### CDK12 regulates estrogen pathways and modulates the ER cistrome in advanced breast cancer

**Figure 2.**
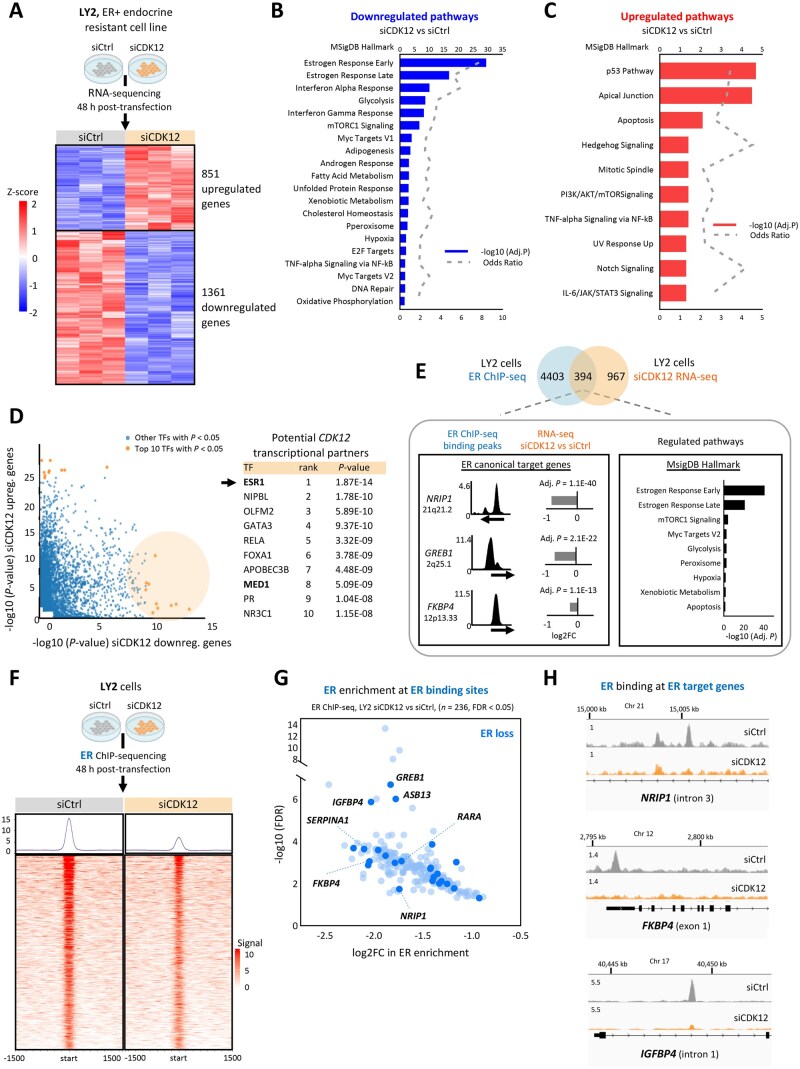
CDK12 regulates estrogen signaling and modulates the ER cistrome in ER+ advanced breast cancer. **A**) RNA-seq analysis after *CDK12* knockdown (siRNA, 48 hours) in LY2 endocrine-resistant ER+ breast cancer cells. A mean-centered heatmap with hierarchical clustering displayed 2212 significantly deregulated genes (adjusted *P* < .05): 851 upregulated genes (log2FC > 0; red) and 1361 downregulated genes (log2FC < 0; blue) in siCDK12 vs siCtrl. **B**) Pathway enrichment analysis of downregulated genes (*n* = 1361) using MSigDB hallmark gene sets (top 20 terms, adj. *P* < .05). Estrogen response—early (adj. *P* = 3.45E-30) and late (adj. *P* = 1.51E-17)—was the most significant downregulated pathway. Additional key pathways affected include cell proliferation, cell cycle progression, and DNA repair. **C**) Pathway analysis of upregulated genes (*n* = 851) in siCDK12 identified p53 signaling (adj. *P* = 2.01E-05) and apoptosis (adj. *P* = .008) as the most enriched pathways. **B-C**) Adjusted *P*-values were computed using the Benjamini-Hochberg method in Enrichr. **D**) In silico transcription factor binding prediction (Epigenetic Landscape In Silico deletion Analysis, LISA) using the top 500 significantly downregulated genes identified ESR1 (black arrow, adj. *P* = 1.87E-14) and MED1 (bold, adj. *P* = 5.09E-14) as candidate transcriptional partners of CDK12. *P-*values were computed in LISA Cistrome online tool. **E**) Integration of LY2 ER ChIP-seq data (ER target genes, *n* = 4403) and CDK12 knockdown RNA-seq data (downregulated genes, *n* = 1361) identified 394 overlapping genes, representing ∼29% of CDK12-regulated transcripts as direct ER targets. **Bottom left**: ER ChIP-seq binding peaks at canonical ER targets *NRIP1*, *GREB1*, and *FKBP4*, with corresponding downregulation by RNA-seq: *NRIP1* (log2FC = -0.81, adj. *P* = 1.1E-40), *GREB1* (log2FC = -0.70, adj. *P* = 2.1E-22), *FKBP4* (log2FC = -0.25, adj. *P* = 1.1E-13). **Bottom right**: Pathway analysis of the overlapping genes (*n* = 394) revealed significant enrichment of estrogen response pathways (early: adj. *P* = 9.53E-42; late adj. *P* = 8.02E-22). Adjusted *P*-values were computed using the Benjamini-Hochberg method in Enrichr. **F**) ER ChIP-seq analysis in LY2 cells after *CDK12* knockdown (48 hours). Heatmap of consensus ER binding sites (*n* = 256, FDR < 0.05) showed altered ER chromatin occupancy between siCtrl and siCDK12 conditions. **G**) Dot plot of differential ER binding (ER ChIPseq, siCDK12 vs siCtrl) showing significant loss of ER occupancy at 236 binding sites (light blue). Canonical ER target genes involved in pro-proliferative estrogenic signaling are highlighted in dark blue. Adjusted *P*-values were computed using the Benjamini-Hochberg method. **H**) Genome browser views of ER ChIP-seq binding peaks at ER target genes *NRIP1*, *FKBP4*, and *IGFBP4*. ER binding is shown in gray (siCtrl) and orange (siCDK12), aligned to RefSeq gene annotations.

Given the association found between CDK12 expression and poor survival in ER+ breast cancer, we investigated the role of CDK12 in global gene expression using an ER+ endocrine resistant model (LY2 cells). RNA-seq analysis of *CDK12* knockdown (siCDK12) in LY2 cells revealed 1361 downregulated and 851 upregulated genes compared with the control (siCtrl) (Figure 2A, Figure S4A, B, C, Table S4). Pathway analysis of downregulated genes (*n* = 1361) revealed *estrogen response* as the top downregulated hallmark term (Figure 2B). Key estrogen response genes, including *ESR1* and canonical ER targets (*ABCA3, FKBP4, GREB1, IGFBP4, NRIP1*), were significantly downregulated in *CDK12* knockdown (Figure S4D), indicating a functional role for CDK12 in estrogen signaling regulation. Other CDK12-regulated processes included cell proliferation, cell cycle progression, and DNA repair (Figure 2B). In contrast, *CDK12* loss upregulated pathways related to p53 activation and apoptosis (Figure 2C), the latter confirmed by PARP cleavage (Figure S4E).

To investigate the CDK12 transcriptional interactome, we performed epigenetic landscape in silico deletion analysis (LISA^36^) identifying ER (*ESR1*) as the top transcription factor co-regulating the CDK12-driven transcriptome (Figure 2D, Table S5). Rapid immunoprecipitation mass spectrometry of endogenous proteins (RIME) analysis in ER+ breast cancer cell models (EstroGene^37^) confirmed significant CDK12-ER interactions. The integration of siCDK12 RNA-seq with ER chromatin immunoprecipitation (ChIP) sequencing (ChIP-seq) data^38^ revealed that nearly 30% (*n* = 394) of the CDK12-regulated genes were direct ER targets (Figure 2E, Table S5). *Estrogen response* emerged as the most significantly enriched pathway, alongside previously identified hallmark terms under CDK12 regulation (mTORC1 signaling, MYC targets, glycolysis) (Figure 2B, E).

The impact of CDK12 on genome-wide ER binding was further investigated using ER ChIP-seq after *CDK12* knockdown in LY2 cells, revealing reduced global ER chromatin recruitment (Figure 2F). Notably, ER enrichment significantly declined at ER-consensus binding sites (siCDK12 vs siCtrl, FDR < 0.05, *n* = 236 sites, *n* = 218 genes) (Figure 2G, Figure S4F, G), including genes previously found downregulated with *CDK12* knockdown (*NRIP1, FKBP4, IGFBP4*) (Figure 2G, H, Figure S4D). Consistent with our earlier observation, pathway analysis of differentially bound ER genes (*n* = 218) confirmed *estrogen response* as the most significant enriched hallmark term (Figure S4H).

Together, these findings highlight a novel role for CDK12 in the regulation of ER gene transcription in advanced ER+ breast cancer.

### MED1 and CDK12 protein expression is associated with poor survival in ER+ breast cancer patients

**Figure 3.**
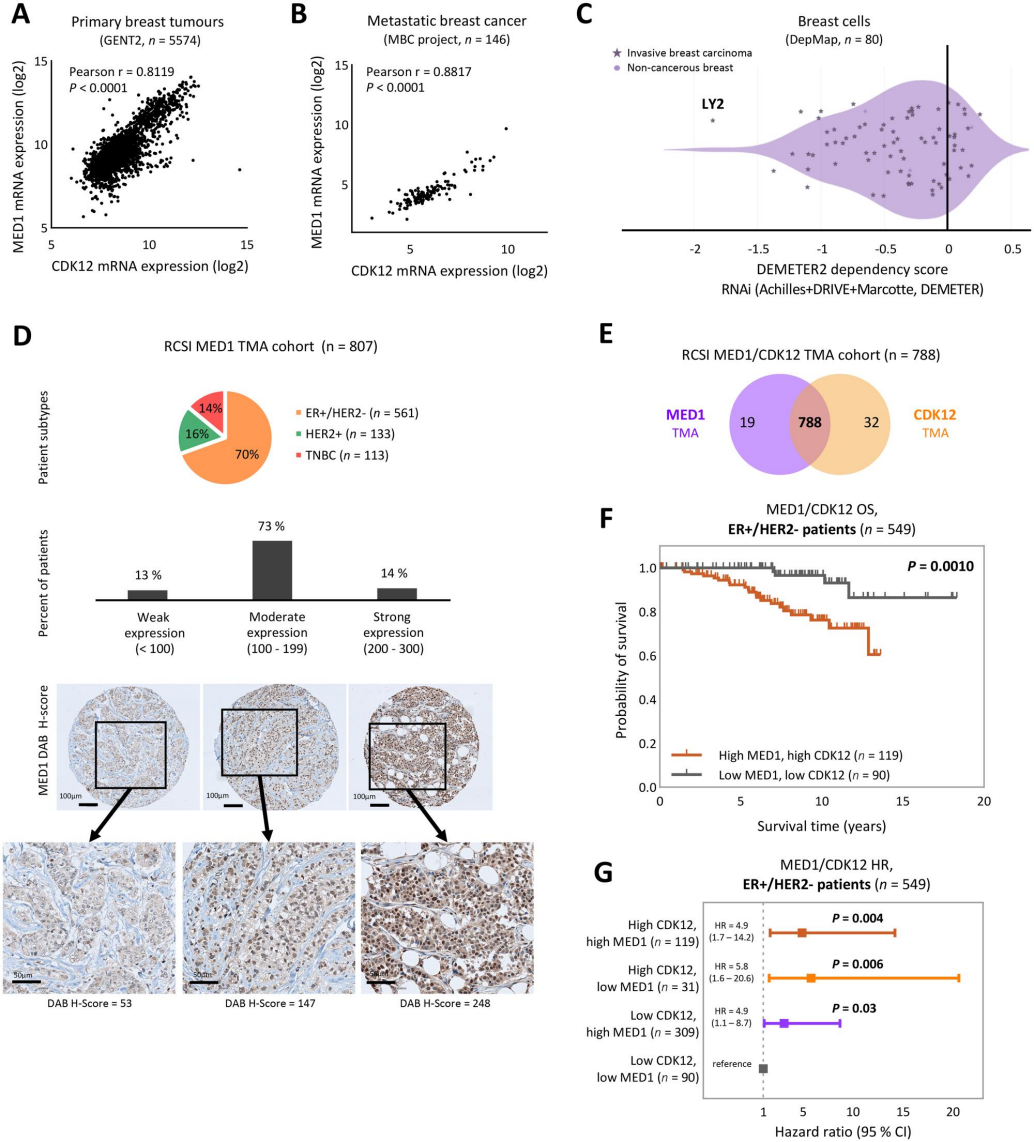
MED1 and CDK12 protein expression correlate with poor prognosis in ER+/HER2- breast cancer patients. **A-B**) Strong positive correlation between MED1 and CDK12 mRNA expression in **(A)** primary breast tumors (GENT2, *n* = 5574; Pearson *r* = 0.8119, *P* < .0001) and **(B)** metastatic breast cancer (MBC project, *n* = 146; Pearson *r* = 0.8817, *P* < .0001). *P*-values calculated by Pearson correlation coefficient (r). **C**) *MED1* knockout gene effect in breast cancer cell lines. MED1 gene dependency across breast cancer cell lines (DepMap, *n* = 80). Invasive breast carcinoma cells (*n* = 77, violet stars) and noncancerous breast cells (*n* = 3, violet dots) are shown. LY2 cells exhibit the lowest DEMETER2 dependency score (–1.85), indicating that *MED1* is essential in this model. **D**) MED1 protein expression was assessed by immunohistochemistry in primary tumors using a tissue microarray (TMA) from the RCSI cohort (*n* = 807). The cohort composition by molecular subtype is shown in the pie chart: ER+/HER2-, HER2+, and TNBC. Protein levels were quantified using the HALO system and classified as weak (DAB H-score <100, *n* = 107, 13%), moderate (H-score = 100-199, *n* = 586, 73%), and strong (H-score = 200-300, *n* = 114, 14%). Representative IHC images of MED1 staining are shown. Scale bars: 100 μm and 50 μm. **E**) Integration of MED1 (*n* = 807) and CDK12 (*n* = 820) TMA cohorts yielded 788 primary tumors for combined analysis. **F**) Kaplan-Meier survival analysis in ER+/HER2- patients (*n* = 549) based on MED1 and CDK12 protein levels. Patients with high MED1 (DAB H-score ≥108.51) and high CDK12 (H-score ≥295) had significantly worse overall survival (OS) than those with low expression levels of both markers (*P* = .0010). Log-rank (Mantel-Cox) test was used for significance testing. **G**) Cox proportional hazards model assessing hazard ratios (HR) for death in ER+/HER2- patients (*n* = 549) by MED1/CDK12 expression status. Relative to the low MED1/low CDK12 reference group (*n* = 90): high CDK12/high MED1 (*n* = 119): HR = 4.9 (1.7-14.2), *P* = .004; high CDK12/low MED1 (n = 31): HR = 5.8 (1.6-20.6), *P* = .006; low CDK12/high MED1 (*n* = 309): HR = 4.9 (1.1-8.7), *P* = .03. Hazard ratios and statistical *P*-values were calculated with Cox proportional hazards model.

Alongside ER, the established ER co-activator MED1^39^ was identified as a potential CDK12 transcriptional partner (Figure 2D). Analysis of public breast cancer datasets revealed a strong positive correlation between *MED1* and *CDK12* expression in both primary (GENT2,^40^  *r *= 0.8119, *P *< .0001) (Figure 3A) and metastatic breast cancer tumors (MBC project, *r *= 0.8817, *P *< .0001) (Figure 3B). Additionally, genome-wide RNAi and CRISPR knockout screens (Cancer Dependency Map, DepMap^41^) showed *MED1* knockout inhibits breast cancer cell lines (Figure 3C) and is essential in LY2 cells, ranking highest among preferentially essential genes (Figure S5A).

At a clinical level, strong MED1 protein expression was detected in 14% of primary tumors from our patient cohort (TMA, *n *= 807 patients) (Table 2, Figure 3D, Figure S5B, C), with no significant association with OS across patient populations (Figure S5D-H). The integration of MED1 and CDK12 TMA analysis (*n* = 788 patients) (Table 3, Figure 3E) revealed that tumors with high MED1 and high CDK12 had poorer survival in ER+/HER2- patients (*n* = 549, *P *= .0010) (Figure 3F, G). No significant associations were observed in ER+/HER2+ or ER- patients (Figure S5I, J), consistent with previous CDK12 and MED1 survival analysis (Figure S2E, F; Figure S5G, H).

### CDK12 regulates ER and MED1 chromatin recruitment

**Figure 4.**
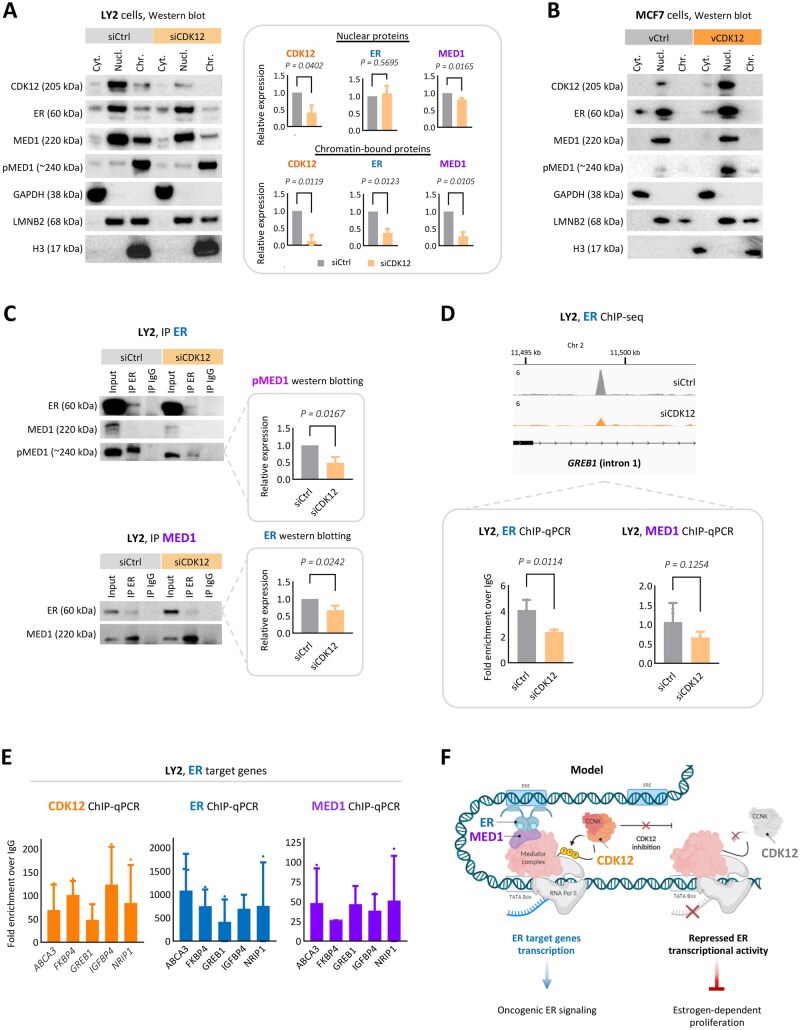
CDK12 modulates ER and MED1 chromatin recruitment. **A**) CDK12 knockdown in LY2 ER+ endocrine resistant cells. **Left**: Immunoblot analysis of LY2 cells after *CDK12* knockdown (siRNA, 48 hours), showing protein levels of CDK12, ER, MED1, and phosphorylated MED1 (pMED1) in cytoplasmic (Cyt.), nuclear (Nucl.), and chromatin-bound (Chr.) fractions. GAPDH, LMNB2, and H3 were used as loading controls for cytoplasmic, nuclear, and chromatin-bound fractions, respectively. Molecular weights (kDa) are indicated. Blot is representative of 3 independent experiments. **Right**: Densitometric quantification of protein levels (siCDK12 relative to siCtrl) in nuclear and chromatin-bound fractions. LMNB2 and H3 were used for normalization. Error bars represent mean ± SD from 3 independent experiments. Statistical *P*-values were calculated by 2-tailed paired *t* test. Nuclear: CDK12, *P* = .0402; MED1, *P* = .0165; ER, *P* = .5695. Chromatin-bound: CDK12, *P* = .0119; MED1, *P* = .0105; ER, *P* = .0123. **B**) CDK12 overexpression in MCF7 ER+ endocrine sensitive cells. Immunoblot analysis of MCF7 cells after *CDK12* overexpression (48 hours). Protein levels of CDK12, ER, MED1, and pMED1 are shown in Cyt., Nucl., and Chr. fractions. GAPDH, LMNB2, and H3 were used as loading controls for cytoplasmic, nuclear, and chromatin-bound fractions, respectively. Molecular weights (kDa) are indicated. Blot is representative of 3 independent experiments. **C**) ER/MED1 endogenous interaction after *CDK12* knockdown in LY2 cells (nuclear proteins). Reciprocal co-immunoprecipitation (co-IP) of nuclear ER (**top**) and MED1 (**bottom**) proteins after *CDK12* knockdown (siRNA, 48 hours) in LY2 cells, with densitometric quantification of protein levels (bar graph, siCDK12 relative to siCtrl). Immunoblots show ER, MED1, and pMED1. Blots represent 3 independent experiments. Error bars represent mean ± SD from 3 independent experiments. Statistical *P*-values were calculated by 1-tailed paired *t* test: IP ER/pMED1 western blotting, *P* = .0167; IP MED1/ER western blotting, *P* = .0242. **D**) ER and MED1 recruitment to ER target gene *GREB1* after *CDK12* knockdown in LY2 cells. **Top**: ER ChIP-seq tracks at *GREB1* (intron 1) in LY2 cells (siRNA, 48 hours: siCtrl, gray; siCDK12, orange). **Bottom**: ER and MED1 chromatin immunoprecipitation followed by qPCR (ChIP-qPCR) at *GREB1* in LY2 cells. Bar graphs show fold enrichment of ER and MED1 binding at *GREB1* after *CDK12* knockdown. Error bars represent mean ± SD from 3 independent experiments. Statistical *P*-values were calculated by 1-tailed unpaired *t* test: ER ChIP-qPCR, *P* = .0114; MED1 ChIP-qPCR, *P* = .1254. **E**) CDK12, ER, and MED1 recruitment to ER target genes in LY2 cells. ChIP-qPCR of CDK12, ER, and MED1 showing protein recruitment to classic ER target genes *ABCA3*, *FKBP4*, *GREB1*, *IGFBP4*, and *NRIP1*. **F**) Proposed model: CDK12-dependent ER pro-tumorigenic signaling. CDK12 promotes pro-tumorigenic ER signaling in advanced ER+ breast cancer by stabilizing the ER-MED1 complex and facilitating their recruitment to chromatin. CDK12 loss reduces ER/MED1 chromatin occupancy and interaction, thereby impairing ER-driven transcription.

*CDK12* knockdown significantly reduced ER and MED1 proteins in the nuclear and, to a greater extent, chromatin-bound fractions of LY2 cells, with no change in the phosphorylated form of MED1 (pMED1) (Figure 4A, Figure S6A). However, in endocrine-sensitive T47D cells, CDK12 depletion did not affect ESR1 or MED1 expression, nor did it alter ER target gene expression (Figure S6B-C). By contrast, overexpression of CDK12 in endocrine-sensitive MCF7 cells leads to enhanced nuclear expression of ER and p-MED1, with modest increases in ER target genes observed (Figure 4B, Figure S6D).

Co-immunoprecipitation revealed a dependency on CDK12 for ER/MED1 interactions, with a significant decrease in their endogenous protein-protein interaction after *CDK12* knockdown (Figure 4C). Additionally, consistent with ChIP-seq findings (Figure 2), *CDK12* knockdown led to reduced ER and MED1 recruitment to the classic ER target gene *GREB1* in ChIP-qPCR assays (Figure 4D). Further supporting these observations, ChIP-qPCR experiments confirmed that CDK12, along with ER, and MED1, are recruited to classic ER target genes (Figure 4E, Figure S4D), reinforcing the role of CDK12 in regulating ER-associated transcription. Of interest, knockdown of *ESR1* in LY2 cells resulted in loss of nuclear CDK12, MED1, and p-MED1, further illustrating the significant collaboration between ER and CDK12 in the endocrine-resistant setting (Figure S6E-F).

Taken together, the data suggest that CDK12 plays a functional role in regulating the ER/MED1 cistrome, being essential for full ER chromatin recruitment. These findings highlight CDK12 involvement in maintaining pro-tumorigenic ER signaling in advanced ER+ breast cancer, by modulation of ER and MED1 chromatin binding (Figure 4F).

### Pharmacological inhibition of CDK12 is detrimental to ER+ breast cancer cells

**Figure 5.**
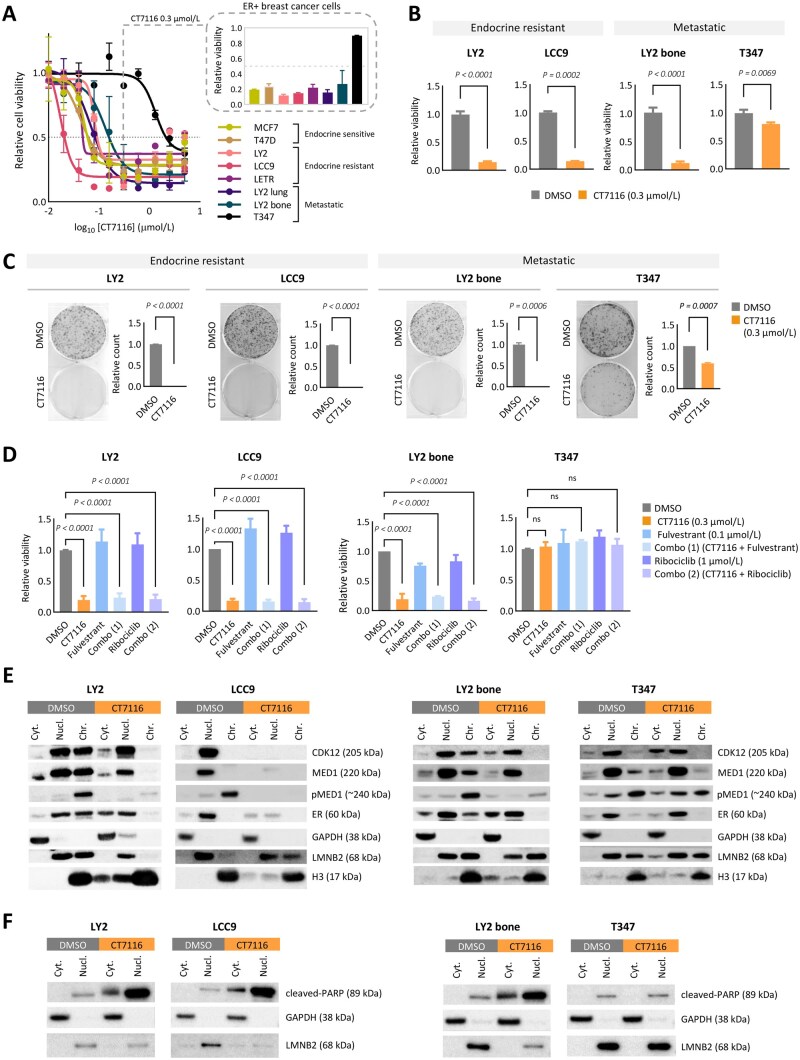
Pharmacological inhibition of CDK12 in endocrine-resistant and metastatic ER+ breast cancer cell models. **A**) Dose-response analysis of ER+ breast cancer cell lines treated with CT7116 (CDK12/13 inhibitor) for 72 hours. Shown are curves for endocrine-sensitive (MCF7, T47D), endocrine-resistant (LY2, LCC9, LETR), and metastatic (LY2 bone, LY2 lung, T347 brain) cell models. Gray arrow indicates the selected concentration (0.3 µmol/L) for follow-up studies, with corresponding relative viability of each model at this dose. **B**) Cell viability of endocrine-resistant (LY2, LCC9) and metastatic (LY2 bone, T347) cell models after CT7116 treatment (0.3 µmol/L, 72 hours). Bar graphs represent viability relative to DMSO control. Error bars denote mean ± SD from 3 independent experiments. Statistical significance calculated by 2-tailed unpaired *t* test: LY2, *P* < .0001; LY2 bone, *P* < .0001; T347, *P* = .0069; LCC9, *P* = .0002 (Welch’s correction). **C**) Cell colony formation assay in endocrine resistant (LY2, LCC9) and metastatic (LY2 bone, T347) cells after prolonged CT7116 (0.3 µmol/L) exposure: 13 days for LY2, LY2 bone, and LCC9; 10 days for T347. Images of representative wells and quantification of clonogenic growth (CT7116 relative to control DMSO) are shown. Error bars indicate mean ± SD of 3 independent replicates. Statistical *P*-values were calculated by 2-tailed unpaired *t* test: LY2, *P* < .0001; LCC9, *P* < .0001; LY2 bone, *P* = .0006 (Welch’s correction); T347, *P* = .0007 (Welch’s correction). **D**) Cell viability after combo treatment with CT7116, fulvestrant, and ribociclib. Evaluation of CT7116 as monotherapy or in combination with standard-of-care agents, in endocrine resistant (LY2, LCC9) and metastatic (LY2 bone, T347) cells. Bar plots represent relative cell viability after 72-hour treatment. Combination groups: Combo:^1^ CT7116 (0.3 µmol/L) + fulvestrant (0.1 µmol/L); Combo:^2^ CT7116 (0.3 µmol/L) + ribociclib (1 µmol/L). Error bars indicate mean ± SD of 3 independent experiments. Statistical analysis by ordinary 1-way ANOVA with multiple comparison correction. LY2, LCC9, and LY2 bone: CT7116, Combo,^1^ Combo^2^ (adj. *P* < .0001). T347: CT7116 (adj. *P* = .9870), Combo^1^ (adj. *P* = .55261), Combo^2^ (adj. *P* = .8927). **E**) Immunoblot analysis of CDK12, MED1, pMED1, and ER protein expression in subcellular fractions cytoplasmic (Cyt.), nuclear (Nucl.), and chromatin-bound (Chr.), after CT7116 treatment (0.3 µmol/L, 24 hours) in endocrine resistant (LY2, LCC9) and metastatic (LY2 bone, T347) cells. **F**) Immunoblot detection of cleaved PARP after CT7116 treatment (0.3 µmol/L, 24 hours) in endocrine resistant (LY2, LCC9) and metastatic (LY2 bone, T347) cells. **E-F**) Molecular weights of immunoblotted proteins indicated in kDa. Blots are representative of 3 independent experiments. GAPDH, LMNB2, and H3 were used as loading controls for Cyt., Nucl., and Chr. fractions, respectively.

To assess the therapeutic relevance of our findings, we tested the CDK12/13 inhibitor CT7116 (Carrick Therapeutics) in a panel of ER+ breast cancer cells: endocrine-sensitive (MCF7, T47D), endocrine-resistant (LY2, LCC9, LETR), and metastatic models (LY2 bone, LY2 lung, T347) (Table S6). Cell viability studies with increasing CT7116 concentrations (0.02-5 µmol/L, 72 hours) identified 0.3 µmol/L as the optimal dose (Figure 5A). CT7116 treatment significantly reduced cell viability (72 hours; Figure 5B) and colony growth (>10 days; Figure 5C) in endocrine-resistant (LY2, LCC9) and metastatic (LY2 bone, T347) models. Notably, the T347 patient-derived brain metastatic tumor, harboring a somatic N885K mutation CDK12’s kinase domain^24^ (Table S6), showed reduced sensitivity to short-term CDK12 inhibition (Figure 5B and D). Treatment of LY2 cells with an alternative CDK12/13 inhibitor (THZ531) reduced viability and colony growth, with slightly weaker effects than CT7116 at the same concentration (Figure S7A, B).

Pharmacological inhibition of CDK12 with CT7116 significantly decreased the viability of endocrine-resistant and metastatic cell models compared with standard therapies, fulvestrant (selective ER degrader) and ribociclib (CDK4/6 inhibitor) (Figure 5D). No synergy was observed with combined CT7116 and fulvestrant or ribociclib treatment (Figure S7C, D).

Consistent with our *CDK12* knockdown studies (Figure 4A), pharmacological inhibition of CDK12 reduced nuclear and chromatin-bound CDK12, ER, MED1 and pMED1 proteins (Figure 5E). In LCC9 cells, the most sensitive to CT7116 (Figure 5A, Table S6), protein expression was completely abrogated after treatment. In contrast, minimal reduction was observed in the endocrine-sensitive T47D cell line, with notable effect only on pMED1 levels (Figure S8A). No detectable effect was observed in the CDK12-mutant T347 metastatic cells.

CDK12 and its binding partner cyclin K are key regulators of transcriptional elongation through phosphorylation of the RNA polymerase II C-terminal domain (CTD).^9-11^ Treatment with the CDK12 inhibitor CT7116 resulted in a marked loss of CDK12-associated proteins, including CDK13 and cyclin K, specifically in the nuclear and chromatin-bound fractions of all ER+ cell models, except for the CDK12-mutant T347 cells (Figure S8B). Additionally, CT7116 treatment led to a global reduction in Ser2 phosphorylation of the RNA Pol II-CTD (pSer2 CTD), along with decreased total CTD expression levels (Figure S8B), confirming effective inhibition of CDK12-mediated transcriptional elongation.

Functionally, consistent with apoptosis being a key CDK12-regulated pathway (Figure 2C), pharmacological inhibition of CDK12 with CT7116 induced apoptosis, demonstrated by cleaved-PARP protein expression (Figure 5F), similar to *CDK12* knockdown (Figure S4E). As previously observed, CT7116 treatment failed to induce apoptosis in the CDK12-mutated brain metastatic T347 cell model (Figure 5F).

### Pharmacological targeting of CDK12 reduces viability and metastatic spread in advanced ER+ breast cancer models

**Figure 6.**
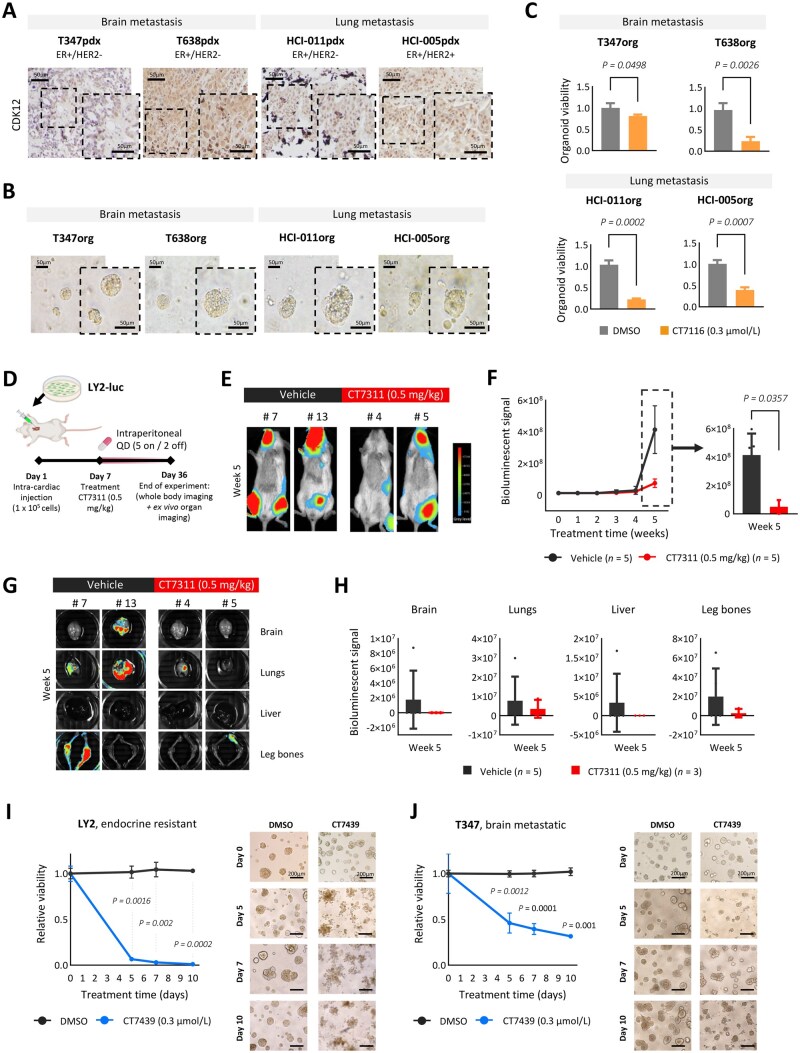
CDK12 inhibition reduces viability and metastatic spread of ER+ breast cancer models. **A**) Immunohistochemical characterization of CDK12 in metastatic ER+ PDX models. Brain metastatic: T347pdx and T638pdx. Lung metastatic: HCI-011pdx and HCI-005pdx. Scale bar at 50 µm. **B**) Representative brightfield images of established organoid cultures (7 days) from metastatic PDX models. Brain metastatic: T347org and T638org. Lung metastatic: HCI-011org and HCI-005org. Scale bar at 50 μm. **C**) Viability assay in metastatic organoid models treated with CT7116 (0.3 µmol/L) for 7 days relative to control (DMSO). Error bars indicate mean values ± SD of 3 independent experiments. Statistical *P*-values were calculated by 2-tailed unpaired *t* test (T347org: *P* = .0498, T638org: *P* = .0026, HCl-011org: *P* = .0002, HCl-005: *P* = .0007). **D**) NOD SCID mice experiment setup. Intracardiac injection of luciferase-tagged LY2 cells (LY2-luc) in NOD SCID female mice treated with vehicle (*n* = 5) or CDK12 inhibitor (CT7311, 0.5 mg/kg) (*n* = 5) by single intraperitoneal (QD) injections (5 on/2 off) for 5 weeks (end of experiment: day 36). **E**) Representative images of whole-body bioluminescence signal of LY2-luc dissemination under CT7311 treatment. Mice treated with vehicle (#7, #13) or CT7311 (#4, #5) at week 5 (terminal) of the experiment. **F**) Whole-body metastatic spread. Quantification of whole-body bioluminescent signal evaluating dissemination of LY2-luc. Error bars represent mean values ± SD. Statistical *P*-values were calculated by Mann-Whitney test (CT7311 vs vehicle: *P* = .0357). **G**) Representative images of ex vivo harvested organs (brain, lungs, liver, and leg bones) from mice treated with vehicle (#7, #13) or CT7311 (#4, #5) at week 5 (terminal) of the experiment. **H**) Quantification of distant metastatic spread (bioluminescent signaling explant organs) in brain, lungs, liver, and leg bones from animals treated with either vehicle or CT7311 at week 5. Error bars represent mean values ± SD. Statistical *P*-values were calculated by Mann-Whitney test (CT7311 vs vehicle. Brain: *P* > .9999, lungs: *P* = .7857, liver: *P* > .9999, leg bones: *P* = .6429). **I-J**) Effect of CT7349 treatment on **(I)** LY2 endocrine-resistant and **(J)** T347 metastatic breast cancer 3D spheroids. Graph shows quantification of spheroid viability over time after CT7349 treatment (0.3 µmol/L), showing a significant reduction in viability in the treated group (blue line) compared with the control (DMSO, black line). Data are presented as mean ± SD (*n* = 3). Statistical *P*-values were calculated by 2-tailed unpaired *t* test (T347, Day 5: *P* = .012, Day 7: *P* = .0001), with Welch’s corrections (T347, Day 10: *P* = .0010; LY2, Day 5: *P* = .0016, Day 7: *P* = .0020, Day 10: *P* = .0002). Representative brightfield images of 3D spheroids: the left columns show control (DMSO), and the right column shows treated spheroids at matching time points (days 0, 5, 7, 10). Scale bars: 200 µm. The images illustrate morphological differences and a reduction in spheroid size and integrity after treatment.

The antitumor efficacy of CT7116 was tested in organoid cultures derived from ER+/CDK12-positive patient-derived xenograft (PDX) models (Figure 6A, B). Patient-derived xenografts were previously established from 2 brain (T347, T638)^24^ and 2 lung (HCI05org, HCI-011org)^42^ metastatic tumors (Figure 6C, Table S6). CT7116 treatment (7 days) significantly reduced organoid viability in both brain (T347org and T638org) and lung (HCI05org and HCI-011org) models (Figure 6D). Consistent with our in vitro findings, the CDK12-mutated T347org model showed reduced sensitivity to CT7116 compared with the other models.

In vivo and organoid studies using the CT7116 analogs with improved pharmacokinetics and bioavailability, CT7311 and CT7439 (validated in vitro; Figure S9A, B), were conducted in advanced ER+ cell and patient-derived models. After drug de-escalation studies in female NOD SCID mice (intraperitoneal injection, 5 on/2 off schedule), 0.5 mg/kg CT7311 was identified as the effective tolerable dose, with no significant weight loss (Figure S9C). To model cancer cell dissemination, luciferase-tagged LY2 cells (LY2-luc) were injected intracardially (Figure 6E). Mice were treated for 35 days with either vehicle or CT7311 (0.5 mg/kg, 5 on/2 off), with weekly in vivo IVIS imaging. Whole-body metastatic spread (LY2-luc dissemination) was significantly reduced in CT7311-treated mice compared with vehicle (*P *= .0079) (Figure 6F-G, Figure S9D). LY2-luc dissemination to individual organs, including brain, lungs, liver, and bone, was also reduced, although not statistically significant (Figure 6H-I, Figure S9E). To further test the durability of CDK12/13 inhibition, we used CT7439 in both cell and patient-derived organoid models. Treatment successfully diminished organoid viability over time (0-10 days) in both the LY2 endocrine resistant cell model and to a lesser extent in the patient-derived T347 brain metastatic model (Figure 6I).

## Discussion

Advanced breast cancer is an aggressive disease, with metastasis progression driven by genetic adaptations that dysregulate transcriptional programs.^43^ Emerging evidence suggests that these cancers are transcriptionally driven, highlighting the central role of gene modulators, such as CDKs, in disease progression.^3^

Previous work from our group identified significant genomic and transcriptomic alterations on brain metastasis, including gains in *ERBB2*/HER2 and kinase signaling.^24^ Here we describe recurrent amplifications of the transcriptional CDK, *CDK12,* not only in brain metastatic tumors but also in the primary tumors from which they arise. The frequency of *CDK12* amplifications reported here is higher than in other reported breast cancer cohorts;^27-32^ this may be due at least in part to the more aggressive nature of the patient tumors reported here. *CDK12*, located on chromosome 17q12, ∼200 kb from *ERBB2*, is co-amplified with *ERBB2* in our primary and brain metastatic tumors, consistent with previous reports.^21^ In addition to amplifications, *CDK12* fusions were found to occur most frequently in brain metastatic tumors compared with other fusion events. These fusions were associated with high gene expression of CDK12, although their significant relevance remains to be elucidated. At the transcriptional level, significant expression gains were noted in metastasis relative to matched primary tumors, and high, druggable CDK12 protein levels were observed in more than 90% of our cohort of primary breast cancer tumors. Despite *CDK12/ERBB2* co-amplification, high clinically relevant protein expression was found mostly in ER+ tumors, suggesting CDK12 has a significant impact in driving progression in this patient population. CDK12 was not an independent predictor of survival in HER2-positive patients, which may reflect the dominant influence of HER2 signaling in driving disease progression in this subgroup, potentially masking the contribution of transcriptional CDKs such as CDK12.

CDK12 transcriptional regulation in cancer has been linked to dysregulation of DNA damage repair and DNA damage response (DDR) genes.^13^^,^^15^ In breast cancer, CDK12 is involved in TNBC and HER2+ disease progression through regulation of c-Myc/WNT/β-catenin and ErbB-PI3K-AKT signaling.^20^^,^^44^^,^^45^ In this study, we identify estrogen signaling as a novel core mechanistic output of aberrant CDK12 expression in advanced ER+ breast cancer models.

Estrogen response genes, such as *ESR1* and canonical ER targets *NRIP1*, *GREB1*, and *ABCA3*, were found to be CDK12-dependent. Consistent with previous findings in ER-negative disease,^18^^,^^46^ cell proliferation, cell cycle progression, and DNA repair were also regulated by CDK12 in our ER+ models.

Considering the highly selective nature of CDK12 regulation, its broad involvement in conventional transcriptional elongation processes is unlikely to represent its only mechanism of action. However, transcriptional processing mediated by CDK12, in collaboration with specific transcription factor partners, has not been previously characterized. In this study, ER and the mediator complex protein MED1 were identified as potential CDK12 transcriptional interactors. CDK12 was confirmed as a critical member of the ER/MED1 regulatory complex, particularly influencing the ER transcriptome.

CDK12 transcriptional regulation has been linked not only to its direct control of RNA Pol II processivity, as seen with core DNA replication and DDR genes,^12-15^ but also to indirect mechanisms through its involvement in regulating other oncogenic pathways.^20^^,^^21^^,^^44^^,^^47^ In this study, CDK12 inhibition led to a loss of CDK13 and cyclin K chromatin binding in ER+ models. Additionally, we observed a global reduction of Ser2 phosphorylation of RNA Pol II’s CTD and decreased RNA Pol II-CTD expression, indicating effective inhibition of CDK12’s transcription elongation function. These findings suggest that CDK12, through the ER/MED1 regulatory complex, mediates targeted gene expression, at least partially by directly controlling RNA Pol II processivity, establishing a new tightly regulated mechanism for CDK12 in advanced ER+ disease.

In recent years, targeting transcription associated CDKs for breast cancer treatment has gained significant attention.^48-52^ Pharmacological inhibition of CDK7 has shown significant efficacy in reducing tumor burden in preclinical breast cancer models.^49^^,^^51^^,^^53^^,^^54^ Recently, the selective oral CDK7 inhibitor samuraciclib demonstrated an acceptable safety profile in a multimodular, open label, phase I study.^55^ Early efficacy data from this trial suggest CDK7 inhibition may be a viable strategy for patients resistant to CDK4/6-directed therapy. Moreover, a phase 1 clinical trial (NCT06600789) evaluating CT7439, a novel CDK12/13 inhibitor/Cyclin K glue-degrader, has recently been initiated and will enroll patients with advanced solid tumors, including breast cancer.

To date, preclinical studies on CDK12 inhibition in breast cancer have focused primarily on HER2+ and TNBC molecular subtypes.^18^^,^^45^ In in vitro and ex vivo ER+ models of endocrine resistance and metastasis, inhibition of CDK12 with the small molecule inhibitor CT7116 significantly reduced tumor cell viability, except in the T347 brain metastatic model, which harbors a CDK12 kinase domain mutation (N885K) likely to alter CDK12 catalytic activity. Furthermore, in vivo treatment with the CDK12 inhibitor CT7311 significantly reduced distant metastatic disease burden.

This study is not without limitations; matched copy number and protein data for a large cohort of patient samples would enable us to determine the definitive relationship between CDK12 amplifications and target expression. Additionally, studies to understand the functional significance of CDK12 fusions and further pharmacological in vivo studies would be beneficial. This work, however, provides the first comprehensive characterization of CDK12 in ER+ advanced breast cancer. We identify a selective transcriptional program through which CDK12 uses the ER/MED1 complex to drive an aggressive phenotype. Clinically significant, druggable CDK12 protein levels were detected in ER+ patient populations, and preclinical evidence supports the potential of CDK12 inhibition to affect metastatic progression in ER+ advanced disease. Further clinical studies are warranted to evaluate the feasibility of targeting CDK12 as a therapeutic strategy for advanced ER+ breast cancer.

## Supplementary Material

djaf295_Supplementary_Data

## Data Availability

**Breast cancer models.** Processed RNA-seq and ChIP-seq data from studies in LY2 cells are available in *figshare*, at https://doi.org/10.6084/m9.figshare.30618569 (RNA-seq), and https://doi.org/10.6084/m9.figshare.30618884 (ChIP-seq). **Patient samples. **Whole exome sequencing (*n* = 39 patients) and RNA sequencing (*n* = 45 patients) data from matched primary breast and brain metastatic tumors have been previously described and made available by Cosgrove et al.^24^ Processed RNA-sequencing data (*n* = 45) is available in *Gene Expression Omnibus (GEO)*, at https://www.ncbi.nlm.nih.gov/geo/, and can be accessed with accession number GSE184869. Processed Whole exome-seq data (*n* = 18) is available in *figshare*, at https://figshare.com/, and can be accessed with 10.6084/m9.figshare.16685680.v1. Processed Whole exome-sequencing data (*n* = 21) can be downloaded upon request from the database of *Genotypes and Phenotypes (dbGaP)*, at https://dbgap.ncbi.nlm.nih.gov/, and can be accessed with accession number phs000730.v1.pl.

## References

[1] Bradner JE , Hnisz D, Young RA. Transcriptional addiction in cancer. Cell. 2017;168:629-643. doi: 10.1016/j.cell.2016.12.013 28187285 PMC5308559

[2] Lee TI , Young RA. Transcriptional regulation and its misregulation in disease. Cell. 2013;152:1237-1251. doi: 10.1016/J.CELL.2013.02.014/ATTACHMENT/F2B97A73-A5C0-4DB6-A48C-850CA6A0D978/MMC1.PDF 23498934 PMC3640494

[3] Chou J , Quigley DA, Robinson TM, Feng FY, Ashworth A. Transcription-associated cyclin-dependent kinases as targets and biomarkers for cancer therapy. Cancer Discov. 2020;10:351-370. doi: 10.1158/2159-8290.CD-19-0528 32071145

[4] Vervoort SJ , Devlin JR, Kwiatkowski N, Teng M, Gray NS, Johnstone RW. Targeting transcription cycles in cancer. Nat Rev Cancer. 2022;22:5-24. doi: 10.1038/s41568-021-00411-8 34675395

[5] Parua PK , Fisher RP. Dissecting the Pol II transcription cycle and derailing cancer with CDK inhibitors. Nat Chem Biol. 2020;16:716-724. doi: 10.1038/s41589-020-0563-4 32572259 PMC7914107

[6] Buratowski S. Progression through the RNA polymerase II CTD cycle. Mol Cell. 2009;36:541-546. doi: 10.1016/j.molcel.2009.10.019 19941815 PMC3232742

[7] Schier AC , Taatjes DJ. Structure and mechanism of the RNA polymerase II transcription machinery. Genes Dev. 2020;34:465-488. doi: 10.1101/GAD.335679.119 32238450 PMC7111264

[8] Kohoutek J , Blazek D. Cyclin K goes with CDK12 and CDK13. Cell Div. 2012;7:12-10. doi: 10.1186/1747-1028-7-12 22512864 PMC3348076

[9] Liang K , Gao X, Gilmore JM, et al Characterization of human cyclin-dependent kinase 12 (CDK12) and CDK13 complexes in C-terminal domain phosphorylation, gene transcription, and RNA processing. Mol Cell Biol. 2015;35:928-938. doi: 10.1128/mcb.01426-14 25561469 PMC4333096

[10] Fan Z , Devlin JR, Hogg SJ, et al CDK13 cooperates with CDK12 to control global RNA polymerase II processivity. Sci Adv. 2020;6:eaaz5041-doi: 10.1126/sciadv.aaz5041 32917631 PMC7190357

[11] Cheng SWG , Kuzyk MA, Moradian A, et al Interaction of cyclin-dependent kinase 12/CrkRS with cyclin K1 is required for the phosphorylation of the C-terminal domain of RNA polymerase II. Mol Cell Biol. 2012;32:4691-4704. doi: 10.1128/MCB.06267-11 22988298 PMC3486194

[12] Blazek D , Kohoutek J, Bartholomeeusen K, et al The cyclin K/CDK12 complex maintains genomic stability via regulation of expression of DNA damage response genes. Genes Dev. 2011;25:2158-2172. doi: 10.1101/gad.16962311 22012619 PMC3205586

[13] Dubbury SJ , Boutz PL, Sharp PA. CDK12 regulates DNA repair genes by suppressing intronic polyadenylation. Nature. 2018;564:141-145. doi: 10.1016/j.physbeh.2017.03.040 30487607 PMC6328294

[14] Chirackal Manavalan AP , Pilarova K, Kluge M, et al CDK12 controls G1/S progression by regulating RNAPII processivity at core DNA replication genes. EMBO Rep. 2019;20:e47592-doi: 10.15252/embr.201847592 31347271 PMC6727028

[15] Krajewska M , Dries R, Grassetti AV, et al CDK12 loss in cancer cells affects DNA damage response genes through premature cleavage and polyadenylation. Nat Commun. 2019;10:1757-16. doi: 10.1038/s41467-019-09703-y 30988284 PMC6465371

[16] Popova T , Manie E, Boeva V, et al Ovarian cancers harboring inactivating mutations in CDK12 display a distinct genomic instability pattern characterized by large tandem duplications. Cancer Res. 2016;76:1882-1891. doi: 10.1158/0008-5472.CAN-15-2128 26787835

[17] Wu YM , Cieślik M, Lonigro RJ, et al; PCF/SU2C International Prostate Cancer Dream Team. Inactivation of CDK12 delineates a distinct immunogenic class of advanced prostate cancer. Cell. 2018;173:1770-1782.e14. doi: 10.1016/j.cell.2018.04.034 29906450 PMC6084431

[18] Quereda V , Bayle S, Vena F, et al Therapeutic targeting of CDK12/CDK13 in triple-negative breast cancer. Cancer Cell. 2019;36:545-558.e7. doi: 10.1016/j.ccell.2019.09.004 31668947

[19] Naidoo K , Wai PT, Maguire SL, et al Evaluation of CDK12 protein expression as a potential novel biomarker for DNA damage response-targeted therapies in breast cancer. Mol Cancer Ther. 2018;17:306-315. doi: 10.1158/1535-7163.MCT-17-0760 29133620 PMC6284786

[20] Choi H , Jin S, Cho H, et al CDK12 drives breast tumor initiation and trastuzumab resistance via WNT and IRS1-ErbB-PI3K signaling. EMBO Rep. 2019;20:e48058. doi: 10.15252/embr.201948058 31468695 PMC6776914

[21] Yang Y , Leonard M, Luo Z, et al Functional cooperation between co-amplified genes promotes aggressive phenotypes of HER2-positive breast cancer. Cell Rep. 2021;34:108822. doi: 10.1016/J.CELREP.2021.108822 33691110 PMC8050805

[22] Bhogal T , Giannoudis A, Sokol E, Ali S, Palmieri C. Analysis of breast cancer brain metastases reveals an enrichment of cyclin-dependent kinase 12 structural rearrangements in human epidermal growth factor receptor 2–positive disease. J Clin Oncol Precis Oncol. 2024;8:e2300639. doi: 10.1200/PO.23.00639 38838276

[23] Forster-Sack M , Zoche M, Pestalozzi B, et al ERBB2-amplified lobular breast carcinoma exhibits concomitant CDK12 co-amplification associated with poor prognostic features. J Pathol Clin Res. 2024;10:e12362. doi: 10.1002/2056-4538.12362 38335502 PMC10800294

[24] Cosgrove N , Varešlija D, Keelan S, et al Mapping molecular subtype specific alterations in breast cancer brain metastases identifies clinically relevant vulnerabilities. Nat Commun. 2022;13:514-516. doi: 10.1038/s41467-022-27987-5 35082299 PMC8791982

[25] Sachs N , de Ligt J, Kopper O, et al A living biobank of breast cancer organoids captures disease heterogeneity. Cell. 2018;172:373-386.e10. doi: 10.1016/J.CELL.2017.11.010 29224780

[26] Zhu LJ , Gazin C, Lawson ND, et al ChIPpeakAnno: a Bioconductor package to annotate ChIP-seq and ChIP-chip data. BMC Bioinformatics. 2010;11:237. doi: 10.1186/1471-2105-11-237 20459804 PMC3098059

[27] Curtis C , Shah SP, Chin SF, et al; METABRIC Group. The genomic and transcriptomic architecture of 2,000 breast tumours reveals novel subgroups. Nature. 2012;486:346-352. doi: 10.1038/NATURE10983 22522925 PMC3440846

[28] Pereira B , Chin SF, Rueda OM, et al The somatic mutation profiles of 2,433 breast cancers refines their genomic and transcriptomic landscapes. Nat Commun. 2016;7:11479. doi: 10.1038/NCOMMS11479 27161491 PMC4866047

[29] Rueda OM , Sammut SJ, Seoane JA, et al Dynamics of breast-cancer relapse reveal late-recurring ER-positive genomic subgroups. Nature. 2019;567:399-404. doi: 10.1038/s41586-019-1007-8 30867590 PMC6647838

[30] Cerami E , Gao J, Dogrusoz U, et al The cBio Cancer Genomics Portal: an open platform for exploring multidimensional cancer genomics data. Cancer Discov. 2012;2:401-404. doi: 10.1158/2159-8290.CD-12-0095 22588877 PMC3956037

[31] Gao J , Aksoy BA, Dogrusoz U, et al Integrative analysis of complex cancer genomics and clinical profiles using the cBioPortal. Sci Signal. 2013;6:pl1. doi: 10.1126/scisignal.2004088 23550210 PMC4160307

[32] de Bruijn I , Kundra R, Mastrogiacomo B, et al; AACR Project GENIE BPC Core Team, AACR Project GENIE Consortium. Analysis and visualization of longitudinal genomic and clinical data from the AACR project GENIE biopharma collaborative in cBioPortal. Cancer Res. 2023;83:3861-3867. doi: 10.1158/0008-5472.CAN-23-0816 37668528 PMC10690089

[33] Angus L , Smid M, Wilting SM, et al The genomic landscape of metastatic breast cancer highlights changes in mutation and signature frequencies. Nat Genet. 2019;51:1450-1458. doi: 10.1038/s41588-019-0507-7 31570896 PMC6858873

[34] Gao Q , Liang WW, Foltz SM, et al; Cancer Genome Atlas Research Network. Driver fusions and their implications in the development and treatment of human cancers. Cell Rep. 2018;23:227-238.e3. doi: 10.1016/j.celrep.2018.03.050 29617662 PMC5916809

[35] Lánczky A , Győrffy B. Web-based survival analysis tool tailored for medical research (KMplot): development and implementation. J Med Internet Res. 2021;23:e27633. doi: 10.2196/27633 34309564 PMC8367126

[36] Qin Q , Fan J, Zheng R, et al Lisa: inferring transcriptional regulators through integrative modeling of public chromatin accessibility and ChIP-seq data. Genome Biol. 2020;21:32-14. doi: 10.1186/S13059-020-1934-6/FIGURES/6 32033573 PMC7007693

[37] Li Z , Li T, Yates ME, et al The EstroGene database reveals diverse temporal, context-dependent, and bidirectional estrogen receptor regulomes in breast cancer. Cancer Res. 2023;83:2656-2674. doi: 10.1158/0008-5472.CAN-23-0539 37272757 PMC10527051

[38] Varešlija D , Mcbryan J, Fagan A, et al Adaptation to AI therapy in breast cancer can induce dynamic alterations in ER activity resulting in estrogen-independent metastatic tumors. Clin Cancer Res. 2016;22:2765-2777. doi: 10.1158/1078-0432.CCR-15-1583 26763249

[39] Zhang X , Krutchinsky A, Fukuda A, et al MED1/TRAP220 exists predominantly in a TRAP/mediator subpopulation enriched in RNA polymerase II and is required for ER-mediated transcription. Mol Cell. 2005;19:89-100. doi: 10.1016/J.MOLCEL.2005.05.015 15989967

[40] Park SJ , Yoon BH, Kim SK, Kim SY. GENT2: an updated gene expression database for normal and tumor tissues. BMC Med Genomics. 2019;12:101. doi: 10.1186/S12920-019-0514-7 31296229 PMC6624177

[41] Tsherniak A , Vazquez F, Montgomery PG, et al Defining a cancer dependency map. Cell. 2017;170:564-576.e16. doi: 10.1016/j.cell.2017.06.010 28753430 PMC5667678

[42] Guillen KP , Fujita M, Butterfield AJ, et al A human breast cancer-derived xenograft and organoid platform for drug discovery and precision oncology. Nat Cancer. 2022;3:232-250. 35221336 10.1038/s43018-022-00337-6 PMC8882468

[43] Ell B , Kang Y. Transcriptional control of cancer metastasis. Trends Cell Biol. 2013;23:603-611. doi: 10.1016/j.tcb.2013.06.001 23838335 PMC3815486

[44] Peng F , Yang C, Kong Y, et al CDK12 promotes breast cancer progression and maintains stemness by activating c-myc/β-catenin signaling. Curr Cancer Drug Targets. 2020;20:156-165. doi: 10.2174/1568009619666191118113220 31744448

[45] Li H , Wang J, Yi Z, et al CDK12 inhibition enhances sensitivity of HER2+ breast cancers to HER2-tyrosine kinase inhibitor via suppressing PI3K/AKT. Eur J Cancer. 2021;145:92-108. doi: 10.1016/j.ejca.2020.11.045 33429148

[46] Tien JF , Mazloomian A, Cheng SWG, et al CDK12 regulates alternative last exon mRNA splicing and promotes breast cancer cell invasion. Nucleic Acids Res. 2017;45:6698-6716. doi: 10.1093/nar/gkx187 28334900 PMC5499812

[47] Filippone MG , Gaglio D, Bonfanti R, et al CDK12 promotes tumorigenesis but induces vulnerability to therapies inhibiting folate one-carbon metabolism in breast cancer. Nat Commun. 2022;13:2642-19. doi: 10.1038/s41467-022-30375-8 35550508 PMC9098894

[48] Li Y , Zhang H, Li Q, et al CDK12/13 inhibition induces immunogenic cell death and enhances anti-PD-1 anticancer activity in breast cancer. Cancer Lett. 2020;495:12-21. doi: 10.1016/j.canlet.2020.09.011 32941949

[49] McDermott MSJ , Sharko AC, Munie J, et al CDK7 inhibition is effective in all the subtypes of breast cancer: determinants of response and synergy with EGFR inhibition. Cells. 2020;9:638. doi: 10.3390/cells9030638 32155786 PMC7140476

[50] McDermott MSJ , Chumanevich AA, Lim CU, et al Inhibition of CDK8 mediator kinase suppresses estrogen dependent transcription and the growth of estrogen receptor positive breast cancer. Oncotarget. 2017;8:12558-12575. doi: 10.18632/ONCOTARGET.14894 28147342 PMC5355036

[51] Wang Y , Zhang T, Kwiatkowski N, et al CDK7-dependent transcriptional addiction in triple-negative breast cancer. Cell. 2015;163:174-186. doi: 10.1016/j.cell.2015.08.063 26406377 PMC4583659

[52] Diab S , Yu M, Wang S. CDK7 inhibitors in cancer therapy: the sweet smell of success? J Med Chem. 2020;63:7458-7474. doi: 10.1021/acs.jmedchem.9b01985 32150405

[53] Patel H , Abduljabbar R, Lai CF, et al Expression of CDK7, cyclin H, and MAT1 is elevated in breast cancer and is prognostic in estrogen receptor-positive breast cancer. Clin Cancer Res. 2016;22:5929-5938. doi: 10.1158/1078-0432.CCR-15-1104 27301701 PMC5293170

[54] Sun B , Mason S, Wilson RC, et al Inhibition of the transcriptional kinase CDK7 overcomes therapeutic resistance in HER2-positive breast cancers. Oncogene. 2020;39:50-63. doi: 10.1038/s41388-019-0953-9 31462705 PMC6937212

[55] Coombes RC , Howell S, Lord SR, et al Dose escalation and expansion cohorts in patients with advanced breast cancer in a phase I study of the CDK7-inhibitor samuraciclib. Nat Commun. 2023;14:4444-10. doi: 10.1038/s41467-023-40061-y 37488191 PMC10366102

