# Supplementary material for: Targeting CDK12 disrupts estrogen-receptor chromatin recruitment and ER-MED1 transcription in advanced ER+ breast cancer
Source: J Natl Cancer Inst. 2025 Oct 15;118(3):404–21. doi: 10.1093/jnci/djaf295 (PMC13016824; doi:10.1093/jnci/djaf295)
Supplement: djaf295_Supplementary_Data [file djaf295_supplementary_data.zip › Ottaviani et al. CDK12_Supplementary Material.docx]

**Targeting CDK12 disrupts estrogen-receptor chromatin recruitment and ER-MED1 transcription in advanced ER+ breast cancer**

Daniela Ottaviani^1*^, PhD, Mihaela Ola^1^, PhD, Alessandra Allotta^1^, MSc, Yasmine Maati Chaibi^1^, MSc, Seán Hickey^1^, BSc, Petra Jagust^1^, PhD, Nicola Cosgrove^1^, PhD, Sinéad Cocchiglia^1^, MSc, Fiona Bane^1^, MSc, Ramón Fallon^1^, MSc, Gordon Daly^1^, MCh, Aisling Hegarty^1,2^, PhD, Lance Hudson^1,2^, BSc, Katherine Sheehan^1,3^, MD, Shannon Kalsi^1^, MSc, Stephen Shovlin^1^, PhD, Aoibhín Powell^1,4^, BSc, Ash Bahl^5^, PhD, Ed Ainscow^5^, PhD, Steffi Oesterreich^6,7^, PhD, Adrian V. Lee^6,7^, PhD, Fergus J. Couch^8^, PhD, Arnold D.K. Hill^9^, MD, Damir Varešlija^1,4*^, PhD, and Leonie Young^1,2,9*#^, PhD.

**Supplementary Material**

Original, uncropped tissue microarray and western blot images corresponding to the main and supplementary figures of this study.

Index

[Supplementary Material 1. Uncropped IHC images in Figure 1E. 3](#_Toc204598856)

[Supplementary Material 2. Uncropped IHC images in Figure 3D. 6](#_Toc204598857)

[Supplementary Material 3. Uncropped western blot membranes in Figure 4A. 9](#_Toc204598858)

[LY2 cells 9](#_Toc204598859)

[Supplementary Material 4. Uncropped western blot membranes in Figure 4B. 14](#_Toc204598860)

[LY2 - IP ER 14](#_Toc204598861)

[LY2 - IP MED1 17](#_Toc204598862)

[Supplementary Material 5. Uncropped western blot membranes in Figure 5E. 20](#_Toc204598863)

[LY2 cells 20](#_Toc204598864)

[LCC9 cells 25](#_Toc204598865)

[LY2 bone cells 31](#_Toc204598866)

[T347 cells 37](#_Toc204598867)

[Supplementary Material 6. Uncropped western blot membranes in Figure 5F. 44](#_Toc204598868)

[LY2 cells 44](#_Toc204598869)

[LCC9 cells 47](#_Toc204598870)

[LY2 bone cells 50](#_Toc204598871)

[T347 cells 53](#_Toc204598872)

[Supplementary Material 7. Uncropped western blot membranes in Supplementary Figure 3E. 56](#_Toc204598873)

[LY2 cells 56](#_Toc204598874)

[Supplementary Material 8. Uncropped western blot membranes in Supplementary Figure 7A. 59](#_Toc204598875)

[T47D cells 59](#_Toc204598876)

[LY2 cells 66](#_Toc204598877)

[LCC9 73](#_Toc204598878)

[LY2 bone cells 80](#_Toc204598879)

[T347 87](#_Toc204598880)

[Supplementary Material 10. Uncropped western blot membranes in Supplementary figure 6B. 94](#_Toc204598881)

[T47D cells 94](#_Toc204598882)

[Supplementary Material 11. Uncropped western blot membranes in Supplementary Figure 6C. 99](#_Toc204598883)

[MCF7 cells 99](#_Toc204598884)

[Supplementary Material 9. Uncropped western blot membranes in Supplementary figure 6D. 107](#_Toc204598885)

[LY2 cells 107](#_Toc204598886)

## Supplementary Material 1. Uncropped IHC images in Figure 1E.

**CDK12 protein expression evaluated by immunohistochemistry (IHC) on a tissue microarray (TMA) comprising primary tumor specimens from the RCSI cohort (n = 820).** CDK12 positivity was defined as nuclear immunoreactivity restricted to tumor epithelial cells.

**Strong CDK12 expression (H-score = 300)**

30X (50µm scale bar)


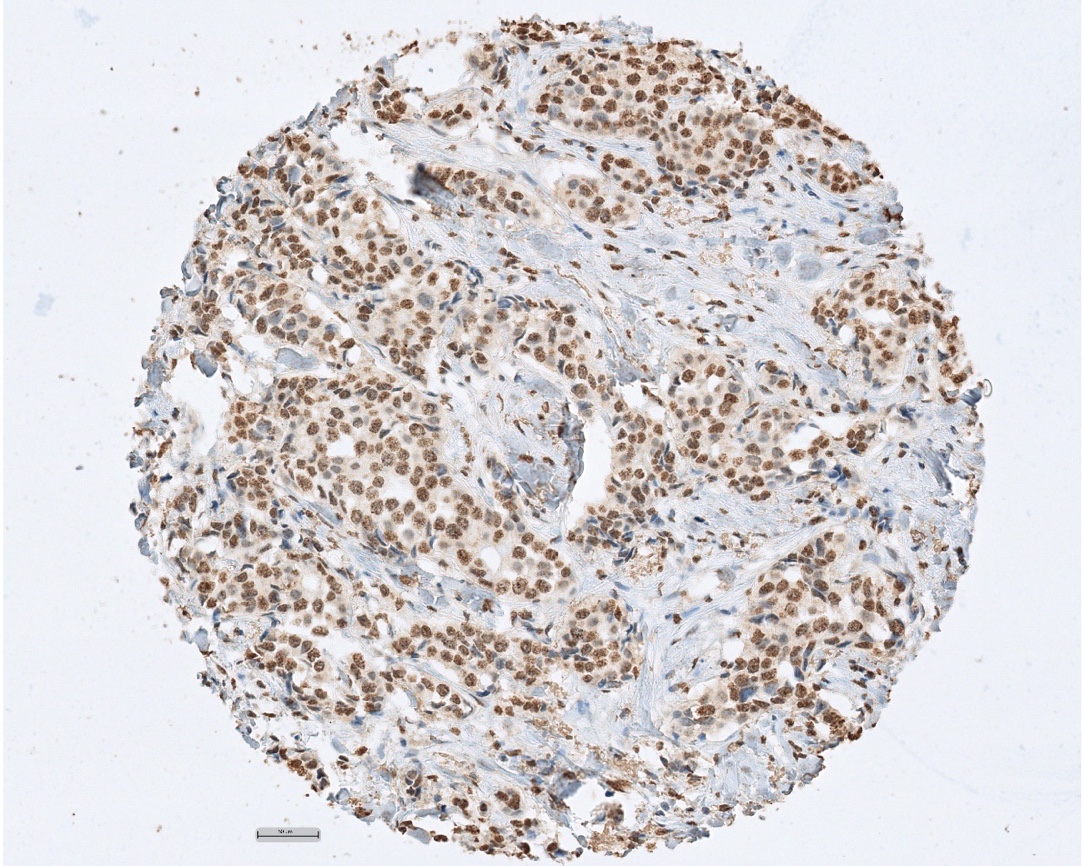


60X (20µm scale bar)


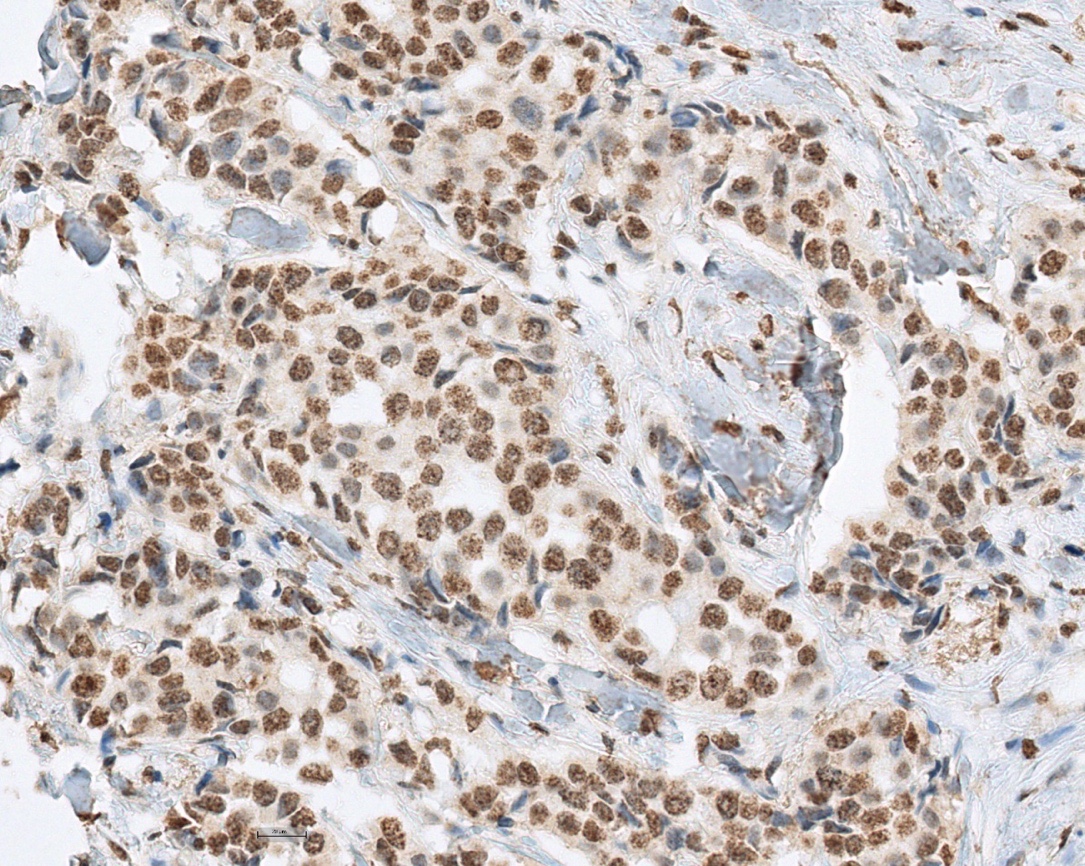


**Moderate CDK12 expression (H-score = 160)**

30X (50µm scale bar)


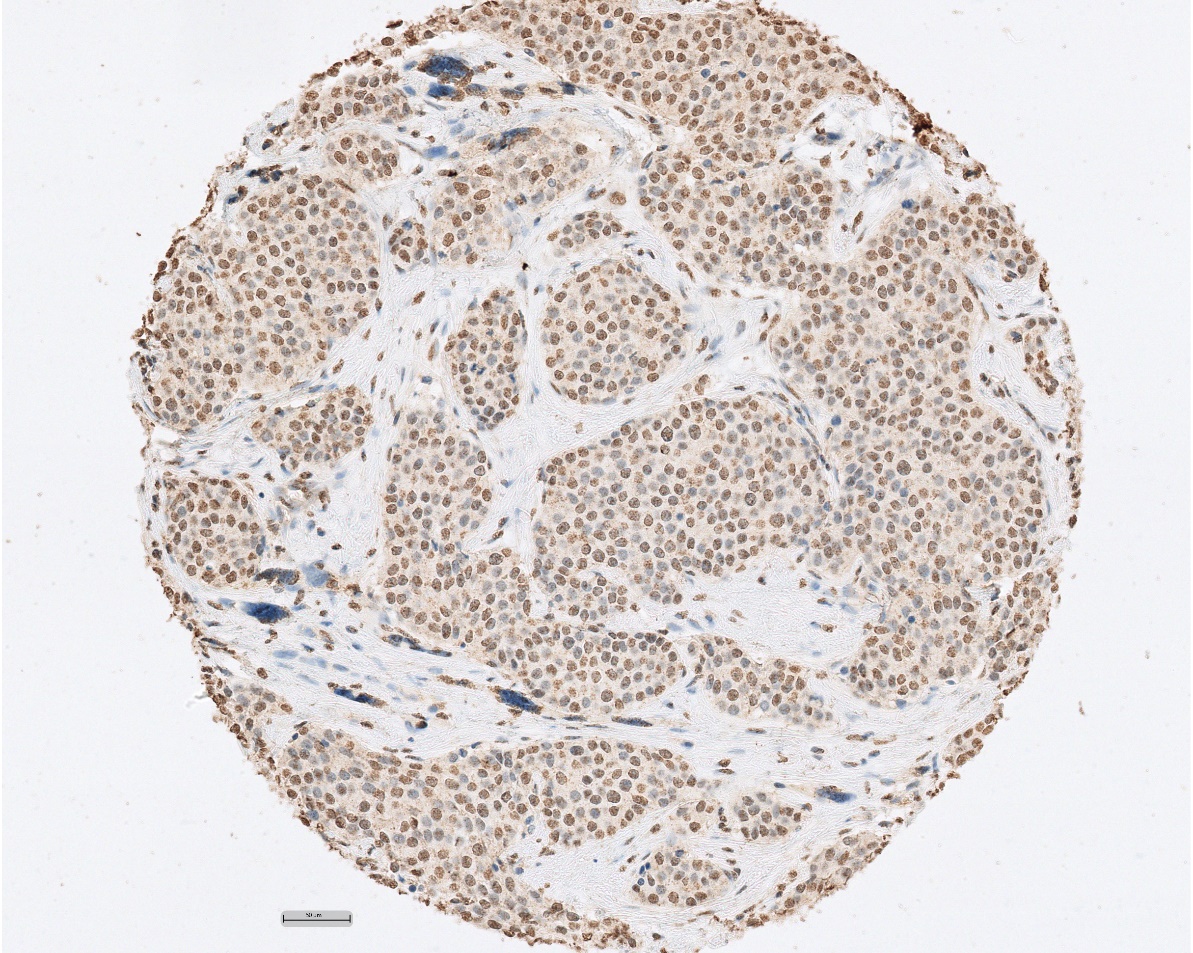


60X (20µm scale bar)


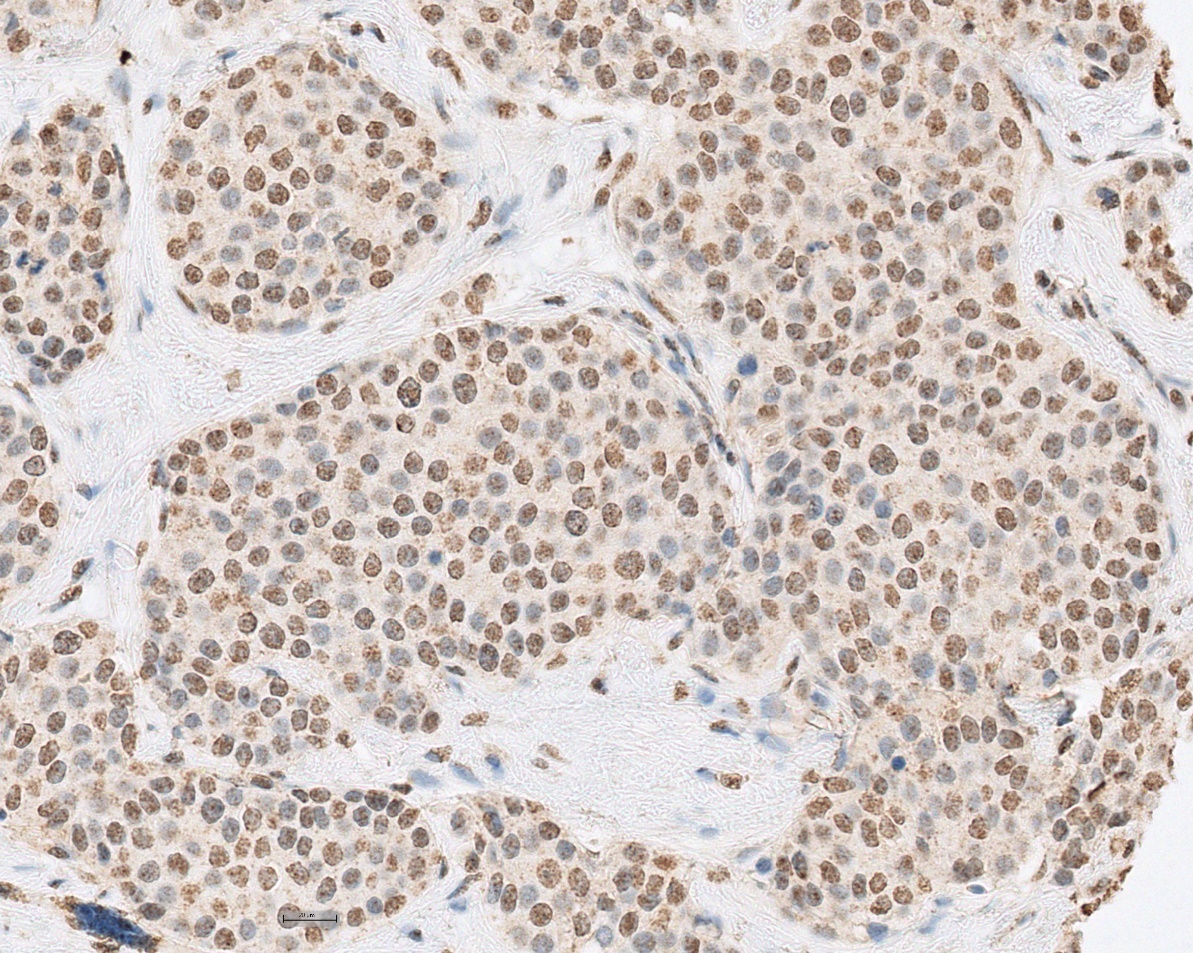


**Weak CDK12 expression (H-score = 60)**

30X (50µm scale bar)


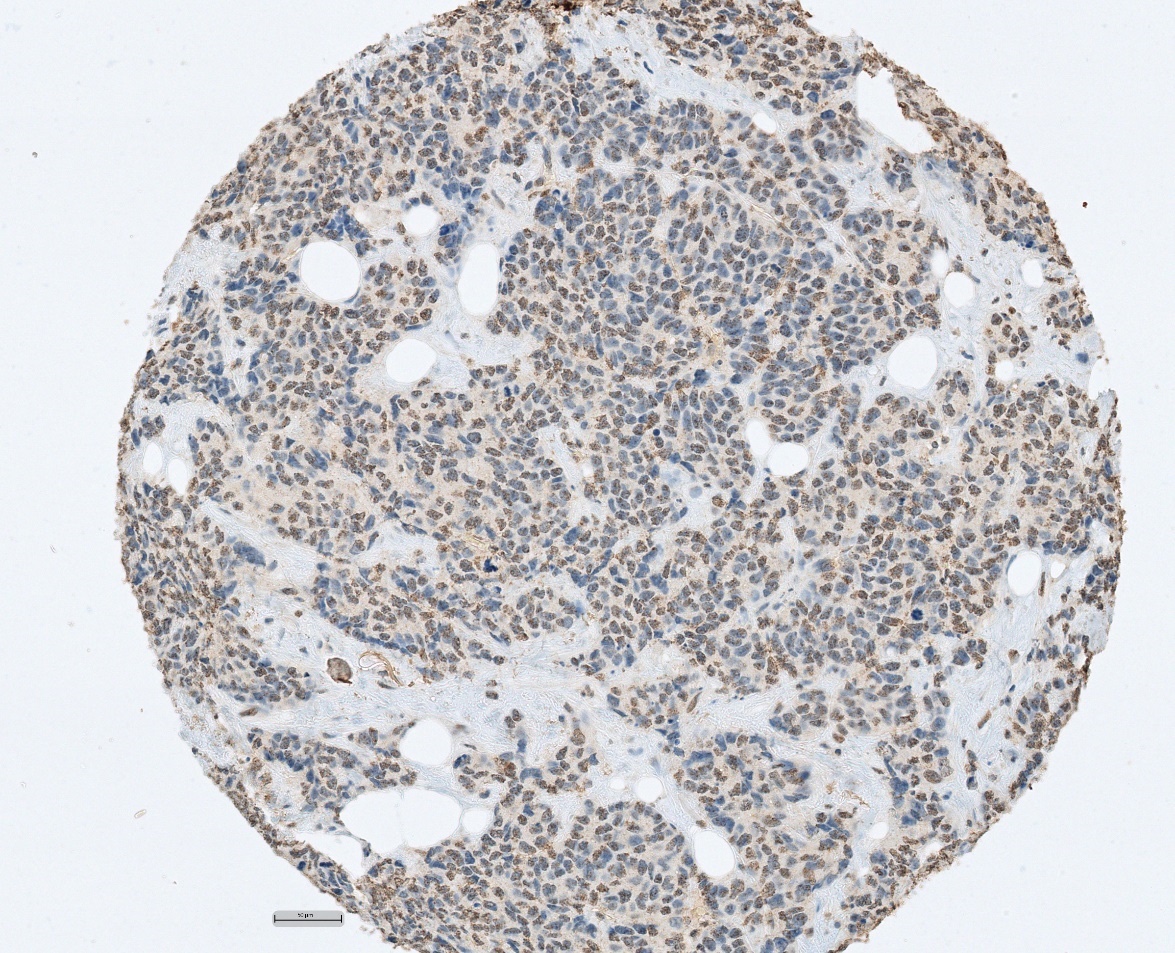


60X (20µm scale bar)

**
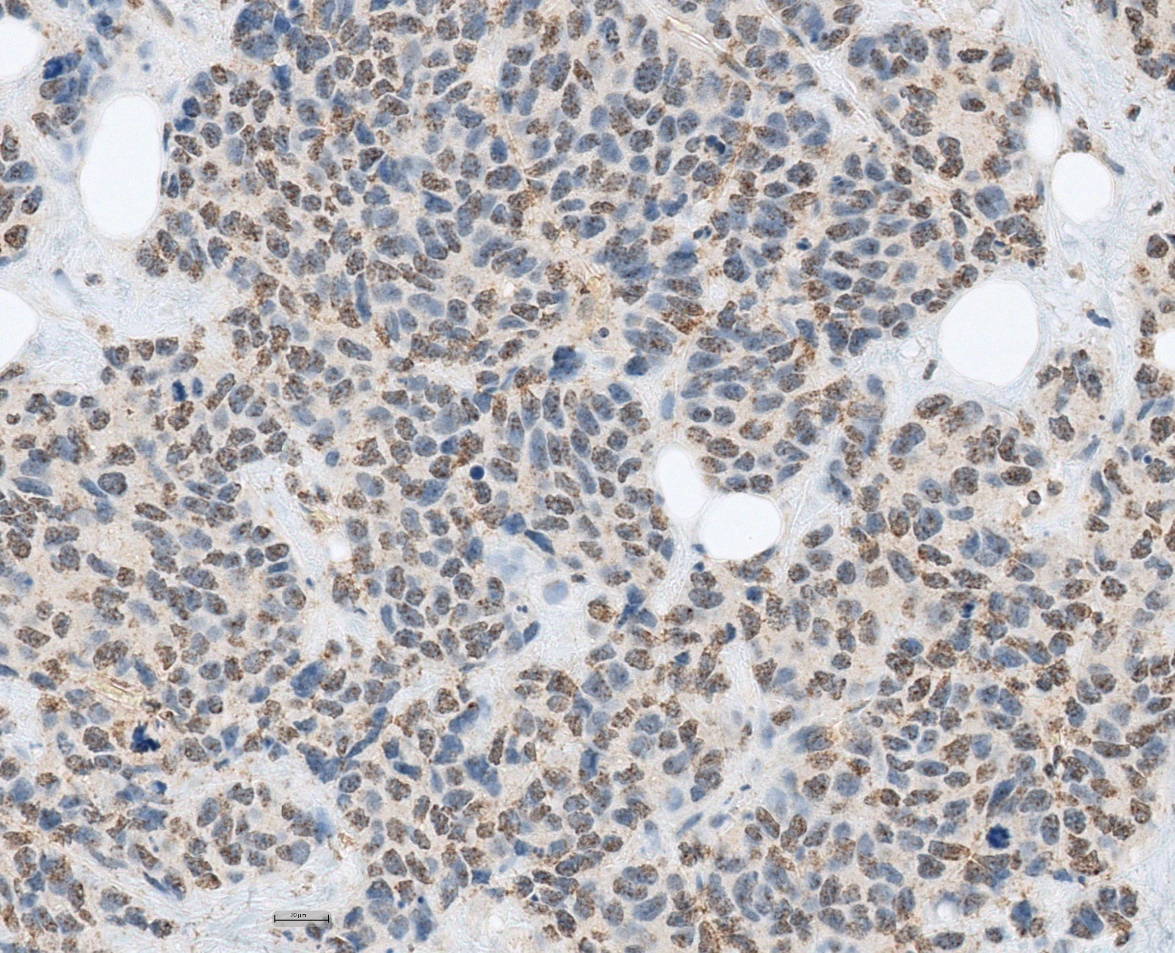
**

## Supplementary Material 2. Uncropped IHC images in Figure 3D.

**MED1 protein expression evaluated by immunohistochemistry (IHC) on a tissue microarray (TMA) comprising primary tumor specimens from the RCSI cohort (n = 807).** MED1 positivity was defined as nuclear immunoreactivity restricted to tumor epithelial cells.

**Strong MED1 expression (DAB H-score = 248)**

30X (50µm scale bar)


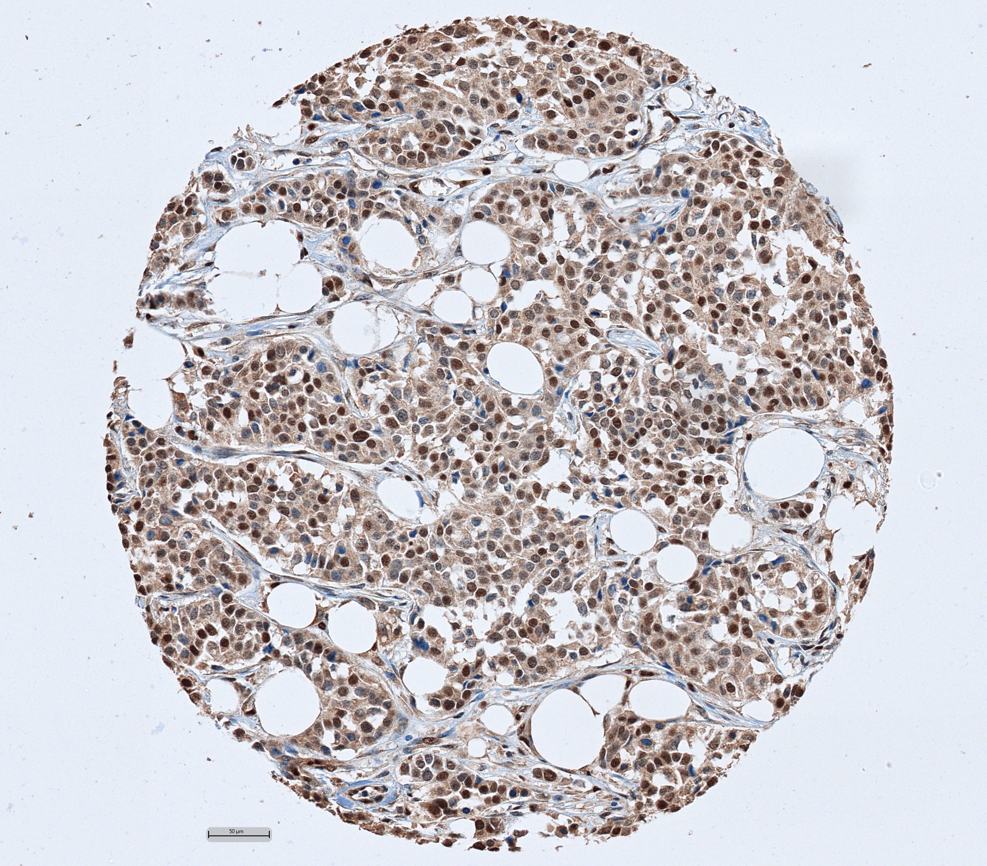


60X (20µm scale bar)


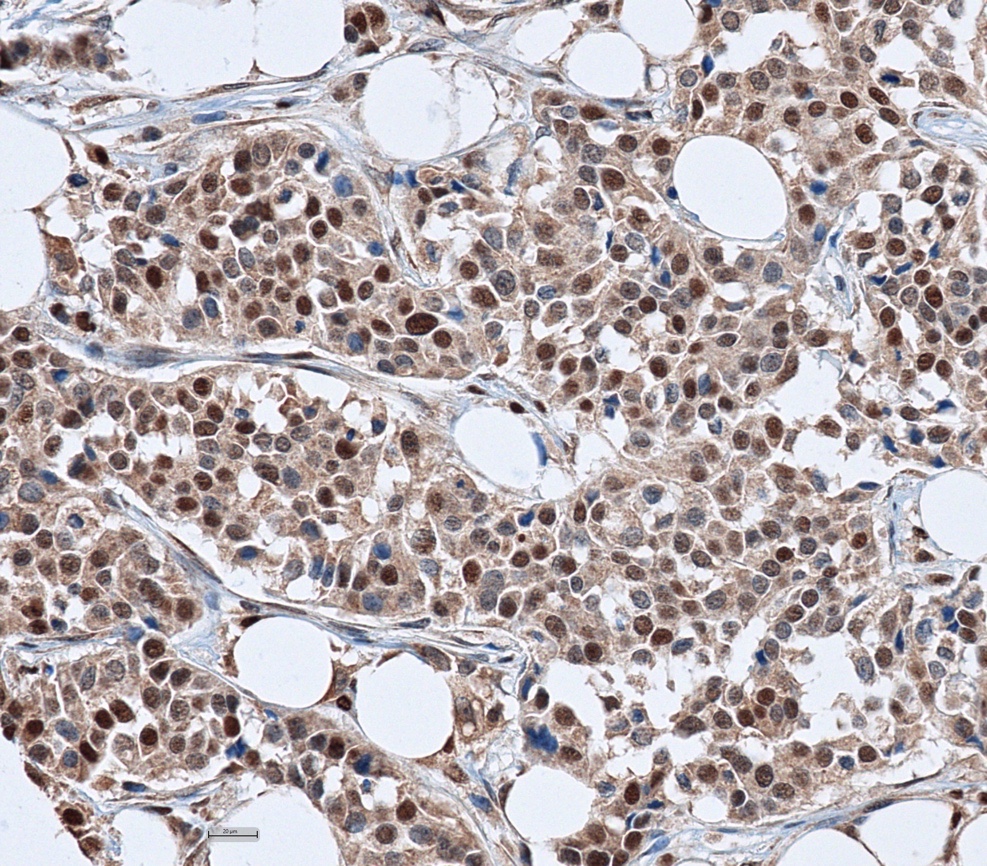


**Moderate MED1 expression (DAB H-score = 147)**

30X (50µm scale bar)


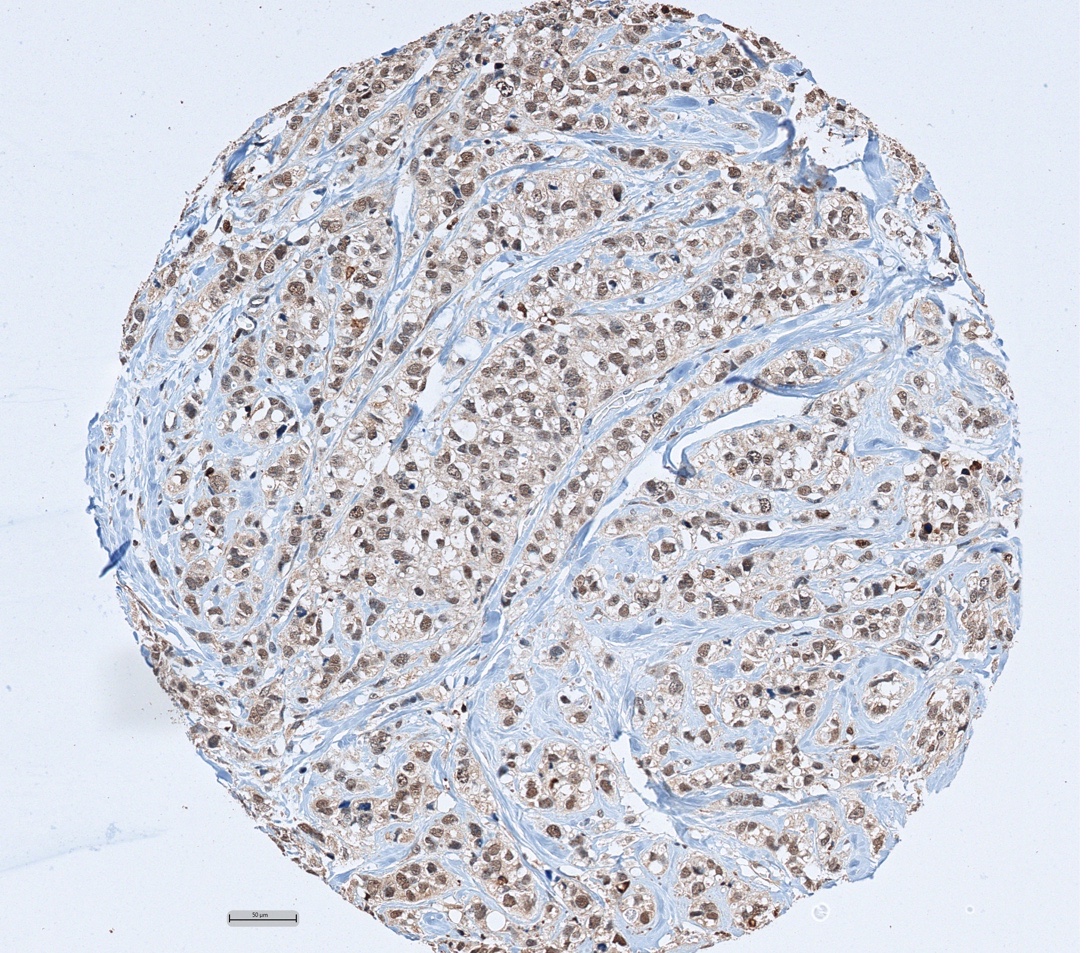


60X (20µm scale bar)


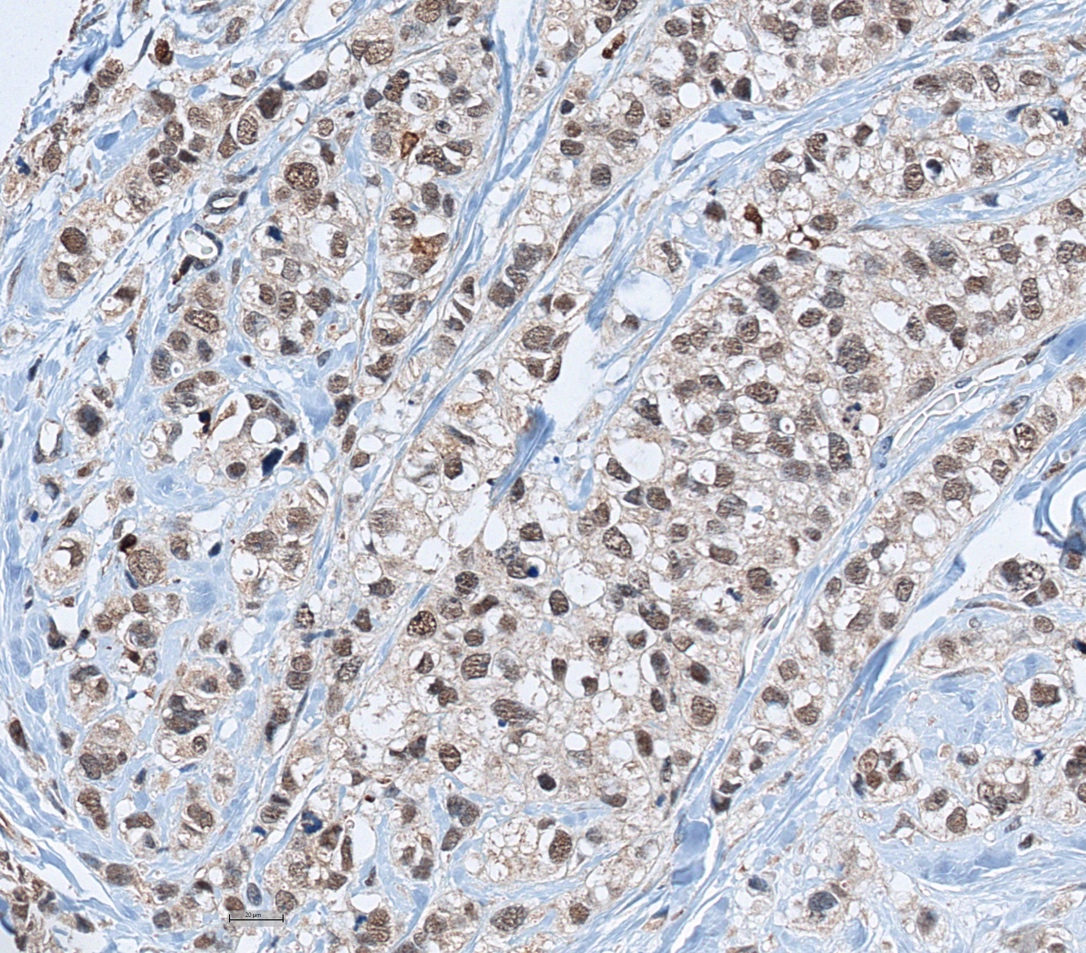


**Weak MED1 expression (DAB H-score = 53)**

30X (50µm scale bar)


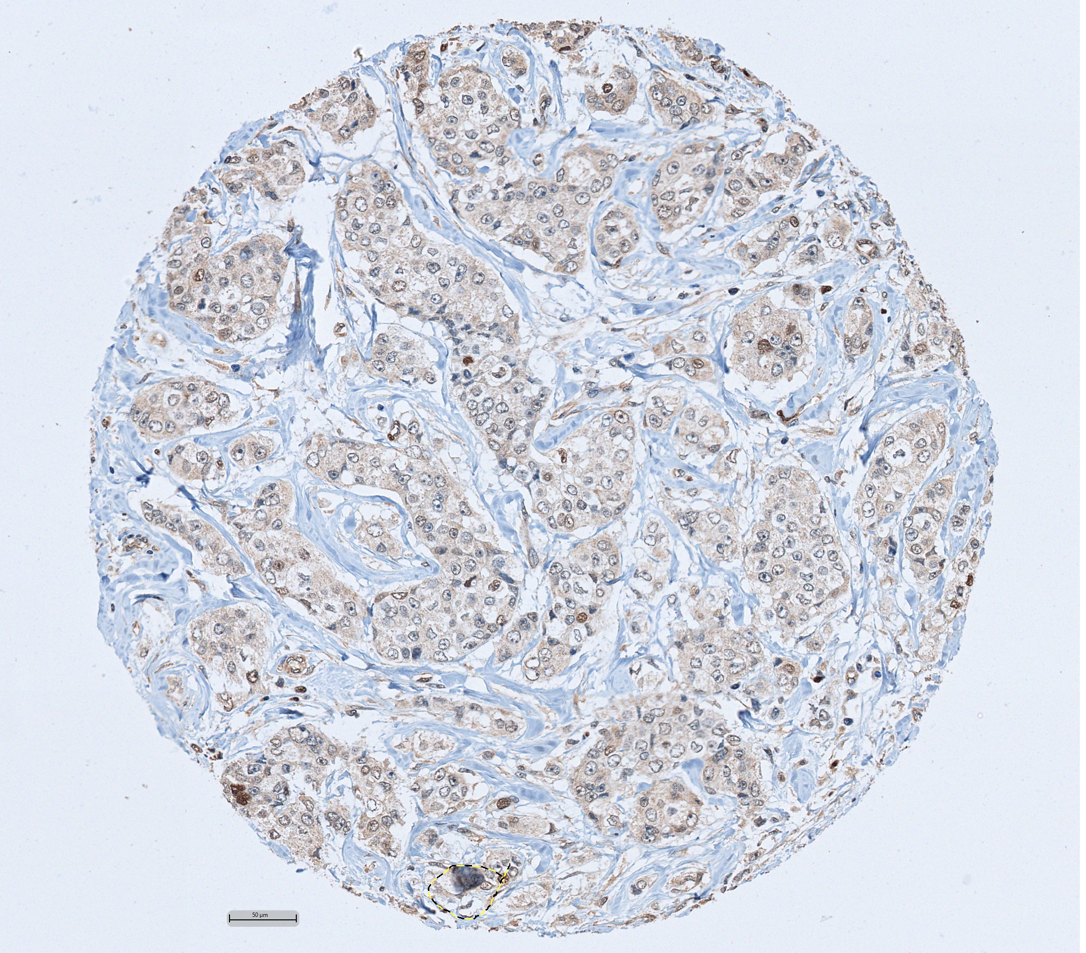


60X (20µm scale bar)


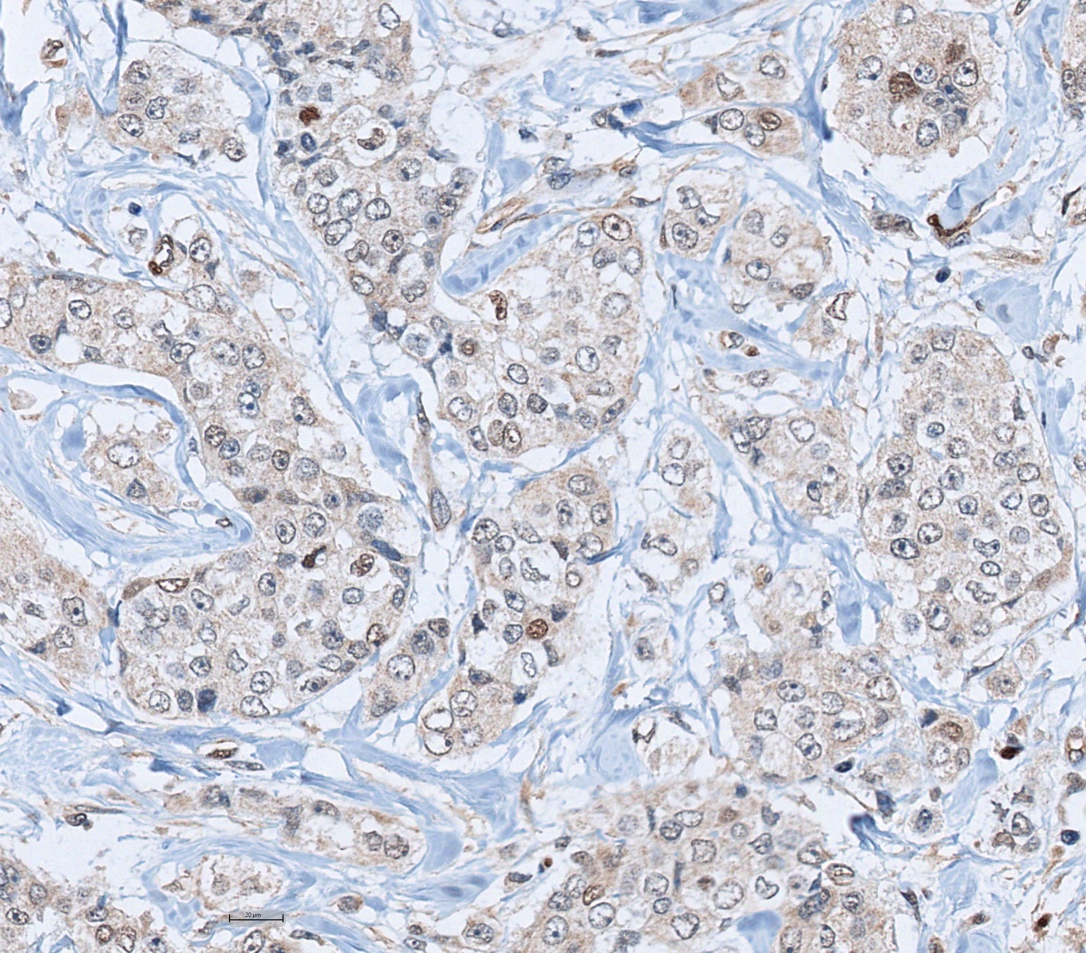


## Supplementary Material 3. Uncropped western blot membranes in Figure 4A.

**Protein expression following CDK12 knockdown (siRNA, 48 hours) in LY2 cells.** CDK12, MED1, p-MED1, ER + loading controls for cytoplasmic (GAPDH), nuclear (LMNB2) and chromatin-bound fractions (Histone 3).

### LY2 cells

#### Replicate number 1

**Ponceau staining**

|  | Replicate n1 |
| --- | --- |
| Lane n | Sample ID |
| 1 | Molecular marker (260-15 kDa) |
| 2 | LY2 siCtrl - Cyt. (n1) |
| 3 | LY2 siCtrl - Nucl. (n1) |
| 4 | LY2 siCtrl - Chrom. (n1) |
| 5 | LY2 siCDK12 - Cyt. (n1) |
| 6 | LY2 siCDK12 - Nucl. (n1) |
| 7 | LY2 siCDK12 - Chrom. (n1) |


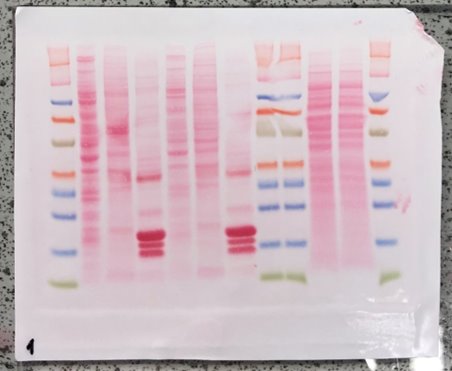


1 2 3 4 5 6 7

**CDK12 205kDa**


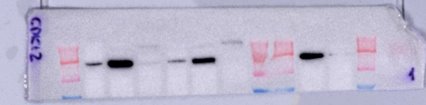


2 3 4 5 6 7

**MED1 220kDa**


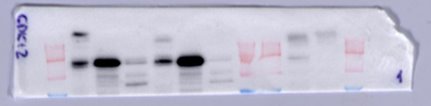


2 3 4 5 6 7

**pMED1 ~240kDa**


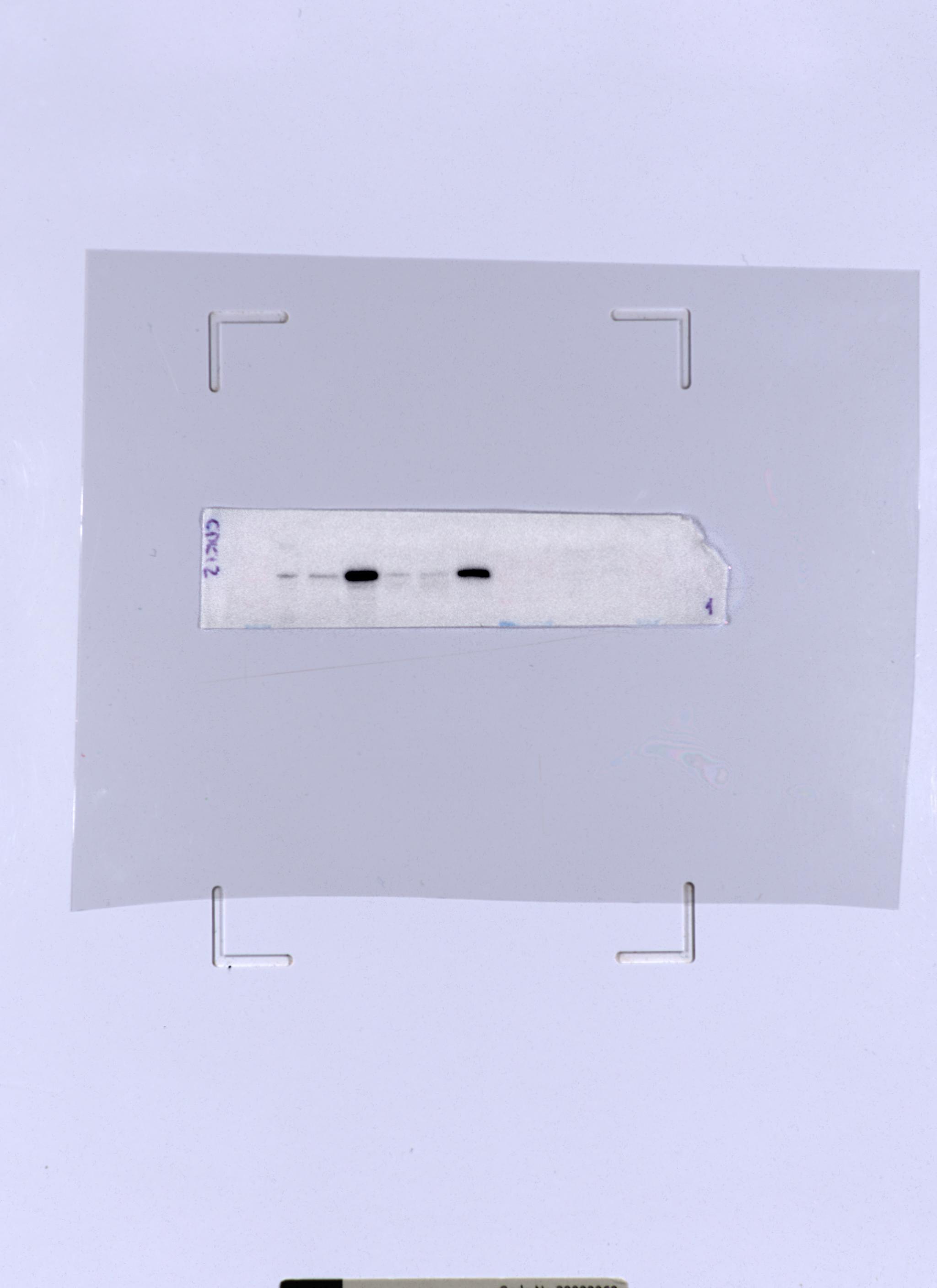


2 3 4 5 6 7

**ER 60kDa**

2 3 4 5 6 7


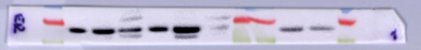


**LMNB2 68kDa**


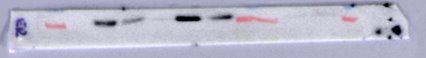


2 3 4 5 6 7

**GAPDH 38kDa**


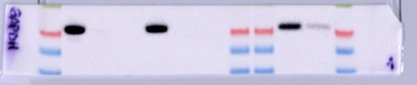


2 3 4 5 6 7

**Histone 3 17kDa**


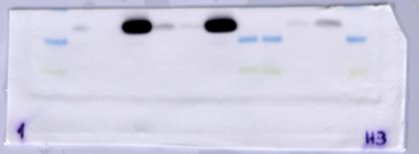


2 3 4 5 6 7

#### Replicate number 2 (lanes 2 to 7) + Replicate number 3 (lanes 9 to 14, shown in Figure 4A)

Membrane I (Immunoblotting: CDK12, pMED1, ER, LMNB2, GAPDH, Histone 3).

**Ponceau staining**

|  | Replicate n2 and n3 |
| --- | --- |
| Lane n | Sample ID |
| 1 | Molecular marker (260-15 kDa) |
| 2 | LY2 siCtrl - Cyt. (n2) |
| 3 | LY2 siCtrl - Nucl. (n2) |
| 4 | LY2 siCtrl - Chrom. (n2) |
| 5 | LY2 siCDK12 - Cyt. (n2) |
| 6 | LY2 siCDK12 - Nucl. (n2) |
| 7 | LY2 siCDK12 - Chrom. (n2) |
| 8 | Molecular marker (260-15 kDa) |
| 9 | LY2 siCtrl - Cyt. (n3) |
| 10 | LY2 siCtrl - Nucl. (n3) |
| 11 | LY2 siCtrl - Chrom. (n3) |
| 12 | LY2 siCDK12 - Cyt. (n3) |
| 13 | LY2 siCDK12 - Nucl. (n3) |
| 14 | LY2 siCDK12 - Chrom. (n3) |
| 15 | Molecular marker (260-15 kDa) |


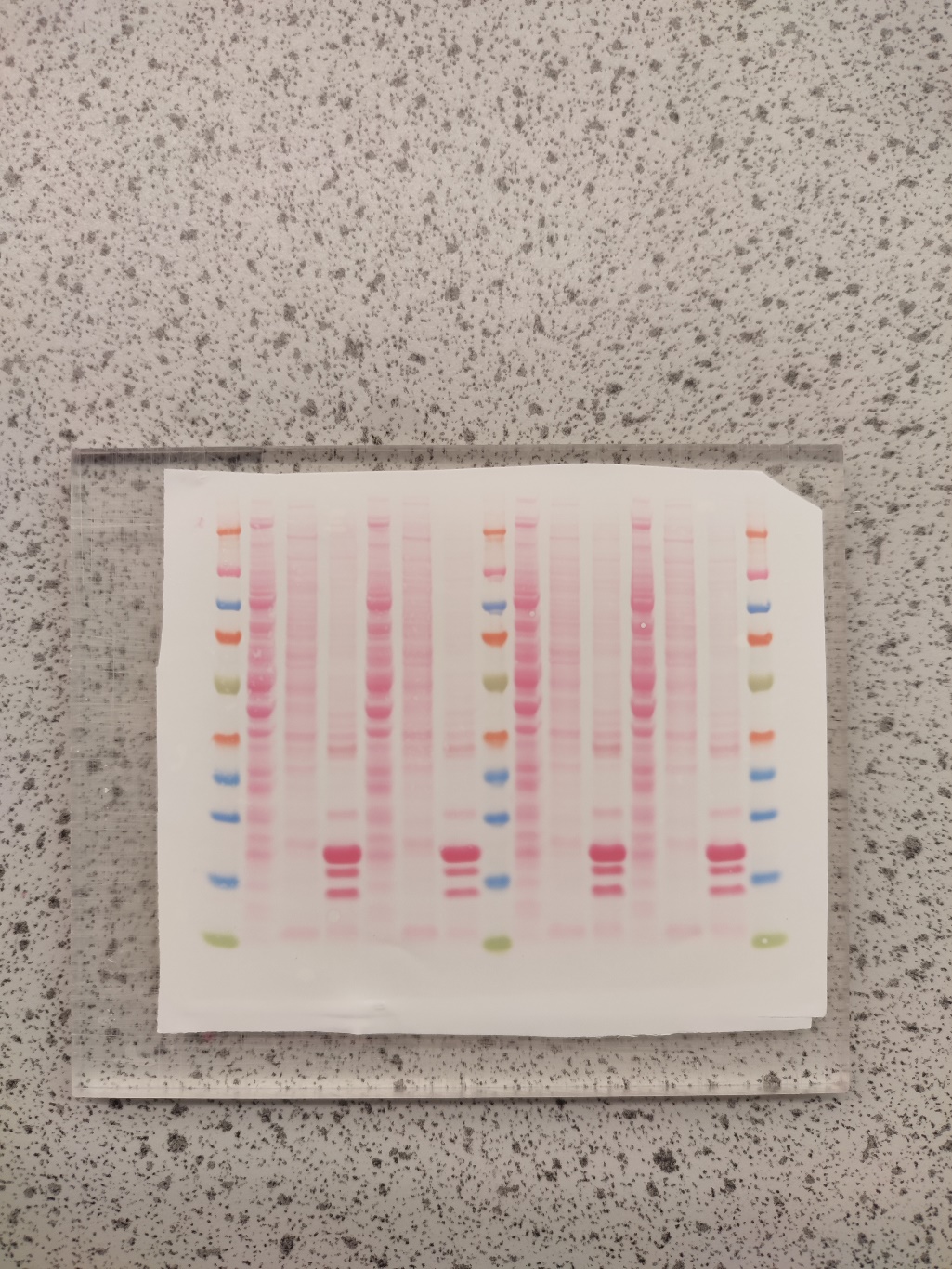


1 2 3 4 5 6 7 8 9 10 11 12 13 14 15

**CDK12 205kDa**


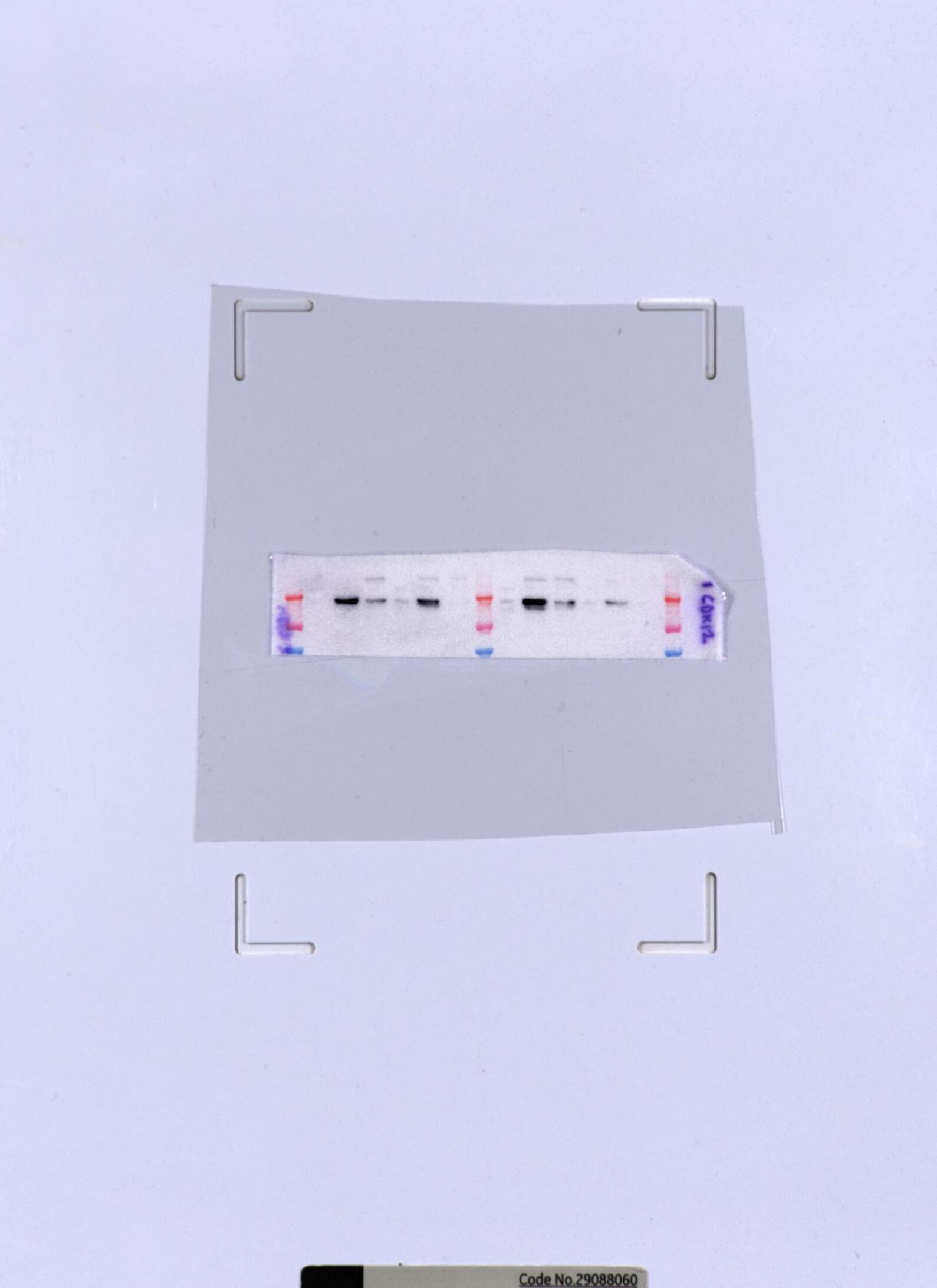


2 3 4 5 6 7 9 10 11 12 13 14

**pMED1 ~240kDa**


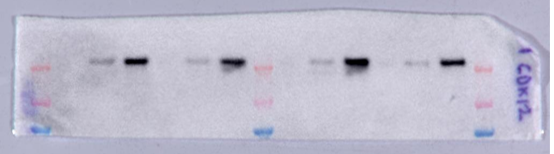


2 3 4 5 6 7 9 10 11 12 13 14

**ER 60kDa**


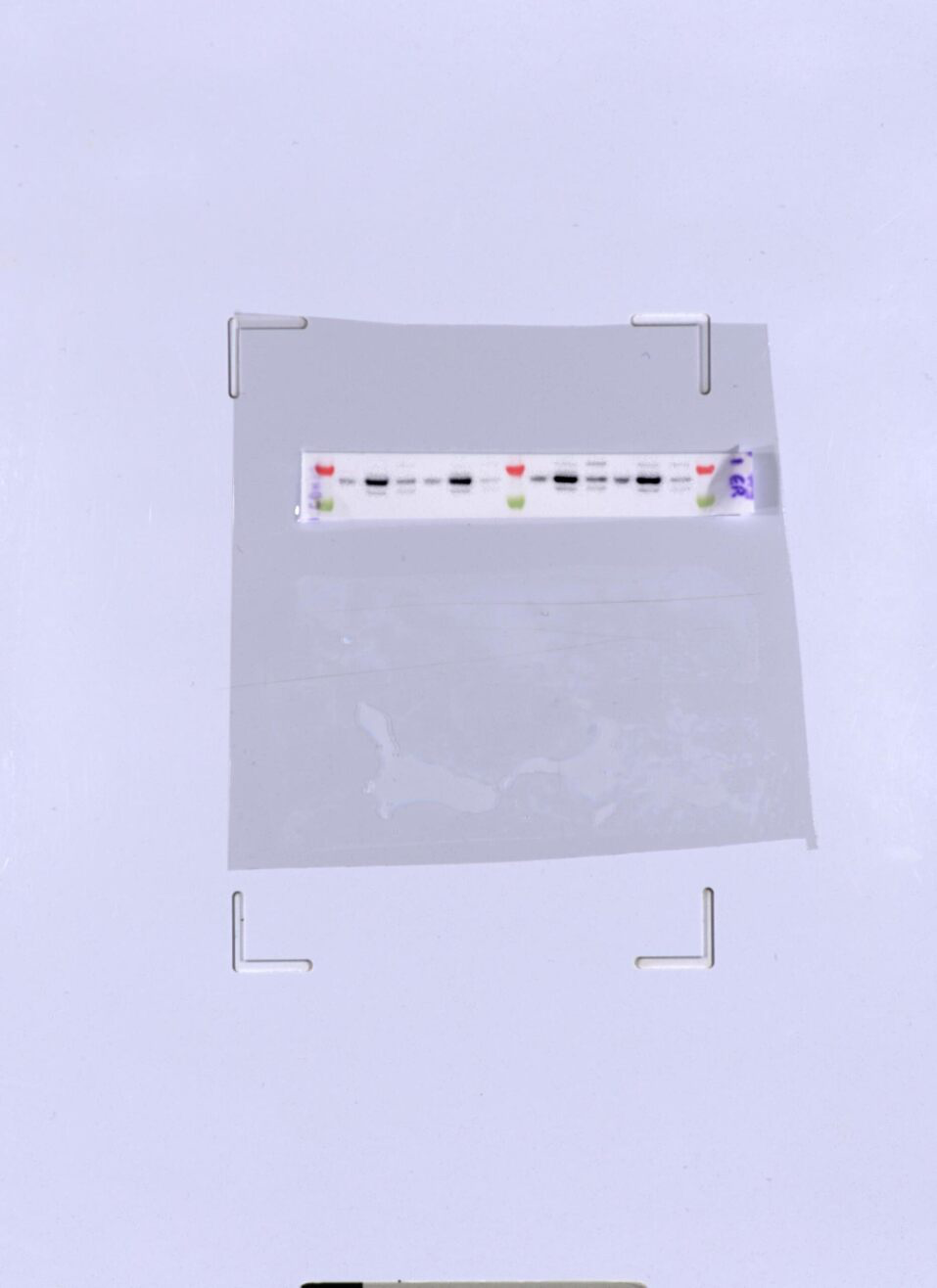


2 3 4 5 6 7 9 10 11 12 13 14

**LMNB2 68 kDa**


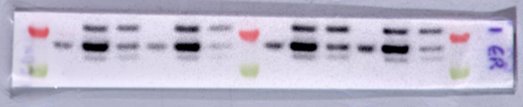


2 3 4 5 6 7 9 10 11 12 13 14

**GAPDH 38kDa**


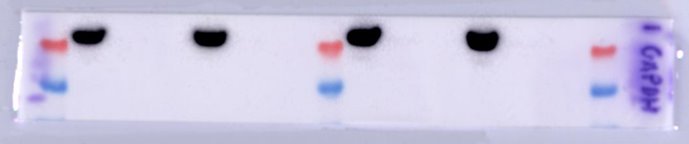


2 3 4 5 6 7 9 10 11 12 13 14

**Histone 3 17kDa**


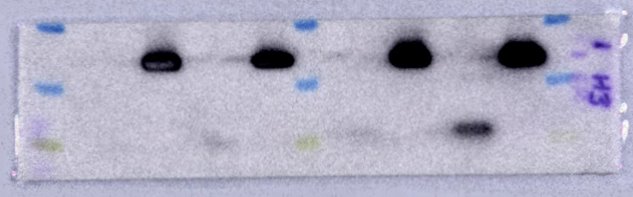


2 3 4 5 6 7 9 10 11 12 13 14

Membrane II (Immunoblotting: MED1, LMNB2, GAPDH, Histone 3)

**Ponceau staining**

|  | Replicate n2 and n3 |
| --- | --- |
| Lane n | Sample ID |
| 1 | Molecular marker (260-15 kDa) |
| 2 | LY2 siCtrl - Cyt. (n2) |
| 3 | LY2 siCtrl - Nucl. (n2) |
| 4 | LY2 siCtrl - Chrom. (n2) |
| 5 | LY2 siCDK12 - Cyt. (n2) |
| 6 | LY2 siCDK12 - Nucl. (n2) |
| 7 | LY2 siCDK12 - Chrom. (n2) |
| 8 | Molecular marker (260-15 kDa) |
| 9 | LY2 siCtrl - Cyt. (n3) |
| 10 | LY2 siCtrl - Nucl. (n3) |
| 11 | LY2 siCtrl - Chrom. (n3) |
| 12 | LY2 siCDK12 - Cyt. (n3) |
| 13 | LY2 siCDK12 - Nucl. (n3) |
| 14 | LY2 siCDK12 - Chrom. (n3) |
| 15 | Molecular marker (260-15 kDa) |


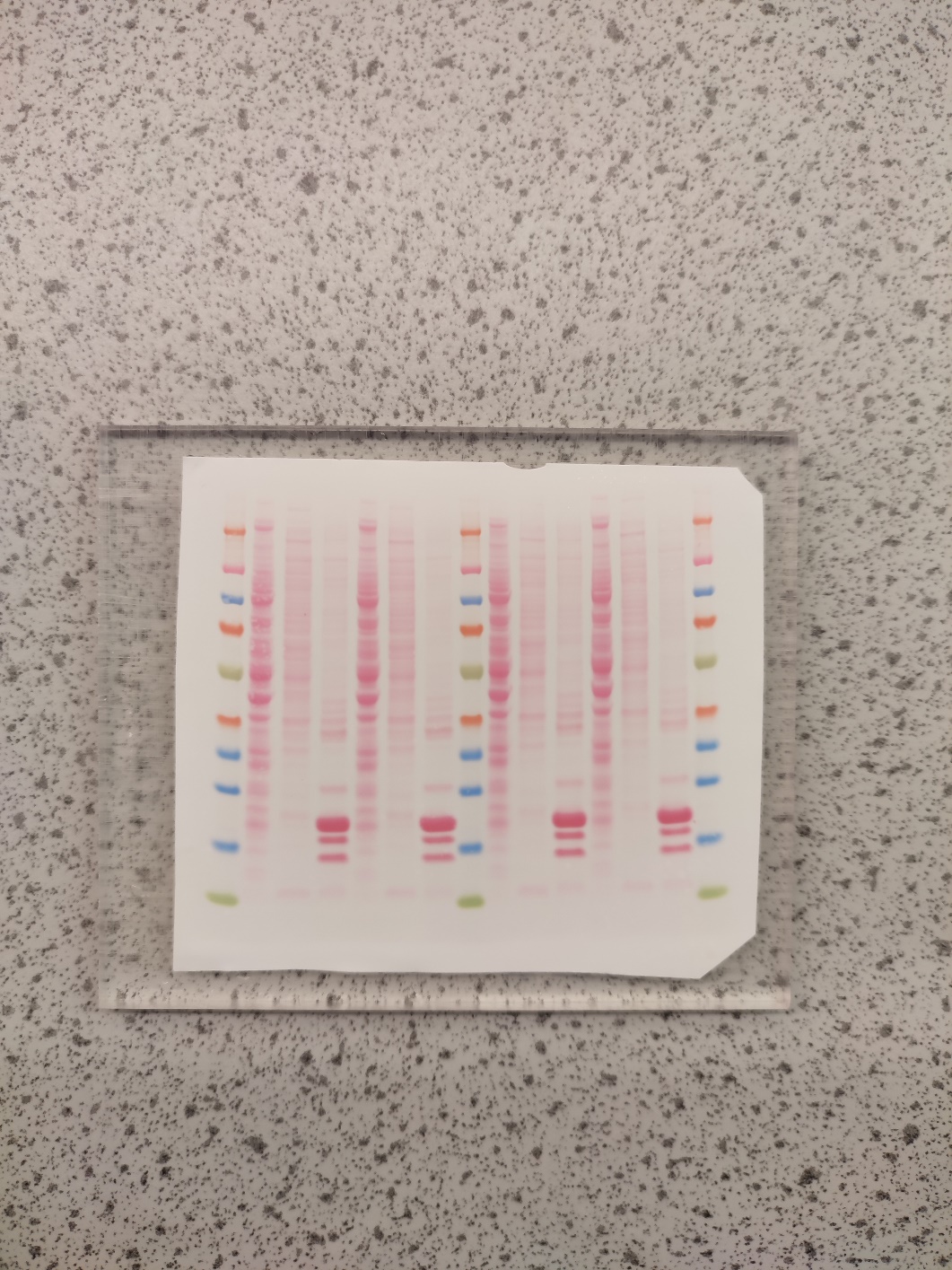


1 2 3 4 5 6 7 8 9 10 11 12 13 14 15

**MED1 220kDa**


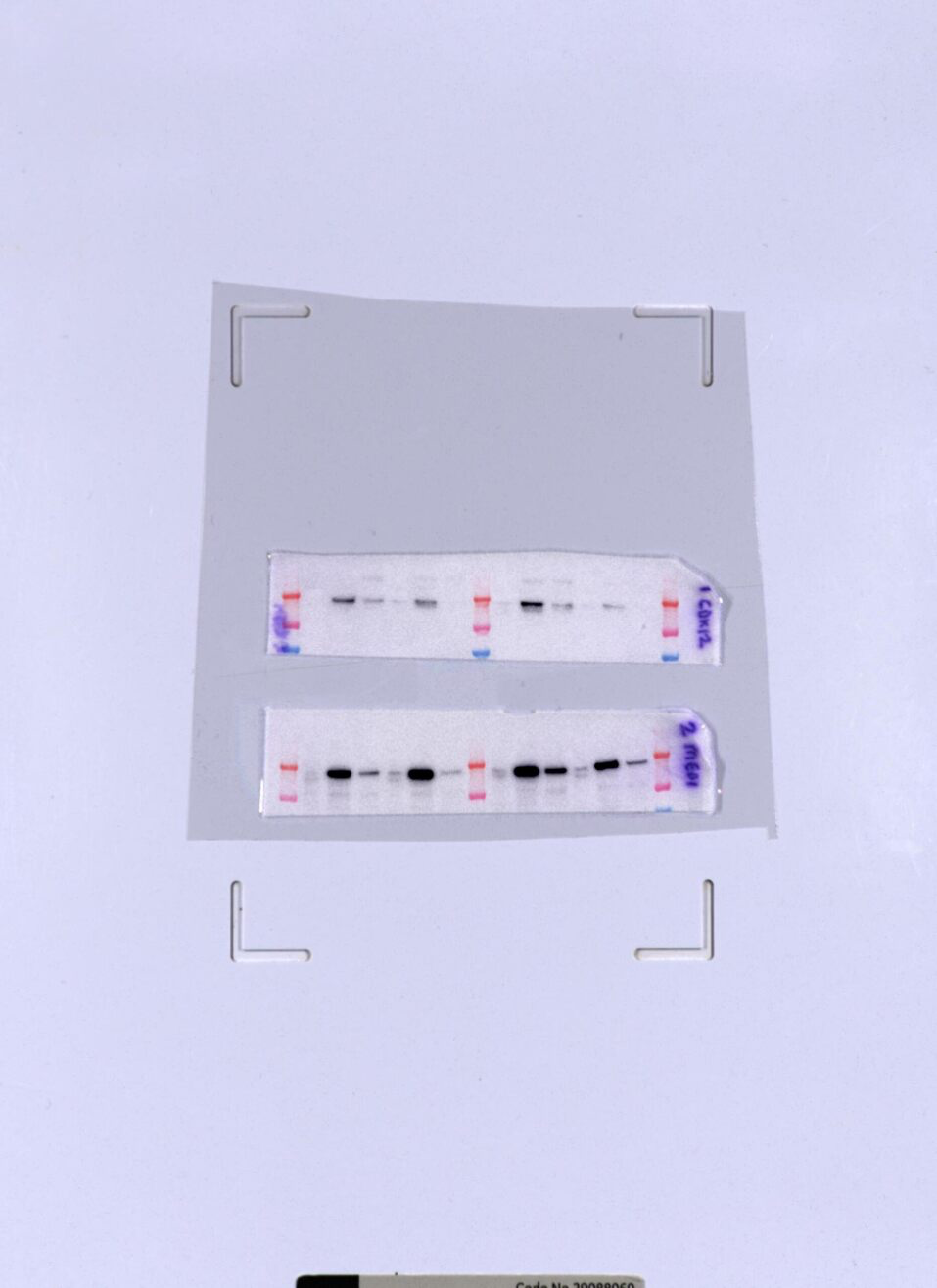


2 3 4 5 6 7 9 10 11 12 13 14

**LMNB2 68kDa**


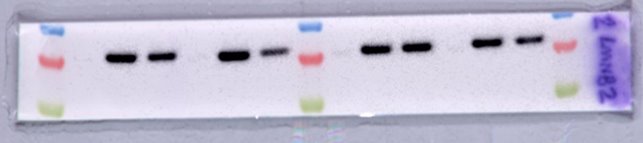


2 3 4 5 6 7 9 10 11 12 13 14

**GAPDH 38kDa**


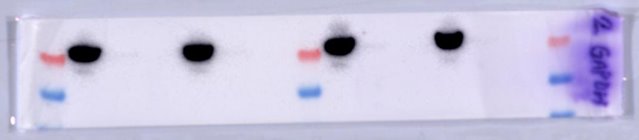


2 3 4 5 6 7 9 10 11 12 13 14

**Histone 3 17kDa**


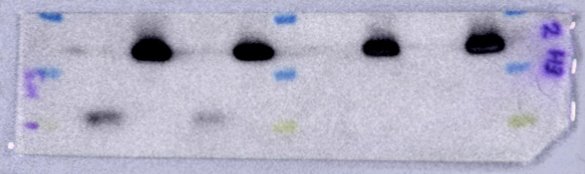


2 3 4 5 6 7 9 10 11 12 13 14

## Supplementary Material 4. Uncropped western blot membranes in Figure 4B.

**Co-immunoprecipitation experiments following CDK12 knockdown (siRNA, 48 hours) in LY2 cells.** MED1, pMED1, and ER immunoblotting.

### LY2 - IP ER

#### Replicate number 1

**Ponceau staining**

|  | Replicate n1 |
| --- | --- |
| Lane n | Sample ID |
| 1 | Molecular marker (260-15 kDa) |
| 2 | LY2 siCtrl – input |
| 3 | LY2 siCtrl – IP ER |
| 4 | LY2 siCtrl – IP IgG |
| 5 | LY2 siCDK12 – input |
| 6 | LY2 siCDK12 – IP ER |
| 7 | LY2 siCDK12 – IP igG |
| 8 | Molecular marker (260-15 kDa) |
| 9 | Molecular marker (260-15 kDa) |
| 10 | LY2 siCtrl – input |
| 11 | LY2 siCtrl – IP ER |
| 12 | LY2 siCtrl – IP IgG |
| 13 | LY2 siCDK12 – input |
| 14 | LY2 siCDK12 – IP ER |
| 15 | LY2 siCDK12 – IP igG |


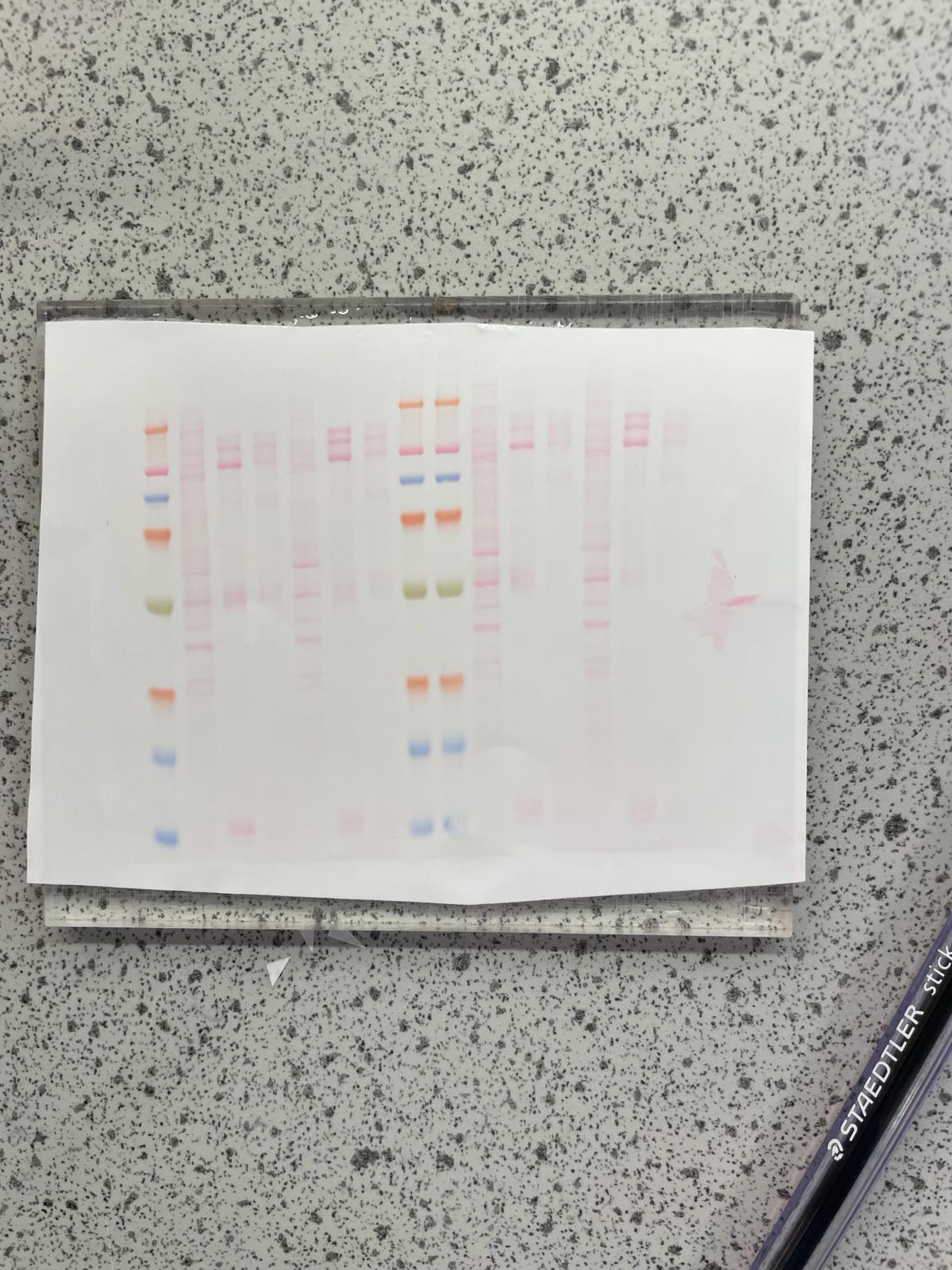


9 10 11 12 13 14 15

1 2 3 4 5 6 7 8

**MED1 220kDa**


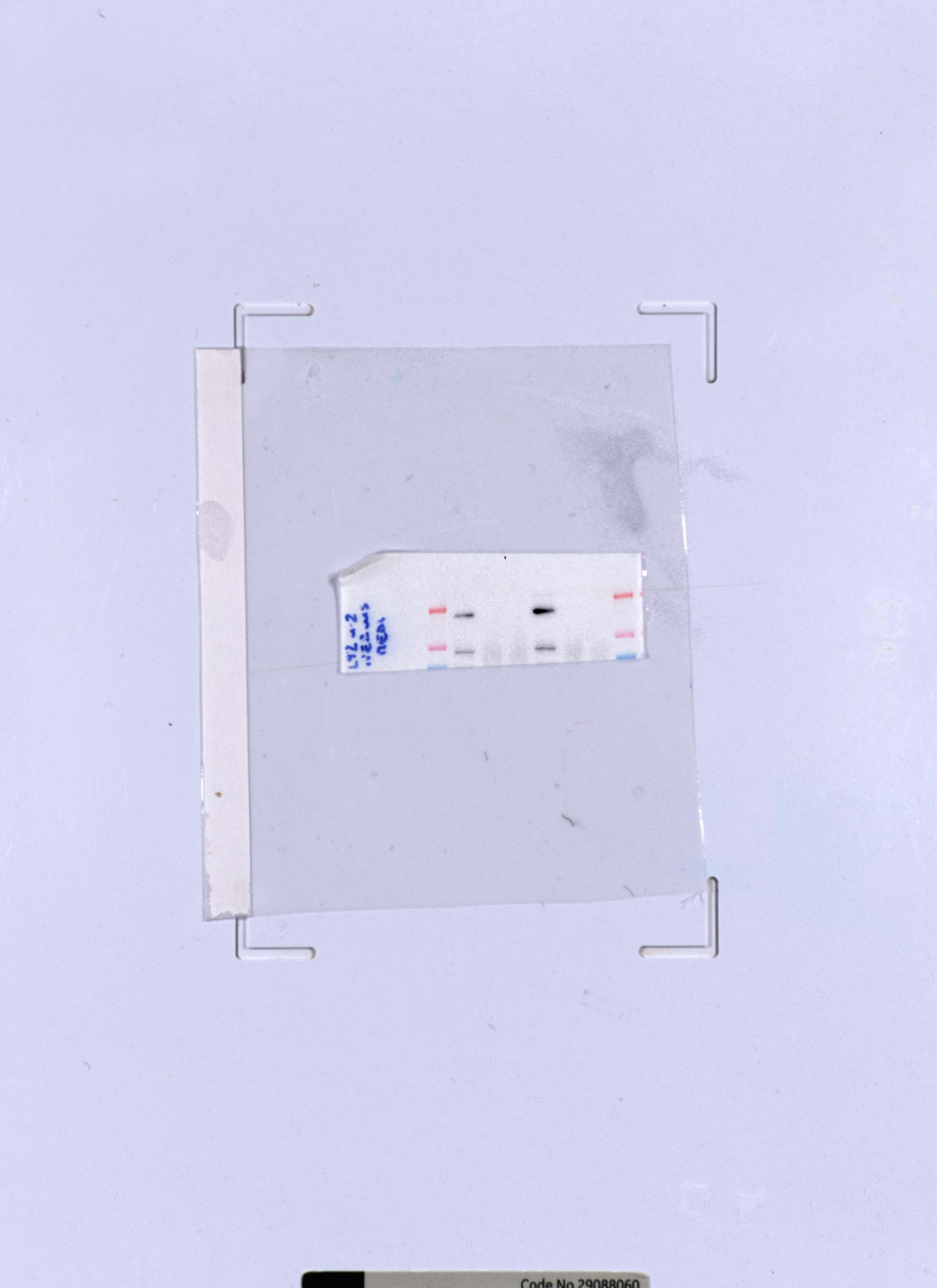


1 2 3 4 5 6 7 8

**pMED1 ~240kDa**

9 10 11 12 13 14 15


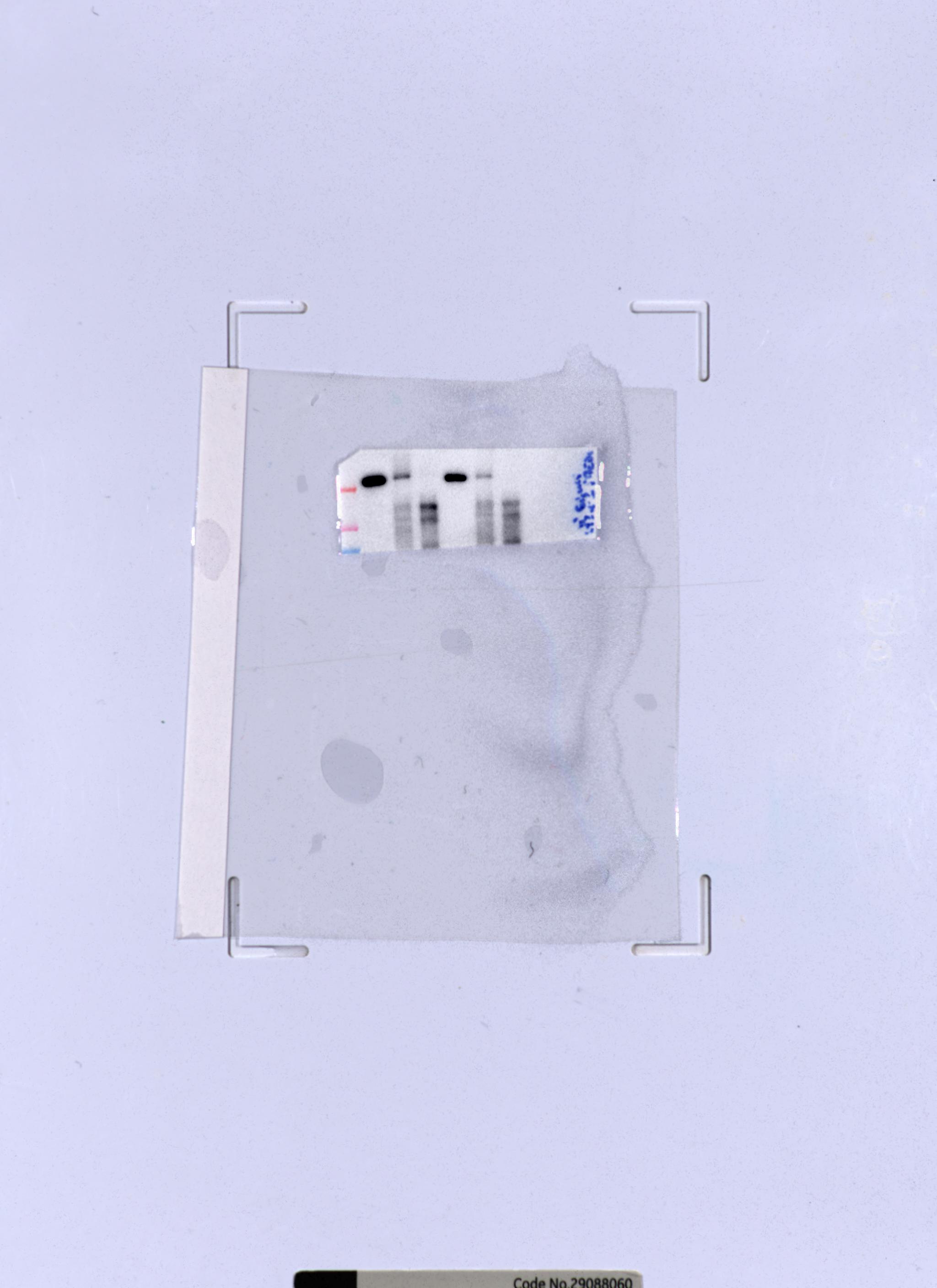


**ER 69kDa**

1 2 3 4 5 6 7 8


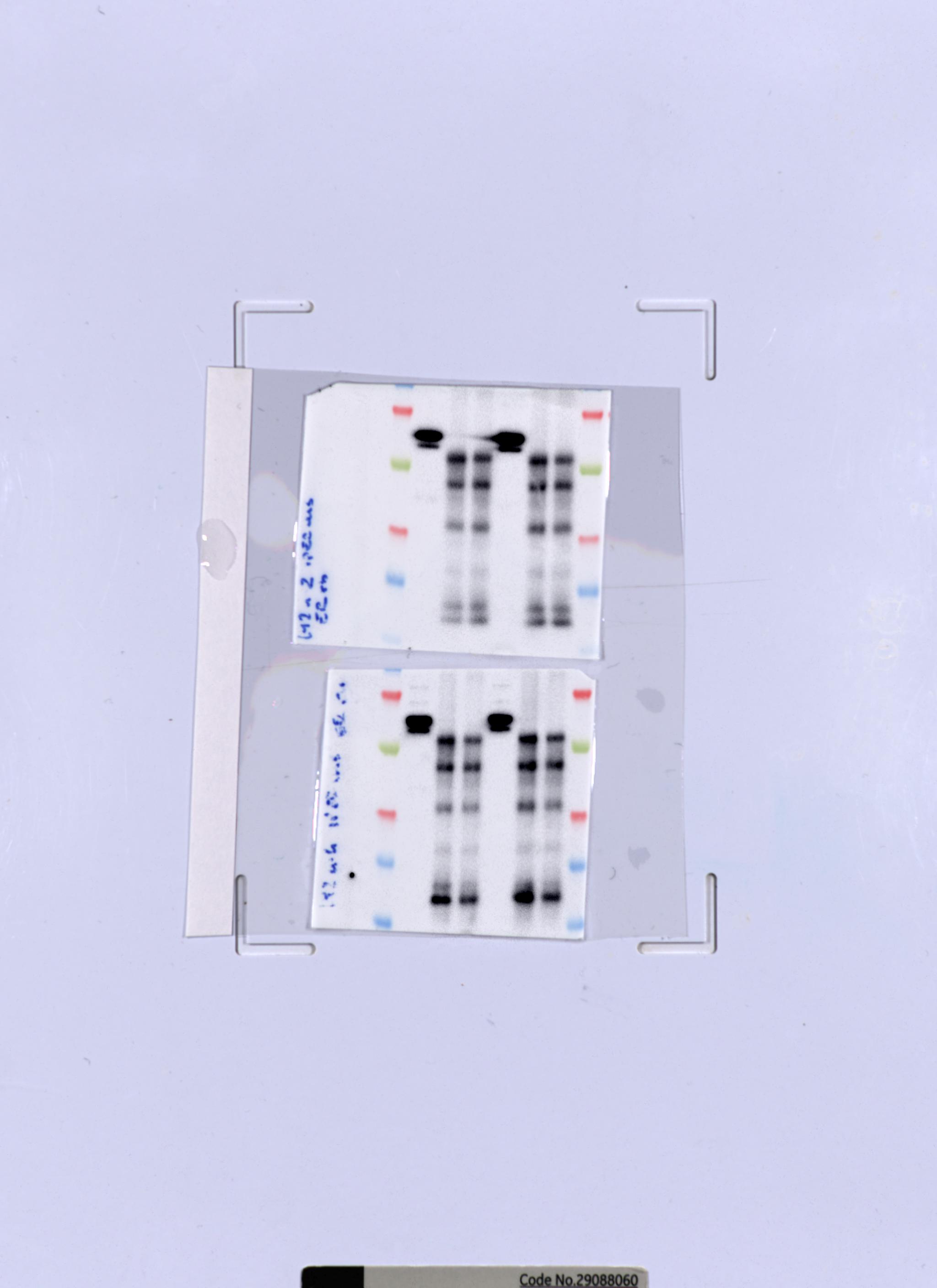


#### Replicate number 2

**Ponceau staining**

|  | Replicate n2 |
| --- | --- |
| Lane n | Sample ID |
| 1 | Molecular marker (260-15 kDa) |
| 2 | LY2 siCtrl – input |
| 3 | LY2 siCtrl – IP ER |
| 4 | LY2 siCtrl – IP IgG |
| 5 | LY2 siCDK12 – input |
| 6 | LY2 siCDK12 – IP ER |
| 7 | LY2 siCDK12 – IP igG |
| 8 | Molecular marker (260-15 kDa) |
| 9 | Molecular marker (260-15 kDa) |
| 10 | LY2 siCtrl – input |
| 11 | LY2 siCtrl – IP ER |
| 12 | LY2 siCtrl – IP IgG |
| 13 | LY2 siCDK12 – input |
| 14 | LY2 siCDK12 – IP ER |
| 15 | LY2 siCDK12 – IP igG |


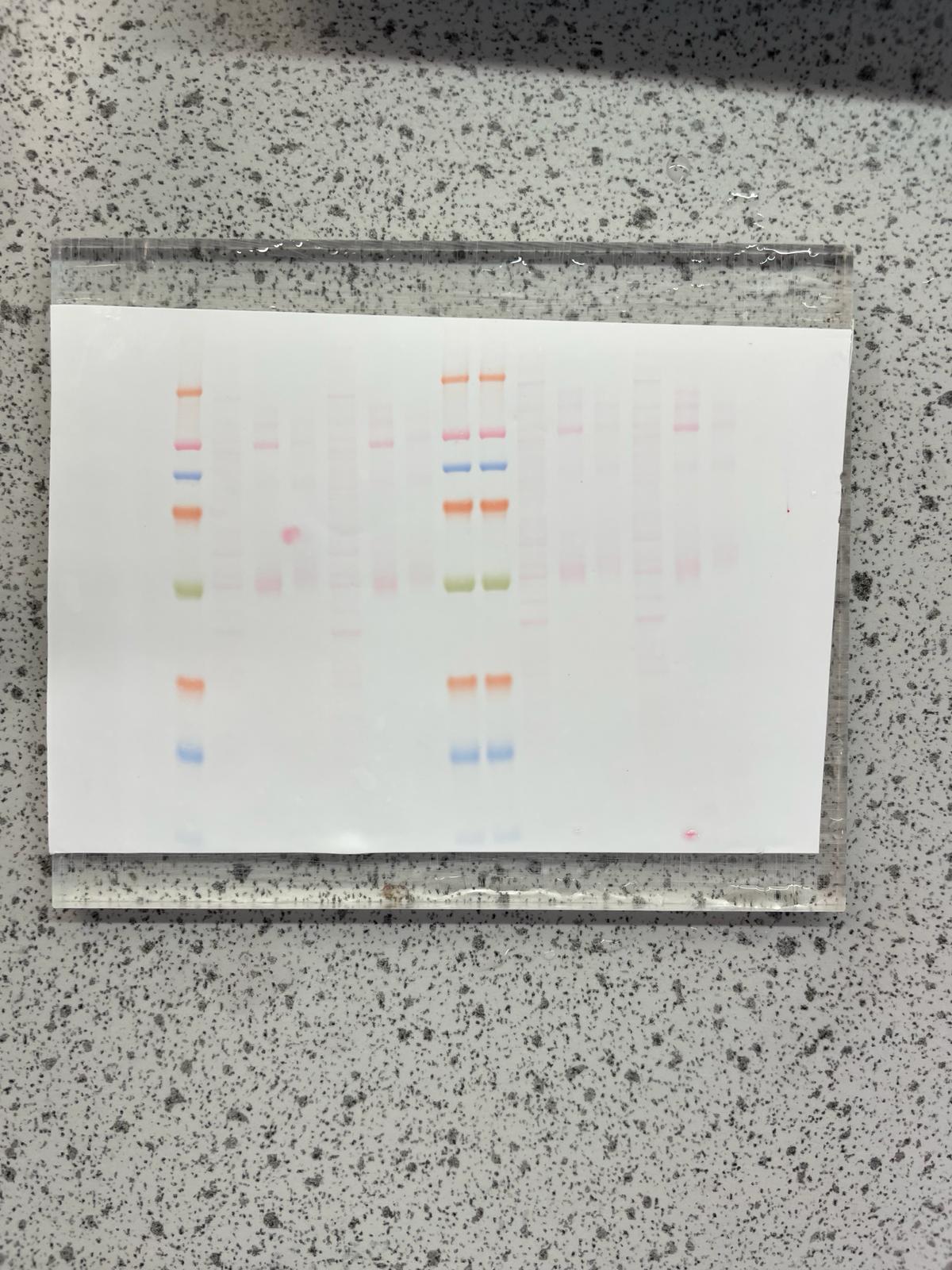


9 10 11 12 13 14 15

1 2 3 4 5 6 7 8

**MED1 220kDa**


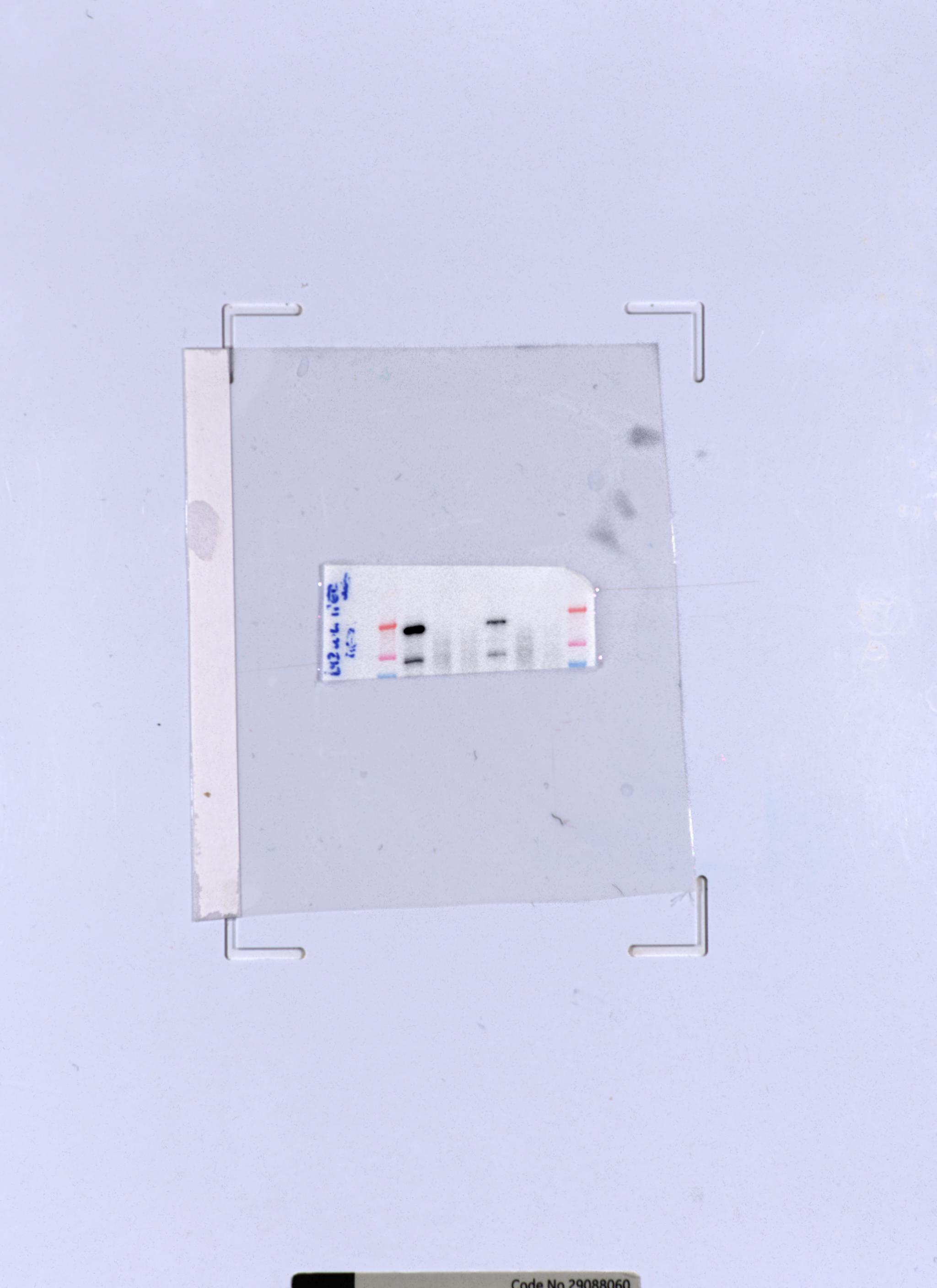


1 2 3 4 5 6 7 8

**pMED1 ~240kDa**

9 10 11 12 13 14 15


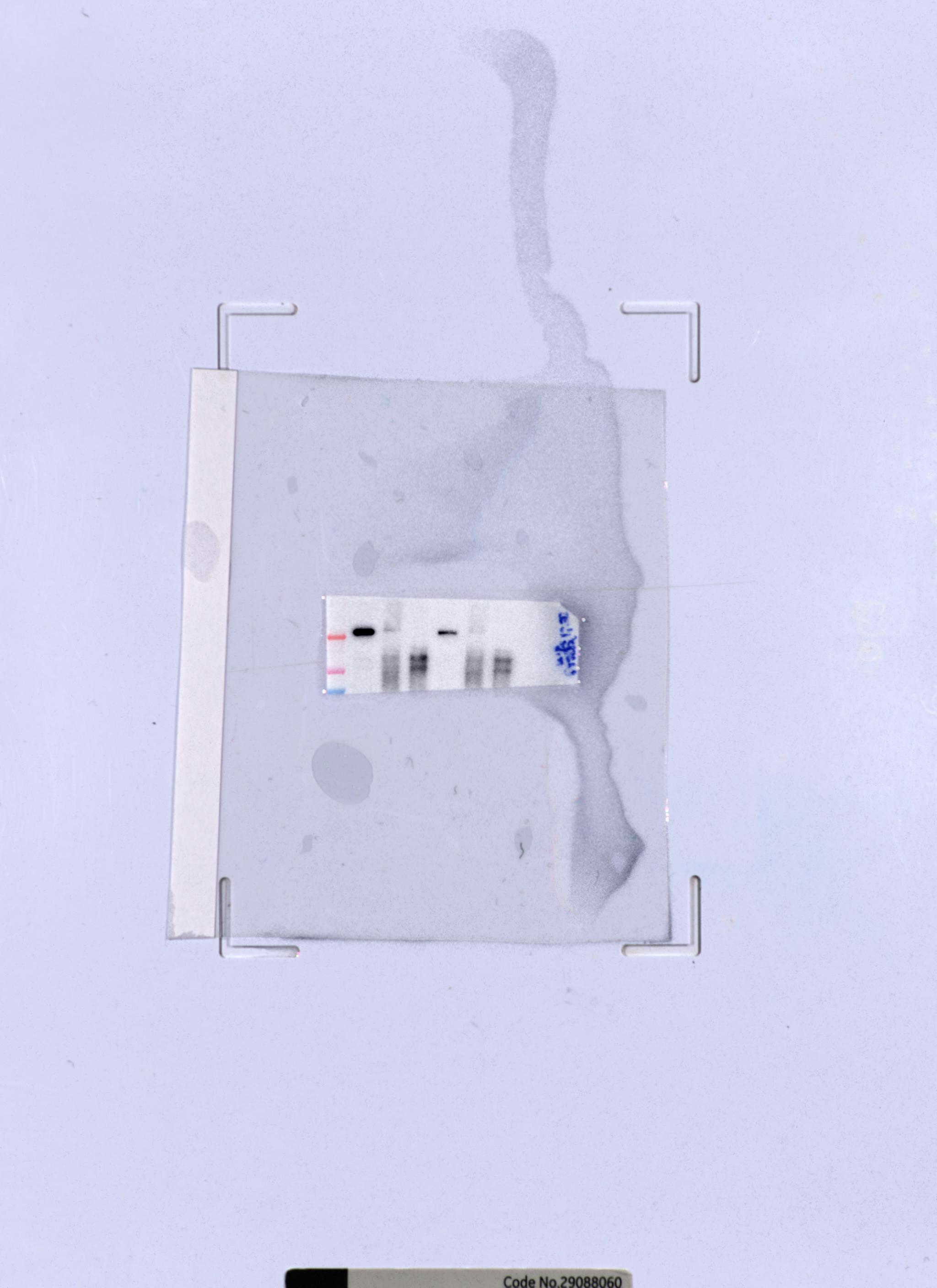


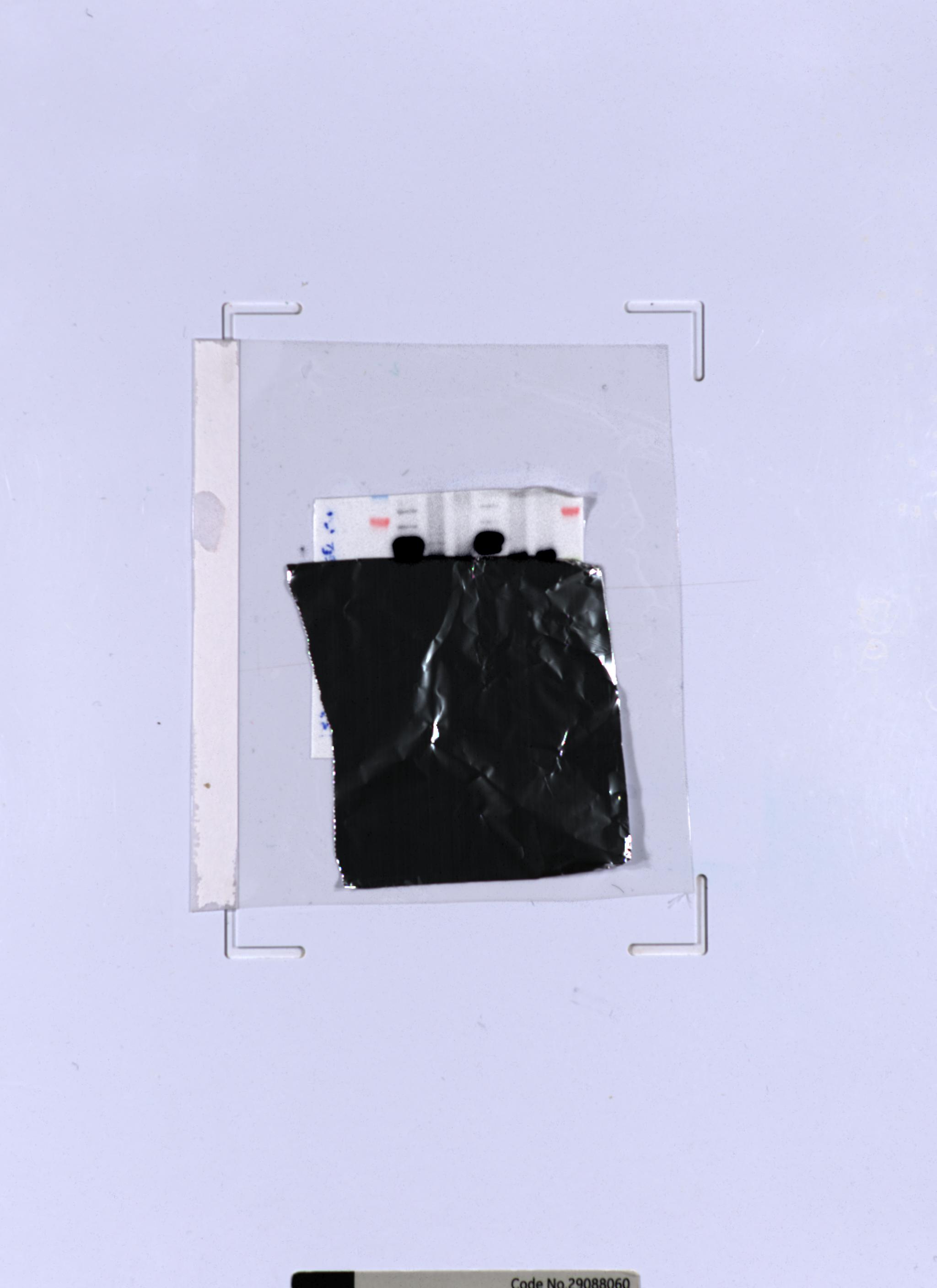
**ER 69kDa**

1 2 3 4 5 6 7 8

1 2 3 4 5 6 7 8


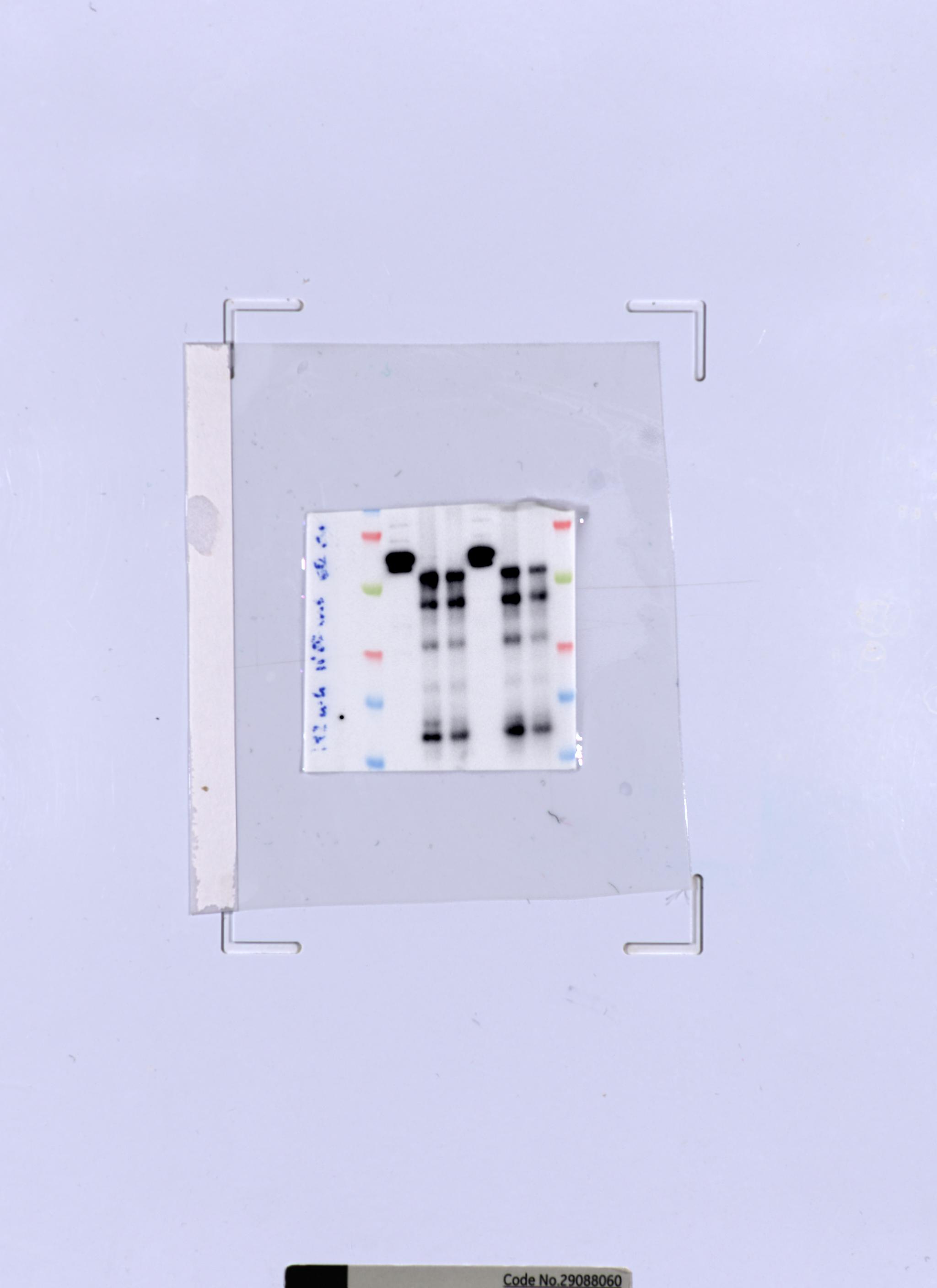


#### Replicate number 3 (shown in Figure 4B)

**Ponceau staining**

|  | Replicate n3 |
| --- | --- |
| Lane n | Sample ID |
| 1 | Molecular marker (260-15 kDa) |
| 2 | LY2 siCtrl – input |
| 3 | LY2 siCtrl – IP ER |
| 4 | LY2 siCtrl – IP IgG |
| 5 | LY2 siCDK12 – input |
| 6 | LY2 siCDK12 – IP ER |
| 7 | LY2 siCDK12 – IP igG |
| 8 | Molecular marker (260-15 kDa) |
| 9 | Molecular marker (260-15 kDa) |
| 10 | LY2 siCtrl – input |
| 11 | LY2 siCtrl – IP ER |
| 12 | LY2 siCtrl – IP IgG |
| 13 | LY2 siCDK12 – input |
| 14 | LY2 siCDK12 – IP ER |
| 15 | LY2 siCDK12 – IP igG |


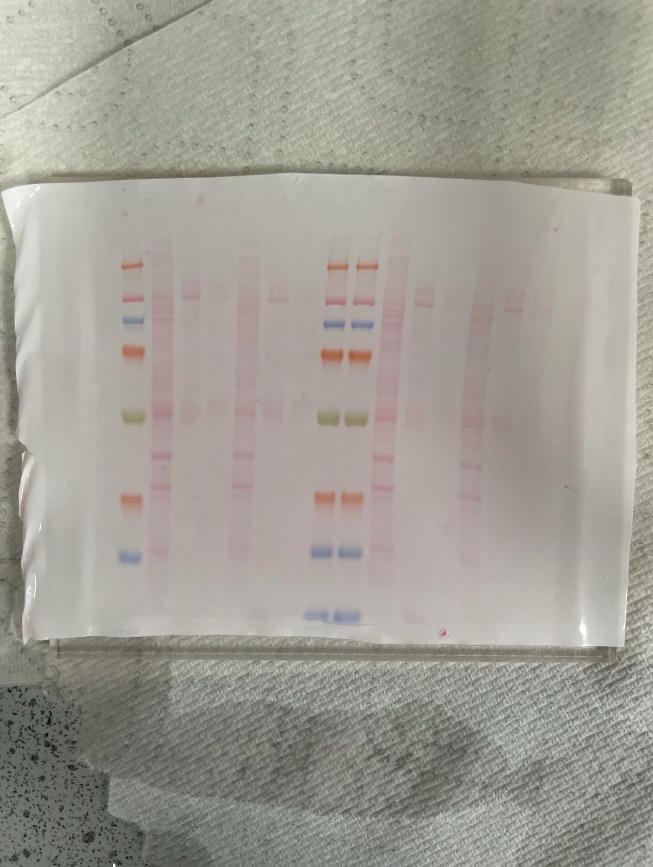


9 10 11 12 13 14 15

1 2 3 4 5 6 7 8

**MED1 220kDa**

1 2 3 4 5 6 7 8


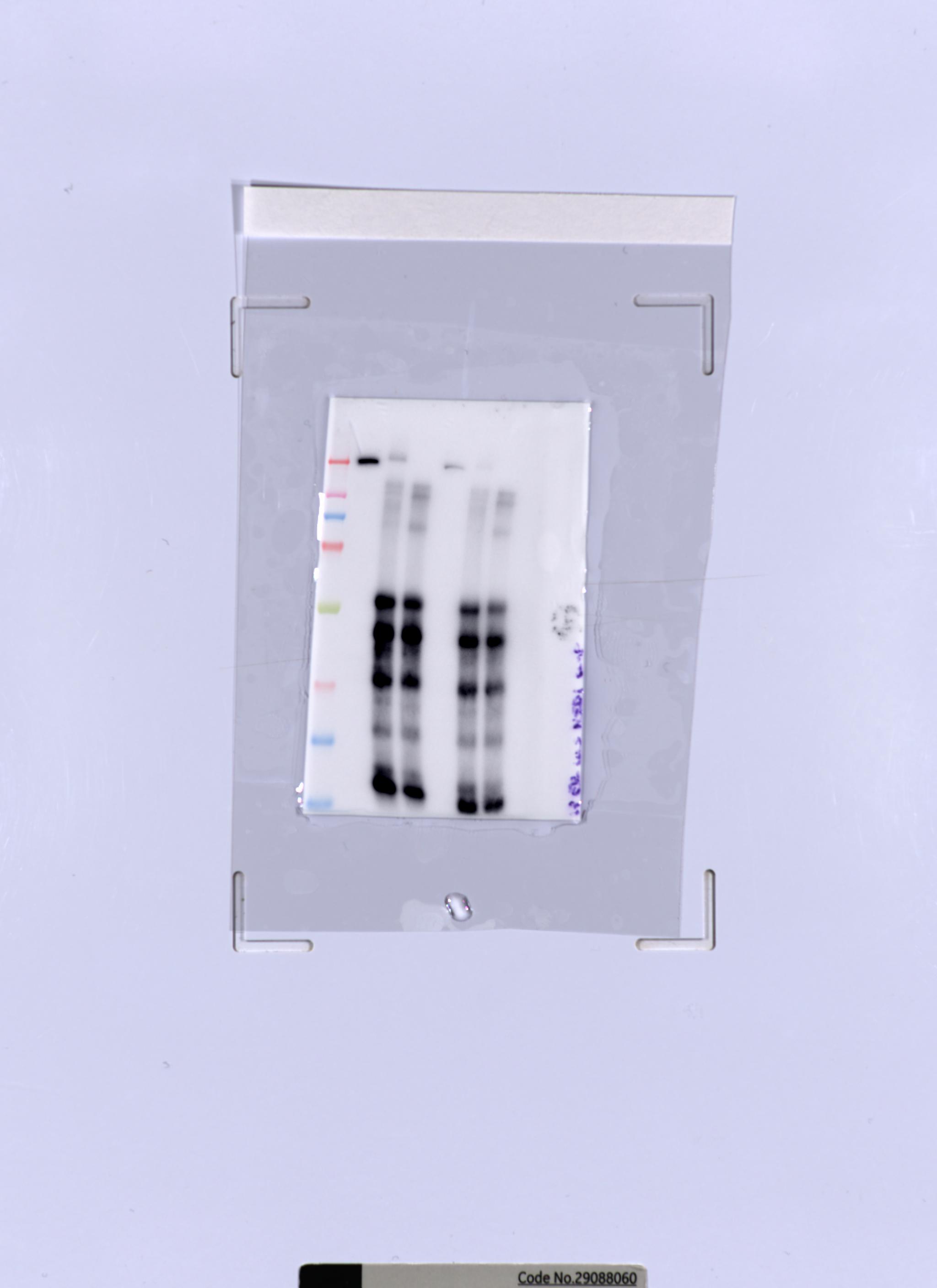

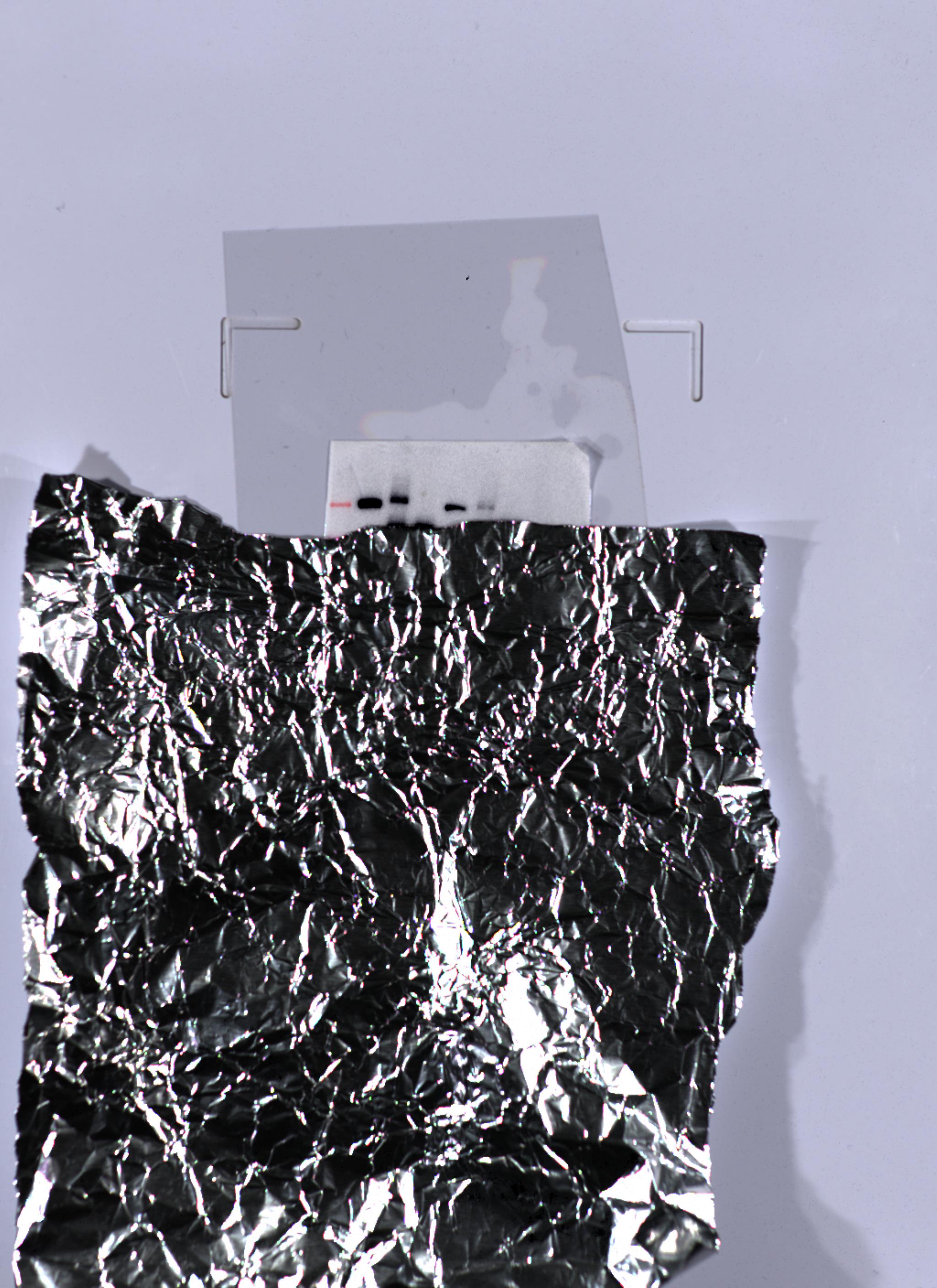

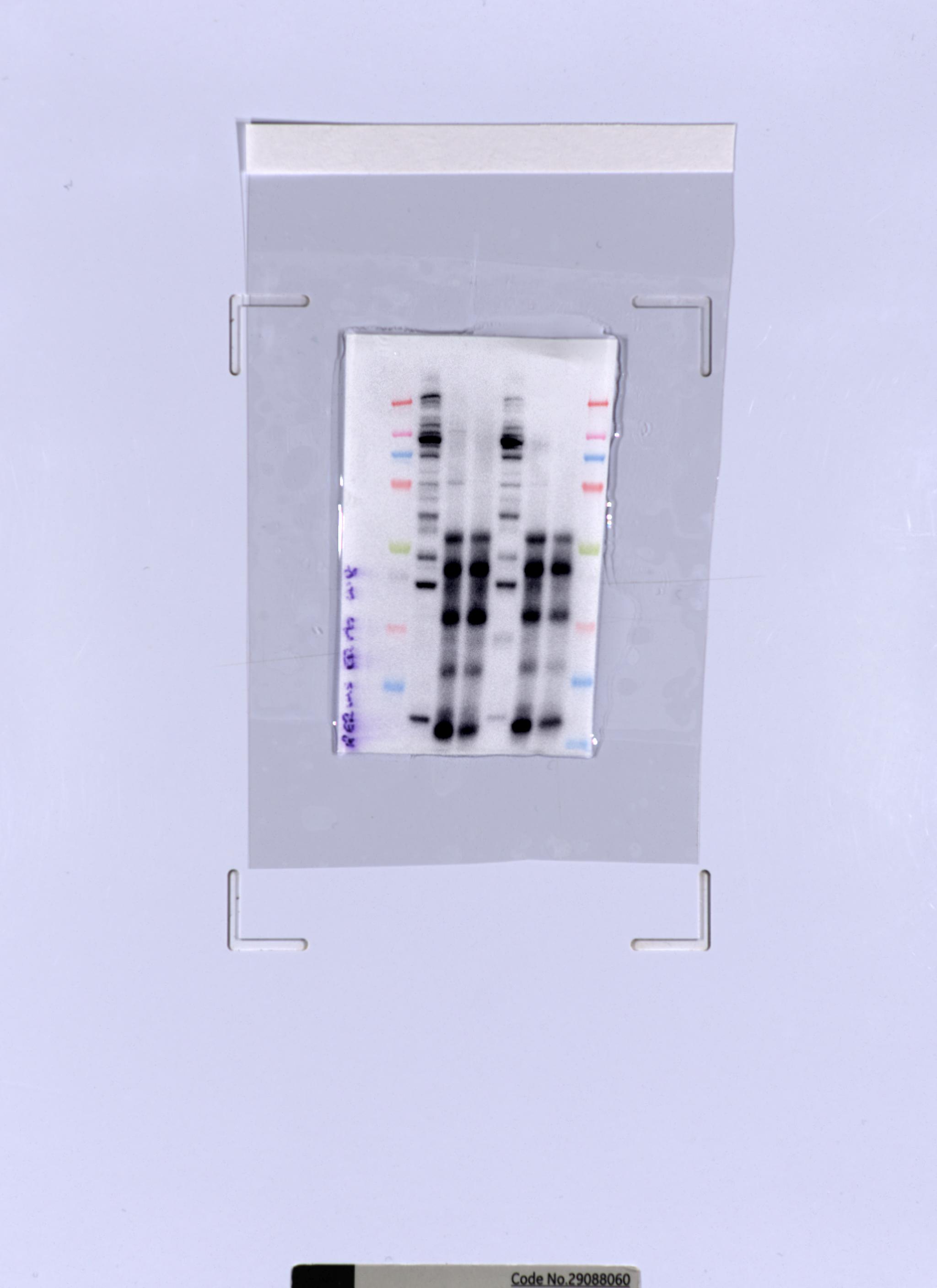


**pMED1 ~240kDa**

9 10 11 12 13 14 15

9 10 11 12 13 14 15

**ER 69kDa**

9 10 1112 13 14 15


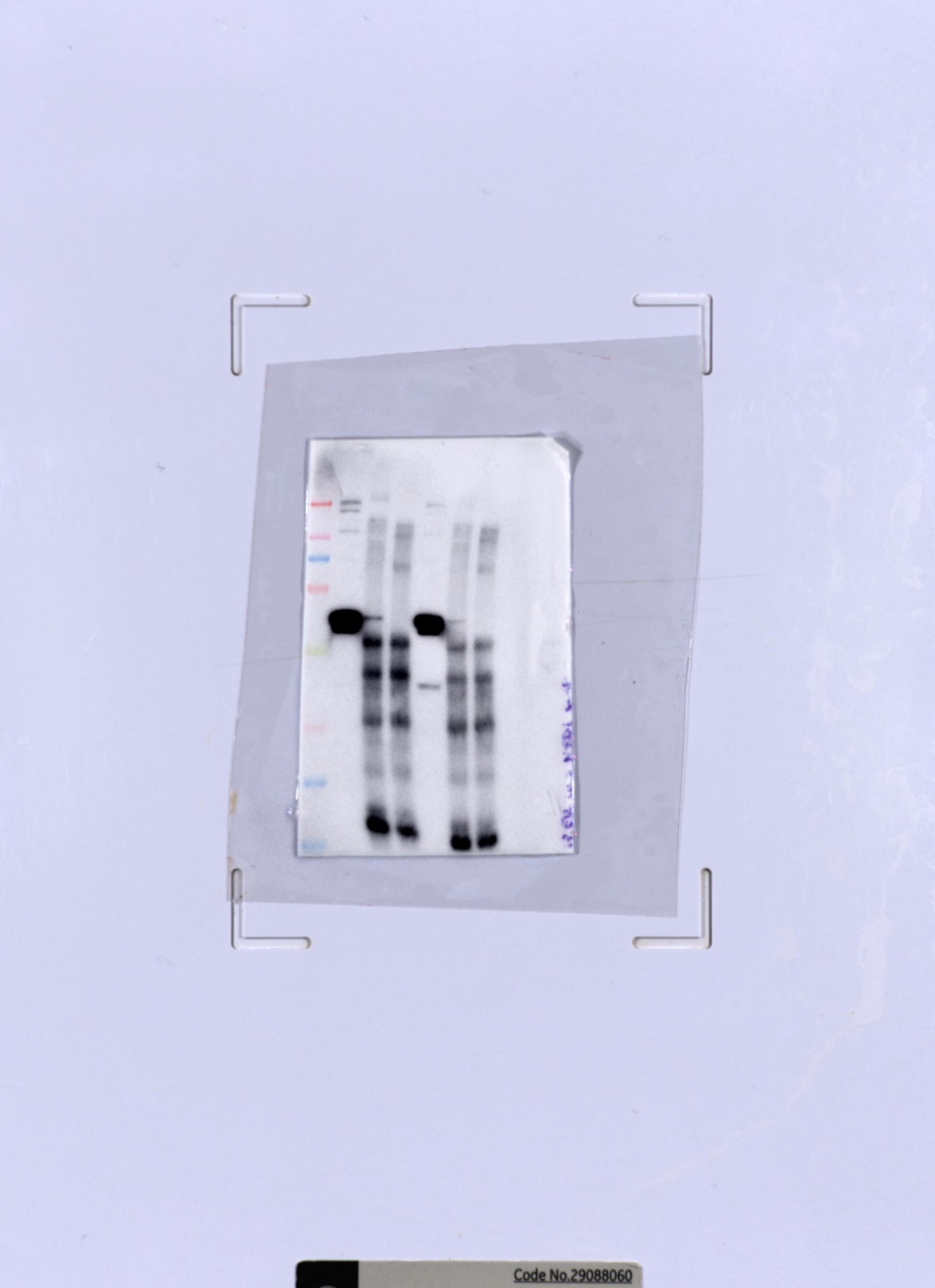


### LY2 - IP MED1

#### Replicate number 1 (shown in Figure 4B)

**Ponceau staining**

|  | Replicate n1 |
| --- | --- |
| Lane n | Sample ID |
| 1 | Molecular marker (260-15 kDa) |
| 2 | LY2 siCtrl – input |
| 3 | LY2 siCtrl – IP MED1 |
| 4 | LY2 siCtrl – IP IgG |
| 5 | LY2 siCDK12 – input |
| 6 | LY2 siCDK12 – IP MED1 |
| 7 | LY2 siCDK12 – IP igG |
| 8 | Molecular marker (260-15 kDa) |


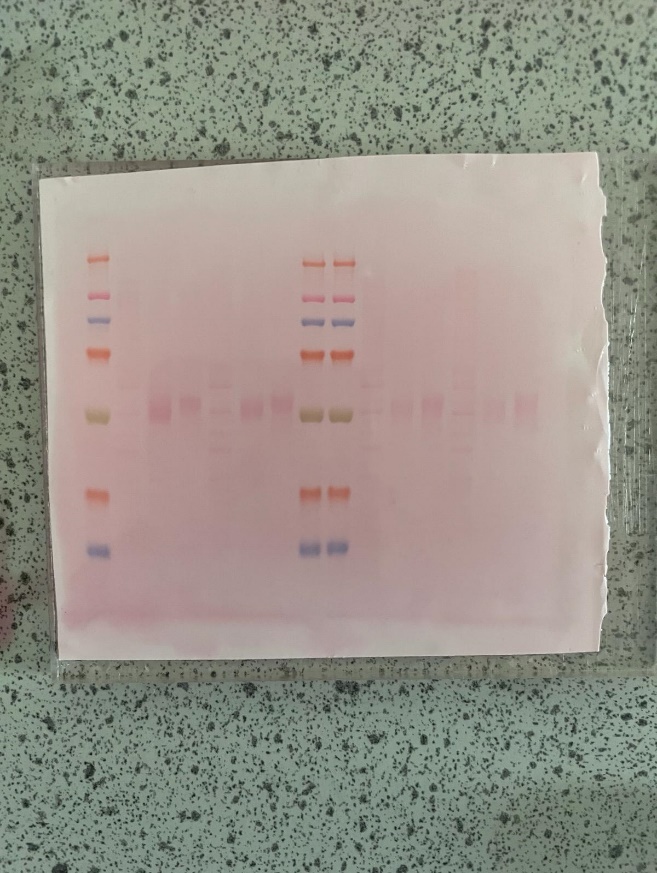


9 10 11 12 13 14 15

1 2 3 4 5 6 7 8

1 2 3 4 5 6 7 8


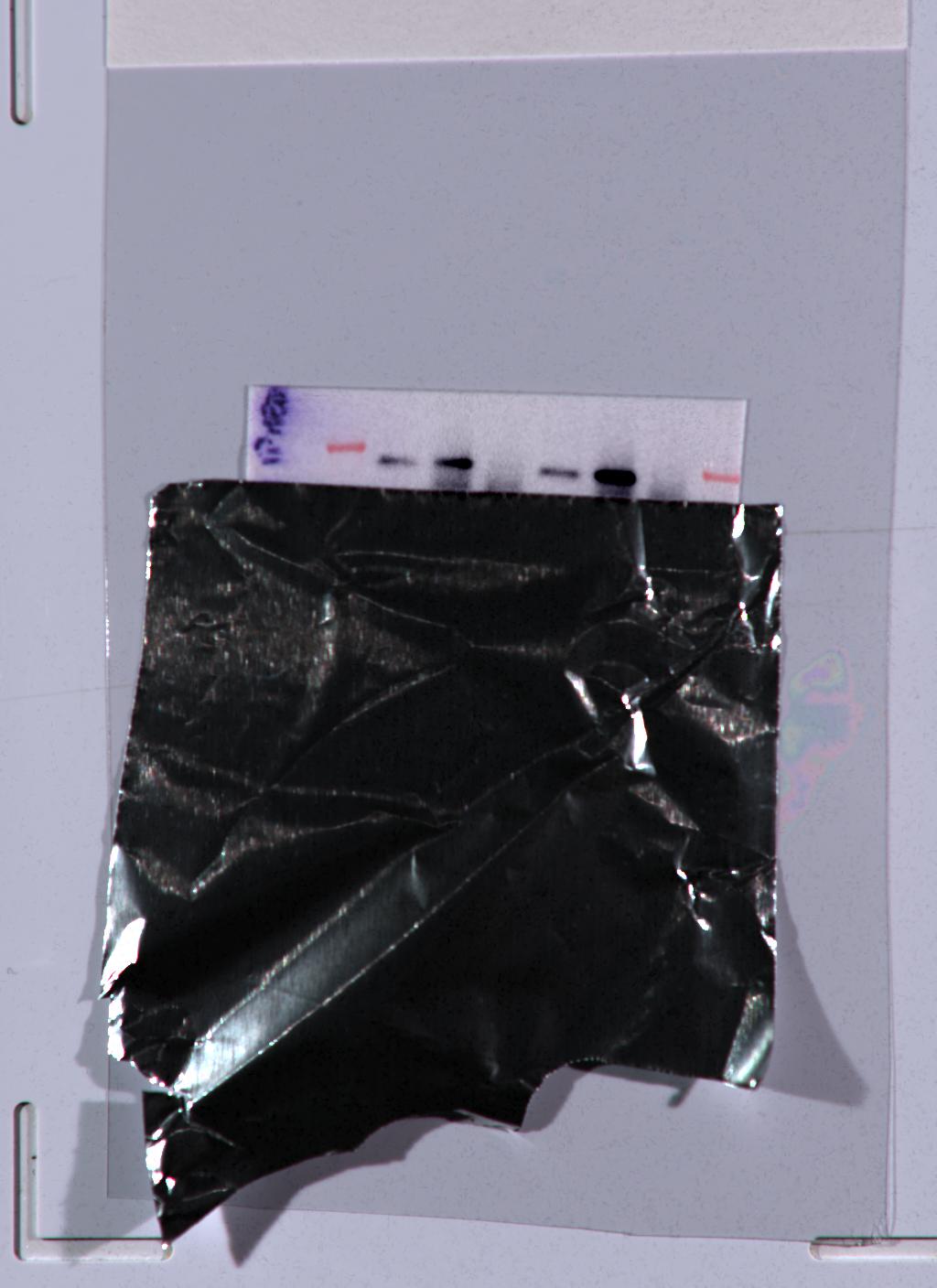
**MED1 220kDa**

1 2 3 4 5 6 7 8


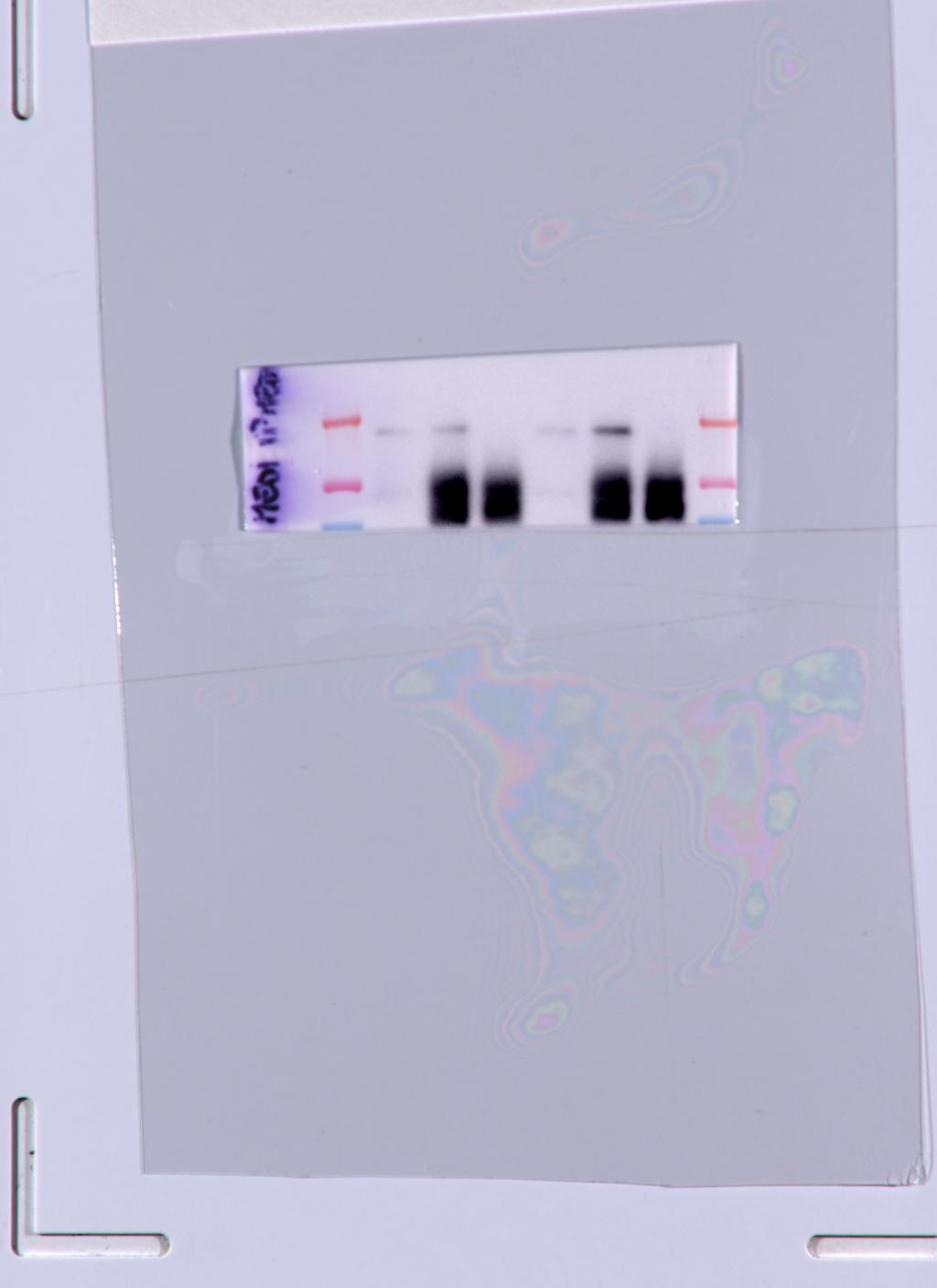


**ER 69kDa**

1 2 3 4 5 6 7 8


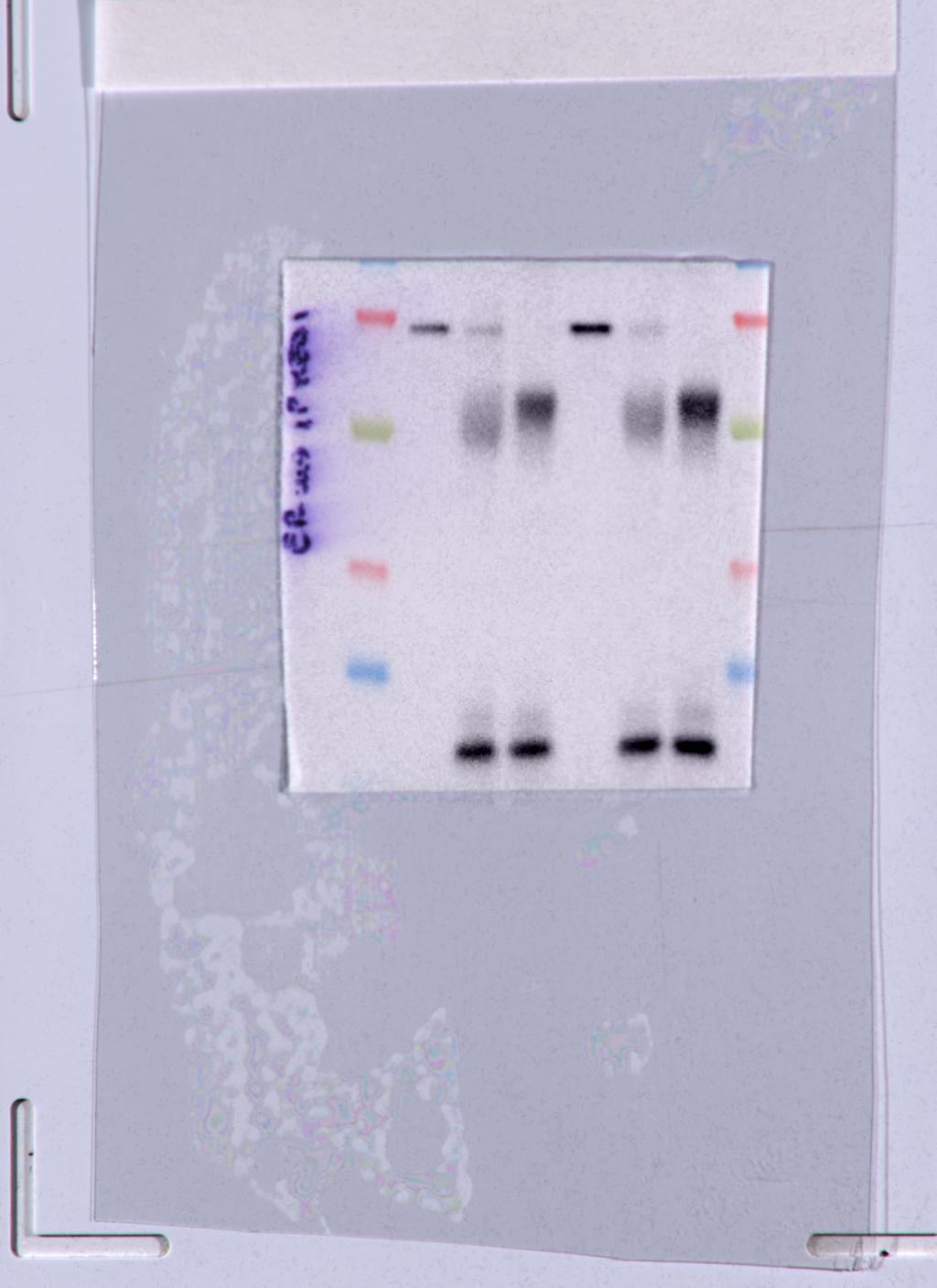


#### Replicate number 2

**Ponceau staining**

1 2 3 4 5 6 7 8

|  | Replicate n2 |
| --- | --- |
| Lane n | Sample ID |
| 1 | Molecular marker (260-15 kDa) |
| 2 | LY2 siCtrl – input |
| 3 | LY2 siCtrl – IP MED1 |
| 4 | LY2 siCtrl – IP IgG |
| 5 | LY2 siCDK12 – input |
| 6 | LY2 siCDK12 – IP MED1 |
| 7 | LY2 siCDK12 – IP igG |
| 8 | Molecular marker (260-15 kDa) |


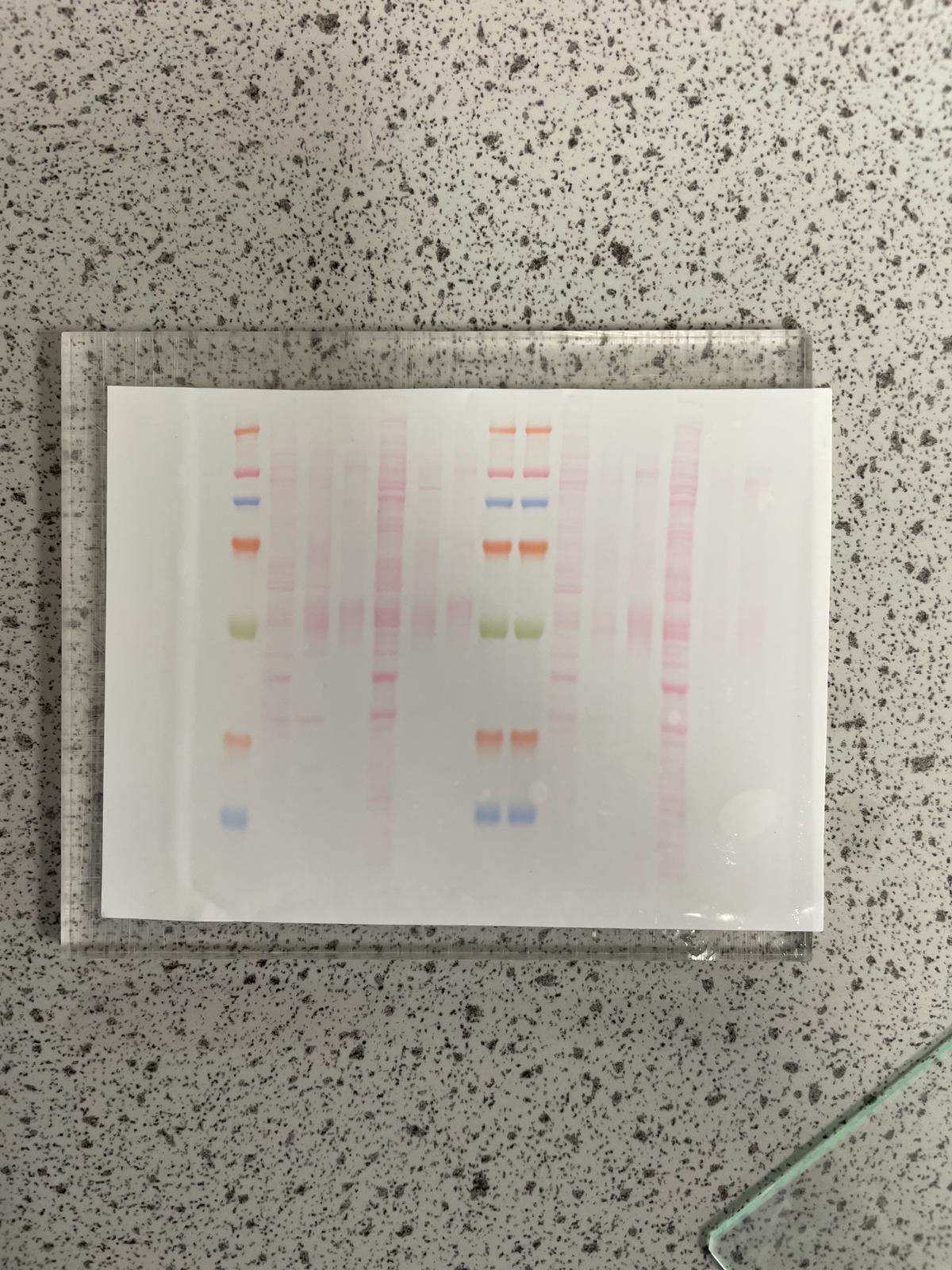


**MED1 220kDa**

1 2 3 4 5 6 7 8

1 2 3 4 5 6 7 8


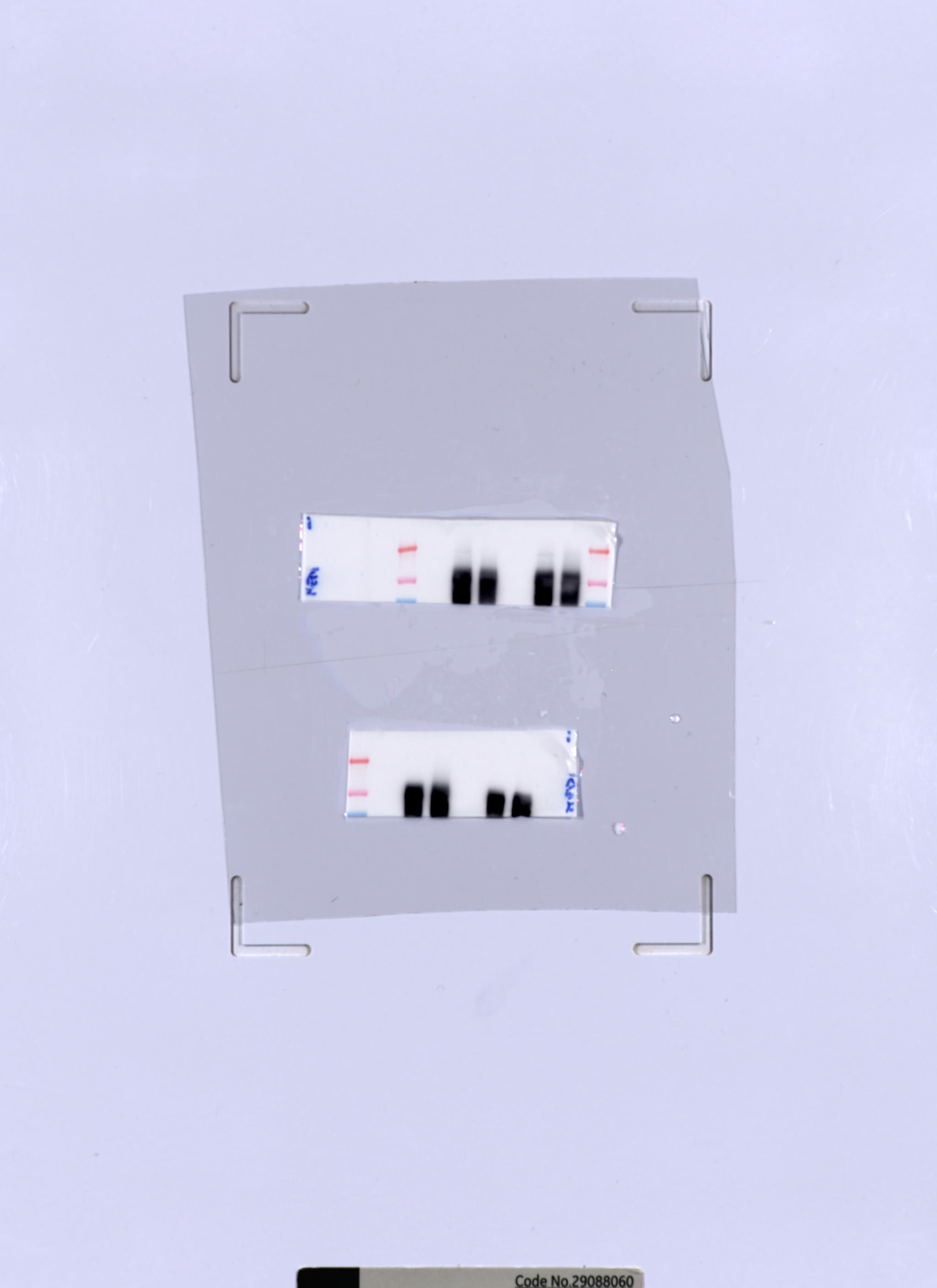


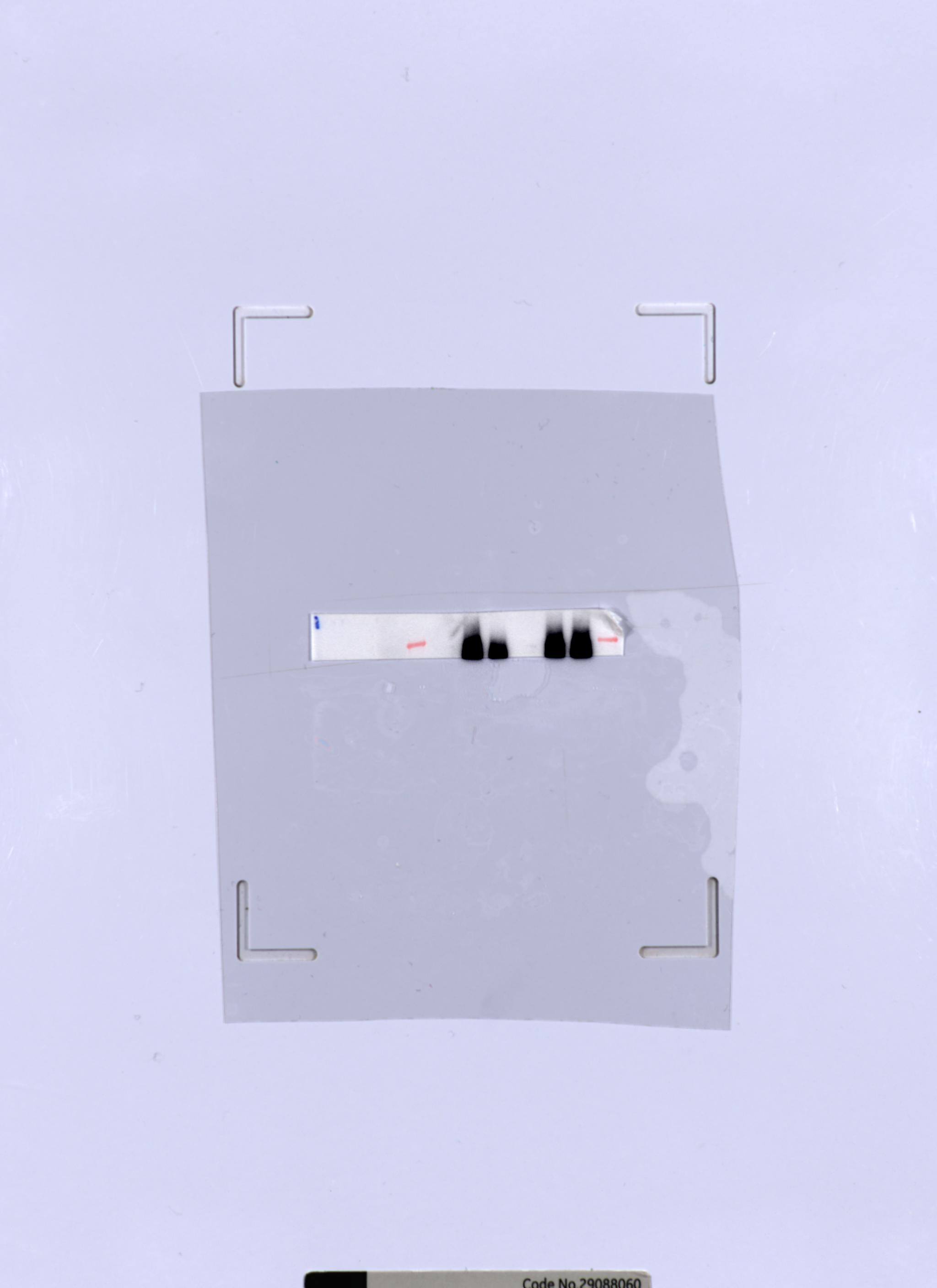


**ER 69kDa**

1 2 3 4 5 6 7 8


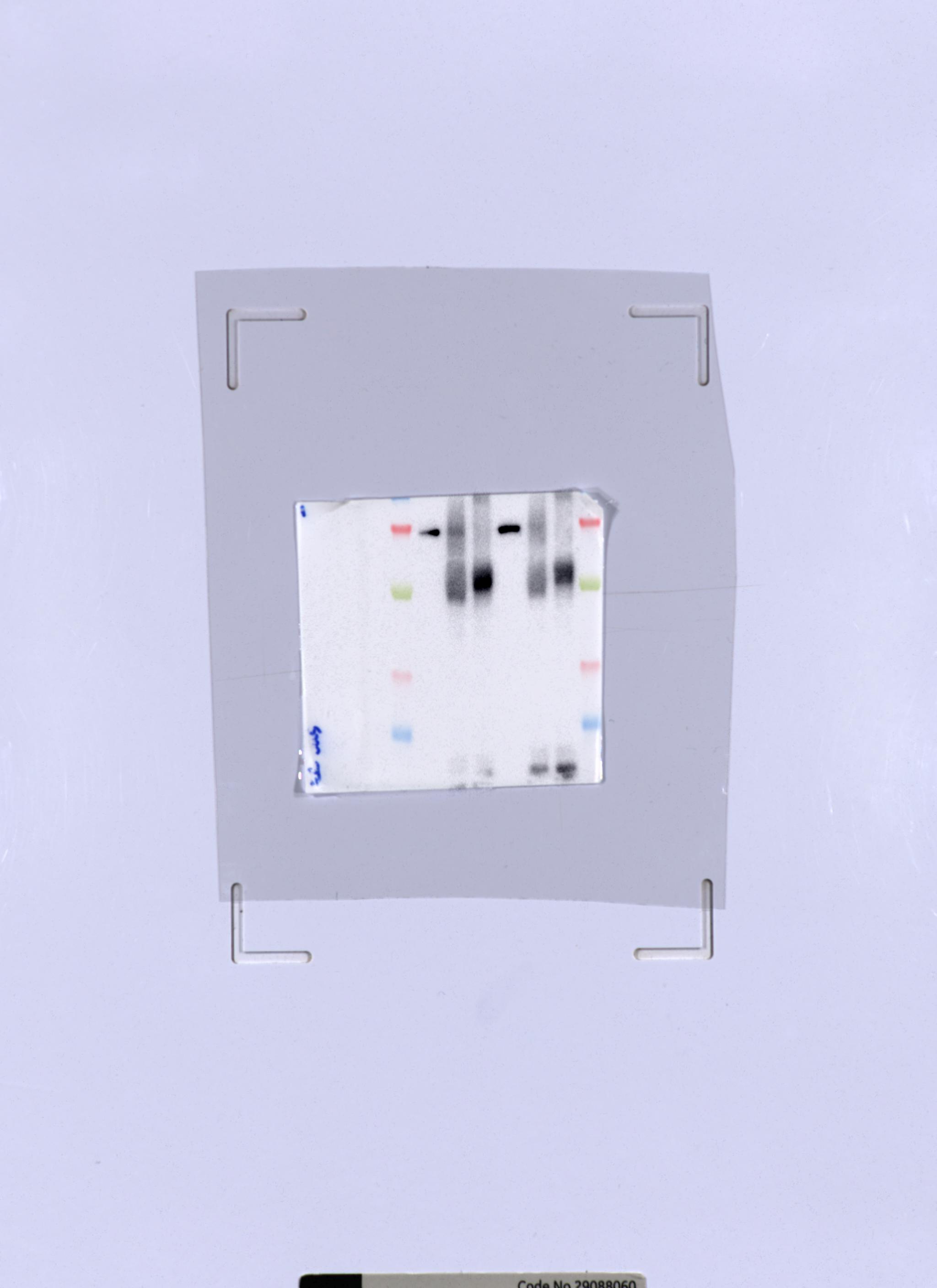


#### Replicate number 3

**Ponceau staining**

|  | Replicate n3 |
| --- | --- |
| Lane n | Sample ID |
| 1 | Molecular marker (260-15 kDa) |
| 2 | LY2 siCtrl – input |
| 3 | LY2 siCtrl – IP MED1 |
| 4 | LY2 siCtrl – IP IgG |
| 5 | LY2 siCDK12 – input |
| 6 | LY2 siCDK12 – IP MED1 |
| 7 | LY2 siCDK12 – IP igG |
| 8 | Molecular marker (260-15 kDa) |
| 9 | Molecular marker (260-15 kDa) |
| 10 | LY2 siCtrl – input |
| 11 | LY2 siCtrl – IP MED1 |
| 12 | LY2 siCtrl – IP IgG |
| 13 | LY2 siCDK12 – input |
| 14 | LY2 siCDK12 – IP MED1 |
| 15 | LY2 siCDK12 – IP igG |


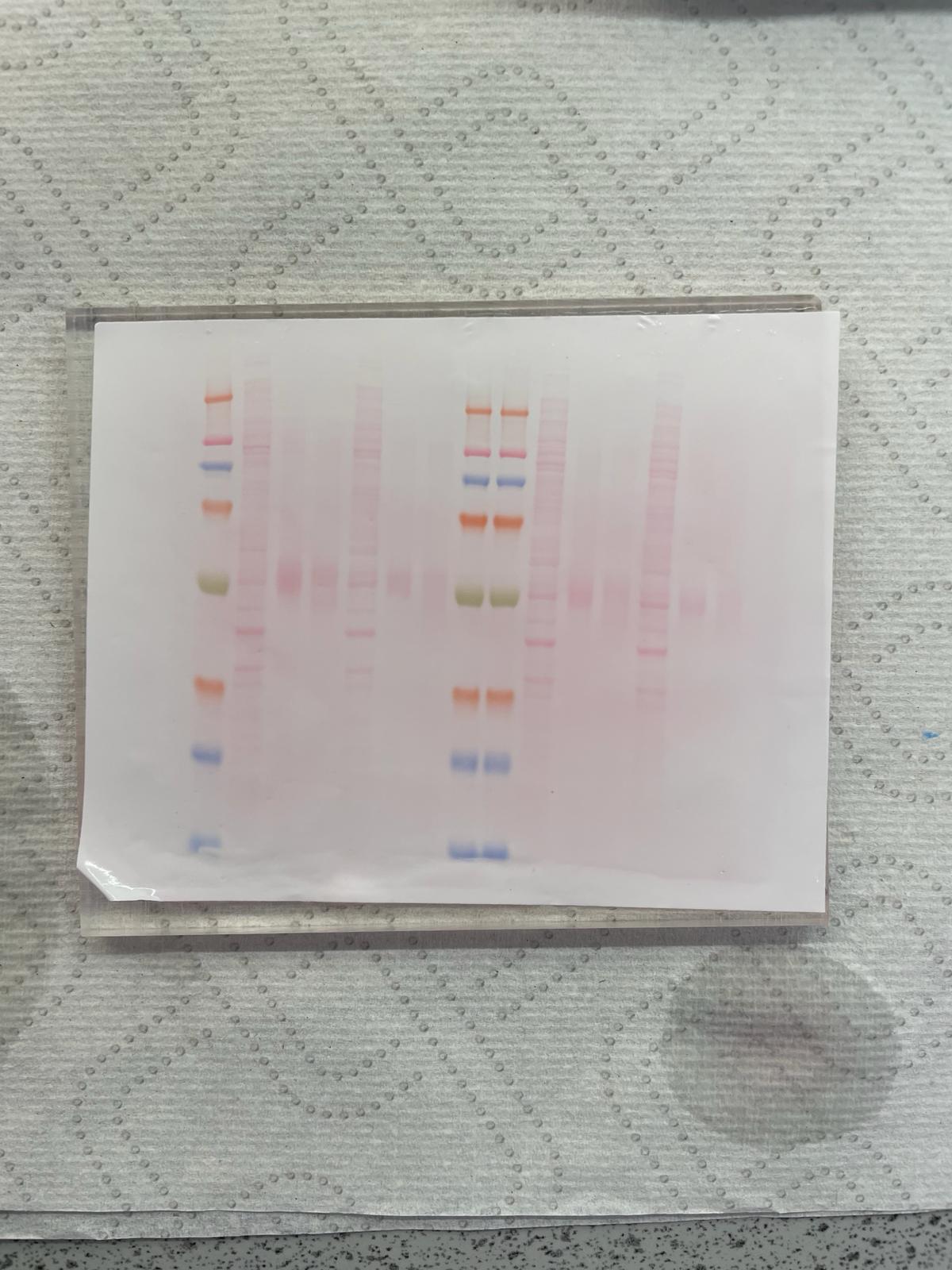


**MED1 220kDa**

9 10 11 12 13 14 15


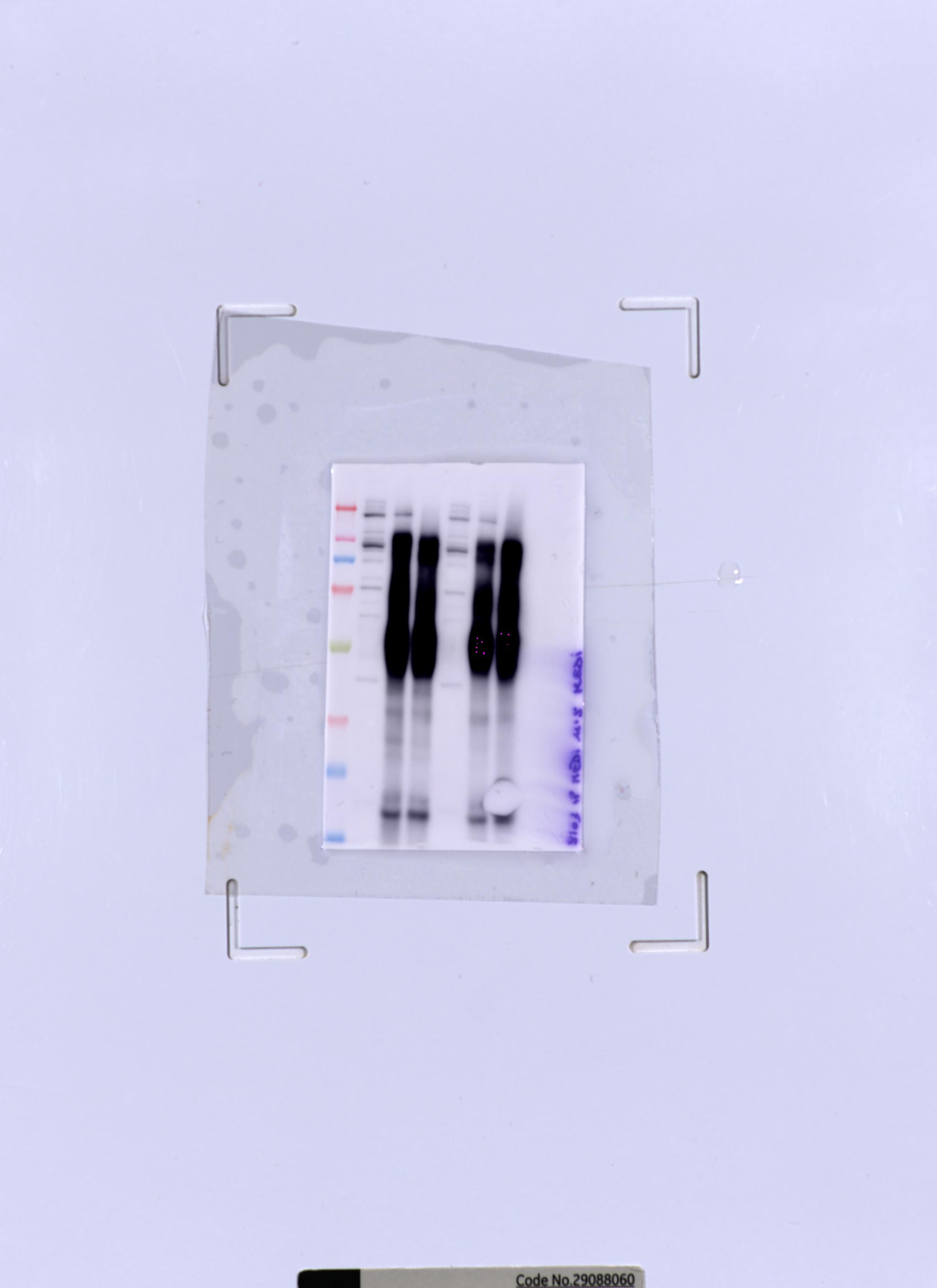


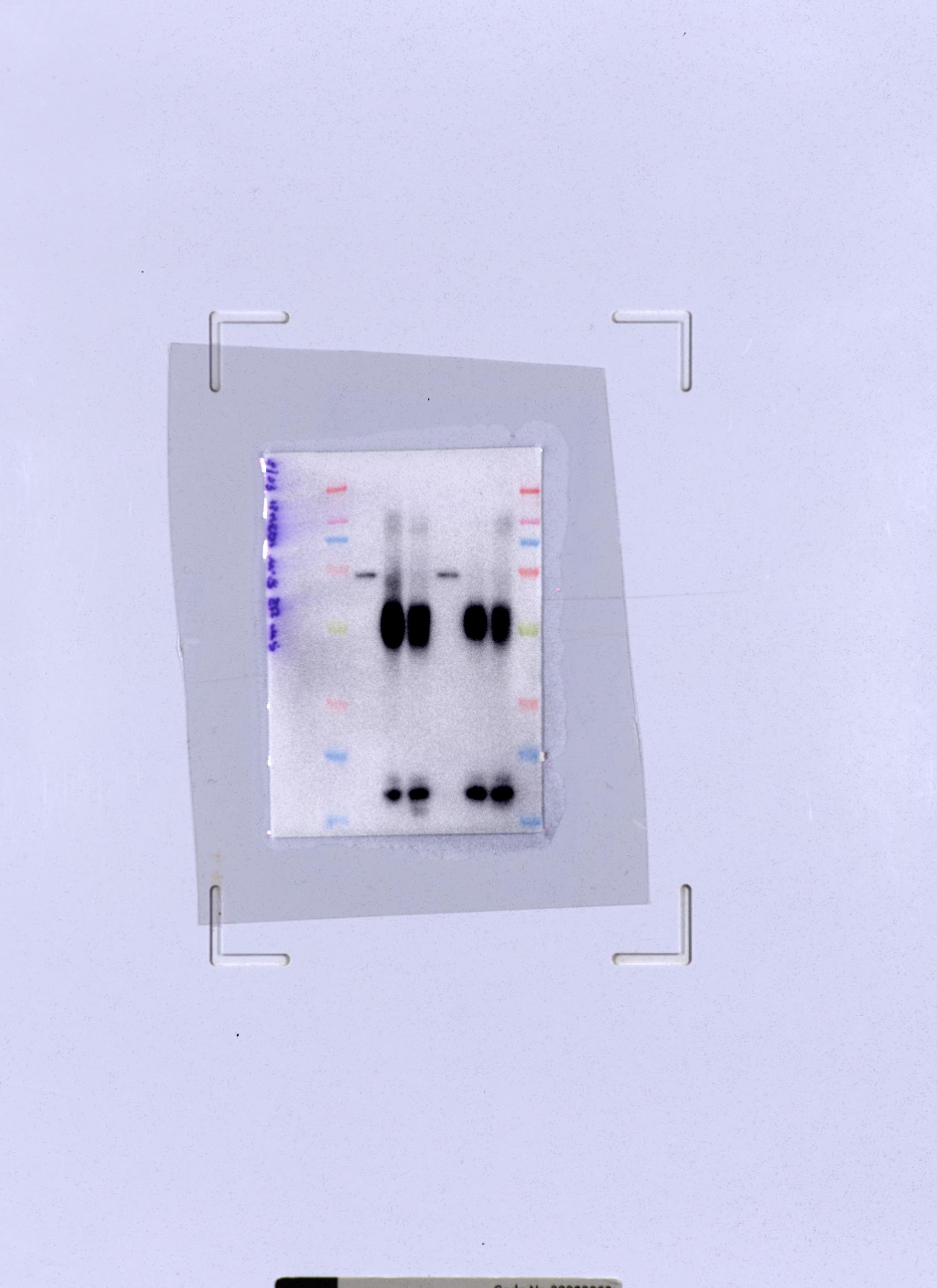


1 2 3 4 5 6 7 8

**ER 69kDa**

## Supplementary Material 5. Uncropped western blot membranes in Figure 5E.

**Protein expression following CT7116 treatment (0.3 µmol/L, 24 hours) in LY2, LCC9, LY2 bone and T347 cells.** CDK12, MED1, pMED1, ER + loading controls for cytoplasmic (GAPDH), nuclear (LMNB2) and chromatin-bound fractions (Histone 3).

### LY2 cells

#### Replicate number 1 (shown in Figure 5E)

**Ponceau staining**

|  | Replicate n1 |
| --- | --- |
| Lane n | Sample ID |
| 1 | Molecular marker (260-15 kDa) |
| 2 | LY2 DMSO – Cyt. (n1) |
| 3 | LY2 DMSO – Nucl. (n1) |
| 4 | LY2 DMSO – Chrom. (n1) |
| 5 | LY2 CT7116 – Cyt. (n1) |
| 6 | LY2 CT7116 – Nucl. (n1) |
| 7 | LY2 CT7116 – Chrom. (n1) |
| 8 | Molecular marker (260-15 kDa) |


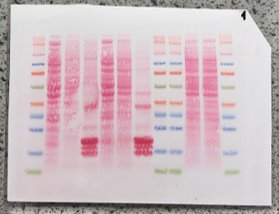


1 2 3 4 5 6 7 8

**CDK12 205 kDa**


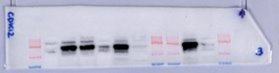


2 3 4 5 6 7

**MED1 220 kDa**


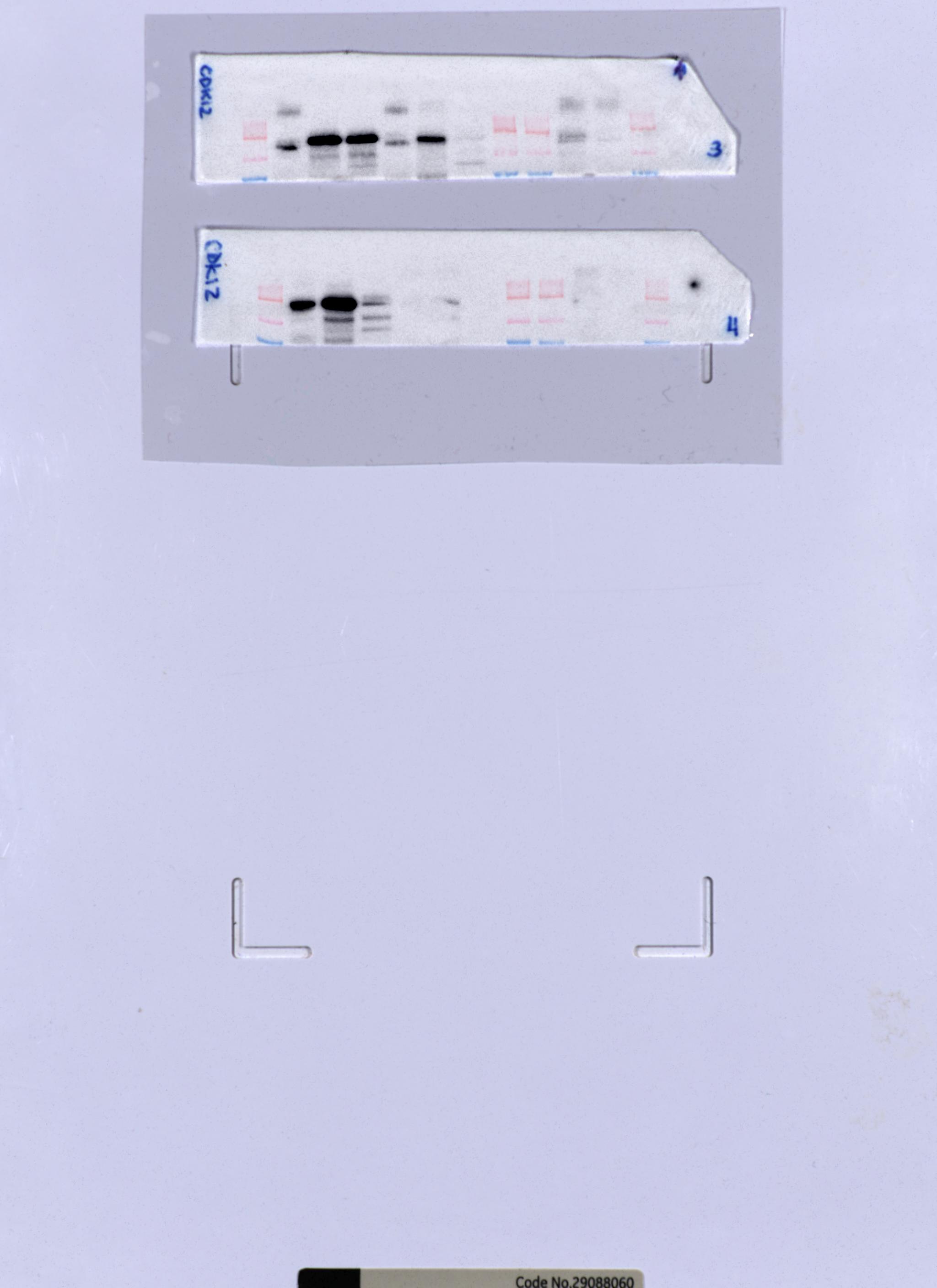


2 3 4 5 6 7

**pMED1 ~240 kDa**


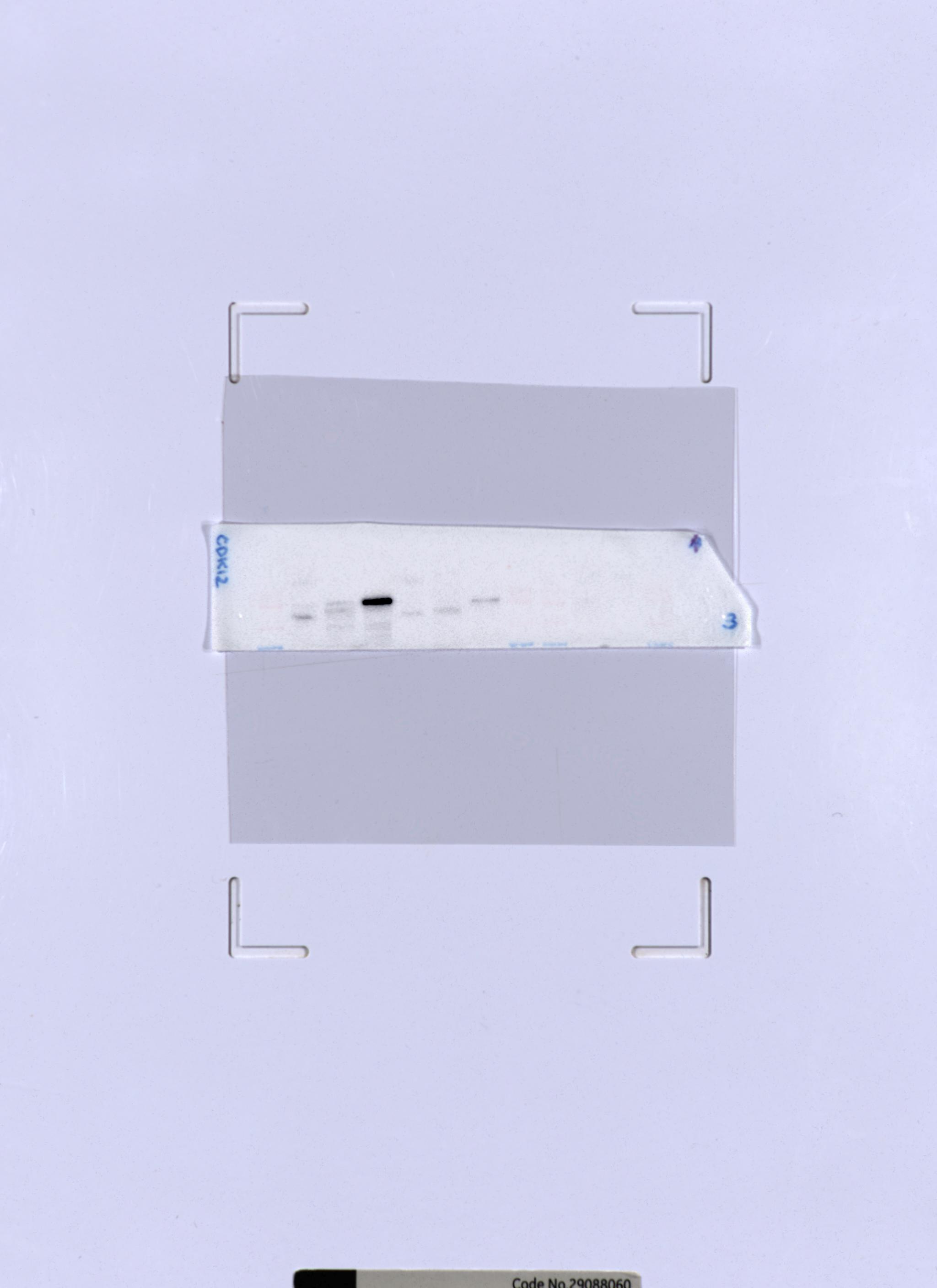


2 3 4 5 6 7

**ER 60 kDa**


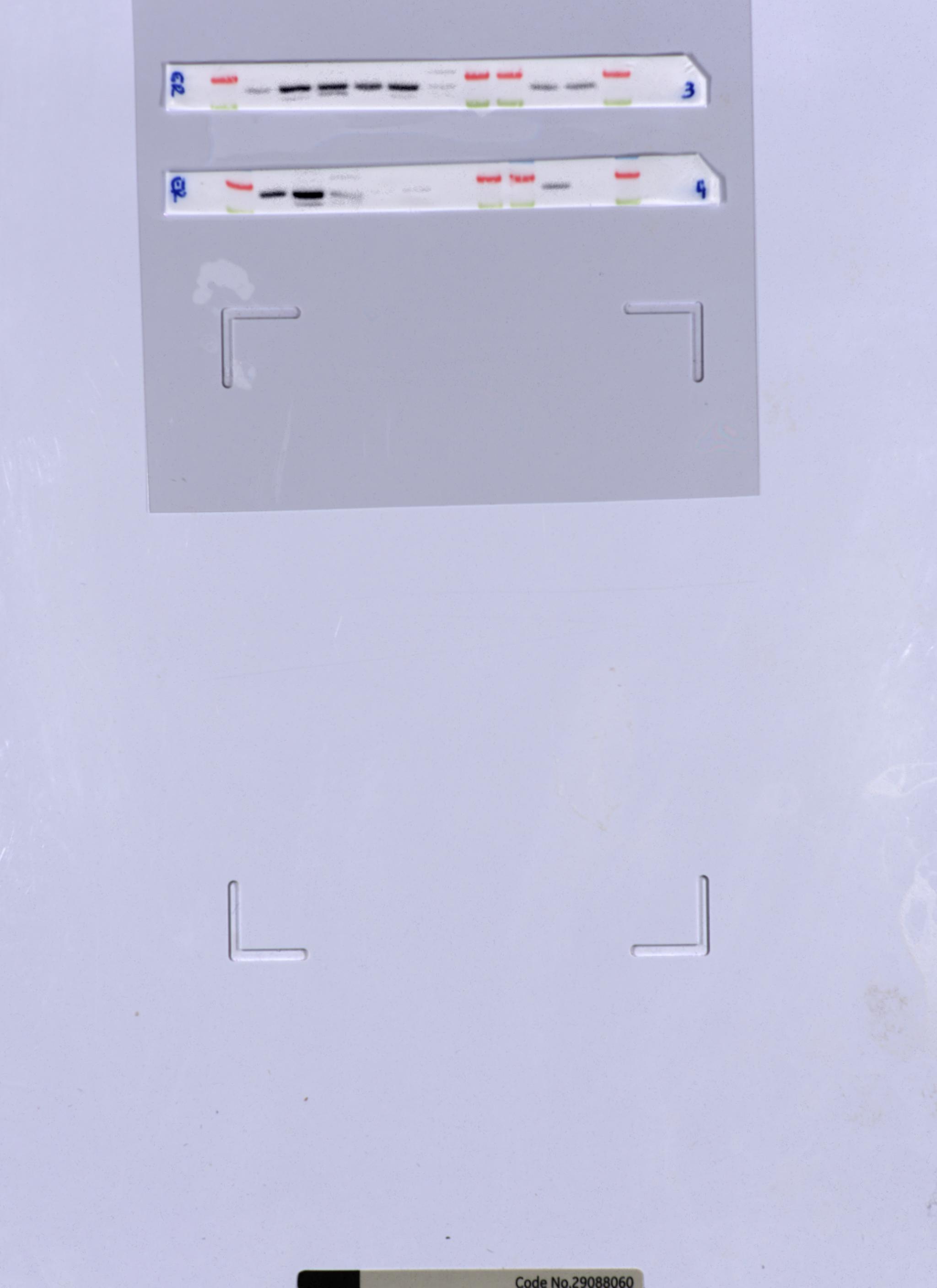


2 3 4 5 6 7

**LMNB2 68 kDa**


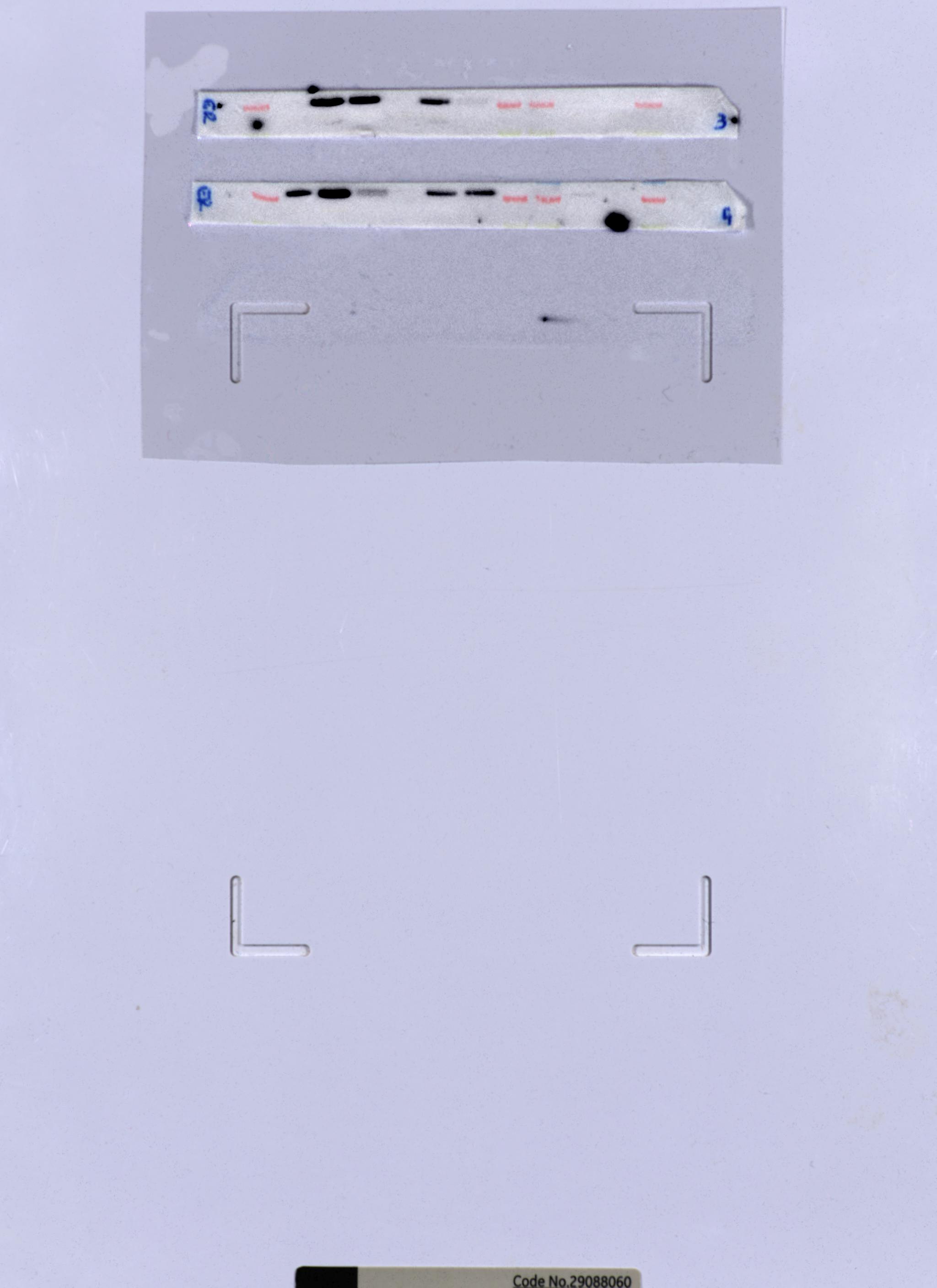


2 3 4 5 6 7

**GAPDH 38 kDa**


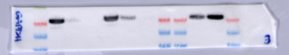


2 3 4 5 6 7

**Histone 3 17 kDa**


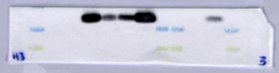


2 3 4 5 6 7

#### Replicate number 2

Membrane I (Immunoblotting: CDK12, pMED1, ER, LMNB2, GAPDH, Histone 3).

|  | Replicate n2 |
| --- | --- |
| Lane n | Sample ID |
| 1 | Molecular marker (260-15 kDa) |
| 2 | LY2 DMSO – Cyt. (n2) |
| 3 | LY2 DMSO – Nucl. (n2) |
| 4 | LY2 DMSO – Chrom. (n2) |
| 5 | LY2 CT7116 – Cyt. (n2) |
| 6 | LY2 CT7116 – Nucl. (n2) |
| 7 | LY2 CT7116 – Chrom. (n2) |
| 8 | Molecular marker (260-15 kDa) |

**Ponceau staining**


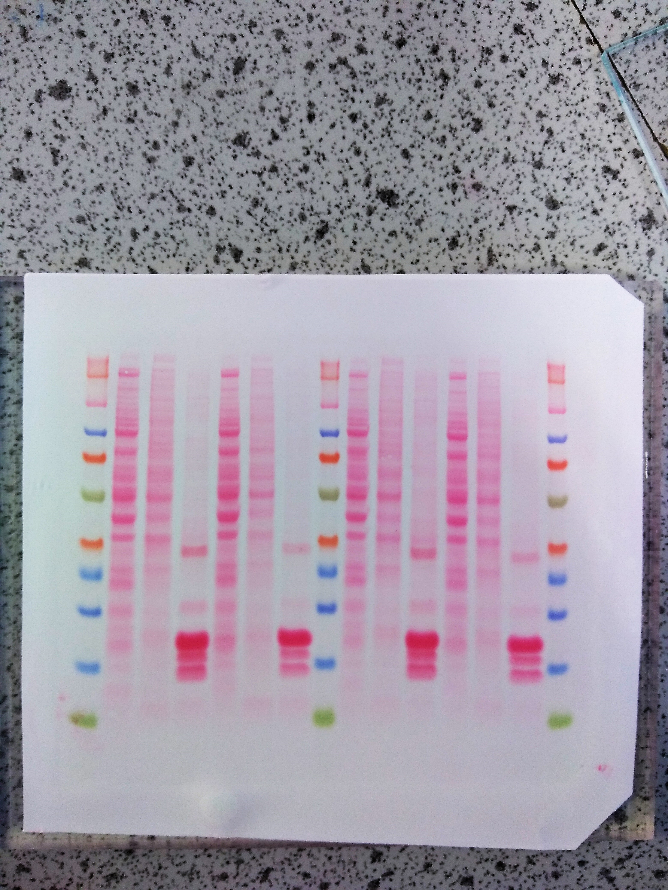


1 2 3 4 5 6 7 8

**CDK12 205 kDa**


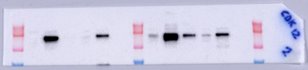


2 3 4 5 6 7

**pMED1 ~240 kDa**


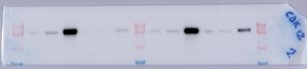


2 3 4 5 6 7

**ER 60 kDa**


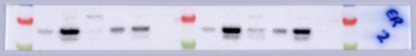


2 3 4 5 6 7

**
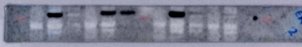
LMNB2 68 kDa**

2 3 4 5 6 7

**GAPDH 38 kDa**


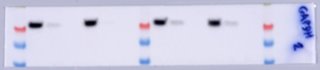


2 3 4 5 6 7

**Histone 3 17 kDa**


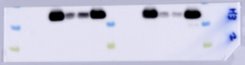


2 3 4 5 6 7

Membrane II (Immunoblotting: MED1, LMNB2, GAPDH, Histone 3).

**
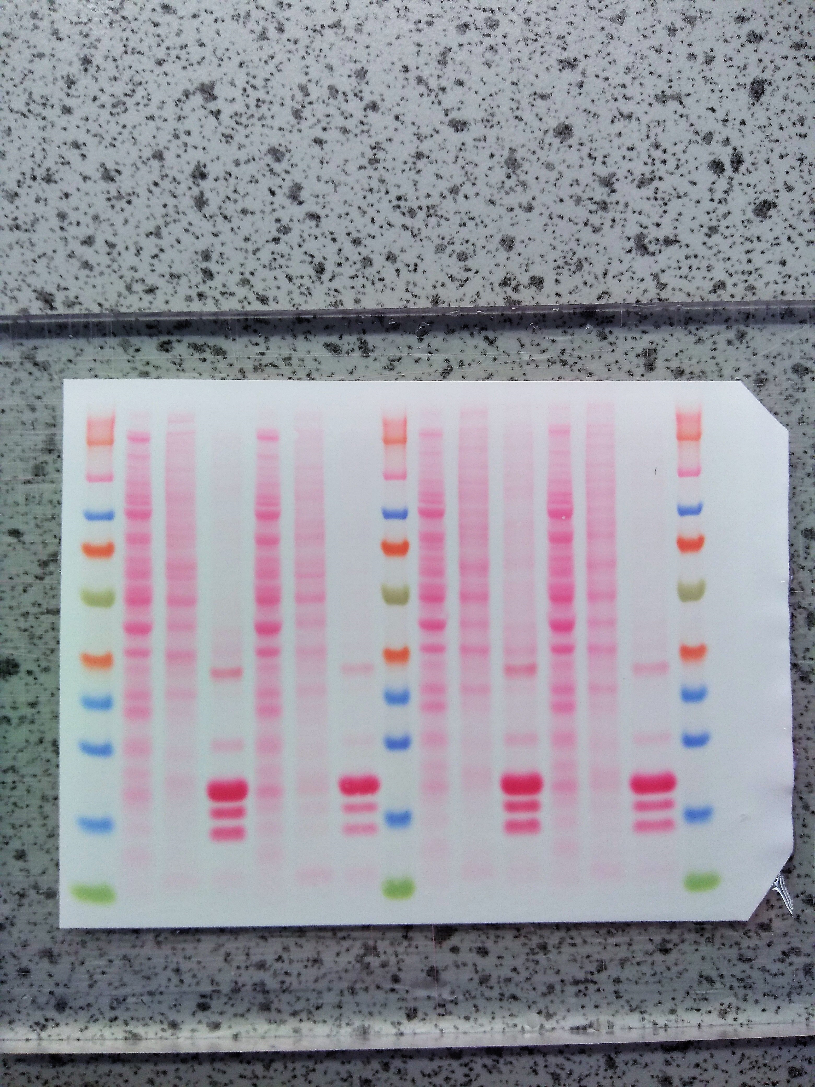
Ponceau staining**

1 2 3 4 5 6 7 8

|  | Replicate n2 |
| --- | --- |
| Lane n | Sample ID |
| 1 | Molecular marker (260-15 kDa) |
| 2 | LY2 DMSO – Cyt. (n2) |
| 3 | LY2 DMSO – Nucl. (n2) |
| 4 | LY2 DMSO – Chrom. (n2) |
| 5 | LY2 CT7116 – Cyt. (n2) |
| 6 | LY2 CT7116 – Nucl. (n2) |
| 7 | LY2 CT7116 – Chrom. (n2) |
| 8 | Molecular marker (260-15 kDa) |

**
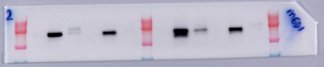
MED1 220 kDa**

2 3 4 5 6 7

**
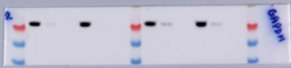
GAPDH 38 kDa**

2 3 4 5 6 7

**
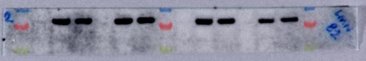
LMNB2 68 kDa**

2 3 4 5 6 7

**
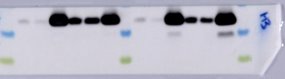
Histone 3 17 kDa**

2 3 4 5 6 7

#### Replicate number 3

**
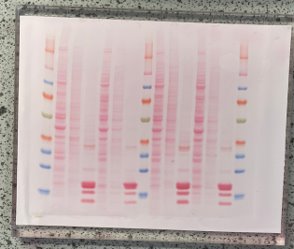
Ponceau staining**

|  | Replicate n3 |
| --- | --- |
| Lane n | Sample ID |
| 1 | Molecular marker (260-15 kDa) |
| 2 | LY2 DMSO – Cyt. (n3) |
| 3 | LY2 DMSO – Nucl. (n3) |
| 4 | LY2 DMSO – Chrom. (n3) |
| 5 | LY2 CT7116 – Cyt. (n3) |
| 6 | LY2 CT7116 – Nucl. (n3) |
| 7 | LY2 CT7116 – Chrom. (n3) |
| 8 | Molecular marker (260-15 kDa) |

1 2 3 4 5 6 7 8

2 3 4 5 6 7

**
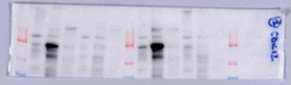
CDK12 205 kDa**

2 3 4 5 6 7

**
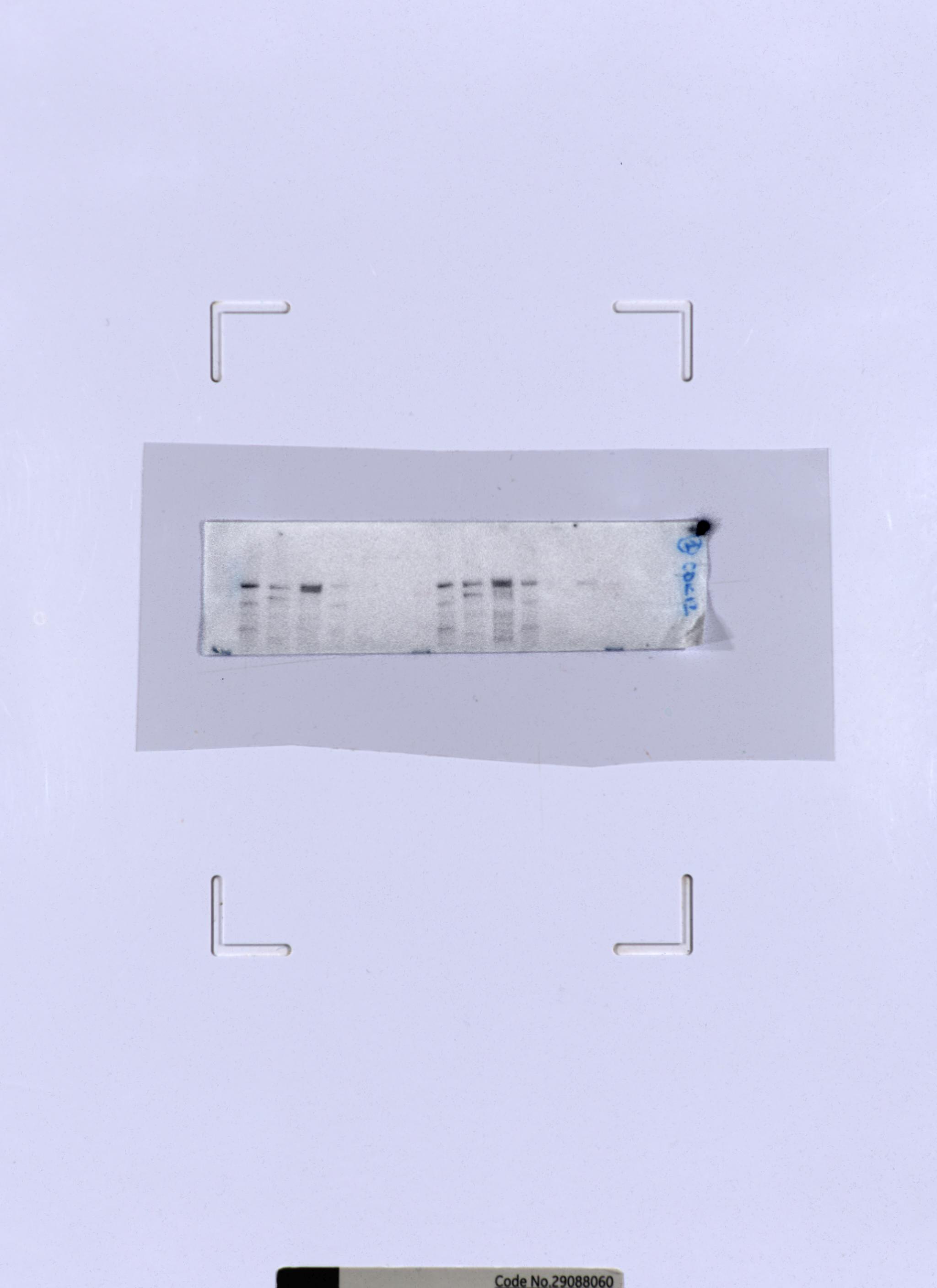
MED1 220 kDa**

2 3 4 5 6 7

**pMED1 ~240 kDa**


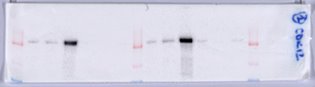


2 3 4 5 6 7

**ER 60 kDa**

2 3 4 5 6 7


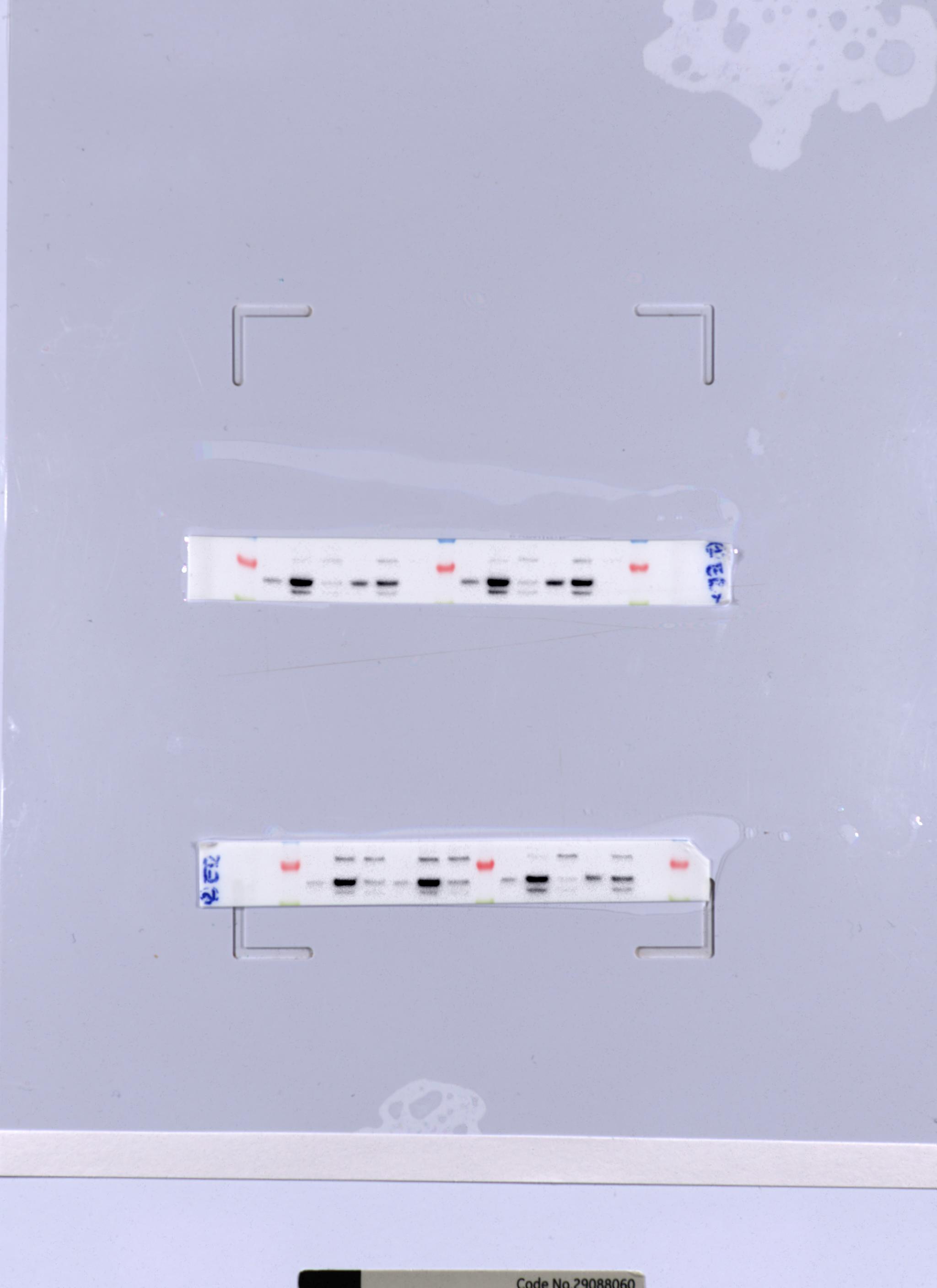


**LMNB2** **68 kDa**

2 3 4 5 6 7


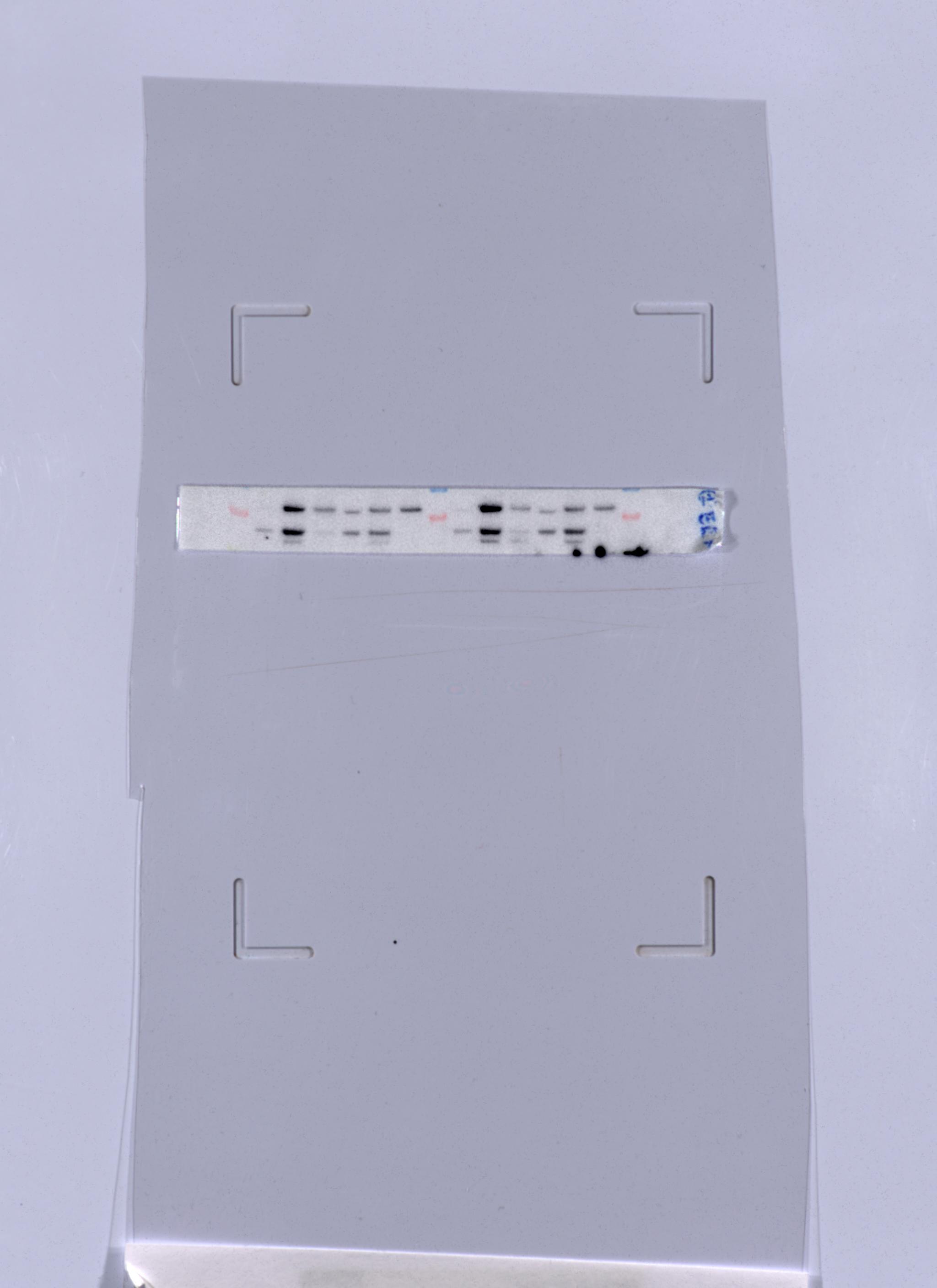


**
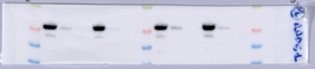
GAPDH 38 kDa**

2 3 4 5 6 7

**
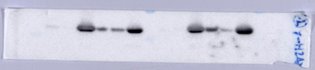
Histone 3 17 kDa**

2 3 4 5 6 7

### LCC9 cells

#### Replicate number 1 (Shown in Figure 5E)

Membrane I (Immunoblotting: CDK12, pMED1, ER, LMNB2, GAPDH, Histone 3).

**Ponceau staining**

8 9 10 11 12 13 14 15

|  | Replicate n1 |
| --- | --- |
| Lane n | Sample ID |
| 8 | Molecular marker (260-15 kDa) |
| 9 | LCC9 DMSO – Cyt. (n1) |
| 10 | LCC9 DMSO – Nucl. (n1) |
| 11 | LCC9 DMSO – Chrom. (n1) |
| 12 | LCC9 CT7116 – Cyt. (n1) |
| 13 | LCC9 CT7116 – Nucl. (n1) |
| 14 | LCC9 CT7116 – Chrom. (n1) |
| 15 | Molecular marker (260-15 kDa) |


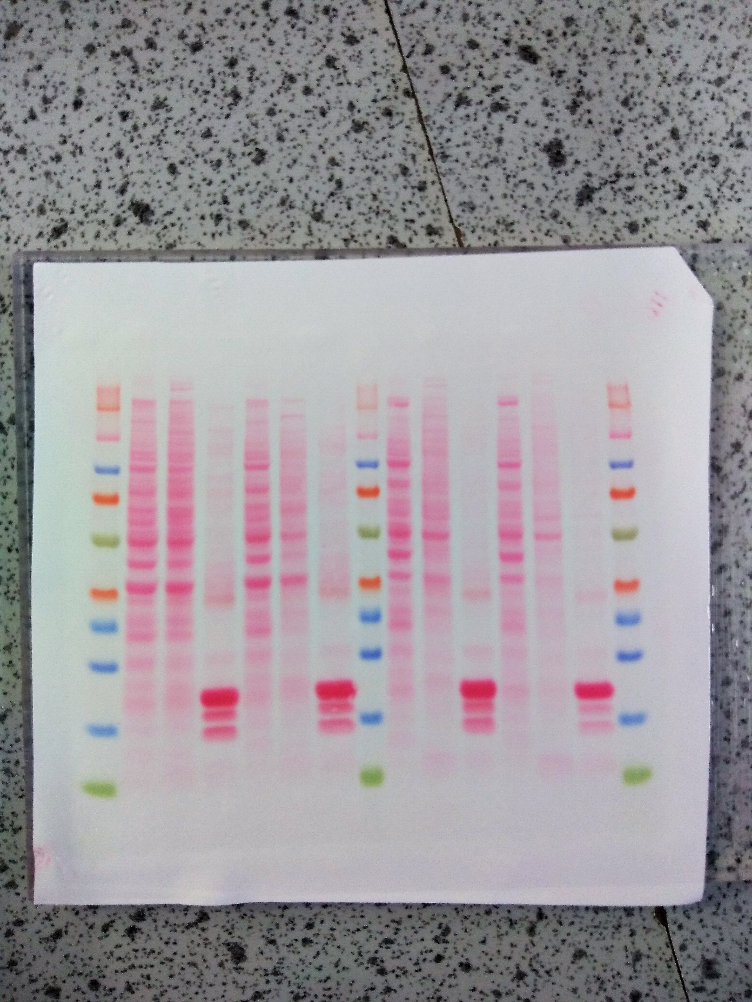


**CDK12 205 kDa**


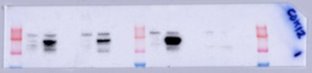


9 10 11 12 13 14

**pMED1 ~240 kDa**


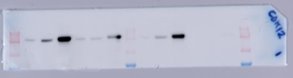


9 10 11 12 13 14

**ER 60 kDa**


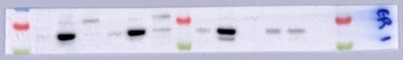


9 10 11 12 13 14

**LMNB2 68 kDa**


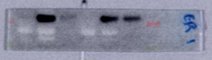


9 10 11 12 13 14

**
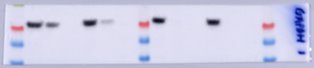
GAPDH** **38** **kDa**

9 10 11 12 13 14

**Histone 3 17 kDa**


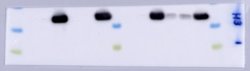


9 10 11 12 13 14

Membrane II (Immunoblotting: MED1, LMNB2, GAPDH, Histone 3).

**Ponceau staining**

|  | Replicate n1 |
| --- | --- |
| Lane n | Sample ID |
| 8 | Molecular marker (260-15 kDa) |
| 9 | LCC9 DMSO – Cyt. (n1) |
| 10 | LCC9 DMSO – Nucl. (n1) |
| 11 | LCC9 DMSO – Chrom. (n1) |
| 12 | LCC9 CT7116 – Cyt. (n1) |
| 13 | LCC9 CT7116 – Nucl. (n1) |
| 14 | LCC9 CT7116 – Chrom. (n1) |
| 15 | Molecular marker (260-15 kDa) |


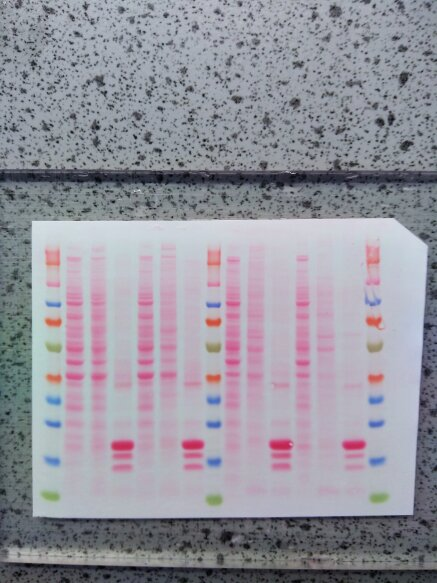


8 9 10 11 12 13 14 15

**
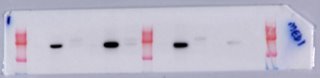
MED1 220 kDa**

9 10 11 12 13 14


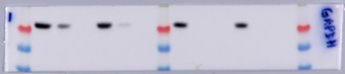
**GAPDH 38 kDa**

9 10 11 12 13 14

**LMNB2 68 kDa**


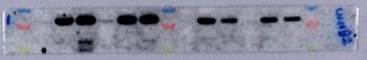


9 10 11 12 13 14

**Histone 3 17 kDa**


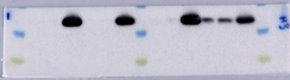


9 10 11 12 13 14

#### Replicate number 2

Membrane I (Immunoblotting: CDK12, pMED1, ER, LMNB2, GAPDH, Histone 3 – lanes 2 to 7; MED1, LMNB2, GAPDH, Histone 3 – lanes 9 to 14).

**Ponceau staining**

1 2 3 4 5 6 7 8 9 10 11 12 13 14 15

|  | Replicate n2 |
| --- | --- |
| Lane n | Sample ID |
| 1 | Molecular marker (260-15 kDa) |
| 2 | LCC9 DMSO – Cyt. (n2) |
| 3 | LCC9 DMSO – Nucl. (n2) |
| 4 | LCC9 DMSO – Chrom. (n2) |
| 5 | LCC9 CT7116 – Cyt. (n2) |
| 6 | LCC9 CT7116 – Nucl. (n2) |
| 7 | LCC9 CT7116 – Chrom. (n2) |
| 8 | Molecular marker (260-15 kDa) |
| 9 | LCC9 DMSO – Cyt. (n2) |
| 10 | LCC9 DMSO – Nucl. (n2) |
| 11 | LCC9 DMSO – Chrom. (n2) |
| 12 | LCC9 CT7116 – Cyt. (n2) |
| 13 | LCC9 CT7116 – Nucl. (n2) |
| 14 | LCC9 CT7116 – Chrom. (n2) |
| 15 | Molecular marker (260-15 kDa) |


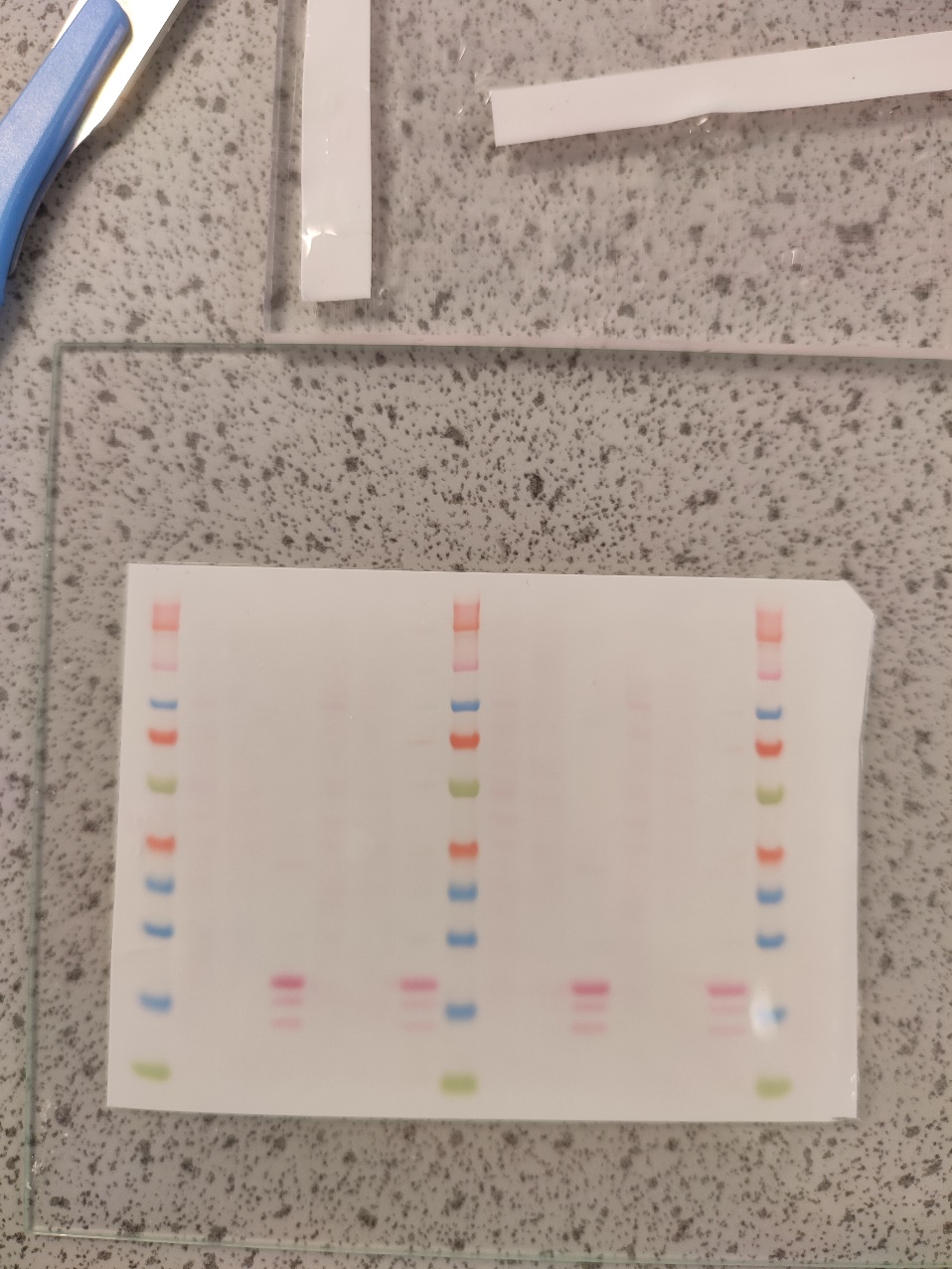


**
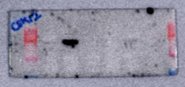
CDK12 205 kDa**

2 3 4 5 6 7

**
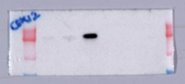
pMED1 ~240 kDa**

2 3 4 5 6 7

**MED1 220 kDa**

9 10 11 12 13 14

**ER 60 kDa**

2 3 4 5 6 7

**LMNB2 68 kDa**

2 3 4 5 6 7 9 10 11 12 13 14

**GAPDH 38 kDa**

2 3 4 5 6 7 9 10 11 12 13 14

**Histone 3 17kDa**

2 3 4 5 6 7 9 10 11 12 13 14

#### Replicate number 3

Membrane I (Immunoblotting: CDK12, pMED1, ER, LMNB2, GAPDH, Histone 3).

|  | Replicate n3 |
| --- | --- |
| Lane n | Sample ID |
| 8 | Molecular marker (260-15 kDa) |
| 9 | LCC9 DMSO – Cyt. (n3) |
| 10 | LCC9 DMSO – Nucl. (n3) |
| 11 | LCC9 DMSO – Chrom. (n3) |
| 12 | LCC9 CT7116 – Cyt. (n3) |
| 13 | LCC9 CT7116 – Nucl. (n3) |
| 14 | LCC9 CT7116 – Chrom. (n3) |
| 15 | Molecular marker (260-15 kDa) |

**Ponceau staining**

8 9 10 11 1213 14 15

**CDK12 205 kDa**

9 10 11 12 13 14

**pMED1 ~240 kDa**

9 10 11 12 13 14

**ER 60 kDa**

9 10 11 12 13 14

**LMNB2 68 kDa**

9 10 11 12 13 14

**GAPDH 38 kDa**

9 10 11 12 13 14

**Histone 3 17 kDa**

9 10 11 12 13 14

Membrane II (Immunoblotting: MED1, LMNB2, GAPDH, Histone 3).

**Ponceau staining**

|  | Replicate n3 |
| --- | --- |
| Lane n | Sample ID |
| 8 | Molecular marker (260-15 kDa) |
| 9 | LCC9 DMSO – Cyt. (n3) |
| 10 | LCC9 DMSO – Nucl. (n3) |
| 11 | LCC9 DMSO – Chrom. (n3) |
| 12 | LCC9 CT7116 – Cyt. (n3) |
| 13 | LCC9 CT7116 – Nucl. (n3) |
| 14 | LCC9 CT7116 – Chrom. (n3) |
| 15 | Molecular marker (260-15 kDa) |

8 9 10 11 12 13 14 15

**MED1 220 kDa**

9 10 11 12 13 14

**GAPDH 38 kDa**

9 10 11 12 13 14

**LMNB2 68 kDa**

9 10 11 12 13 14

**Histone 3 17 kDa**

9 10 11 12 13 14

### LY2 bone cells

#### Replicate number 1 (shown in Figure 5E)

Membrane I (Immunoblotting: CDK12, pMED1, ER, LMNB2, GAPDH, Histone 3).

**Ponceau staining**

1 2 3 4 5 6 7 8

|  | Replicate n1 |
| --- | --- |
| Lane n | Sample ID |
| 1 | Molecular marker (260-15 kDa) |
| 2 | LY2 bone DMSO – Cyt. (n1) |
| 3 | LY2 bone DMSO – Nucl. (n1) |
| 4 | LY2 bone DMSO – Chrom. (n1) |
| 5 | LY2 bone CT7116 – Cyt. (n1) |
| 6 | LY2 bone CT7116 – Nucl. (n1) |
| 7 | LY2 bone CT7116 – Chrom. (n1) |
| 8 | Molecular marker (260-15 kDa) |

**CDK12 205 kDa**

2 3 4 5 6 7

**pMED1 ~240 kDa**

2 3 4 5 6 7

**ER 60 kDa**

2 3 4 5 6 7

**LMNB2 68 kDa**

2 3 4 5 6 7

**GAPDH 38 kDa**

2 3 4 5 6 7

**Histone 3 17 kDa**

2 3 4 5 6 7

Membrane II (Immunoblotting: MED1, LMNB2, GAPDH, Histone 3).

**Ponceau staining**

|  | Replicate n1 |
| --- | --- |
| Lane n | Sample ID |
| 8 | Molecular marker (260-15 kDa) |
| 9 | LY2 bone DMSO – Cyt. (n1) |
| 10 | LY2 bone DMSO – Nucl. (n1) |
| 11 | LY2 bone DMSO – Chrom. (n1) |
| 12 | LY2 bone CT7116 – Cyt. (n1) |
| 13 | LY2 bone CT7116 – Nucl. (n1) |
| 14 | LY2 bone CT7116 – Chrom. (n1) |
| 15 | Molecular marker (260-15 kDa) |

8 9 10 11 12 13 14 15

**MED1 220 kDa**

9 10 11 12 13 14

**GAPDH 38 kDa**

9 10 11 12 13 14

**LMNB2 68 kDa**

9 10 11 12 13 14

**Histone 3 17 kDa**

9 10 11 12 13 14

#### Replicate number 2

Membrane I (Immunoblotting: pMED1, ER, LMNB2, GAPDH, Histone 3).

**Ponceau staining**

1 2 3 4 5 6 7 8

|  | Replicate n2 |
| --- | --- |
| Lane n | Sample ID |
| 1 | Molecular marker (260-15 kDa) |
| 2 | LY2 bone DMSO – Cyt. (n2) |
| 3 | LY2 bone DMSO – Nucl. (n2) |
| 4 | LY2 bone DMSO – Chrom. (n2) |
| 5 | LY2 bone CT7116 – Cyt. (n2) |
| 6 | LY2 bone CT7116 – Nucl. (n2) |
| 7 | LY2 bone CT7116 – Chrom. (n2) |
| 8 | Molecular marker (260-15 kDa) |

**pMED1 ~240 kDa**

2 3 4 5 6 7

**ER 60 kDa**

2 3 4 5 6 7

**LMNB2 68 kDa**

2 3 4 5 6 7

**GAPDH 38 kDa**

2 3 4 5 6 7

**Histone 3 17 kDa**

2 3 4 5 6 7

Membrane II (Immunoblotting: MED1, CDK12, LMNB2, GAPDH, Histone 3).

8 9 10 11 12 13 14 15

**Ponceau staining**

|  | Replicate n2 |
| --- | --- |
| Lane n | Sample ID |
| 8 | Molecular marker (260-15 kDa) |
| 9 | LY2 bone DMSO – Cyt. (n2) |
| 10 | LY2 bone DMSO – Nucl. (n2) |
| 11 | LY2 bone DMSO – Chrom. (n2) |
| 12 | LY2 bone CT7116 – Cyt. (n2) |
| 13 | LY2 bone CT7116 – Nucl. (n2) |
| 14 | LY2 bone CT7116 – Chrom. (n2) |
| 15 | Molecular marker (260-15 kDa) |

9 10 11 12 13 14

**MED1 220 kDa**

9 10 11 12 13 14

**CDK12 205 kDa**

**LMNB2 68 kDa**

9 10 11 12 13 14

**GAPDH 38 kDa**

9 10 11 12 13 14

**Histone 3 17 kDa**

9 10 11 12 13 14

#### Replicate number 3

Membrane I (Immunoblotting: CDK12, MED1, pMED1, ER, LMNB2, GAPDH, Histone 3).

**Ponceau staining**

|  | Replicate n3 |
| --- | --- |
| Lane n | Sample ID |
| 8 | Molecular marker (260-15 kDa) |
| 9 | LY2 bone DMSO – Cyt. (n3) |
| 10 | LY2 bone DMSO – Nucl. (n3) |
| 11 | LY2 bone DMSO – Chrom. (n3) |
| 12 | LY2 bone CT7116 – Cyt. (n3) |
| 13 | LY2 bone CT7116 – Nucl. (n3) |
| 14 | LY2 bone CT7116 – Chrom. (n3) |
| 15 | Molecular marker (260-15 kDa) |

8 9 10 11 12 13 14 15

**CDK12 205 kDa**

9 10 11 12 13 14

**MED1 220 kDa**

9 10 11 12 13 14

9 10 11 12 13 14

**pMED1 ~240 kDa**

**ER 60 kDa**

9 10 11 12 13 14

**LMNB2 68 kDa**

9 10 11 12 13 14

**GAPDH 38kDa**

9 10 11 12 13 14

9 10 11 12 13 14

**Histone 3 17 kDa**

### T347 cells

#### Replicate number 1 (shown in Figure 5E)

Membrane I (Immunoblotting: CDK12, pMED1, ER, LMNB2, GAPDH, Histone 3).

**Ponceau staining**

|  | Replicate n1 |
| --- | --- |
| Lane n | Sample ID |
| 1 | Molecular marker (260-15 kDa) |
| 2 | T347 DMSO – Cyt. (n1) |
| 3 | T347 DMSO – Nucl. (n1) |
| 4 | T347 DMSO – Chrom. (n1) |
| 5 | T347 CT7116 – Cyt. (n1) |
| 6 | T347 CT7116 – Nucl. (n1) |
| 7 | T347 CT7116 – Chrom. (n1) |
| 8 | Molecular marker (260-15 kDa) |

1 2 3 4 5 6 7 8

**CDK12 205 kDa**

2 3 4 5 6 7

**pMED1 ~240 kDa**

2 3 4 5 6 7

**ER 60 kDa**

2 3 4 5 6 7

**LMNB2 68 kDa**

2 3 4 5 6 7

**GAPDH 38 kDa**

2 3 4 5 6 7

**Histone 3 17 kDa**

2 3 4 5 6 7

Membrane II (Immunoblotting: MED1, LMNB2, GAPDH, Histone 3).

**Ponceau staining**

|  | Replicate n1 |
| --- | --- |
| Lane n | Sample ID |
| 1 | Molecular marker (260-15 kDa) |
| 2 | T347 DMSO – Cyt. (n1) |
| 3 | T347 DMSO – Nucl. (n1) |
| 4 | T347 DMSO – Chrom. (n1) |
| 5 | T347 CT7116 – Cyt. (n1) |
| 6 | T347 CT7116 – Nucl. (n1) |
| 7 | T347 CT7116 – Chrom. (n1) |
| 8 | Molecular marker (260-15 kDa) |

1 2 3 4 5 6 7 8

**MED1 220 kDa**

2 3 4 5 6 7

**LMNB2 68 kDa**

2 3 4 5 6 7

**GAPDH 38 kDa**

2 3 4 5 6 7

**Histone 3 17 kDa**

2 3 4 5 6 7

#### Replicate number 2

Membrane I (Immunoblotting: CDK12, pMED1, ER, LMNB2, GAPDH, Histone 3).

**Ponceau staining**

|  | Replicate n2 |
| --- | --- |
| Lane n | Sample ID |
| 1 | Molecular marker (260-15 kDa) |
| 2  1 2 3 4 5 6 7 8 | T347 DMSO – Cyt. (n2) |
| 3 | T347 DMSO – Nucl. (n2) |
| 4 | T347 DMSO – Chrom. (n2) |
| 5 | T347 CT7116 – Cyt. (n2) |
| 6 | T347 CT7116 – Nucl. (n2) |
| 7 | T347 CT7116 – Chrom. (n2) |
| 8 | Molecular marker (260-15 kDa) |

**CDK12 205 kDa**

2 3 4 5 6 7

**pMED1 ~240 kDa**

2 3 4 5 6 7

**ER 60 kDa**

2 3 4 5 6 7

**LMNB2 68 kDa**

2 3 4 5 6 7

**GAPDH 38 kDa**

2 3 4 5 6 7

**Histone 3 17 kDa**

2 3 4 5 6 7

Membrane II (Immunoblotting: MED1, LMNB2, GAPDH, Histone 3).

**Ponceau staining**

|  | Replicate n2 |
| --- | --- |
| Lane n | Sample ID |
| 1 | Molecular marker (260-15 kDa) |
| 2 | T347 DMSO – Cyt. (n2) |
| 3 | T347 DMSO – Nucl. (n2) |
| 4 | T347 DMSO – Chrom. (n2) |
| 5 | T347 CT7116 – Cyt. (n2) |
| 6 | T347 CT7116 – Nucl. (n2) |
| 7 | T347 CT7116 – Chrom. (n2) |
| 8 | Molecular marker (260-15 kDa) |

1 2 3 4 5 6 7 8

**MED1 220 kDa**

2 3 4 5 6 7

**GAPDH 38 kDa**

2 3 4 5 6 7

**LMNB2 68 kDa**

2 3 4 5 6 7

**Histone 3 17 kDa**

2 3 4 5 6 7

#### Replicate number 3

Membrane I (Immunoblotting: CDK12, pMED1, ER, LMNB2, GAPDH, Histone 3).

**Ponceau staining**

|  | Replicate n3 |
| --- | --- |
| Lane n | Sample ID |
| 1 | Molecular marker (260-15 kDa) |
| 2 | T347 DMSO – Cyt. (n3) |
| 3 | T347 DMSO – Nucl. (n3) |
| 4 | T347 DMSO – Chrom. (n3) |
| 5 | T347 CT7116 – Cyt. (n3) |
| 6 | T347 CT7116 – Nucl. (n3) |
| 7 | T347 CT7116 – Chrom. (n3) |
| 8 | Molecular marker (260-15 kDa) |

1 2 3 4 5 6 7 8

**CDK12 205 kDa**

2 3 4 5 6 7

**pMED1 ~240 kDa**

2 3 4 5 6 7

2 3 4 5 6 7

**ER 60 kDa**

**LMNB2 68 kDa**

2 3 4 5 6 7

**GAPDH 38 kDa**

2 3 4 5 6 7

**Histone 3 17 kDa**

2 3 4 5 6 7

Membrane II (Immunoblotting: MED1, LMNB2, GAPDH, Histone 3).

**Ponceau staining**

|  | Replicate n3 |
| --- | --- |
| Lane n | Sample ID |
| 1 | Molecular marker (260-15 kDa) |
| 2 | T347 DMSO – Cyt. (n3) |
| 3 | T347 DMSO – Nucl. (n3) |
| 4 | T347 DMSO – Chrom. (n3) |
| 5 | T347 CT7116 – Cyt. (n3) |
| 6 | T347 CT7116 – Nucl. (n3) |
| 7 | T347 CT7116 – Chrom. (n3) |
| 8 | Molecular marker (260-15 kDa) |

1 2 3 4 5 6 7 8

**MED1 220 kDa**

2 3 4 5 6 7

**GAPDH 38 kDa**

2 3 4 5 6 7

**LMNB2 68kDa**

2 3 4 5 6 7

**Histone 3 17 kDa**

2 3 4 5 6 7

## Supplementary Material 6. Uncropped western blot membranes in Figure 5F.

**Cleaved-PARP (cPARP) protein expression following CT7116 treatment (0.3 µmol/L, 24 hours) in LY2, LCC9, LY2 bone and T347 cells.** cPARP + loading controls for cytoplasmic (GAPDH) and nuclear (LMNB2) protein fractions.

### LY2 cells

#### Replicate number 1 (shown in Figure 5F)

**Ponceau staining**

|  | Replicate n1 |
| --- | --- |
| Lane n | Sample ID |
| 1 | Molecular marker (260-15 kDa) |
| 2 | LY2 DMSO – Cyt. (n1) |
| 3 | LY2 DMSO – Nucl. (n1) |
| 4 | LY2 CT7116 – Cyt. (n1) |
| 5 | LY2 CT7116 – Nucl. (n1) |
| 6 | Molecular marker (260-15 kDa) |

1 2 3 4 5 6

**cPARP 89 kDa**

2 3 4 5

**LMNB2 68 kDa**

2 3 4 5

**GAPDH 38 kDa**

2 3 4 5

#### Replicate number 2

**Ponceau staining**

|  | Replicate n2 |
| --- | --- |
| Lane n | Sample ID |
| 1 | Molecular marker (260-15 kDa) |
| 2 | LY2 DMSO – Cyt. (n2) |
| 3 | LY2 DMSO – Nucl. (n2) |
| 4 | LY2 CT7116 – Cyt. (n2) |
| 5 | LY2 CT7116 – Nucl. (n2) |
| 6 | Molecular marker (260-15 kDa) |

1 2 3 4 5 6

**cPARP 89 kDa**

2 3 4 5

**LMNB2 68 kDa**

2 3 4 5

**GAPDH 38 kDa**

2 3 4 5

#### Replicate number 3

**Ponceau staining**

|  | Replicate n3 |
| --- | --- |
| Lane n | Sample ID |
| 1 | Molecular marker (260-15 kDa) |
| 2 | LY2 DMSO – Cyt. (n3) |
| 3 | LY2 DMSO – Nucl. (n3) |
| 4 | LY2 CT7116 – Cyt. (n3) |
| 5 | LY2 CT7116 – Nucl. (n3) |
| 6 | Molecular marker (260-15 kDa) |

1 2 3 4 5 6

**cPARP 89 kDa**

2 3 4 5

**LMNB2 68 kDa**

2 3 4 5

**GAPDH 38 kDa**

2 3 4 5

### LCC9 cells

#### Replicate number 1 (shown in Figure 5F)

6 7 8 9 10 11

**Ponceau staining**

|  | Replicate n1 |
| --- | --- |
| Lane n | Sample ID |
| 6 | Molecular marker (260-15 kDa) |
| 7 | LCC9 DMSO – Cyt. (n1) |
| 8 | LCC9 DMSO – Nucl. (n1) |
| 9 | LCC9 CT7116 – Cyt. (n1) |
| 10 | LCC9 CT7116 – Nucl. (n1) |
| 11 | Molecular marker (260-15 kDa) |

**cPARP 89 kDa**

7 8 9 10

**LMNB2 68 kDa**

7 8 9 10

**GAPDH 38 kDa**

7 8 9 10

#### Replicate number 2

**Ponceau staining**

|  | Replicate n2 |
| --- | --- |
| Lane n | Sample ID |
| 6 | Molecular marker (260-15 kDa) |
| 7 | LCC9 DMSO – Cyt. (n2) |
| 8 | LCC9 DMSO – Nucl. (n2) |
| 9 | LCC9 CT7116 – Cyt. (n2) |
| 10 | LCC9 CT7116 – Nucl. (n2) |
| 11 | Molecular marker (260-15 kDa) |

6 7 8 9 10 11

**cPARP 89 kDa**

7 8 9 10

**LMNB2 68 kDa**

7 8 9 10

**GAPDH 38 kDa**

7 8 9 10

#### Replicate number 3

6 7 8 9 10 11

**Ponceau staining**

|  | Replicate n3 |
| --- | --- |
| Lane n | Sample ID |
| 6 | Molecular marker (260-15 kDa) |
| 7 | LCC9 DMSO – Cyt. (n3) |
| 8 | LCC9 DMSO – Nucl. (n3) |
| 9 | LCC9 CT7116 – Cyt. (n3) |
| 10 | LCC9 CT7116 – Nucl. (n3) |
| 11 | Molecular marker (260-15 kDa) |

**cPARP 89 kDa**

7 8 9 10

**LMNB2 68 kDa**

7 8 9 10

**GAPDH 38 kDa**

7 8 9 10

### LY2 bone cells

#### Replicate number 1 (shown in Figure 5F)

**Ponceau staining**

|  | Replicate n1 |
| --- | --- |
| Lane n | Sample ID |
| 6 | Molecular marker (260-15 kDa) |
| 7 | LY2 bone DMSO – Cyt. (n1) |
| 8 | LY2 bone DMSO – Nucl. (n1) |
| 9 | LY2 bone CT7116 – Cyt. (n1) |
| 10 | LY2 bone CT7116 – Nucl. (n1) |
| 11 | Molecular marker (260-15 kDa) |

6 7 8 9 10 11

**cPARP 89 kDa**

7 8 9 10

**LMNB2 68 kDa**

7 8 9 10

**GAPDH 38 kDa**

7 8 9 10

#### Replicate number 2

**Ponceau staining**

|  | Replicate n2 |
| --- | --- |
| Lane n | Sample ID |
| 6 | Molecular marker (260-15 kDa) |
| 7 | LY2 bone DMSO – Cyt. (n2) |
| 8 | LY2 bone DMSO – Nucl. (n2) |
| 9 | LY2 bone CT7116 – Cyt. (n2) |
| 10 | LY2 bone CT7116 – Nucl. (n2) |
| 11 | Molecular marker (260-15 kDa) |

6 7 8 9 10 11

**cPARP 89 kDa**

7 8 9 10

**LMNB2 68 kDa**

7 8 9 10

**GAPDH 38 kDa**

7 8 9 10

#### Replicate number 3

**Ponceau staining**

6 7 8 9 10 11

|  | Replicate n3 |
| --- | --- |
| Lane n | Sample ID |
| 6 | Molecular marker (260-15 kDa) |
| 7 | LY2 bone DMSO – Cyt. (n3) |
| 8 | LY2 bone DMSO – Nucl. (n3) |
| 9 | LY2 bone CT7116 – Cyt. (n3) |
| 10 | LY2 bone CT7116 – Nucl. (n3) |
| 11 | Molecular marker (260-15 kDa) |

**cPARP 89 kDa**

7 8 9 10

**LMNB2 68 kDa**

7 8 9 10

**GAPDH 38 kDa**

7 8 9 10

### T347 cells

#### Replicate number 1 (shown in Figure 5F)

**Ponceau staining**

|  | Replicate n1 |
| --- | --- |
| Lane n | Sample ID |
| 1 | Molecular marker (260-15 kDa) |
| 2 | T347 DMSO – Cyt. (n1) |
| 3 | T347 DMSO – Nucl. (n1) |
| 4 | T347 CT7116 – Cyt. (n1) |
| 5 | T347 CT7116 – Nucl. (n1) |
| 6 | Molecular marker (260-15 kDa) |

1 2 3 4 5 6

**cPARP 89 kDa**

2 3 4 5

**LMNB2 68 kDa**

2 3 4 5

**GAPDH 38 kDa**

2 3 4 5

#### Replicate number 2

**Ponceau staining**

|  | Replicate n2 |
| --- | --- |
| Lane n | Sample ID |
| 1 | Molecular marker (260-15 kDa) |
| 2 | T347 DMSO – Cyt. (n2) |
| 3 | T347 DMSO – Nucl. (n2) |
| 4 | T347 CT7116 – Cyt. (n2) |
| 5 | T347 CT7116 – Nucl. (n2) |
| 6 | Molecular marker (260-15 kDa) |

1 2 3 4 5 6

**cPARP 89 kDa**

2 3 4 5

**LMNB2 68 kDa**

2 3 4 5

**GAPDH 38 kDa**

2 3 4 5

#### Replicate number 3

**Ponceau staining**

1 2 3 4 5 6

|  | Replicate n3 |
| --- | --- |
| Lane n | Sample ID |
| 1 | Molecular marker (260-15 kDa) |
| 2 | T347 DMSO – Cyt. (n3) |
| 3 | T347 DMSO – Nucl. (n3) |
| 4 | T347 CT7116 – Cyt. (n3) |
| 5 | T347 CT7116 – Nucl. (n3) |
| 6 | Molecular marker (260-15 kDa) |

**cPARP 89 kDa**

2 3 4 5

**LMNB2 68 kDa**

2 3 4 5

**GAPDH 38 kDa**

2 3 4 5

## Supplementary Material 7. Uncropped western blot membranes in Supplementary Figure 3E.

**Apoptosis activation detected by cleaved-PARP (cPARP) protein expression following CDK12 knockdown (siRNA, 48 hours) in LY2 cells.** cPARP + loading controls for cytoplasmic (GAPDH) and nuclear (LMNB2) protein fractions.

### LY2 cells

#### Replicate number 1

**Ponceau staining**

|  | Replicate n1 |
| --- | --- |
| Lane n | Sample ID |
| 1 | Molecular marker (260-15 kDa) |
| 2 | LY2 siCtrl – Cyt. (n1) |
| 3 | LY2 siCtrl – Nucl. (n1) |
| 4 | LY2 siCDK12 – Cyt. (n1) |
| 5 | LY2 siCDK12 – Nucl. (n1) |
| 6 | Molecular marker (260-15 kDa) |

1 2 3 4 5 6

**CDK12 205 kDa**

2 3 4 5

**cPARP 89 kDa**

2 3 4 5

**LMNB2 68 kDa**

2 3 4 5

**GAPDH 38kDa**

2 3 4 5

#### Replicate number 2 and number 3 (shown in Supplementary Figure 3E)

|  | Replicate n2 and n3 |
| --- | --- |
| Lane n | Sample ID |
| 1 | Molecular marker (260-15 kDa) |
| 2 | LY2 siCtrl – Cyt. (n2) |
| 3 | LY2 siCtrl – Nucl. (n2) |
| 4 | LY2 siCDK12 – Cyt. (n2) |
| 5 | LY2 siCDK12 – Nucl. (n2) |
| 6 | Molecular marker (260-15 kDa) |
| 7 | LY2 siCtrl – Cyt. (n3) |
| 8 | LY2 siCtrl – Nucl. (n3) |
| 9 | LY2 siCDK12 – Cyt. (n3) |
| 10 | LY2 siCDK12 – Nucl. (n3) |
| 11 | Molecular marker (260-15 kDa) |

**Ponceau staining**

1 2 3 4 5 6 7 8 9 10 11

**CDK12 205 kDa**

2 3 4 5 7 8 9 10

**cPARP 89 kDa**

2 3 4 5 7 8 9 10

**LMNB2 68 kDa**

2 3 4 5 7 8 9 10

**GAPDH 38 kDa**

2 3 4 5 7 8 9 10

## Supplementary Material 8. Uncropped western blot membranes in Supplementary Figure 7A.

**Protein expression following CT7116 treatment (0.3 µmol/L, 24 hours) in T47D cells.** CDK12, MED1, pMED1, ER + loading controls for cytoplasmic (GAPDH), nuclear (LMNB2), and chromatin-bound (Histone 3) protein fractions.

### T47D cells

#### Replicate number 1

Membrane I (Immunoblotting: CDK12, pMED1, ER, LMNA, GAPDH, Histone 3).

**Ponceau staining**

1 2 3 4 5 6 7 8 9

|  | Replicate n1 |
| --- | --- |
| Lane n | Sample ID |
| 1 | Molecular marker (260-15 kDa) |
| 2 | T47D DMSO - Cyt. (n1) |
| 3 | T47D DMSO - Nucl. (n1) |
| 4 | T47D DMSO - Chrom. (n1) |
| 5 | T47D CT7116 - Cyt. (n1) |
| 6 | T47D CT7116 - Nucl. (n1) |
| 7 | T47D CT7116 - Chrom. (n1) |
| 8 | Molecular marker (260-15 kDa) |
| 9 | Molecular marker (260-15 kDa) |

**CDK12 205 kDa**

1 2 3 4 5 6 7 8 9

**pMED1 ~240 kD**

1 2 3 4 5 6 7 8 9

**ER ~65 kDa**

1 2 3 4 5 6 7 8 9

**LMNA ~85 kDa**

1 2 3 4 5 6 7 8 9

**GAPDH 38 kDa**

1 2 3 4 5 6 7 8 9

**H3 17 kDa**

Membrane II (Immunoblotting: MED1, LMNA, GAPDH, Histone 3).

**Ponceau staining**

|  | Replicate n1 |
| --- | --- |
| Lane n | Sample ID |
| 1 | Molecular marker (260-15 kDa) |
| 2 | T47D DMSO - Cyt. (n1) |
| 3 | T47D DMSO - Nucl. (n1) |
| 4 | T47D DMSO - Chrom. (n1) |
| 5 | T47D CT7116 - Cyt. (n1) |
| 6 | T47D CT7116 - Nucl. (n1) |
| 7 | T47D CT7116 - Chrom. (n1) |
| 8 | Molecular marker (260-15 kDa) |
| 9 | Molecular marker (260-15 kDa) |

1 2 3 4 5 6 7 8. 9

**MED1 220 kDa**

1 2 3 4 5 6 7 8. 9

**LMNA ~85 kDa**

**GAPDH 38 kDa**

**H3 17 kDa**

1 2 3 4 5 6 7 8. 9

#### Replicate number 2 (shown in Supplementary Figure 7A)

Membrane I (Immunoblotting: CDK12, ER + LMNA, GAPDH, Histone 3).

**Ponceau staining**

1 2 3 4 5 6 7 8 9

|  | Replicate n2 |
| --- | --- |
| Lane n | Sample ID |
| 1 | Molecular marker (260-15 kDa) |
| 2 | Molecular marker (260-15 kDa) |
| 3 | T47D DMSO - Cyt. (n2) |
| 4 | T47D DMSO - Nucl. (n2) |
| 5 | T47D DMSO - Chrom. (n2) |
| 6 | T47D CT7116 - Cyt. (n2) |
| 7 | T47D CT7116 - Nucl. (n2) |
| 8 | T47D CT7116 - Chrom. (n2) |
| 9 | Molecular marker (260-15 kDa) |

**CDK12 205 kDa**

1 2 3 4 5 6 7 8 9

**pMED1 ~240 kD**

1 2 3 4 5 6 7 8 9

**ER ~65 kDa**

1 2 3 4 5 6 7 8 9

**LMNA ~85 kDa**

1 2 3 4 5 6 7 8 9

**GAPDH 38 kDa**

1 2 3 4 5 6 7 8 9

**H3 17 kDa**

1 2 3 4 5 6 7 8 9

Membrane II (Immunoblotting: MED1, LMNA, GAPDH, Histone 3).

**Ponceau staining**

1 2 3 4 5 6 7 8 9

|  | Replicate n2 |
| --- | --- |
| Lane n | Sample ID |
| 1 | Molecular marker (260-15 kDa) |
| 2 | Molecular marker (260-15 kDa) |
| 3 | T47D DMSO - Cyt. (n2) |
| 4 | T47D DMSO - Nucl. (n2) |
| 5 | T47D DMSO - Chrom. (n2) |
| 6 | T47D CT7116 - Cyt. (n2) |
| 7 | T47D CT7116 - Nucl. (n2) |
| 8 | T47D CT7116 - Chrom. (n2) |
| 9 | Molecular marker (260-15 kDa) |

**MED1 220 kDa**

1 2 3 4 5 6 7 8 9

**LMNA ~85 kDa**

1 2 3 4 5 6 7 8 9

**GAPDH (38 kDa)**

1 2 3 4 5 6 7 8 9

**H3 17 kDa**

1 2 3 4 5 6 7 8 9

#### Replicate number 3

Membrane I (Immunoblotting: CDK12, ER, LMNA, GAPDH, Histone 3).

**Ponceau staining**

1 2 3 4 5 6 7 8

|  | Replicate n3 |
| --- | --- |
| Lane n | Sample ID |
| 1 | Molecular marker (260-15 kDa) |
| 2 | Molecular marker (260-15 kDa) |
| 3 | T47D DMSO - Cyt. (n3) |
| 4 | T47D DMSO - Nucl. (n3) |
| 5 | T47D DMSO - Chrom. (n3) |
| 6 | T47D CT7116 - Cyt. (n3) |
| 7 | T47D CT7116 - Nucl. (n3) |
| 8 | T47D CT7116 - Chrom. (n3) |
| 9 | Molecular marker (260-15 kDa) |

**CDK12 205 kDa**

1 2 3 4 5 6 7 8

**pMED1 ~240 kDa**

1 2 3 4 5 6 7 8

**ER ~65 kDa**

1 2 3 4 5 6 7 8

**LMNA ~85 kDa**

1 2 3 4 5 6 7 8

**GAPDH 38 kDa**

1 2 3 4 5 6 7 8

**H3 17 kDa**

1 2 3 4 5 6 7 8

Membrane II (Immunoblotting: MED1, LMNA, GAPDH, Histone 3).

**Ponceau staining**

|  | Replicate n3 |
| --- | --- |
| Lane n | Sample ID |
| 1 | Molecular marker (260-15 kDa) |
| 2 | Molecular marker (260-15 kDa) |
| 3 | T47D DMSO - Cyt. (n3) |
| 4 | T47D DMSO - Nucl. (n3) |
| 5 | T47D DMSO - Chrom. (n3) |
| 6 | T47D CT7116 - Cyt. (n3) |
| 7 | T47D CT7116 - Nucl. (n3) |
| 8 | T47D CT7116 - Chrom. (n3) |
| 9 | Molecular marker (260-15 kDa) |

1 2 3 4 5 6 7 8

**MED1 220 kDa**

1 2 3 4 5 6 7 8

**LMNA ~85 kDa**

1 2 3 4 5 6 7 8

**GAPDH 38 kDa**

1 2 3 4 5 6 7 8

**H3 17 kDa**

1 2 3 4 5 6 7 8

Supplementary Material 9. Uncropped western blot membranes in Supplementary Figure 7B.

**Protein expression following CT7116 treatment (0.3 µmol/L, 24 hours) in LY2, LCC9, LY2 bone, and T347 cells.** CDK13, CCNK, RNA PolII-CTD, and pSer2-CTD + loading controls for cytoplasmic (GAPDH), nuclear (LMNB2/LMNA) and chromatin-bound fractions (Histone 3).

### LY2 cells

#### Replicate number 1 (shown in Supplementary Figure 7A)

Membrane I (Immunoblotting: CDK13, pSer2 CTD, LMNB2, GAPDH, Histone 3).

**Ponceau staining**

1 2 3 4 5 6 7 8

|  | Replicate n1 |
| --- | --- |
| Lane n | Sample ID |
| 1 | Molecular marker (260-15 kDa) |
| 2 | LY2 DMSO - Cyt. (n1) |
| 3 | LY2 DMSO - Nucl. (n1) |
| 4 | LY2 DMSO - Chrom. (n1) |
| 5 | LY2 CT7116 - Cyt. (n1) |
| 6 | LY2 CT7116 - Nucl. (n1) |
| 7 | LY2 CT7116 - Chrom. (n1) |
| 8 | Molecular marker (260-15 kDa) |

**CDK13 170 kDa**

2 3 4 5 6 7

**pSer2 CTD 250 kDa**

2 3 4 5 6 7

**LMNB2 68 kDa**

2 3 4 5 6 7

**GAPDH 38 kDa**

2 3 4 5 6 7

**Histone 3 17 kDa**

2 3 4 5 6 7

Membrane II (Immunoblotting: RNA pol II CTD, CCNK, LMNB2, GAPDH, Histone 3).

**Ponceau staining**

1 2 3 4 5 6 7 8

|  | Replicate n1 |
| --- | --- |
| Lane n | Sample ID |
| 1 | Molecular marker (260-15 kDa) |
| 2 | LY2 DMSO - Cyt. (n1) |
| 3 | LY2 DMSO - Nucl. (n1) |
| 4 | LY2 DMSO - Chrom. (n1) |
| 5 | LY2 CT7116 - Cyt. (n1) |
| 6 | LY2 CT7116 - Nucl. (n1) |
| 7 | LY2 CT7116 - Chrom. (n1) |
| 8 | Molecular marker (260-15 kDa) |

**RNA pol II CTD 250 kDa**

2 3 4 5 6 7

**CCNK 70 kDa**

2 3 4 5 6 7

**LMNB2 68 kDa**

2 3 4 5 6 7

**GAPDH 38 kDa**

2 3 4 5 6 7

**Histone 3 17 kDa**

2 3 4 5 6 7

#### Replicate number 2

Membrane I (Immunoblotting: pSer2 CTD, CCNK, LMNA, GAPDH, Histone 3).

**Ponceau staining**

1 2 3 4 5 6 7 8

|  | Replicate n2 |
| --- | --- |
| Lane n | Sample ID |
| 1 | Molecular marker (260-15 kDa) |
| 2 | LY2 DMSO - Cyt. (n2) |
| 3 | LY2 DMSO - Nucl. (n2) |
| 4 | LY2 DMSO - Chrom. (n2) |
| 5 | LY2 CT7116 - Cyt. (n2) |
| 6 | LY2 CT7116 - Nucl. (n2) |
| 7 | LY2 CT7116 - Chrom. (n2) |
| 8 | Molecular marker (260-15 kDa) |

**pSer2 CTD 250 kDa**

2 3 4 5 6 7

**CCNK 70 kDa**

2 3 4 5 6 7

**LMNA ~85 kDa**

2 3 4 5 6 7

**GAPDH 38 kDa**

2 3 4 5 6 7

**Histone 3 17 kDa**

2 3 4 5 6 7

Membrane II (Immunoblotting: CDK13, RNA pol II CTD, LMNB2, GAPDH, Histone 3).

**Ponceau staining**

1 2 3 4 5 6 7 8

|  | Replicate n2 |
| --- | --- |
| Lane n | Sample ID |
| 1 | Molecular marker (260-15 kDa) |
| 2 | LY2 DMSO - Cyt. (n2) |
| 3 | LY2 DMSO - Nucl. (n2) |
| 4 | LY2 DMSO - Chrom. (n2) |
| 5 | LY2 CT7116 - Cyt. (n2) |
| 6 | LY2 CT7116 - Nucl. (n2) |
| 7 | LY2 CT7116 - Chrom. (n2) |
| 8 | Molecular marker (260-15 kDa) |

**CDK13 170 kDa**

2 3 4 5 6 7

**RNA pol II CTD 250 kDa**

2 3 4 5 6 7

**LMNB2 68 kDa**

2 3 4 5 6 7

**GAPDH 38 kDa**

2 3 4 5 6 7

**Histone 3 17 kDa**

2 3 4 5 6 7

#### Replicate number 3

Membrane I (Immunoblotting: pSer2 CTD, CCNK, LMNB2, GAPDH, Histone 3).

**Ponceau staining**

1 2 3 4 5 6 7 8

|  | Replicate n3 |
| --- | --- |
| Lane n | Sample ID |
| 1 | Molecular marker (260-15 kDa) |
| 2 | LY2 DMSO - Cyt. (n3) |
| 3 | LY2 DMSO - Nucl. (n3) |
| 4 | LY2 DMSO - Chrom. (n3) |
| 5 | LY2 CT7116 - Cyt. (n3) |
| 6 | LY2 CT7116 - Nucl. (n3) |
| 7 | LY2 CT7116 - Chrom. (n3) |
| 8 | Molecular marker (260-15 kDa) |

**pSer2 CTD 250 kDa**

2 3 4 5 6 7

**CCNK 70 kDa**

2 3 4 5 6 7

**LMNA ~85 kDa**

2 3 4 5 6 7

**GAPDH 38 kDa**

2 3 4 5 6 7

**Histone 3 17 kDa**

2 3 4 5 6 7

Membrane II (Immunoblotting: CDK13, RNA pol II CTD, LMNB2, GAPDH, Histone 3).

**Ponceau staining**

1 2 3 4 5 6 7 8

|  | Replicate n3 |
| --- | --- |
| Lane n | Sample ID |
| 1 | Molecular marker (260-15 kDa) |
| 2 | LY2 DMSO - Cyt. (n3) |
| 3 | LY2 DMSO - Nucl. (n3) |
| 4 | LY2 DMSO - Chrom. (n3) |
| 5 | LY2 CT7116 - Cyt. (n3) |
| 6 | LY2 CT7116 - Nucl. (n3) |
| 7 | LY2 CT7116 - Chrom. (n3) |
| 8 | Molecular marker (260-15 kDa) |

**CDK13 170 kDa**

2 3 4 5 6 7

**RNA pol II CTD 250 kDa**

2 3 4 5 6 7

**LMNA ~85 kDa**

2 3 4 5 6 7

**GAPDH 38 kDa**

2 3 4 5 6 7

**Histone 3 17 kDa**

2 3 4 5 6 7

### LCC9

#### Replicate number 1 (shown in Supplementary Figure 7A)

Membrane 1 (CDK13, pSer2 CTD, LMNB2, GAPDH, Histone 3) (lanes 9 to 14).

Ponceau staining

8 9 10 11 12 13 14 15

|  | Replicate n1 |
| --- | --- |
| Lane n | Sample ID |
| 8 | Molecular marker (260-15 kDa) |
| 9 | LCC9 DMSO - Cyt. (n1) |
| 10 | LCC9 DMSO - Nucl. (n1) |
| 11 | LCC9 DMSO - Chrom. (n1) |
| 12 | LCC9 CT7116 - Cyt. (n1) |
| 13 | LCC9 CT7116 - Nucl. (n1) |
| 14 | LCC9 CT7116 - Chrom. (n1) |
| 15 | Molecular marker (260-15 kDa) |

9 10 11 12 13 14

**CDK13 170 kDa**

**pSer2 CTD 250 kDa**

9 10 11 12 13 14

**LMNB2 68 kDa**

9 10 11 12 13 14

**GAPDH 38 kDa**

9 10 11 12 13 14

9 10 11 12 13 14

**Histone 3 17 kDa**

Membrane II (Immunoblotting: RNA pol II CTD, CCNK, LMNB2, GAPDH, Histone 3).

**Ponceau staining**

8 9 10 11 12 13 14 15

|  | Replicate n1 |
| --- | --- |
| Lane n | Sample ID |
| 8 | Molecular marker (260-15 kDa) |
| 9 | LCC9 DMSO - Cyt. (n1) |
| 10 | LCC9 DMSO - Nucl. (n1) |
| 11 | LCC9 DMSO - Chrom. (n1) |
| 12 | LCC9 CT7116 - Cyt. (n1) |
| 13 | LCC9 CT7116 - Nucl. (n1) |
| 14 | LCC9 CT7116 - Chrom. (n1) |
| 15 | Molecular marker (260-15 kDa) |

**RNA pol II CTD 250 kDa**

9 10 11 12 13 14

**CCNK 70 kDa**

9 10 11 12 13 14

**LMNB2 68 kDa**

9 10 11 12 13 14

**GAPDH 38 kDa**

9 10 11 12 13 14

**Histone 3 17 kDa**

9 10 11 12 13 14

#### Replicate number 2

Membrane I (Immunoblotting: pSer2 CTD, CCNK, LMNA, GAPDH, Histone 3).

**Ponceau staining**

8 9 10 11 12 13 14 15

|  | Replicate n2 |
| --- | --- |
| Lane n | Sample ID |
| 8 | Molecular marker (260-15 kDa) |
| 9 | LCC9 DMSO - Cyt. (n2) |
| 10 | LCC9 DMSO - Nucl. (n2) |
| 11 | LCC9 DMSO - Chrom. (n2) |
| 12 | LCC9 CT7116 - Cyt. (n2) |
| 13 | LCC9 CT7116 - Nucl. (n2) |
| 14 | LCC9 CT7116 - Chrom. (n2) |
| 15 | Molecular marker (260-15 kDa) |

**pSer2 CTD 250 kDa**

9 10 11 12 13 14

**CCNK 70 kDa**

9 10 11 12 13 14

**LMNA ~85 kDa**

9 10 11 12 13 14

**GAPDH 38 kDa**

9 10 11 12 13 14

**Histone 3 17 kDa**

9 10 11 12 13 14

Membrane II (Immunoblotting: CDK13, RNA pol II CTD, LMNA, GAPDH, Histone 3).

**Ponceau staining**

8 9 10 11 12 13 14 15

8 9 10 11 12 13 14 15

|  | Replicate n2 |
| --- | --- |
| Lane n | Sample ID |
| 8 | Molecular marker (260-15 kDa) |
| 9 | LCC9 DMSO - Cyt. (n2) |
| 10 | LCC9 DMSO - Nucl. (n2) |
| 11 | LCC9 DMSO - Chrom. (n2) |
| 12 | LCC9 CT7116 - Cyt. (n2) |
| 13 | LCC9 CT7116 - Nucl. (n2) |
| 14 | LCC9 CT7116 - Chrom.(n2) |
| 15 | Molecular marker (260-15 kDa) |

**CDK13 170 kDa**

9 10 11 12 13 14

**RNA pol II CTD 250 kDa**

9 10 11 12 13 14

**LMNA ~85 kDa**

9 10 11 12 13 14

**GAPDH 38 kDa**

9 10 11 12 13 14

**Histone 3 17 kDa**

9 10 11 12 13 14

#### Replicate number 3

Membrane I (Immunoblotting: pSer2 CTD, CCNK, LMNA, GAPDH, Histone 3).

8 9 10 11 12 13 14 15

**Ponceau staining**

|  | Replicate n3 |
| --- | --- |
| Lane n | Sample ID |
| 8 | Molecular marker (260-15 kDa) |
| 9 | LCC9 DMSO - Cyt. (n3) |
| 10 | LCC9 DMSO - Nucl. (n3) |
| 11 | LCC9 DMSO - Chrom. (n3) |
| 12 | LCC9 CT7116 - Cyt. (n3) |
| 13 | LCC9 CT7116 - Nucl. (n3) |
| 14 | LCC9 CT7116 - Chrom. (n3) |
| 15 | Molecular marker (260-15 kDa) |

**pSer2 CTD 250 kDa**

9 10 11 12 13 14

**CCNK 70 kDa**

9 10 11 12 13 14

**LMNA ~85 kDa**

9 10 11 12 13 14

**GAPDH 38 kDa**

9 10 11 12 13 14

**Histone 3 17 kDa**

9 10 11 12 13 14

Membrane II (Immunoblotting: CDK13, RNA polII CTD, LMNA, GAPDH, Histone 3).

8 9 10 11 12 13 14 15

**Ponceau staining**

|  | Replicate n3 |
| --- | --- |
| Lane n | Sample ID |
| 8 | Molecular marker (260-15 kDa) |
| 9 | LCC9 DMSO - Cyt. (n3) |
| 10 | LCC9 DMSO - Nucl. (n3) |
| 11 | LCC9 DMSO - Chrom. (n3) |
| 12 | LCC9 CT7116 - Cyt. (n3) |
| 13 | LCC9 CT7116 - Nucl. (n3) |
| 14 | LCC9 CT7116 - Chrom. (n3) |
| 15 | Molecular marker (260-15 kDa) |

**CDK13 170 kDa**

9 10 11 12 13 14

**RNA pol II CTD 250 kDa**

9 10 11 12 13 14

**LMNA ~85 kDa**

9 10 11 12 13 14

**GAPDH 38 kDa**

9 10 11 12 13 14

**Histone 3 17 kDa**

9 10 11 12 13 14

### LY2 bone cells

#### Replicate number 1 (shown in Supplementary Figure 7A)

Membrane I (Immunoblotting: CDK13, pSer2 CTD, LMNB2, GAPDH, Histone 3).

**Ponceau staining**

8 9 10 11 12 13 14 15

|  | Replicate n1 |
| --- | --- |
| Lane n | Sample ID |
| 8 | Molecular marker (260-15 kDa) |
| 9 | LY2 bone DMSO - Cyt. (n1) |
| 10 | LY2 bone DMSO - Nucl. (n1) |
| 11 | LY2 bone DMSO - Chrom. (n1) |
| 12 | LY2 bone CT7116 - Cyt. (n1) |
| 13 | LY2 bone CT7116 - Nucl. (n1) |
| 14 | LY2 bone CT7116 - Chrom. (n1) |
| 15 | Molecular marker (260-15 kDa) |

**CDK13 170 kDa**

9 10 11 12 13 14

**pSer2 CTD 250 kDa**

9 10 11 12 13 14

**LMNB2 68 kDa**

9 10 11 12 13 14

**GAPDH 38 kDa**

9 10 11 12 13 14

**Histone 3 17 kDa**

9 10 11 12 13 14

Membrane II (Immunoblotting: RNA polII CTD, CCNK, LMNB2, GAPDH, Histone 3).

**Ponceau staining**

8 9 10 11 12 13 14 15

|  | Replicate n1 |
| --- | --- |
| Lane n | Sample ID |
| 8 | Molecular marker (260-15 kDa) |
| 9 | LY2 bone DMSO - Cyt. (n1) |
| 10 | LY2 bone DMSO - Nucl. (n1) |
| 11 | LY2 bone DMSO - Chrom. (n1) |
| 12 | LY2 bone CT7116 - Cyt. (n1) |
| 13 | LY2 bone CT7116 - Nucl. (n1) |
| 14 | LY2 bone CT7116 - Chrom. (n1) |
| 15 | Molecular marker (260-15 kDa) |

**RNA pol II CTD 250 kDa**

9 10 11 12 13 14

9 10 11 12 13 14

**CCNK 70 kDa**

**LMNB2 68 kDa**

9 10 11 12 13 14

**GAPDH 38 kDa**

9 10 11 12 13 14

**Histone 3 17 kDa**

9 10 11 12 13 14

#### Replicate number 2

Membrane I (Immunoblotting: pSer2 CTD, CCNK, LMNA, GAPDH, Histone 3).

8 9 10 11 12 13 14 15

**Ponceau staining**

|  | Replicate n2 |
| --- | --- |
| Lane n | Sample ID |
| 8 | Molecular marker (260-15 kDa) |
| 9 | LY2 bone DMSO - Cyt. (n2) |
| 10 | LY2 bone DMSO - Nucl. (n2) |
| 11 | LY2 bone DMSO - Chrom. (n2) |
| 12 | LY2 bone CT7116 - Cyt. (n2) |
| 13 | LY2 bone CT7116 - Nucl. (n2) |
| 14 | LY2 bone CT7116 - Chrom. (n2) |
| 15 | Molecular marker (260-15 kDa) |

**pSer2 CTD 250 kDa**

9 10 11 12 13 14

**CCNK 70 kDa**

9 10 11 12 13 14

**LMNA ~85 kDa**

9 10 11 12 13 14

**GAPDH 38 kDa**

9 10 11 12 13 14

**Histone 3 17 kDa**

9 10 11 12 13 14

Membrane II (Immunoblotting: CDK13, RNA pol II CTD, LMNA, GAPDH, Histone 3).

**Ponceau staining**

8 9 10 11 12 13 14 15

|  | Replicate n2 |
| --- | --- |
| Lane n | Sample ID |
| 8 | Molecular marker (260-15 kDa) |
| 9 | LY2 bone DMSO - Cyt. (n2) |
| 10 | LY2 bone DMSO - Nucl. (n2) |
| 11 | LY2 bone DMSO - Chrom. (n2) |
| 12 | LY2 bone CT7116 - Cyt. (n2) |
| 13 | LY2 bone CT7116 - Nucl. (n2) |
| 14 | LY2 bone CT7116 - Chrom. (n2) |
| 15 | Molecular marker (260-15 kDa) |

**CDK13 170 kDa**

9 10 11 12 13 14

**RNA pol II CTD 250 kDa**

9 10 11 12 13 14

**LMNA ~85 kDa**

9 10 11 12 13 14

**GAPDH 38 kDa**

9 10 11 12 13 14

**Histone 3 17 kDa**

9 10 11 12 13 14

#### Replicate number 3

Membrane I (Immunoblotting: pSer2 CTD, CCNK, LMNA, GAPDH, Histone 3).

8 9 10 11 12 13 14 15

**Ponceau staining**

|  | Replicate n3 |
| --- | --- |
| Lane n | Sample ID |
| 8 | Molecular marker (260-15 kDa) |
| 9 | LY2 bone DMSO - Cyt. (n3) |
| 10 | LY2 bone DMSO - Nucl. (n3) |
| 11 | LY2 bone DMSO - Chrom. (n3) |
| 12 | LY2 bone CT7116 - Cyt. (n3) |
| 13 | LY2 bone CT7116 - Nucl. (n3) |
| 14 | LY2 bone CT7116 - Chrom. (n3) |
| 15 | Molecular marker (260-15 kDa) |

**pSer2 CTD 250 kDa**

9 10 11 12 13 14

**CCNK 70 kDa**

9 10 11 12 13 14

**LMNA ~85 kDa**

9 10 11 12 13 14

**GAPDH 38 kDa**

9 10 11 12 13 14

**Histone 3 17 kDa**

9 10 11 12 13 14

Membrane II (Immunoblotting: CDK13, RNA pol II CTD, LMNB2, GAPDH, Histone 3).

8 9 10 11 12 13 14 15

**Ponceau staining**

|  | Replicate n3 |
| --- | --- |
| Lane n | Sample ID |
| 8 | Molecular marker (260-15 kDa) |
| 9 | LY2 bone DMSO - Cyt. (n3) |
| 10 | LY2 bone DMSO - Nucl. (n3) |
| 11 | LY2 bone DMSO - Chrom. (n3) |
| 12 | LY2 bone CT7116 - Cyt. (n3) |
| 13 | LY2 bone CT7116 - Nucl. (n3) |
| 14 | LY2 bone CT7116 - Chrom. (n3) |
| 15 | Molecular marker (260-15 kDa) |

**CDK13 170 kDa**

9 10 11 12 13 14

**RNA pol II CTD 250 kDa**

9 10 11 12 13 14

**LMNA ~85 kDa**

9 10 11 12 13 14

**GAPDH 38 kDa**

9 10 11 12 13 14

**Histone 3 17 kDa**

9 10 11 12 13 14

### T347

#### Replicate number 1 (hown in Supplementary Figure 7A)

Membrane I (Immunoblotting: CDK13, pSer2 CTD, LMNB2, GAPDH, Histone 3).

**Ponceau staining**

1 2 3 4 5 6 7 8

|  | Replicate n1 |
| --- | --- |
| Lane n | Sample ID |
| 1 | Molecular marker (260-15 kDa) |
| 2 | T347 DMSO - Cyt. (n1) |
| 3 | T347 DMSO - Nucl. (n1) |
| 4 | T347 DMSO - Chrom. (n1) |
| 5 | T347 CT7116 - Cyt. (n1) |
| 6 | T347 CT7116 - Nucl. (n1) |
| 7 | T347 CT7116 - Chrom. (n1) |
| 8 | Molecular marker (260-15 kDa) |

**CDK13 170 kDa**

2 3 4 5 6 7

**pSer2 CTD 250 kDa**

2 3 4 5 6 7

**LMNB2 68 kDa**

2 3 4 5 6 7

**GAPDH 38 kDa**

2 3 4 5 6 7

**Histone 3 17 kDa**

2 3 4 5 6 7

Membrane II (Immunoblotting: RNA polII CTD, CCNK, LMNB2, GAPDH, Histone 3).

**Ponceau staining**

1 2 3 4 5 6 7 8

|  | Replicate n1 |
| --- | --- |
| Lane n | Sample ID |
| 1 | Molecular marker (260-15 kDa) |
| 2 | T347 DMSO - Cyt. (n1) |
| 3 | T347 DMSO - Nucl. (n1) |
| 4 | T347 DMSO - Chrom. (n1) |
| 5 | T347 CT7116 - Cyt. (n1) |
| 6 | T347 CT7116 - Nucl. (n1) |
| 7 | T347 CT7116 - Chrom. (n1) |
| 8 | Molecular marker (260-15 kDa) |

**RNA polII CTD 250 kDa**

2 3 4 5 6 7

**CCNK 70 kDa**

2 3 4 5 6 7

**LMNB2 68 kDa**

2 3 4 5 6 7

**GAPDH 38 kDa**

2 3 4 5 6 7

**Histone 3 17 kDa**

2 3 4 5 6 7

#### Replicate number 2

Membrane I (pSer2 CTD, CCNK, LMNA, GAPDH, Histone 3).

**Ponceau staining**

1 2 3 4 5 6 7 8

|  | Replicate n2 |
| --- | --- |
| Lane n | Sample ID |
| 1 | Molecular marker (260-15 kDa) |
| 2 | T347 DMSO - Cyt. (n2) |
| 3 | T347 DMSO - Nucl. (n2) |
| 4 | T347 DMSO - Chrom. (n2) |
| 5 | T347 CT7116 - Cyt. (n2) |
| 6 | T347 CT7116 - Nucl. (n2) |
| 7 | T347 CT7116 - Chrom. (n2) |
| 8 | Molecular marker (260-15 kDa) |

**pSer2 CTD 250 kDa**

2 3 4 5 6 7

**CCNK 70 kDa**

2 3 4 5 6 7

**LMNA ~85 kDa**

2 3 4 5 6 7

**GAPDH 38 kDa**

2 3 4 5 6 7

**Histone 3 17 kDa**

2 3 4 5 6 7

Membrane II (CDK13, RNA pol II CTD, LMNA, GAPDH, Histone 3).

**Ponceau staining**

1 2 3 4 5 6 7 8

|  | Replicate n2 |
| --- | --- |
| Lane n | Sample ID |
| 1 | Molecular marker (260-15 kDa) |
| 2 | T347 DMSO - Cyt. (n2) |
| 3 | T347 DMSO - Nucl. (n2) |
| 4 | T347 DMSO - Chrom. (n2) |
| 5 | T347 CT7116 - Cyt. (n2) |
| 6 | T347 CT7116 - Nucl. (n2) |
| 7 | T347 CT7116 - Chrom. (n2) |
| 8 | Molecular marker (260-15 kDa) |

**CDK13 170 kDa**

2 3 4 5 6 7

**RNA pol II CTD 250 kDa**

2 3 4 5 6 7

**LMNA ~85 kDa**

2 3 4 5 6 7

**GAPDH 38 kDa**

2 3 4 5 6 7

**Histone 3 17 kDa**

2 3 4 5 6 7

#### Replicate number 3

Membrane I (Immunoblotting: pSer2 CTD, CCNK, LMNA, GAPDH, Histone 3).

|  | Replicate n3 |
| --- | --- |
| Lane n | Sample ID |
| 1 | Molecular marker (260-15 kDa) |
| 2 | T347 DMSO - Cyt. (n3) |
| 3 | T347 DMSO - Nucl. (n3) |
| 4 | T347 DMSO - Chrom. (n3) |
| 5 | T347 CT7116 - Cyt. (n3) |
| 6 | T347 CT7116 - Nucl. (n3) |
| 7 | T347 CT7116 - Chrom. (n3) |
| 8 | Molecular marker (260-15 kDa) |

**Ponceau staining**

1 2 3 4 5 6 7 8

**pSer2 CTD 250 kDa**

2 3 4 5 6 7

**CCNK 70 kDa**

2 3 4 5 6 7

**LMNA ~85 kDa**

2 3 4 5 6 7

**GAPDH 38 kDa**

2 3 4 5 6 7

**Histone 3 17 kDa**

2 3 4 5 6 7

Membrane II (Immunoblotting: CDK13, RNA pol II CTD, LMNA, GAPDH, Histone 3).

**Ponceau staining**

1 2 3 4 5 6 7 8

|  | Replicate n3 |
| --- | --- |
| Lane n | Sample ID |
| 1 | Molecular marker (260-15 kDa) |
| 2 | T347 DMSO - Cyt. (n3) |
| 3 | T347 DMSO - Nucl. (n3) |
| 4 | T347 DMSO - Chrom. (n3) |
| 5 | T347 CT7116 - Cyt. (n3) |
| 6 | T347 CT7116 - Nucl. (n3) |
| 7 | T347 CT7116 - Chrom. (n3) |
| 8 | Molecular marker (260-15 kDa) |

**CDK13 170 kDa**

2 3 4 5 6 7

**RNA pol II CTD 250 kDa**

2 3 4 5 6 7

**LMNA ~85 kDa**

2 3 4 5 6 7

**GAPDH 38 kDa**

2 3 4 5 6 7

**Histone 3 17 kDa**

2 3 4 5 6 7

## Supplementary Material 9. Uncropped western blot membranes in Supplementary figure 6B.

**Protein expression following CDK12 knockdown (siRNA, 48 hours) in T47D cells.** CDK12, MED1, ER + loading controls for cytoplasmic (GAPDH), nuclear (LMNB2) and chromatin-bound protein fractions (Histone 3).

### T47D cells

#### Replicate number 1 (lanes 2 to 7) + Replicate number 2 (lanes 9 to 14, shown in Supplementary Figure 6B)

**Ponceau staining**

|  | Replicate n1 and n2 |
| --- | --- |
| Lane n | Sample ID |
| 1 | Molecular marker (260-15 kDa) |
| 2 | T47D siCtrl - Cyt. (n1) |
| 3 | T47D siCtrl - Nucl. (n1) |
| 4 | T47D siCtrl - Chrom. (n1) |
| 5 | T47D siCDK12 - Cyt. (n1) |
| 6 | T47D siCDK12 - Nucl. (n1) |
| 7 | T47D siCDK12 - Chrom. (n1) |
| 8 | Molecular marker (260-15 kDa) |
| 9 | T47D siCtrl - Cyt. (n2) |
| 10 | T47D siCtrl - Nucl. (n2) |
| 11 | T47D siCtrl - Chrom. (n2) |
| 12 | T47D siCDK12 - Cyt. (n2) |
| 13 | T47D siCDK12 - Nucl. (n2) |
| 14 | T47D siCDK12 - Chrom. (n2) |
| 15 | Molecular marker (260-15 kDa) |

1 2 3 4 5 6 7 8 9 10 11 12 13 14 15

**CDK12 205kDa**

1 2 3 4 5 6 7 8 9 10 1 1 12 13 14 15

**ER 60kDa**

1 2 3 4 5 6 7 8 9 10 1 1 12 13 14 15

**pMED1 ~240kDa**

1 2 3 4 5 6 7 8 9 10 1 1 12 13 14 15

**LMNA ~85 kDa**

1 2 3 4 5 6 7 8 9 10 1 1 12 13 14 15

**GAPDH 38kDa**

1 2 3 4 5 6 7 8 9 10 1 1 12 13 14 15

**Histone 3 17kDa**

1 2 3 4 5 6 7 8 9 10 1 1 12 13 14 15

Membrane II (Immunoblotting: MED1, LMNB2, GAPDH, Histone 3).

**Ponceau staining**

|  | Replicate n1 and n2 |
| --- | --- |
| Lane n | Sample ID |
| 1 | Molecular marker (260-15 kDa) |
| 2 | T47D siCtrl - Cyt. (n1) |
| 3 | T47D siCtrl - Nucl. (n1) |
| 4 | T47D siCtrl - Chrom. (n1) |
| 5 | T47D siCDK12 - Cyt. (n1) |
| 6 | T47D siCDK12 - Nucl. (n1) |
| 7 | T47D siCDK12 - Chrom. (n1) |
| 8 | Molecular marker (260-15 kDa) |
| 9 | T47D siCtrl - Cyt. (n2) |
| 10 | T47D siCtrl - Nucl. (n2) |
| 11 | T47D siCtrl - Chrom. (n2) |
| 12 | T47D siCDK12 - Cyt. (n2) |
| 13 | T47D siCDK12 - Nucl. (n2) |
| 14 | T47D siCDK12 - Chrom. (n2) |
| 15 | Molecular marker (260-15 kDa) |

1 2 3 4 5 6 7 8 9 10 1 1 12 13 14 15

**MED1 220kDa**

1 2 3 4 5 6 7 8 9 10 1 1 12 13 14 15

**LMNA ~85 kDa**

1 2 3 4 5 6 7 8 9 10 1 1 12 13 14 15

**GAPDH 38kDa**

1 2 3 4 5 6 7 8 9 10 1 1 12 13 14 15

**Histone 3 17kDa**

1 2 3 4 5 6 7 8 9 10 11 12 13 14 15

#### Replicate number 3

**Ponceau staining**

|  | Replicate n3 |
| --- | --- |
| Lane n | Sample ID |
| 1 | Molecular marker (260-15 kDa) |
| 2 | T47D siCtrl - Cyt. (n2) |
| 3 | T47D siCtrl - Nucl. (n2) |
| 4 | T47D siCtrl - Chrom. (n2) |
| 5 | T47D siCDK12 - Cyt. (n2) |
| 6 | T47D siCDK12 - Nucl. (n2) |
| 7 | T47D siCDK12 - Chrom. (n2) |
| 8 | Molecular marker (260-15 kDa) |
| 9 | T47D siCtrl - Cyt. (n3) |
| 10 | T47D siCtrl - Nucl. (n3) |
| 11 | T47D siCtrl - Chrom. (n3) |
| 12 | T47D siCDK12 - Cyt. (n3) |
| 13 | T47D siCDK12 - Nucl. (n3) |
| 14 | T47D siCDK12 - Chrom. (n3) |
| 15 | Molecular marker (260-15 kDa) |

1 2 3 4 5 6 7 8 9 10 1 1 12 13 14 15

**CDK12 205kDa**

1 2 3 4 5 6 7 8

**ER 60kDa**

1 2 3 4 5 6 7 8

**pMED1 ~240kDa**

1 2 3 4 5 6 7 8

**LMNA ~85 kDa**

1 2 3 4 5 6 7 8

**GAPDH 38kDa**

1 2 3 4 5 6 7 8 9 10 11 12 13 14 15

**Histone 3 17kDa**

1 2 3 4 5 6 7 8 9 10 11 12 13 14 15

**MED1 220kDa**

8 9 10 11 12 13 14 15

**LMNA ~85 kDa**

8 9 10 11 12 13 14 15

## Supplementary Material 10. Uncropped western blot membranes in Supplementary Figure 6C.

**Protein expression following CDK12 overexpression (48 hours) in MCF7 cells.** CDK12, MED1, ER, p-MED1 + loading controls for cytoplasmic (GAPDH), nuclear (LMNA) and chromatin-bound protein fractions (Histone 3).

### MCF7 cells

#### Replicate number 1 (shown in Supplementary Figure 6C)

Membrane I (Immunoblotting: CDK12, ER, pMED1, LMNA, GAPDH, Histone 3).

**Ponceau staining**

|  | Replicate n1 |
| --- | --- |
| Lane n | Sample ID |
| 1 | Molecular marker (260-15 kDa) |
| 2 | MCF7 vCTRL - Cyt. (n1) |
| 3 | MCF7 vCTRL - Nucl. (n1) |
| 4 | MCF7 vCTRL - Chrom. (n1) |
| 5 | MCF7 vCDK12 - Cyt. (n1) |
| 6 | MCF7 vCDK12 - Nucl. (n1) |
| 7 | MCF7 vCDK12 – Chrom (n1) |

1 2 3 4 5 6 7

**CDK12 205 kDa**

1 2 3 4 5 6 7

**pMED1 ~240 kDa**

1 2 3 4 5 6 7

**ER ~65 kDa**

1 2 3 4 5 6 7

**LMNA ~85 kDa**

1 2 3 4 5 6 7

**GAPDH 38 kDa**

1 2 3 4 5 6 7

**H3 17 kDa**

1 2 3 4 5 6 7

Membrane II (Immunoblotting: MED1, LMNA, GAPDH, Histone 3).

**Ponceau staining**

|  | Replicate n1 |
| --- | --- |
| Lane n | Sample ID |
| 1 | Molecular marker (260-15 kDa) |
| 2 | MCF7 vCTRL - Cyt. (n1) |
| 3 | MCF7 vCTRL - Nucl. (n1) |
| 4 | MCF7 vCTRL - Chrom. (n1) |
| 5 | MCF7 vCDK12 - Cyt. (n1) |
| 6 | MCF7 vCDK12 - Nucl. (n1) |
| 7 | MCF7 vCDK12 – Chrom (n1) |

1 2 3 4 5 6 7

**MED1 220 kDa**

1 2 3 4 5 6 7

**LMNA ~85 kDa**

1 2 3 4 5 6 7

**GAPDH 38 kDa**

1 2 3 4 5 6 7

**H3 17 kDa**

1 2 3 4 5 6 7

#### Replicate number 2

Membrane I (Immunoblotting: CDK12, ER, p-MED1, LMNA, GAPDH, Histone 3).

**Ponceau staining**

|  | Replicate n2 |
| --- | --- |
| Lane n | Sample ID |
| 8 | Molecular marker (260-15 kDa) |
| 9 | MCF7 vCTRL - Cyt. (n2) |
| 10 | MCF7 vCTRL - Nucl. (n2) |
| 11 | MCF7 vCTRL - Chrom. (n2) |
| 12 | MCF7 vCDK12 - Cyt. (n2) |
| 13 | MCF7 vCDK12 - Nucl. (n2) |
| 14 | MCF7 vCDK12 – Chrom (n2) |

8 9 10 11 12 13 14

**CDK12 205 kDa**

8 9 10 11 12 13 14

**ER ~65 kDa**

8 9 10 11 12 13 14

**LMNA ~85 kDa**

8 9 10 11 12 13 14

**GAPDH 38 kDa**

8 9 10 11 12 13 14

**H3 17 kDa**

8 9 10 11 12 13 14

Membrane II (Immunoblotting: MED1, LMNA, GAPDH, Histone 3).

**Ponceau staining**

|  | Replicate n2 |
| --- | --- |
| Lane n | Sample ID |
| 8 | Molecular marker (260-15 kDa) |
| 9 | MCF7 vCTRL - Cyt. (n2) |
| 10 | MCF7 vCTRL - Nucl. (n2) |
| 11 | MCF7 vCTRL - Chrom. (n2) |
| 12 | MCF7 vCDK12 - Cyt. (n2) |
| 13 | MCF7 vCDK12 - Nucl. (n2) |
| 14 | MCF7 vCDK12 – Chrom (n2) |

8 9 10 11 12 13 14

**MED1 220 kDa**

8 9 10 11 12 13 14

**LMNA ~85 kDa**

8 9 10 11 12 13 14

**GAPDH 38 kDa**

8 9 10 11 12 13 14

**H3 17 kDa**

8 9 10 11 12 13 14

Membrane III (Immunoblotting: pMED1, LMNA, GAPDH, Histone 3).

**Ponceau staining**

|  | Replicate n2 |
| --- | --- |
| Lane n | Sample ID |
| 1 | Molecular marker (260-15 kDa) |
| 2 | MCF7 vCTRL - Cyt. (n2) |
| 3 | MCF7 vCTRL - Nucl. (n2) |
| 4 | MCF7 vCTRL - Chrom. (n2) |
| 5 | MCF7 vCDK12 - Cyt. (n2) |
| 6 | MCF7 vCDK12 - Nucl. (n2) |
| 7 | MCF7 vCDK12 – Chrom (n2) |

1 2 3 4 5 6 7

**pMED1 ~240 kD**

1 2 3 4 5 6 7

**LMNA ~85 kDa**

1 2 3 4 5 6 7

**GAPDH 38 kDa**

1 2 3 4 5 6 7

**H3 17 kDa**

1 2 3 4 5 6 7

#### Replicate number 3

Membrane I (Immunoblotting: CDK12, ER, p-MED1, LMNA, GAPDH, Histone 3).

|  | Replicate n3 |
| --- | --- |
| Lane n | Sample ID |
| 1 | Molecular marker (260-15 kDa) |
| 2 | MCF7 vCTRL - Cyt. (n3) |
| 3 | MCF7 vCTRL - Nucl. (n3) |
| 4 | MCF7 vCTRL - Chrom. (n3) |
| 5 | MCF7 vCDK12 - Cyt. (n3) |
| 6 | MCF7 vCDK12 - Nucl. (n3) |
| 7 | MCF7 vCDK12 - Chrom. (n3) |
| 8 | Molecular marker (260-15 kDa) |
| 9 | MCF7 vCTRL - Cyt. (n3) |
| 10 | MCF7 vCTRL - Nucl. (n3) |
| 11 | MCF7 vCTRL - Chrom. (n3) |
| 12 | MCF7 vCDK12 - Cyt. (n3) |
| 13 | MCF7 vCDK12 - Nucl. (n3) |
| 14 | MCF7 vCDK12 - Chrom. (n3) |
| 15 | Molecular marker (260-15 kDa) |

1 2 3 4 5 6 7 8 9 10 11 12 13 14 15

**CDK12 (205 kDa)**

**pMED1 (~240 kD)**

**ER ~65 kDa**

1 2 3 4 5 6 7

**LMNA ~85 kDa**

1 2 3 4 5 6 7

**GAPDH 38 kDa**

1 2 3 4 5 6 7

**H3 17 kDa**

1 2 3 4 5 6 7

**MED1 (220 kDa)**

8 9 10 11 12 13 14

**LMNA (~85 kDa)**

8 9 10 11 12 13 14

**GAPDH (38 kDa)**

8 9 10 11 12 13 14

**H3 (17 kDa)**

8 9 10 11 12 13 14

## Supplementary Material 11. Uncropped western blot membranes in Supplementary figure 6D.

**Protein expression following ESR1 knockdown (48 hours) in LY2 cells.** ER, CDK12, MED1 + loading controls for cytoplasmic (GAPDH), nuclear (LMNB2) and chromatin-bound protein fractions (Histone 3).

### LY2 cells

#### Replicate number 1 (lanes 3 to 8) + Replicate number 2 (lanes 10 to 15, shown in Supplementary Figure 6D)

Membrane I (Immunoblotting: ER, CDK12, pMED1, LMNA, GAPDH, Histone 3).

**Ponceau staining**

|  | Replicate n1 and n2 |
| --- | --- |
| Lane n | Sample ID |
| 1 | Molecular marker (260-15 kDa) |
| 2 | - |
| 3 | LY2 siCtrl - Cyt. (n1) |
| 4 | LY2 siCtrl - Nucl. (n1) |
| 5 | LY2 siCtrl - Chrom. (n1) |
| 6 | LY2 siESR1 - Cyt. (n1) |
| 7 | LY2 siESR1 - Nucl. (n1) |
| 8 | LY2 siESR1 - Chrom. (n1) |
| 9 | Molecular marker (260-15 kDa) |
| 10 | LY2 siCtrl - Cyt. (n2) |
| 11 | LY2 siCtrl - Nucl. (n2) |
| 12 | LY2 siCtrl - Chrom. (n2) |
| 13 | LY2 siESR1 - Cyt. (n2) |
| 14 | LY2 siESR1 - Nucl. (n2) |
| 15 | LY2 siESR1 - Chrom. (n2) |

1 2 3 4 5 6 7 8 9 10 11 12 13 14 15

**ER 60kDa**

1 2 3 4 5 6 7 8 9 10 11 12 13 14 15

**CDK12 205kDa**

1 2 3 4 5 6 7 8 9 10 11 12 13 14 15

**pMED1 ~240kDa**

1 2 3 4 5 6 7 8

9 10 11 12 13 14 15

**LMNA ~85 kDa**

1 2 3 4 5 6 7 8 9 10 11 12 13 14 15

**GAPDH 38kDa**

1 2 3 4 5 6 7 8 9 10 11 12 13 14 15

**Histone 3 17kDa**

1 2 3 4 5 6 7 8 9 10 11 12 13 14 15

Membrane II (Immunoblotting: MED1, LMNA, GAPDH, Histone 3).

**Ponceau staining**

|  | Replicate n1 and n2 |
| --- | --- |
| Lane n | Sample ID |
| 1 | Molecular marker (260-15 kDa) |
| 2 | - |
| 3 | LY2 siCtrl - Cyt. (n1) |
| 4 | LY2 siCtrl - Nucl. (n1) |
| 5 | LY2 siCtrl - Chrom. (n1) |
| 6 | LY2 siESR1 - Cyt. (n1) |
| 7 | LY2 siESR1 - Nucl. (n1) |
| 8 | LY2 siESR1 - Chrom. (n1) |
| 9 | Molecular marker (260-15 kDa) |
| 10 | LY2 siCtrl - Cyt. (n2) |
| 11 | LY2 siCtrl - Nucl. (n2) |
| 12 | LY2 siCtrl - Chrom. (n2) |
| 13 | LY2 siESR1 - Cyt. (n2) |
| 14 | LY2 siESR1 - Nucl. (n2) |
| 15 | LY2 siESR1 - Chrom. (n2) |

1 2 3 4 5 6 7 8 9 10 11 12 13 14 15

**MED1 220kDa**

1 2 3 4 5 6 7 8 9 10 11 12 13 14 15

**LMNA ~85 kDa**

1 2 3 4 5 6 7 8 9 10 11 12 3 14 15

**GAPDH 38kDa**

1 2 3 4 5 6 7 8 9 10 11 12 13 14 15

**Histone 3 17kDa**

1 2 3 4 5 6 7 8 9 10 11 12 13 14 15

#### Replicate number 3

**Ponceau staining**

|  | Replicate n3 | |
| --- | --- | --- |
| Lane n | Sample ID | |
| 1 | Molecular marker (260-15 kDa) | |
| 2 | LY2 siCtrl - Cyt. (n3) | |
| 3 | LY2 siCtrl - Nucl. (n3) | |
| 4 | LY2 siCtrl - Chrom. (n3) | |
| 5 | LY2 siESR1 - Cyt. (n3) |  |
| 6 | LY2 siESR1 - Nucl. (n3) | |
| 7 | LY2 siESR1 - Chrom. (n3) | |
| 8 | Molecular marker (260-15 kDa) | |
| 9 | LY2 siCtrl - Cyt. (n3) | |
| 10 | LY2 siCtrl - Nucl. (n3) | |
| 11 | LY2 siCtrl - Chrom. (n3) | |
| 12 | LY2 siESR1 - Cyt. (n3) | |
| 13 | LY2 siESR1 - Nucl. (n3) | |
| 14 | LY2 siESR1 - Chrom. (n3) | |
| 15 | Molecular marker (260-15 kDa) | |

1 2 3 4 5 6 7 8 9 10 11 12 13 14 15

**ER 60kDa**

1 2 3 4 5 6 7 8

**CDK12 205kDa**

1 2 3 4 5 6 7 8

**pMED1 ~240kDa**

1 2 3 4 5 6 7 8

**LMNA ~85 kDa**

1 2 3 4 5 6 7 8

**GAPDH 38kDa**

1 2 3 4 5 6 7 8 9 10 11 12 13 14 15

**Histone 3 17kDa**

1 2 3 4 5 6 7 8 9 10 11 12 13 14 15

**MED1 220kDa**

8 9 10 11 12 13 14 15

**LMNA ~85 kDa**

8 9 10 11 12 13 14 15
