# Supplementary material for: Targeting CDK12 disrupts estrogen-receptor chromatin recruitment and ER-MED1 transcription in advanced ER+ breast cancer
Source: J Natl Cancer Inst. 2025 Oct 15;118(3):404–21. doi: 10.1093/jnci/djaf295 (PMC13016824; doi:10.1093/jnci/djaf295)
Supplement: djaf295_Supplementary_Data [file djaf295_supplementary_data.zip › Ottaviani et al. CDK12_Supplementary Methods .docx]

# Targeting CDK12 disrupts estrogen-receptor chromatin recruitment and ER-MED1 transcription in advanced ER+ breast cancer

Daniela Ottaviani^1*^, PhD, Mihaela Ola^1^, PhD, Alessandra Allotta^1^, MSc, Yasmine Maati Chaibi^1^, MSc, Seán Hickey^1^, BSc, Petra Jagust^1^, PhD, Nicola Cosgrove^1^, PhD, Sinéad Cocchiglia^1^, MSc, Fiona Bane^1^, MSc, Ramón Fallon^1^, MSc, Gordon Daly^1^, MCh, Aisling Hegarty^1,2^, PhD, Lance Hudson^1,2^, BSc, Katherine Sheehan^1,3^, MD, Shannon Kalsi^1^, MSc, Stephen Shovlin^1^, PhD, Aoibhín Powell^1,4^, BSc, Ash Bahl^5^, PhD, Ed Ainscow^5^, PhD, Steffi Oesterreich^6,7^, PhD, Adrian V. Lee^6,7^, PhD, Fergus J. Couch^8^, PhD, Arnold D.K. Hill^9^, MD, Damir Varešlija^1,4*^, PhD, and Leonie Young^1,2,9*#^, PhD.

## Supplementary Methods

### Patient acquisition

Informed and written consent was obtained before clinical material was collected under the observational clinical trial NCT01840293 (https://clinicaltrials.gov), following ethical approval from Beaumont Hospital Medical Research Ethics Committee (Dublin, Ireland). Clinical data from the University of Pittsburgh and Mayo Clinic was approved as previously described(1). The study population included female breast cancer patients from three independent institutions: Royal College of Surgeons in Ireland, University of Medicine and Health Sciences (Dublin, Ireland), University of Pittsburgh (Pittsburgh, Pennsylvania, United States) and Mayo Clinic (Rochester, Minnesota, United States).

**Patient sample processing and sequencing**

Tissue processing, whole-exome DNA sequencing (39 patients, 78 tumors), and RNA sequencing library preparation (45 patients, 90 tumors) for patient-matched primary breast and brain metastatic tumor samples are described in Cosgrove et al(1).

### Genomic Analysis

#### Somatic copy number alterations (SCNA)

Segmentation files from FACETS allele-specific copy number calling were used as input for identification of recurrent amplifications and deletions using GISTIC2.0 (v2.0.23) ([https://github.com/broadinstitute/gistic2](https://eur02.safelinks.protection.outlook.com/?url=https%3A%2F%2Fgithub.com%2Fbroadinstitute%2Fgistic2&data=05%7C02%7Cdanielaottaviani%40rcsi.ie%7Ca353a7e574e446d03ee908dc6e1dfc37%7C607041e7a8124670bd3030f9db210f06%7C0%7C0%7C638506322289615559%7CUnknown%7CTWFpbGZsb3d8eyJWIjoiMC4wLjAwMDAiLCJQIjoiV2luMzIiLCJBTiI6Ik1haWwiLCJXVCI6Mn0%3D%7C0%7C%7C%7C&sdata=Rm6ij76ua%2BTXGnvVn64osHMoKUW6Ir2BiYbaefEiMAs%3D&reserved=0)). To identify recurrent SCNA specific to tumour type, GISTIC2 was run on the primary breast tumors (n = 39) and brain metastatic tumors (n = 39). GISTIC2.0 parameters used were ta=0.1 (amplification threshold); td=0.1 (deletion threshold); qvt=0.25 (q-value significance threshold); maxseg=4000 (maximum number of segments allowed for a sample); brlen=0.5 (broad length cutoff); conf=0.9 (confidence level of 90%); genegistic=1 (the gene GISTIC algorithm to be used for deletion significance calculations at gene level rather than at marker level); armpeel=1 (arm-level peel-off of events during peak definition are enabled). Top ten genes shown in Figure 1 were defined by percentage of amplifications.

#### Gene fusions

Gene fusions were detected in RNA sequencing data from the cohort of primary breast (n *=* 45) and brain metastatic tumors (n = 45) using two fusion detection algorithms, Arriba (v1.1.0)(2) and STAR Fusion (v1.4.0)(3), on each sample separately. Arriba was run with default parameters (including position specific blacklist (hg38 GRCH38 2018-11-04) to remove recurrent artifacts and transcripts observed in benign tissue). The STAR-Fusion pipeline was run with default parameters using STAR aligner v2.6.0a. The STAR-Fusion “*FusionFilter*” software module was applied on fusion candidates to remove artifacts, false positive fusion genes and any fusions having less than 1 evidence fragment per 10 M total read (0.1 FFPM). Further filtering was applied to remove any fusion calls that matched the Heyer et al., 2019(4) manually curated blacklist of false positive fusion calls. For each tumour sample, the Arriba and STAR-Fusion filtered fusion sets were combined (union). Fusions detected by both algorithms, high or medium confidence in Arriba or a FFPM > 0.1 by STAR-Fusion, were kept for downstream analysis. Recurrent fusions were defined as those found in more than two tumour samples.

#### Gene expression

The ComplexHeatmap(5) R package was used to generate the oncoprint graph to show gene expression differences between the brain metastatic samples relative to their matched primary tumors. Batch corrected log2 normalised values (log2 CPM TMM) were calculated for each sample. Gene expression differences between brain metastasis and primary tumors normalised values were calculated for each patient and sorted in ascending order. Gene expression difference events for CDKs and cyclin-related genes (*CDK1*, C*DK2*, *CDK4*, *CDK7*, *CDK9*, *CDK12*, *CDK13*, *E2F1*, *E2F7*, *CCND1*, *CCNE1*, *RB1*, *MYC*) were defined as *increased*, *decreased* or no significant difference observed. An increase was registered if the difference of expression of a given patient’s sample pair (brain metastasis/primary tumor) was within the top 95 % quantile of the expression differences of all genes for that patient. A decrease was defined when the difference of expression of a given patient’s sample pair (brain metastasis/primary tumor) was within the lowest 5 % quantile of the expression differences of all genes for that patient. Eight of the selected genes were found with more than two increase/decrease occurrences and were judged recurrent, as shown in Fig. 1D. Genes are ordered by the total number of increase/decrease events.

### Tissue microarray (TMA)

Tissue microarrays (TMAs) were constructed from formalin-fixed, paraffin-embedded (FFPE) primary breast cancer samples collected from patients enrolled at Beaumont Hospital (Dublin, Ireland) under the observational clinical trial NCT01840293, as previously described(6). All tumor samples included in the TMAs were derived from treatment-naïve patients.

Comprehensive clinical data for the samples are provided in Tables 1, 2, and 3. Tumor staging was performed according to the American Joint Committee on Cancer (AJCC) Staging Manual(7). Tumor grade and receptor status (ER, PR, HER2) were assessed in accordance with American Society of Clinical Oncology / College of American Pathologists (ASCO/CAP) guidelines(8,9).

TMA sections were processed using an automated immunohistochemical staining platform. Immunoreactivity was assessed and validated by a board-certified pathologist. CDK12 expression was evaluated using the H-score method. MED1 expression was quantified using the DAB H-score algorithm within the HALO image analysis software (Indica Labs). For both markers, positivity was defined as nuclear staining restricted to tumor epithelial cells. The CDK12-stained TMA included 820 primary tumor specimens, and the MED1-stained TMA comprised 807 primary tumors. A total of 788 primary tumors were represented in both the CDK12 and MED1 TMAs.

### Survival analysis

Kaplan-Meier estimates for overall survival (OS) and progression-free survival (PFS) were generated based on CDK12 protein expression in the RCSI CDK12 TMA cohort (*n* = 820). OS was calculated from the date of diagnosis (or, when unavailable, date of primary surgery) to death or last follow-up. PFS was calculated from the date of diagnosis (or primary surgery) to first recurrence surgery, death, or last follow-up.

CDK12 H-score and MED1 DAB H-score distributions were assessed using summary and histogram functions in GraphPad Prism (v10.1.0). For survival analysis: CDK12 expression cutoff was set at the 75th percentile (H-score = 295), dividing primary tumors into high (≥ 295) and low (< 295) expressors (Supplementary Fig. 2D). MED1 DAB expression cutoff was determined by ROC analysis using the cutpointr R package (v1.1.2), with high expression defined as H-score ≥ 108.51 (Supplementary Fig. 5C).

Tumors included in both CDK12 and MED1 TMAs were categorized into four groups based on expression status: High CDK12/High MED1, High CDK12/Low MED1, Low CDK12/High MED1, and Low CDK12/Low MED1. Kaplan-Meier survival curves were generated using GraphPad Prism and R functions survfit and ggsurvplot from packages: survival (v3.4-0), survminer (v0.4.9) (Fig. 1F, G; Fig. 3F; Supplementary Figs. 2E-H, 4D-H). Cox proportional hazards models for univariate and multivariate analysis were fitted using the coxph function in the survival R package, with hazard ratios plotted in GraphPad (Fig. 3G; Supplementary Fig. 4I, J) or using the ggforest function in the R package survminer (Supplementary Fig. 3A-C). Multivariate analysis was carried out on CDK12 protein expression, age groups (<40, 40-60, >60 years), pathological grade and pathological stage as independent variables. Patients with missing information in either of these variables were excluded from multivariate analysis. Kaplan-Meier survival estimates were generated using GraphPad Prism (v. 10.1.0) (Fig. 1F, G; Fig. 3F; Supplementary Fig. 2B-E; Supplementary Fig. 5A, C), and the survfit function in the R package survival (v3.4-0) with the ggsurvplot function in the R package survminer (v0.4.9) (Supplementary Fig. 4C-G).

***M stage Unknown*** designates patients for whom primary staging data is entirely unavailable in the clinical record. For statistical purposes, patients labeled *Mx* were clinically considered *M0* at diagnosis and have therefore been included as *M0* in regression analyses. In contrast, those labeled *Unknown* have been excluded from analyses requiring complete staging data.

### Publicly available data analysis

#### CDK12 amplifications

SCNA of primary and metastatic breast cancer were obtained from cBioportal(10–12) (<https://www.cbioportal.org/>) using the METABRIC(13–15) and the Metastatic Breast Cancer project (provisional, December 2021) on-line data sets.

#### Kaplan-Meier survival curves

Kaplan-Meier estimates of breast cancer overall survival (OS) based on CDK12 gene expression were obtained from KM plotter(16) (<https://kmplot.com/analysis/>).

#### CDKs and cyclin-related genes frequently altered in metastatic breast cancer

Gene expression was obtained using the Metastatic Breast Cancer project (provisional, December 2021) on-line data set from cBioportal(10–12) (<https://www.cbioportal.org/>).

**Epigenetic Landscape *In Silico* deletion Analysis**

Potential transcriptional partners for CDK12 were investigated using LISA(17) online tool (<http://lisa.cistrome.org/>).

**Genome-wide RNAi and CRISPR loss-of-function screening**

The effect of *MED1* knockout on breast cancer cell lines was investigated using the Cancer Dependency Map(18) (<https://depmap.org/portal/>). The DEMETER2 dependency score is based on RNAi screening datasets(19–21) available on this website. A score of 0 is equivalent to a gene that is not essential and a score of -1 corresponds to the median of all common essential genes in a given cell line.

### Cell lines and culture conditions

This study utilized endocrine-sensitive, endocrine-resistant, and metastatic breast cancer cell lines: **Endocrine sensitive:** MCF7, T47D. **Endocrine resistant:** LY2 (tamoxifen-resistant), LCC9 (anti-estrogen ICI 182,780-resistant), LETR (letrozole-resistant). **Metastatic:** LY2 bone, LY2 lung, T347.

MCF7 and T47D cells were obtained from the American Type Culture Collection (ATCC). MCF7 cells were cultured in Minimum Essential Medium (MEM; M2279, Merck) supplemented with 10% fetal calf serum (FCS; F7524, Merck) and 2 mmol/L L-glutamine (G7513, Merck). T47D cells were maintained in RPMI-1640 (R0883, Sigma-Aldrich) with 10% FCS, 2 mmol/L L-glutamine, and 1X Insulin-Transferrin-Selenium (41400-045, Fisher Scientific).

LY2(22) and LCC9 cells were kindly provided by Dr. Robert Clarke (Georgetown University, USA). LCC9 were maintained in phenol red-free MEM (PRF-MEM; 51200046, Fisher Scientific) supplemented with 5% charcoal-dextran stripped FCS (CDS-FCS; F6765, Merck). LY2 cells and their luciferase-tagged derivatives were cultured in PRF-MEM with 10% CDS-FCS, 2 mmol/L L-glutamine, and 10⁻⁸ mol/L 4-hydroxytamoxifen (4-OHT; H7904, Merck). LY2 bone and LY2 lung cells were established from intracardiac injection models as previously described (23).

LETR cells, established as described previously(24),were cultured in PRF-MEM with 10% CDS-FCS, 2 mmol/L L-glutamine, 25 nmol/L androstenedione (A-9630, Merck), 1 µmol/L letrozole (L6545, Merck), and 200 µg/mL geneticin (11811031, Fisher Scientific).

T347 cells, derived from a patient brain metastasis(25), were cultured in HBEC medium (HyClone™ DMEM/F12 1:1; SH30023.01, Cytiva) supplemented with 10 mmol/L HEPES (H3537, Merck), 5% FCS, 1X Insulin-Transferrin-Selenium, 0.5 µg/mL hydrocortisone (H0888, Merck), and 1X Antibiotic-Antimycotic (15240096, Fisher Scientific).

All cell lines were maintained at 37°C in a humidified 5% CO₂ incubator. All lines (except patient-derived T347) were genotyped and authenticated by ATCC.

### CDK12 status in cell and organoid models

CDK12 status for cell line models was determined using data from Cell Model Passports, van der Meer et al.(26), Daemen et al. (27), and our own RNA-sequencing analyses. CDK12 status for patient-derived organoid models was obtained from previously published datasets, as described in Cosgrove et al.(1) and Bruna et al.(28)

### Transient gene knockdown

Cells were seeded at 1 × 10⁵ cells per well in 2 mL of complete media in 6-well plates. After 24 h of serum deprivation (and 4-OHT-free media for LY2 cells), cells were transfected with: *CDK12* knockdown: 30 nmol/L ON-TARGETplus SMARTpool siRNA targeting *CDK12* (siCDK12; L-004031-00-0010, Dharmacon), or a non-targeting siRNA control (siCtrl; D-001810-10-20, Dharmacon); *ESR1* knockdown: 30 nmol/L Human Pre-designed siRNA Set (HY-RS04529, MedChemExpress) targeting *ESR1* (siESR1) and non-targeting control (siCtrl).

Transfections were carried out using Lipofectamine™ 2000 (11668019, Invitrogen) following the manufacturer’s instructions. After 48 h (CDK12) or 72 h (ESR1), cells were harvested for RNA or protein extraction.

### Transient gene overexpression

pcDNA3.1-CDK12 expression plasmid was kindly provided by Dr. Qintong Li (West China Second University Hospital, Sichuan University, Chengdu, China).

For overexpression experiments, cells were steroid-starved for 72 hours and subsequently seeded at a density of 1 × 10⁵ cells per well in 2 mL of complete media in 6-well plates. After 24 hours, cells were transfected with 1 µg of pcDNA3.1-CDK12 expression plasmid or empty vector control using Lipofectamine™ 2000 (11668019, Invitrogen) according to the manufacturer’s protocol. Following 48 hours of transfection, cells were harvested for RNA or protein extraction.

### RNA isolation, reverse transcription, and quantitative real-time PCR (qPCR)

Total RNA was extracted using the RNeasy Mini Kit (74106, Qiagen), and cDNA was synthesized with SuperScript III Reverse Transcriptase (18080-044, Applied Biosystems). qPCR reaction was performed using either TaqMan Fast Advance Mix (4444964) or PowerUp SYBR Green Master Mix (A25741, both Applied Biosystems), on a StepOnePlus Real-Time PCR System (Applied Biosystems) under standard cycling conditions. Gene expression levels were quantified using the ΔΔCt method and normalized to housekeeping genes ACTB and TBP.

TaqMan probes (Applied Biosystems): *CDK12* (Hs00212914_m1), *ACTB* (4333762F).

Primer sequences (designed using Primer3Plus(29)):

***ESR1*** F: CCTCCTCATCCTCTCCCACA, R: CAGCAGCAGGTCATAGAGGG

***GREB1*** F: ACGACACGCTTTGAAGAGGT, R: CATTGTCCACTCGGCTACCA

***IGFBP4*** F: CTGACAAGGACGAGGGTGAC, R: TTTTGGCGAAGTGCTTCTGC

***MED1*** F: TGGCTATCTCACACCAAGGAG, R: TGCAGTCTTGTCATCCAGTAGG

***NRIP1*** F: ACACAGCCAGAAGATGCACA, R: TCTCCAAGCTCTGAGCCTCT

***TBP*** F: TATAATCCCAAGCGGTTTGC, R: GCACACCATTTTCCCAGAAC

### RNA sequencing (RNA-seq)

RNA-seq was carried out on LY2 cells (siCDK12: CDK12 knockdown, siCtrl: control) on the BGISEQ-500 PE100 platform with 20 megabase (Mb) clean reads per sample. Experiment was performed in three biological replicates.

### RNA-seq analysis

#### Differential gene expression and pathway enrichment analysis

Raw sequencing reads were pre-filtered using BGI internal software SOAPnuke(30) (v2.2.5) to remove adaptors, reads containing more than 0.1 % unknown bases (N) and low quality reads. Raw RNA-seq read counts for each treatment condition were used as input for DESeq2. Clean reads were mapped to the GRCh38 human reference genome using Bowtie2(31) (v2.2.5) and gene expression levels were estimated with RSEM(32) (v1.2.12).

Genes differentially expressed between LY2 siCDK12 vs siCtrl were determined using DESeq2(33). Adjusted *P*-value < 0.05 was used for comparative analysis. All genes with log2FC < 0 were considered to be downregulated in siCDK12, and all genes with log2FC > 0 were considered upregulated. Pathway enrichment analysis was performed using Enrichr(34).

### Chromatin immunoprecipitation (ChIP)

**ER ChIP-sequencing.** LY2 cells were seeded at 9 × 10⁴ cells per well in 6-well plates (2 mL complete media). After 24 h of serum deprivation in 4-OHT-free media, cells were transfected with siCDK12 or siCtrl. Following 48 h of transfection, cells were treated with 1 µmol/L β-estradiol for 1 h. Cells were cross-linked with 1% formaldehyde (F8775, Merck) for 10 min and quenched with 1 mol/L glycine (G7126, Merck). After PBS washes, cells were collected in PBS with protease inhibitors (04693124001, Roche) and centrifuged at 3000 g, 10 min, 4°C. Chromatin extraction was performed as described by Schmidt et al.(35). Chromatin was sonicated using a Bioruptor Pico (Diagenode) to ~200 bp fragments. Triton X-100 was added to a final concentration of 1%, followed by centrifugation at 20,000 g, 10 min, 4°C.

Protein A and G Dynabeads (10002D, 10003D, Fisher Scientific) were incubated with 5 µg antibody for 2 h at 4°C with rotation. Sonicated chromatin (50 µg) was incubated with antibody-coupled beads overnight at 4°C. Beads were washed 7× with RIPA buffer and once with 1× Tris-EDTA buffer (93283, Merck). Chromatin complexes were eluted in 200 µL elution buffer (10 mmol/L Tris-HCl pH 8, 1 mmol/L EDTA, 1% SDS), treated with RNase A (EN0531, 1 µL, 30 min at 37°C), and Proteinase K (5 µL, 03115828001, overnight at 65°C). DNA was purified using the MinElute PCR Purification Kit (28004, Qiagen). Experiments were conducted in triplicate (n = 3).

**CDK12, ER and MED1 ChIP-qPCR.** LY2 cells were seeded at 2.5 × 10^6^ cells per flask in T175 flask with 20 mL of complete media. After 24h of serum deprivation in 4-OHT-free media, cells were returned to complete media (4-OHT-free media) for an additional 24h. One hour prior to fixation, cells were treated with 1 µmol/L β-estradiol for 1 h. Chromatin immunoprecipitation was performed using the iDeal ChIP-seq kit for Transcription Factors (C01010170, Diagenode) according to the manufacturer’s protocol. Briefly, cells were first cross-linked with Diagenode ChiP Cross-link Gold (C01019027, Diagenode) for 30 minutes at room temperature, followed by 1% formaldehyde for 15 minutes. Cells were then washed twice with PBS, collected and Lysed. Chromatin was sonicated using a Bioruptor Pico (Diagenode) to ~200 bp fragments.

DiaMag protein A-coated magnetic beads (Diagenode) were incubated with 5 µg antibody for 4h at 4°C with rotation. Sonicated chromatin (200 µL) was incubated with antibody-coupled beads overnight at 4°C. Chromatin complexes were washed, eluted, and reverse cross-linked according to the manufacturer’s instructions. DNA was purified using iPure columns (Diagenode), and eluted DNA was used for downstream qPCR.

### ChIP-sequencing (ChIP-seq)

Library preparation and DNA sequencing (10 ng minimum DNA) were performed by BGI (Hong Kong). Sequencing was carried out on the BGISEQ-500 PE100 platform with 20 megabase (Mb) clean reads per sample. Experiment was performed in three replicates.

### ChIP-seq analysis

Raw sequencing data were processed to remove adapter sequences and low-quality reads using software SOAPnuke(30). Quality-filtered (clean) reads were analyzed using the nf-core ChIP-seq framework(36). Quality control for each sample was performed with FastQC (<https://www.bioinformatics.babraham.ac.uk/projects/fastqc/>) (v0.11.9). Reads (50 bp, single-end) were aligned to the GRCh38 human reference genome using BWA-MEM(37) (v0.7.17-r1188). Peak calling was performed using MACS2(38) (v2.2.7.1) in narrowPeak mode with a false discovery rate (FDR) threshold of 0.05.

For differential binding analysis, peak sets were imported into DiffBind(39,40) (v3.8.4), a Bioconductor R package. Quality and replicate consistency were assessed through principal component analysis (PCA) and sample clustering. One divergent replicate per condition was excluded from downstream analysis. A consensus peak set was defined by retaining peaks present in at least two replicates per condition, and peak summits were re-centered to create a binding affinity matrix.

Differentially bound sites (DBS) between siCDK12 knockdown and siCtrl conditions were identified using dba.analyze with the default DESeq2 method. Peaks with FDR < 0.05 were considered statistically significant. Genomic annotation of significant DBS relative to the nearest gene was performed using ChIPpeakAnno(41) (v3.32.0) with the UCSC hg38 gene annotation.

### ChIP-qPCR

ChIP-qPCR reactions were prepared using PowerUp SYBR Green Master Mix (A25741, Applied Biosystems), and performed on a StepOnePlus Real-Time PCR System (Applied Biosystems) with standard cycling conditions. ChIP-qPCR data was analyzed by fold enrichment over IgG.

Primer sequences (designed using Primer3Plus(29)):

*ABCA3* F: ACCCTGAGGTTTGGGAACAC, R: TCAAACACCTTCCATCTGTCC

*FKBP4* F: CGCTCGACTACAAATAGCC, R: TCACCATCTCTGATTCTCCC

*GREB1* F: CCCGTTTTGTCTCCTCTGTC, R: TTTTAAGCAGCCAGCAGC

*IGFBP4* F: GGAAAGTCAACAAACCACGG, R: CCAGACACACATTCATCCAC

*NRIP1* F: GCCCCCCAAAGAAGAAAAAG, R: GCCCTGTGACATTCAACAAC

### Protein extraction and immunoblotting

Whole-cell lysates were prepared using a lysis buffer containing 0.1% IGEPAL® CA-630 (I8896, Merck), 0.5% deoxycholic acid (D2510, Merck), and 0.1% SDS (L3771, Merck), freshly supplemented with protease and phosphatase inhibitors (78442, Fisher Scientific). For subcellular fractionation, cytoplasmic, nuclear, and chromatin-bound protein fractions were isolated using the Subcellular Protein Fractionation Kit for Cultured Cells (78840, Fisher Scientific) according to the manufacturer’s instructions. Nuclear protein extracts were obtained separately using the NE-PER™ Nuclear Extraction Kit (78835, Fisher Scientific).

Protein concentrations were determined using the Pierce™ BCA Protein Assay Kit (23227, Fisher Scientific). For immunoblotting, 30 µg of protein was loaded per lane and resolved on Bolt™ Bis-Tris Plus Mini 4–12% gradient gels (NW04125BOX, Invitrogen), followed by transfer onto nitrocellulose membranes (GE10600015, GE Healthcare). Proteins were detected using enhanced chemiluminescence (ECL) substrate (32106, Fisher Scientific) and imaged using the Amersham Imager 680 Imaging System (GE Healthcare). When appropriate, densitometric analysis was performed using ImageJ software. All experiments were conducted in triplicate (n = 3).

### Co-immunoprecipitation (co-IP) experiments

LY2 cells were seeded in 6-well plates at a density of 9 × 10⁴ cells per well in 2 mL of complete media. The following day, cells were serum-depleted for 24 h in 4-hydroxytamoxifen (4-OHT)-free media and transfected with 30 nmol/L siCDK12 or siCtrl for 48 h, as previously described. After transfection, cells were treated with 1 µmol/L β-estradiol (E4389, Merck) for 1 h in 4-OHT-free media. Nuclear proteins were extracted using the NE-PER™ Nuclear Extraction Kit (Thermo Fisher Scientific).

Endogenous co-immunoprecipitation was carried out using the Dynabeads™ Protein G Immunoprecipitation Kit (10007D, Fisher Scientific). Briefly, 300 µg of nuclear protein was pre-cleared by incubation with a 30 µL mixture of Protein A and G Dynabeads (10002D, 10003D, Fisher Scientific) for 1 hour at 4°C on a rotating platform. After pre-clearing, 5 µg of primary antibody was added to the lysates and incubated overnight at 4°C with rotation. The following day, 50 µL of fresh Protein A/G Dynabeads was added, and the samples were incubated for an additional 1 hour at 4°C.

Bead-bound antigen-antibody complexes were washed four times with IP washing buffer, and then resuspended in 20 µL of elution buffer and 20 µL of 2× sample buffer (S3401-1VL, Sigma-Aldrich). Samples were incubated at 70°C for 10 minutes, and then subjected to immunoblotting as described previously. All co-IP experiments were performed in triplicate (n = 3).

### Drug treatments

CDK12 small molecule inhibitors, CT7116, CT7311 and CT7349 were obtained from Carrick Therapeutics. Fulvestrant (S1191), Ribociclib (S5187) and THZ531 (S6595) were purchased from Selleckchem. *In-vitro* studies were performed using 300 nmol/L CT7116, 300 nmol/L THZ531, 100 nmol/L Fulvestrant, and 1 µmol/L Ribociclib. DMSO (0.01 %) was used as vehicle control.

Carrick Therapeutics compounds including CT7311, CT7116 and CT7439 are CDK12/13 inhibitors with a dual mechanism of action: inhibition of the kinase activity of CDK12 and CDK13, and molecular glue degradation of Cyclin K, the obligate co-factor for both kinases. THZ531 (Dana-Farber) is a first-generation, covalent CDK12/13 inhibitor that selectively targets Cys1039 on CDK12. While it has shown strong preclinical efficacy, it has poor pharmacokinetic properties, lacks oral bioavailability, and remains limited to preclinical research(42).

### Dose-response and cell viability assays

Cell viability in response to increasing concentrations of CDK12/13 inhibitors CT7116, CT7311, CT7349 and THZ531 (0.02–5 μmol/L), or control (0.01% DMSO) was assessed using the MTS assay (G1111, Promega). A total of 2.5 × 10³ cells per well (MCF7, LY2, LCC9, LETR, LY2 lung, LY2 bone, T47D and T347) were seeded in 96-well plates (100 μL/well). Cells were serum-depleted for 24 h prior to treatment and then incubated in complete media with compounds for 72 h. Absorbance at 495 nm was measured using a BioTek Synergy HTX Multi-Mode Microplate Reader (Agilent). Values were normalized to DMSO-treated controls, and IC50 (half maximal inhibitory concentration) values were calculated using nonlinear regression with a four-parameter variable slope model.

A fixed concentration of CT7116 (300 nmol/L) was used in subsequent *in vitro* studies, showing inhibitory effects in all models except the CDK12-mutant T347 cells (harboring the N885K mutation). Combination viability assays (combo treatments) with CT7116 (300 nmol/L), Fulvestrant (100 nmol/L), or Ribociclib (1 μmol/L) were conducted in LY2, LCC9, LY2 bone, and T347 cells under similar conditions. Results were normalized to DMSO controls. All experiments were performed in triplicate (n = 3).

### Colony Formation Assays

For colony formation assays, 3 × 10³ cells per well (LY2, LCC9, LY2 bone, T347) were seeded in 6-well plates (2.5 mL complete media/well). After 24 h, cells were treated with CT7116 (300 nmol/L), THZ531 (300 nmol/L), or vehicle (0.01% DMSO), with treatment refreshed every 2-3 days. Cells were incubated for 10 days (T347) or 13 days (other cell lines). Colonies were fixed in glacial acetic acid:methanol (1:7) for 5 minutes, stained with 0.1% crystal violet (C0775, Merck) for 60 minutes, washed with distilled water, and air-dried. Images were acquired using an HP Scanjet 7400c scanner, and colonies were quantified using ImageJ (v1.53k), with counts normalized to DMSO controls. All experiments were performed in triplicate (n = 3).

### Drug synergistic experiments

For synergy assays, LY2 and LY2 bone cells (2.5 × 10³ per well) were seeded in 96-well plates (100 μL/well). After 24 h of serum depletion, cells were treated in complete media for 72 h with CT7116 (10, 100, or 300 nmol/L), either alone or in combination with Fulvestrant (10, 50, or 100 nmol/L) or Ribociclib (100, 500, or 1000 nmol/L). Cell viability was measured using the MTS assay (G1111, Promega) as previously described. Values were normalized to DMSO-treated controls, and drug interaction effects were analyzed using SynergyFinder (43).

### Drug synergy analysis

Drug interaction analysis was performed using the SynergyFinder 3.0 web application. The Zero Interaction Potency (ZIP) model was applied to assess synergy between CT7116 and Fulvestrant, and between CT7116 and Ribociclib. This model assumes minimal changes in dose-response curves for non-interacting drugs. Three independent replicates per dose combination were uploaded in table format. The response type was set to inhibition, and dose-response curves for single agents were fit using the default four-parameter logistic regression. Baseline correction was applied to reduce the impact of outlier data points on synergy scoring. Synergy maps display dose-specific interactions, with red indicating *synergy* and green indicating *antagonism*. Summary ZIP synergy scores were interpreted as follows: < -10 = Antagonistic interaction; -10 to 10 = Additive interaction; > 10 = Synergistic interaction.

### Immunohistochemistry (IHC) for mouse xenografts

Tissues from PDX (Patient Derived Xenograft) models (T347pdx, T638pdx, HCl-011, HCl-005) were fixed in 4% formaldehyde for 48 h and paraffin-embedded. IHC was performed using the Rat, Rabbit, and Mouse HRP/DAB 1-Step Ready-to-Use Polymer Detection Kit (AGIB0466, Assay Genie) according to the manufacturer’s instructions. Briefly, 3 μm tissue sections were mounted on slides and incubated at 65 °C for 6 h. Sections were deparaffinized in xylene (534056, Merck) and rehydrated in industrial methylated spirits (IMS; CRTSI0331614, TE Laboratories). Antigen retrieval was performed by microwaving slides in sodium citrate buffer (pH 6) for 8 minutes at high power. Endogenous peroxidase activity was blocked for 10 minutes at room temperature, followed by protein blocking for 10 minutes. Slides were incubated with primary antibodies for 1 h at room temperature, followed by One-Step HRP Polymer for 30 minutes, and DAB chromogen for 10 minutes. Tissues were counterstained with hematoxylin (MHS16-500, Merck) for 3 minutes, dehydrated in IMS and xylene, and mounted with DPX (06522, Merck). Images were captured using Olympus CellSens software on a Leica DMI6000B microscope (20× objective). Images shown are representative of n = 3.

### Organoid cultures

ER-positive organoid models were derived from patient brain (T347, T638) and lung metastases (HCI-05, HCI-11), as previously described by Sachs et al. (44). Detailed model information is provided in Supplementary Table 6.

#### Organoid viability assay

Organoids were dissociated into single cells and seeded in organoid culture media supplemented with 5% Cultrex® Reduced Growth Factor Basement Membrane Matrix, type 2 (BME; 533-001-02, Trevigen). After 24 h, cells were treated with CT7116 (300 nmol/L) or vehicle control (0.1% DMSO). Viability was assessed 7 days post-treatment using the CellTiter-Glo® 3D Cell Viability Assay (G9682, Promega), with luminescence recorded at 1-second integration time using a Victor3 plate reader (Perkin Elmer). Values were normalized to vehicle controls. Experiments were performed in triplicate (n = 3).

3D Spheroid Assay
Three-dimensional (3D) spheroids were generated from LY2 and T347 breast cancer cell lines, following the protocol described by Jagust et al.(45). White 96-well plates were pre-coated with Cultrex® Reduced Growth Factor Basement Membrane Matrix, Type 2 (BME; 533-001-02, Trevigen), and 5 × 10³ cells per well were seeded in medium containing 5% BME. Spheroids were allowed to form over 7–9 days before treatment with CT7439 (300 nmol/L) or vehicle control (DMSO). Treatment was refreshed every 3 days. Cell viability was assessed on days 5, 7, and 10 post-treatment using the CellTiter-Glo® 3D Cell Viability Assay (G9682, Promega). Luminescence was measured using a Victor3 plate reader (Perkin Elmer) with a 1-second integration time and normalized to vehicle-treated controls. Images of spheroids were taken at each time point. All conditions were performed in triplicate (n = 3).

### *In vivo* study

The *in vivo* study was carried out with Axis Bio, Discovery Services (Northern Ireland). Protocols and procedures used in this study were approved by the Axis Bioservices Animal Welfare and Ethical Review Committee, under the guidelines of the Animal (Scientific Procedures) Act 1986. Female NOD SCID mice, bred in-house at Axis Bio, aged 5 – 8 weeks and weighing approximately 18 – 22 grams were used. The animals were housed in IVC cages (maximum 5 mice per cage) and each had individual tail markings. Bedding and water were sanitized before use and environmental enrichment was provided for nesting. The animals had free access to a certified diet and water which was replaced when necessary. Mice received β-estradiol (8 µg/mL) in their drinking water and water consumption was monitored. Cage changes took place on a weekly basis and weights were measured three times weekly. The holding room was maintained under standard conditions (20 – 24°C, 40 – 70 % humidity, and a 12 h light/dark cycle).

Mice were anesthetized with a ketamine/xylazine mixture followed by intra-cardiac injection of 1 × 10^5^ LY2-Luc cells (in PBS) over a 1-minute period into the left ventricle of the heart (day 0). Following cell injection, mice underwent bioluminescent imaging (BLI) (in both prone and supine position) using the In Vivo Imaging System (Newton 7.0). Prior to imaging (10 – 15 minutes) mice were injected with D-luciferin potassium salt (150 mg/kg). Anesthesia was maintained with a mix of isoflurane and oxygen gas throughout imaging. Thereafter, imaging was carried out once per week, including a final image prior to termination.

Treatment began 24 h post injection (day 1) and random allocation to treatment groups was assigned prior to dosing, vehicle (n = 5), CT7311 (n = 5). Dosing was administered via intraperitoneal (IP) injection on a schedule 5 on/2 off.

In dose studies CT7311 at 10 mg/kg (in 10 % DMSO; 90 % isotonic glucose 5 % w/v) was found to be outside the tolerable dose.

2 mg/kg CT7311 was administered, after three doses 10 % weight loss was observed. Treatment was paused until day 8 to encourage weight gain. When bodyweight had returned to less than 5 % loss compared to pre-treatment levels, three of five animals resumed treatment at 0.5 mg/kg. The final animal (# 6) resumed treatment on day 15. Following bioluminescent imaging at week 4, animal # 1 died under anesthesia. On day 35 post treatment, animal # 2 was terminated and sampled due to hind limb paralysis.

At termination (day 36), the remaining animals were euthanized via injection of a lethal overdose of pentobarbital. The brain, liver, lungs, and hind limbs were harvested and imaged *ex vivo*.

### Antibodies

#### Immunoblotting

Primary antibodies: anti-βactin (1:5000, A1978, Merck), anti-CDK12 (1:500, 11973, Cell Signaling Technology), anti-CDK13 (1:250, ABE1860, Sigma), anti-CCNK (1:3000, A301-939A, Bethyl Laboratories), anti-Cleaved-PARP (cPARP) (1:1000, 9541, Cell Signaling Technology), anti-ERα (ER) (1:1000, 06-935, Merck), anti-ERα (ER) (1:200, NCL-L-ER-6F11, Novocastra), anti-ERα (6F11), (1:200, MA1-80216, Invitrogen), anti-GAPDH (1:1000, MAB374, Merck), anti-Histone 3 (H3) (1:2000, 9715, Cell Signaling Technology), anti-LMNA (1:1000, MA1-06101, Invitrogen), anti-LMNB2 (1:1000, MAB3536, Merck), anti-MED1 (1:1000, A300-793A, Bethyl Laboratories), anti-Phospho MED1 (pMED1) (1:500, ab60950, Abcam), anti-RNA-pol II CTD (CTD) (1:1000, 2629, Cell Signaling Technology), anti-Phospho Ser2 CTD (pSer2 CTD) (1:1000, 13499, Cell Signaling Technology).

Secondary antibodies: anti-mouse IgG (7076, Cell Signaling Technology), anti-rabbit IgG (7074, Cell Signaling Technology).

#### Co-immunoprecipitation

anti-ERα (MA3-310, Invitrogen), anti-MED1 (A300-793A, Bethyl Laboratories), anti-rabbit IgG (C15410206, Diagenode), anti-mouse IgG (C15400001, Diagenode).

#### Chromatin immunoprecipitation

anti-ERα (06-935, Merck), anti-rabbit IgG (17-10530, Sigma), anti-MED1 (A300-793A, Bethyl Laboratories), anti-CDK12 (LS-A7350, LSBio), anti-rabbit IgG (C01010170, Diagenode).

#### Immunohistochemistry (patient derived xenografts)

anti-CDK12 (1:25, HPA008038, Sigma).

#### Tissue microarray (TMA)

anti-CDK12 (1:100, H00051755-M04, Abnova), anti-MED1 (1:500, A300-793A, Bethyl Laboratories).

### Statistical analysis

All statistical analyses were performed using GraphPad Prism (v10.1.0) or R studio. The following tests were applied as appropriate: **Log-rank (Mantel-Cox) test**: Survival analysis. **Benjamini-Hochberg**: Differential ER enrichment at ER binding sites (ER ChIP-seq). **Pearson correlation (r)**: CDK12 and MED1 gene expression correlation. **Cox Proportional-Hazards model**: Hazard ratios for death by MED1 and CDK12 expression. **Two-tailed paired *t*-test**: Densitometry of CDK12, MED1, ER, and pMED1 protein expression. **One-tailed paired *t*-test**: Densitometry of co-IP experiments. **One-tailed unpaired *t*-test**: ER and MED1 ChIP-qPCR assays. **Two-tailed unpaired *t*-test**: RT-qPCR of CDK12 gene expression, cell viability, colony formation, and organoid viability assays (using with Welch’s correction when appropriate). **Ordinary one-way ANOVA**: Drug combination treatment assays. **Two-tailed Mann-Whitney U test**: Animal whole-body and *ex vivo* organ bioluminescence imaging analyses. CDK12 gene expression in primary and brain metastatic patient tumors. Statistical significance thresholds and *P*-values are indicated in the corresponding figure legends.

## References

1. Cosgrove N, Varešlija D, Keelan S, et al. Mapping molecular subtype specific alterations in breast cancer brain metastases identifies clinically relevant vulnerabilities. *Nat Commun*. 2022;13(1):1-16. doi:10.1038/s41467-022-27987-5

2. Uhrig S, Ellermann J, Walther T, et al. Accurate and efficient detection of gene fusions from RNA sequencing data. *Genome Res*. 2021;31(3):448-460. doi:10.1101/GR.257246.119

3. Haas BJ, Dobin A, Li B, Stransky N, Pochet N, Regev A. Accuracy assessment of fusion transcript detection via read-mapping and de novo fusion transcript assembly-based methods. *Genome Biol*. 2019;20(1):1-16. doi:10.1186/s13059-019-1842-9

4. Heyer EE, Deveson IW, Wooi D, et al. Author Correction: Diagnosis of fusion genes using targeted RNA sequencing (Nature Communications, (2019), 10, 1, (1388), 10.1038/s41467-019-09374-9). *Nat Commun*. 2020;11(1):41467. doi:10.1038/s41467-020-15697-9

5. Gu Z, Eils R, Schlesner M. Complex heatmaps reveal patterns and correlations in multidimensional genomic data. *Bioinformatics*. 2016;32(18):2847-2849. doi:10.1093/bioinformatics/btw313

6. Charmsaz S, Doherty B, Cocchiglia S, et al. ADAM22/LGI1 complex as a new actionable target for breast cancer brain metastasis. *BMC Med*. 2020;18(1). doi:10.1186/S12916-020-01806-4

7. Giuliano AE, Edge SB, Hortobagyi GN. Eighth Edition of the AJCC Cancer Staging Manual: Breast Cancer. *Ann Surg Oncol*. 2018;25(7):1783-1785. doi:10.1245/S10434-018-6486-6,

8. Hammond MEH, Hayes DF, Dowsett M, et al. American Society of Clinical oncology/college of American Pathologists guideline recommendations for immunohistochemical testing of estrogen and progesterone receptors in breast cancer. *Arch Pathol Lab Med*. 2010;134(6):907-922. doi:10.5858/134.7.e48

9. Wolff AC, Hammond MEH, Hicks DG, et al. Recommendations for human epidermal growth factor receptor 2 testing in breast. *Journal of Clinical Oncology*. 2013;31(31):3997-4013. doi:10.1200/JCO.2013.50.9984,

10. Cerami E, Gao J, Dogrusoz U, et al. The cBio Cancer Genomics Portal: An open platform for exploring multidimensional cancer genomics data. *Cancer Discov*. 2012;2(5):401-404. doi:10.1158/2159-8290.CD-12-0095

11. Gao J, Aksoy BA, Dogrusoz U, et al. Integrative Analysis of Complex Cancer Genomics and Clinical Profiles Using the cBioPortal. *Sci Signal*. 2013;6(269):pl1-pl1. doi:10.1126/scisignal.2004088

12. de Bruijn I, Kundra R, Mastrogiacomo B, et al. Analysis and Visualization of Longitudinal Genomic and Clinical Data from the  AACR Project GENIE Biopharma Collaborative in cBioPortal. *Cancer Res*. 2023;83(23):3861-3867. doi:10.1158/0008-5472.CAN-23-0816

13. Curtis C, Shah SP, Chin SF, et al. The genomic and transcriptomic architecture of 2,000 breast tumours reveals novel subgroups. *Nature*. 2012;486(7403):346-352. doi:10.1038/NATURE10983

14. Pereira B, Chin SF, Rueda OM, et al. The somatic mutation profiles of 2,433 breast cancers refines their genomic and transcriptomic landscapes. *Nat Commun*. 2016;7. doi:10.1038/NCOMMS11479

15. Rueda OM, Sammut SJ, Seoane JA, et al. Dynamics of breast-cancer relapse reveal late-recurring ER-positive genomic subgroups. *Nature*. 2019;567(7748):399-404. doi:10.1038/s41586-019-1007-8

16. Lánczky A, Győrffy B. Web-Based Survival Analysis Tool Tailored for Medical Research (KMplot): Development and Implementation. *J Med Internet Res*. 2021;23(7):e27633. doi:10.2196/27633

17. Qin Q, Fan J, Zheng R, et al. Lisa: inferring transcriptional regulators through integrative modeling of public chromatin accessibility and ChIP-seq data. *Genome Biol*. 2020;21(1):1-14. doi:10.1186/S13059-020-1934-6/FIGURES/6

18. Tsherniak A, Vazquez F, Montgomery PG, et al. Defining a Cancer Dependency Map. *Cell*. 2017;170(3):564-576.e16. doi:10.1016/j.cell.2017.06.010

19. McDonald ER, de Weck A, Schlabach MR, et al. Project DRIVE: A Compendium of Cancer Dependencies and Synthetic Lethal Relationships Uncovered by Large-Scale, Deep RNAi Screening. *Cell*. 2017;170(3):577-592.e10. doi:10.1016/j.cell.2017.07.005

20. Marcotte R, Sayad A, Brown KR, et al. Functional Genomic Landscape of Human Breast Cancer Drivers, Vulnerabilities, and Resistance. *Cell*. 2016;164(1-2):293-309. doi:10.1016/j.cell.2015.11.062

21. McFarland JM, Ho Z V., Kugener G, et al. Improved estimation of cancer dependencies from large-scale RNAi screens using model-based normalization and data integration. *Nat Commun*. 2018;9(1):1-13. doi:10.1038/s41467-018-06916-5

22. Clarke R, Thompson EW, Leonessa F, et al. Hormone resistance, invasiveness, and metastatic potential in breast cancer. *Breast Cancer Res Treat*. 1993;24(3):227-239. doi:10.1007/BF01833263

23. Varešlija D, Ward E, Purcell SP, et al. Comparative analysis of the AIB1 interactome in breast cancer reveals MTA2 as a repressive partner which silences E-Cadherin to promote EMT and associates with a pro-metastatic phenotype. *Oncogene*. 2021;40(7):1318-1331. doi:10.1038/S41388-020-01606-3

24. Varešlija D, Mcbryan J, Fagan A, et al. Adaptation to AI therapy in breast cancer can induce dynamic alterations in ER activity resulting in estrogen-independent metastatic tumors. *Clinical Cancer Research*. 2016;22(11):2765-2777. doi:10.1158/1078-0432.CCR-15-1583

25. Varešlija D, Priedigkeit N, Fagan A, et al. Transcriptome Characterization of Matched Primary Breast and Brain Metastatic Tumors to Detect Novel Actionable Targets. *JNCI: Journal of the National Cancer Institute*. 2019;111(4):388-398. doi:10.1093/jnci/djy110

26. Van Der Meer D, Barthorpe S, Yang W, et al. Cell Model Passports - a hub for clinical, genetic and functional datasets of preclinical cancer models. *Nucleic Acids Res*. 2019;47(D1):D923-D929. doi:10.1093/NAR/GKY872,

27. Daemen A, Griffith OL, Heiser LM, et al. Modeling precision treatment of breast cancer. *Genome Biol*. 2013;14(10):1-14. doi:10.1186/GB-2013-14-10-R110/FIGURES/5

28. Bruna A, Rueda OM, Greenwood W, et al. A Biobank of Breast Cancer Explants with Preserved Intra-tumor Heterogeneity to Screen Anticancer Compounds. *Cell*. 2016;167(1):260-274.e22. doi:10.1016/j.cell.2016.08.041

29. Untergasser A, Nijveen H, Rao X, Bisseling T, Geurts R, Leunissen JAM. Primer3Plus, an enhanced web interface to Primer3. *Nucleic Acids Res*. 2007;35(suppl_2):W71-W74. doi:10.1093/NAR/GKM306

30. Chen Y, Chen Y, Shi C, et al. SOAPnuke: a MapReduce acceleration-supported software for integrated quality control and preprocessing of high-throughput sequencing data. *Gigascience*. 2018;7(1):gix120. doi:10.1093/gigascience/gix120

31. Langmead B, Salzberg SL. Fast gapped-read alignment with Bowtie 2. *Nat Methods*. 2012;9(4):357-359. doi:10.1038/nmeth.1923

32. Li B, Dewey CN. RSEM: accurate transcript quantification from RNA-Seq data with or without a  reference genome. *BMC Bioinformatics*. 2011;12:323. doi:10.1186/1471-2105-12-323

33. Love MI, Huber W, Anders S. Moderated estimation of fold change and dispersion for RNA-seq data with DESeq2. *Genome Biol*. 2014;15(12):1-21. doi:10.1186/s13059-014-0550-8

34. Kuleshov M V., Jones MR, Rouillard AD, et al. Enrichr: a comprehensive gene set enrichment analysis web server 2016 update. *Nucleic Acids Res*. 2016;44(W1):W90-W97. doi:10.1093/NAR/GKW377

35. Schmidt D, Wilson MD, Spyrou C, Brown GD, Odom DT. ChIP-seq : using high-throughput sequencing to discover protein-DNA interactions. *Methods*. 2009;48(3):240-248. doi:10.1016/j.ymeth.2009.03.001.ChIP-seq

36. Ewels P, Peltzer A, Fillinger S, et al. The nf-core framework for community-curated bioinformatics pipelines. Published online October 2022. doi:10.5281/zenodo.7139814

37. Li H, Durbin R. Fast and accurate long-read alignment with Burrows-Wheeler transform. *Bioinformatics*. 2010;26(5):589-595. doi:10.1093/bioinformatics/btp698

38. Zhang Y, Liu T, Meyer CA, et al. Model-based analysis of ChIP-Seq (MACS). *Genome Biol*. 2008;9(9):R137. doi:10.1186/gb-2008-9-9-r137

39. Rory S, Gord B. DiffBind: Differential binding analysis of ChIPSeq peak data. Published online 2021:1-73.

40. Ross-Innes CS, Stark R, Teschendorff AE, et al. Differential oestrogen receptor binding is associated with clinical outcome in breast cancer. *Nature*. 2012;481(7381):389-393. doi:10.1038/nature10730

41. Zhu LJ, Gazin C, Lawson ND, et al. ChIPpeakAnno: a Bioconductor package to annotate ChIP-seq and ChIP-chip data. *BMC Bioinformatics*. 2010;11:237. doi:10.1186/1471-2105-11-237

42. Zhang T, Kwiatkowski N, Olson CM, et al. Covalent targeting of remote cysteine residues to develop CDK12 and CDK13 inhibitors. *Nat Chem Biol*. 2016;12(10):876-884. doi:10.1038/nchembio.2166

43. Ianevski A, Giri AK, Aittokallio T. SynergyFinder 3.0: an interactive analysis and consensus interpretation of multi-drug synergies across multiple samples. *Nucleic Acids Res*. 2022;50(W1):W739-W743. doi:10.1093/nar/gkac382

44. Sachs N, de Ligt J, Kopper O, et al. A Living Biobank of Breast Cancer Organoids Captures Disease Heterogeneity. *Cell*. 2018;172(1-2):373-386.e10. doi:10.1016/J.CELL.2017.11.010

45. Jagust P, Powell AM, Ola M, et al. RET overexpression leads to increased brain metastatic competency in luminal breast cancer. *JNCI: Journal of the National Cancer Institute*. 2024;116(10):1632-1644. doi:10.1093/JNCI/DJAE091
